# Mechanism of Fructus Mume Pills Underlying Their Protective Effects in Rats with Acetic Acid-Inducedulcerative Colitis via the Regulation of Inflammatory Cytokines and the VEGF-PI3K/Akt-eNOS Signaling Pathway

**DOI:** 10.1155/2022/4621131

**Published:** 2022-05-02

**Authors:** Zongying Xu, Xueli Zhang, Ruimin Lu, Di Zhang, Tianyuan Zou, Meng Chen, Dongmei Zhang

**Affiliations:** ^1^College of Chinese Medicine, Beijing University of Chinese Medicine, Beijing, China; ^2^Dongzhimen Hospital, Beijing University of Chinese Medicine, Beijing, China

## Abstract

**Background:**

Fructus mume pills (FMPs) have been clinically proven to be effective for treating ulcerative colitis (UC). However, the therapeutic and protective mechanisms have not been fully studied.

**Aim:**

We aimed to explore the mechanism of FMPs in an acetic acid (AA)-induced ulcerative colitis rat model.

**Methods:**

The targets, GO terms, and KEGG pathways for the FMPs and UC were screened and constructed using network pharmacology. A possible mechanism was verified in a 4% AA-induced colitis rat model. Colitis activity and state were evaluated using the disease activity index, and colon ulceration and intestinal mucosal damage were determined by histopathological observation through HE, AB-PAS, and Masson pathological staining. The concentrations of TNF-*α*, IL-6, IL-8, IL-10, MPO, MMP9, CXCR1, eNOS, and VEGF were measured to evaluate vascular permeability effects.

**Results:**

The network pharmacology results showed 108 active compounds, and 139 FMP-related targets were identified. Twenty-nine targets were identified for FMPs against UC, which included MMP9, MMP3, ESR1, PTGS1, PPARA, MPO, and NOS2. A total of 1,536 GO terms and 41 pathways were associated with FMP treatment of UC. The pharmacological evaluation showed that FMPs attenuated inflammation in AA-induced colitis by reducing the serum concentrations of TNF-*α*, IL-6, IL-8, and IL-10 and the colonic concentrations of MPO, MMP9, and CXCR1. FMPs ameliorated hyperpermeability by reducing the colonic VEGF and eNOS concentrations. FMPs also significantly decreased the VEGFA, VEGFR2, Src, and eNOS protein expressions in colon tissue through the VEGF-PI3K/Akt-eNOS signaling pathway.

**Conclusion:**

These results suggest that FMPs control UC inflammation by regulating inflammatory cytokine concentrations. FMPs alleviate AA-induced UC by regulating microvascular permeability through the VEGF-PI3K/Akt-eNOS signaling pathway.

## 1. Introduction

Ulcerative colitis (UC) is a chronic nonspecific refractory inflammatory colon disease with typical clinical symptoms of diarrhea, mucopurulent bloody stool, rectal bleeding, abdominal pain, and weight loss [1]. It is associated with major morbidities in the Western countries [2], and its incidence continues to increase, which is approximately between 0.3/10^6^ and 2/10^6^ in China [3]. A report showed an increased risk of colorectal cancer in up to 30% of affected patients after 35 years of UC [4]. Multiple treatment pathways are often required to facilitate long-term survival. Evidence suggests that both traditional treatment and bioinhibitors have a considerable impact related to costs to society, health systems, and individuals [5].

The risk of UC is associated with genetic predisposition, epithelial barrier defects, dysregulated immune responses, and environmental factors. Inflammation is crucial in determining whether mucous colitis is mitigated or exacerbated [6]. The loss of immune tolerance leads to a persistent imbalance in the concentrations of proinflammatory cytokines such as TNF-*α*, IL-6, and IL-8, as well as those of the anti-inflammatory cytokine IL-10 [7]. These perturbations contribute to chronic inflammation and colon tissue damage associated. with UC.

The herbal formulas of traditional Chinese medicine (TCM) have the advantages for individual treatment. It is characterized as “multicomponent and multichannel,” which allows the treatment of complex diseases. Fructus mume pills (Wumei Wan, FMPs) have been recommended for treating ascarides and chronic diarrhea in China since A.D. 200. FMPs have been used to treat various digestive diseases, such as enteritis, irritable bowel syndrome, diarrhea, and colitis [8]. Based on the original work of Treatise on Febrile Disease Caused by Cold (Shanghan Lun), FMPs contain 10 herbs, including 25 g of Fructus mume (Wumei, WM), 10 g of Rhizoma Zingiberis (Ganjiang, GJ), 16 g of Phizoma Coptidis (Huanglian, HL), 6 g of Herba Asari (Xi Xin, XX), 4 g of Radix Angelicae Sinensis (Danggui, DG), 6 g of Radix Aconiti Praeparata (Fuzi, FZ), 4 g of Pericarpium Zanthoxyli (Shujiao, SJ), 6 g of Ramulus Cinnamomi (Guizhi, GZ), 6 g of Radix Codcnopsitis Pilosulas (Dangsheng, DS), and 6 g of Cortex Phellodendri (Huangbo, HB) [9]. Studies indicate that FMPs effectively protect against UC by regulating the imbalance of inflammatory cytokines, improving analgesia, preventing oxidative stress, inhibiting Bcl-2/Bax expression [10], and restoring the balance of the intestinal bacterial population [11]. However, the chemical and pharmacological foundations for the treatment of UC with FMPs have not been globally evaluated using appropriate approaches.

Network pharmacology is a cross-discipline based on systems biology, which combines polypharmacology, molecular network data, bioinformatics, and computer simulation [12] that have revealed molecular phenotypic robustness and a network structure [13]. The type of compound-protein/gene-disease network construction facilitates the analysis of multitargeted agents in complex TCM prescriptions [14].

FMPs have been widely adopted for UC treatment, but their active compounds, potential targets, and molecular mechanisms are not known. Hence, we combined network pharmacology with experimental pharmacology to evaluate the possible protective effects of FMPs via an FMP-UC network pharmacology study and verified the potential mechanism of FMPs against 4% acetic acid (AA)-induced UC in rats.

## 2. Materials and Methods

### 2.1. Network Pharmacology Analyses

#### 2.1.1. Collection of FMP Compounds and Targets

Compounds of the ten herbs in FMPs were collected from the Traditional Chinese Medicine Systems Pharmacology Database and Analysis Platform [15] (TCMSP, http://lsp.nwu.edu.cn/tcmsp.php). Two important in silico ADME indexes, oral bioavailability (OB) of ≥30% and drug-likeness (DL) of ≥0.18, were used to screen the candidate active compounds. OB was used to determine the rate and extent of absorption of active pharmaceutical ingredients in herbs, and OB of ≥30% was generally considered to indicate that these lead compounds in oral medicines may have good utilization and development value. The DL value was based on the physical and chemical properties of the compounds and topological structures, and DL of ≥0.18 suggested that the lead compounds had the potential to become drugs [16].

The compound-related targets of FMPs depend on their chemical structures. We collected the SMILE forms of compounds from PubChem [17] (https://pubchem.ncbi.nlm.nih.gov/) and predicted potential targets from the “Homo sapiens” species with a *p*-value of ≥0.5 in SwissTargetPrediction [18, 19] (http://www.swisstargetprediction.ch/, updated in 2019). The target proteins were selected using the criteria for the “Homo sapiens” species and a confidence score of ≥0.7 in the STITCH database [20] (http://stitch.embl.de/, version 5.0).

#### 2.1.2. Collection of UC Targets

The acknowledged UC-related targets were collected from Genecards [21] (https://www.genecards.org/, vision 4.13), the Therapeutic Targets Database [22] (TTD, http://db.idrblab.net/ttd/), and the DisGeNET platform [23] (http://www.disgenet.org/, updated on May 13, 2019). The standard target names were identified using the Gene ID conversion tool from the Database for Annotation, Visualization, and Integrated Discovery [24] (https://david.ncifcrf.gov/home.jsp, DAVID 6.8).

#### 2.1.3. Construction and Analyses of FMP-UC Networks

The shared targets of the FMPs and UC were obtained using the Venny 2.1.0 online tool [25]. These target protein-protein interactions (PPIs) were acquired from the STRING database (https://string-db.org/cgi/input.pl, version 11.0). The minimum required interaction score for the PPIs was ≥0.7 [26]. All nets were constructed using Cytoscape v3.7.1 [27] and analyzed using the topological method. The important targets were evaluated; degree centrality (DC), betweenness centrality (BC), closeness centrality, DC ≥ 2 × median DC, BC of ≥ median BC, and closeness centrality of ≥ median closeness centrality were considered key nodes.

#### 2.1.4. Gene Ontology (GO) and Kyoto Encyclopedia of Genes and Genomes (KEGG) Pathway Enrichment Analyses

Based on the above analyses, GO analysis and KEGG pathway enrichment analyses were performed to further understand the mechanism of FMPs for the treatment of UC. The enrichment results with a *p* value of <0.05 were regarded as statistically significant.

### 2.2. Experiment Verification

#### 2.2.1. Materials and Reagents

The FMPs were obtained from Sichuan Neautus Traditional Chinese Medicine Co., Ltd. (Sichuan, China). AA (AR, 99.7%) was obtained from Alfa Aesar (China) Chemical Co., Ltd. AA was diluted with 0.9% saline to a final concentration of 4%. Pentobarbital sodium salt (98%) was obtained from Sigma-Aldrich (St. Louis, MO, USA), dissolved in deionized water, and configured at a 3% concentration. Fecal occult blood-testing kits (orthotolidine method) were obtained from Shanghai Yuanye Bio-Technology Co., Ltd. (Shanghai, China). Myeloperoxidase (MPO) was assayed using commercial test kits from the Nanjing Jiancheng Bioengineering Institute (Nanjing, China). Interleukin-6 (IL-6), interleukin-10 (IL-10), interleukin-8 (IL-8), C-X-C chemokine receptor type 1 (CXCR1), matrix metalloproteinase-9 (MMP9), and vascular endothelial growth factor (VEGF) were obtained from Biolianshuo Co., Ltd. (Shanghai, China); tumor necrosis factor-*α* (TNF-*α*) kits were obtained from RayBiotech Inc. (Peachtree Corners, USA), and endothelial nitric oxide synthase (eNOS) ELISA kits were purchased from CLOUD-CLONE CORP. (Houston, USA). A BCA Protein Assay Kit (200T) and anti-*α*-tubulin rabbit pAb were purchased from Beyotime Co., Ltd. (Shanghai, China). Anti-VEGFA rabbit pAb, anti-Akt rabbit pAb, anti-Src rabbit pAb, anti-eNOS rabbit pAb, anti-GAPDH rabbit pAb, anti-beta actin rabbit pAb, and HRP-conjugated goat anti-rabbit IgG were obtained from Sevier Biological Technology Co. (Wuhan, China). Anti-PI3K rabbit pAb was obtained from BIOSS Biological Technology Co. (Beijing, China), and anti-VEGF receptor 2 (VEGFR2) rabbit pAb was obtained from Boster Biological Technology Co., Ltd. (Beijing, China). Other chemicals were of analytical grade and were purchased from commercial sources.

#### 2.2.2. FMP Aqueous Extract Preparation and Quality Control

To simulate the medicinal and pill production methods, aqueous high-dose FMPs (H-FMPs) were prepared as follows: 900 mg of FMP powder was accurately weighed and placed in 10 ml of ultrapure water and heated in a 100°C water bath for 60 min, and the mass lost was replenished by adding an infinitesimal amount of ultrapure water. Aqueous low-dose FMPs (L-FMPs) were prepared using the same method with 450 mg of FMPs in 10 ml of water. The prepared aqueous FMP solution was stored at 4°C.

The quality of the FMPs was analyzed using a Waters e2695 Ultra performance liquid chromatograph (UPLC), Waters 2489 ultraviolet detector, and Waters 2998 PAD detector (Milford, USA). Please refer to the supplementary materials for the specific methods (Supplement 1).

#### 2.2.3. Animals and Treatment Design

A total of 43 male 220 ± 20 g SD rats were purchased from SPF (Beijing) Biotechnology Co., Ltd. (Certification number SCXK-JING 2020–0033). All animals were allowed to acclimate for 1 week before the experiment and kept at 25 ± 2°C with 50% humidity and under 12 : 12 h light:dark cycle lighting conditions in an SPF feeding room, and water and food were provided ad libitum. This research adhered to the guidelines of experimental animal ethics and was approved by the Ethics Committee (BUCM-4-2020092905–3119).

The rats were randomly divided into four groups: each of the control, high-dose FMP (H-FMP), and low-dosage FMP (L-FMP) groups comprised 10 rats, and the model group comprised 13 rats. All rats fasted for 12 h before modeling. According to a previous study, an enema with 4% AA was used to induce the UC rat model [28]. Anesthesia was induced by intraperitoneal injection of 3% pentobarbital sodium (30 mg/kg). An enema tube (2 mm × 10 cm) was inserted through the anus for up to approximately 8 cm; it was infused with 2 ml of 0.9% saline and colonic content was extracted to decrease fecal interference. Two milliliters of 4% AA solution was infused with a new tube, and the rat buttocks were elevated to prevent AA leakage. The AA solution was retained for 2 min and extracted. To reduce anal tissue damage, the anus and surrounding skin were gently wiped dry. Rats in the control group received 2 ml of saline instead of AA. After 24 h, three rat models were randomly selected to check whether the UC model was successfully constructed.

**Figure 1:**
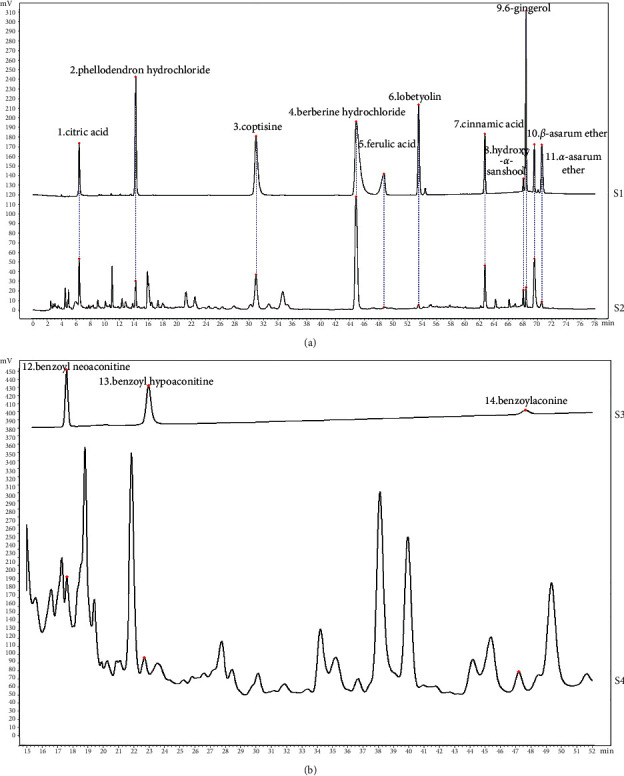
UPLC profiles of standards and FMP aqueous extract. (a) Chromatograms of citric acid, phellodendron hydrochloride, coptisine hydrochloride, berberine hydrochloride, ferulic acid, lobetyolin, cinnamic acid, hydroxy-*α*-sanshool, 6-gingerol, *ß*-asarum ether, and *a*-asarum standards under 285 nm detection wavelength. S1: standard chromatograms, S2 : FMP chromatograms. (b) Chromatograms of benzoylaconine, benzoyl neoaconitine, and benzoyl hypoaconitine standards under 235 nm detection wavelength. S3: standard chromatograms, S4 : FMP chromatogram.

On the ninth day, rats in the H-FMP group were orally administered 900 mg/kg of FMPs, and rats in the L-FMP group were orally administered 450 mg/kg of FMPs once a day. The dose selection for the FMPs was based on the clinical therapeutic dosage that a 60-kg patient would take an 8–10 g FMP every day. The control and model groups were administered the same volumes of saline. A schematic of the experimental animal design is shown in Figure 1(a) and 1(b).

#### 2.2.4. Sample Collection

After the last administration, the rats fasted for 12 h before sacrifice. Blood samples were collected from the inferior vena cava, and the distal 8 cm of the colon tissue was removed immediately after sacrifice. Serum was collected by centrifugation at 3,500 rpm for 10 min and stored at -80°C. One portion of colon tissue was stored in 4% paraformaldehyde for fixation, and another portion was stored at -80°C for detection.

#### 2.2.5. Disease Activity Index (DAI)

The DAI in this study was observed every other day to dynamically study rat colon damage changes and FMP protective effects. The DAI assessment scores included weight loss, stool consistency, and stool bleeding, and were calculated based on the average score of these three parts [29]. Diarrhea was evident by the mucus on feces stuck to animal fur, while rectal bleeding ranged from occult blood to gross bleeding. Rectal bleeding was detected using fecal occult blood test kits. Bodyweight loss was scored as follows: no bodyweight loss, 0 points; loss of 1–5%, 1 point; loss of 5–10%, 2 points; loss of 10–15%, 3 points; and loss of >15%, 4 points. Normal stool was scored as 0 points, soft sticky stool was scored as 2 points, and loose stool was scored as 4 points. Blood in stool was scored as follows: bleeding stool, 0 points; occult blood (+), 1 point; occult blood (++), 2 points; occult blood (+++), 3 points; and gross blood, 4 points.

#### 2.2.6. Colon Damage Observation

Colon damage was evaluated using gross pathology and histopathology. A microscopic assessment was performed using a magnifying lens. Histological samples were prepared from three or four paraffin-embedded sections (4–5 *μ*m thick) per specimen, routine dewaxing, hematoxylin and eosin (H&E) staining, Alcian blue-periodic acid Schiff (AB-PAS), and Masson staining. The stained sections were examined under an Olympus BX53 microscope (Tokyo, Japan).

#### 2.2.7. Detection of Cytokines

The serum concentrations of IL-6, IL-10, TNF-*α*, and IL-8 were determined using double-antibody sandwich enzyme-linked immunosorbent assay (ELISA) kits according to the manufacturer's instructions. The plate was placed into a microplate analyzer (Bio-Rad, California, USA) and the optical density (OD) values were read at 450 nm. The concentration is directly proportional to the OD value. The concentrations in the samples were calculated using a standard curve.

The colonic MMP9, CXCR-1, VEGF, and eNOS concentrations were detected using ELISA kits. The colon supernatant samples were collected from the colon tissue lysis solution. The colon tissue samples (0.1 g) were homogenized with the corresponding tissue lysis buffer (1 : 20 w/v) and centrifuged at 10,000 × *g* for 5 min, and the colon tissue supernatant was collected as the sample. The rest of the steps were the same as those for serum cytokine detection.

#### 2.2.8. MPO Assay

According to the kit instructions, 0.1 g of colon tissue was homogenized with 1.9 ml of reagent 2 to prepare 5% tissue homogenate, and 0.5 ml of the homogenate was added to 0.1 ml of reagent 3. The test samples were mixed thoroughly and heated in a 37°C water bath for 15 min. The centrifugal rotational speed settings were based on the manufacturer's instructions. Following the manual, the samples were detected at 460 nm using a spectrophotometer (UV 2600, Shimadzu).

#### 2.2.9. VEGF Signaling Pathway Assay

The colonic protein concentrations of VEGFA, VEGFR2, Src, PI3K, Akt, and eNOS were detected by western blotting (WB). Colon tissue (0.1 g) was lysed in 1 ml of RIPA lysis buffer containing phenylmethanesulfonyl fluoride. Approximately 100 or 150 *μ*g of protein was separated on SDS-PAGE gels and transferred to polyvinylidene fluoride (PVDF) membranes. After blocking with QuickBlock™ Buffer at room temperature for 30 min, the PVDF membranes were incubated for 12 h with the corresponding primary antibodies at 4°C. Primary antibodies against VEGF, Akt, and Src were diluted to 1 : 1000, and eNOS, PI3K, and VEGFR2 were diluted at 1 : 500. The PVDF membranes were washed three times with 1× TBST for 10 min. The PVDF membranes were incubated with a secondary antibody (1 : 3000) for 1 h at room temperature. The PVDF membranes were washed with 1× TBST for 10 min three times, and the ECL agent was immediately applied to the surface of the PVDF membranes for signal detection on a chemiluminescence instrument (CLINX 6300, Shanghai, China). The image grey values were processed using Photoshop 2020 (Adobe, California, USA).

### 2.3. Statistical Analysis

The data were analyzed using SPSS (version 22.0; IBM, New York, USA) and Origin 2018 software (OriginLab, Northampton, USA). The measurement data are presented as the mean ± standard deviation (‾x ± s). Gaussian distribution was analyzed using the Shapiro–Wilk results, *p* values for the Shapiro–Wilk results of >0.05, and judged Gaussian data. Analysis of variance (ANOVA) was used to analyze the data among groups that were consistent with a Gaussian distribution. Non-Gaussian data were analyzed using the Kruskal–Wallis test. The differences were considered statistically significant at *p* < 0.05, and *p* < 0.001 was considered statistically significant. A corresponding histogram of the results was derived using Origin 2018.

## 3. Results

### 3.1. FMP Quality Control Results

**Figure 2:**
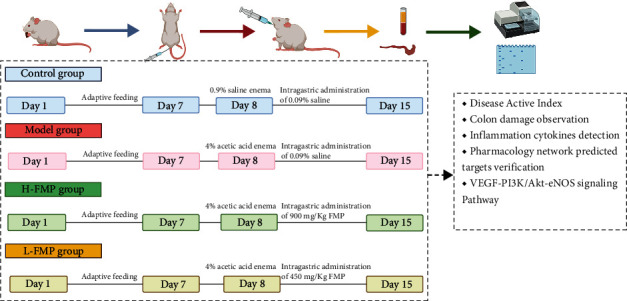
Experiment process design. Control, model, H-FMP, and L-FMP groups were set up on day 1, UC rat model was established with 4% acetic acid on day 8, and FMP administration lasted for seven days. Pharmacological evaluation of FMP was conducted using the disease activity index, colon histopathological changes, UC basic pathological mechanisms, and network pharmacology results for predicted targets and pathways.

The UPLC results showed that the retention durations of citric acid, phellodendron hydrochloride, coptisine hydrochloride, berberine hydrochloride, ferulic acid, lobetyolin, cinnamic acid, hydroxy-*α*-sanshool, 6-gingerol, *ß*-asarum ether, and *a*-asarum ether in FMPs were 6.54 min, 14.21 min, 30.83 min, 44.74 min, 48.86 min, 53.58 min, 62.64 min, 68.20 min, 68.38 min, 69.60 min, and 70.56 min, respectively. The retention durations were consistent with those of the standard reference substances and the absorption waves were consistent with those of the standard reference substances (Figure 2). The content of citric acid was approximately 56.47 mg/g and the RSD was 2.97%. The retention durations of benzoyl neoaconitine, benzoyl hypoaconitine, and benzoylaconine in the FMPs were 17.51 min, 22.81 min, and 47.2 min, respectively, at a wavelength of 235 nm (Figure 2).

### 3.2. Herb-Compound-Target Network of the FMPs

After eliminating duplicates, 108 active compounds were identified in the FMPs from the TCMSP database and 139 compound-based targets were obtained from the databases. The compounds and targets obtained from the herbs are as follows: WM, 8 active compounds and 104 targets; HB, 36 compounds and 113 targets; HL, 14 compounds and 103 targets; DS, 21 compounds and 49 targets; DG, 2 active compounds and 7 targets; GJ, 5 compounds and 8 targets; GZ, 7 compounds and 8 targets; SJ, 5 compounds and 96 targets; XX, 8 compounds and 43 targets; FZ, 21 compounds and 13 targets (Supplement 2).

**Figure 3:**
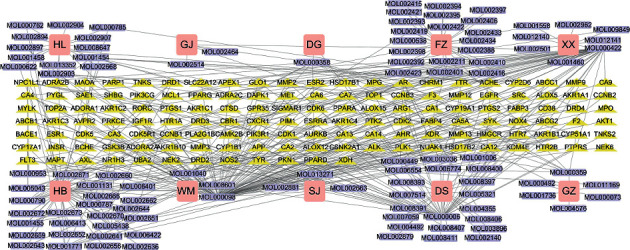
Herb-compound-target network for FMP. The FMP herb-compound-target network contained 10 herbs, 108 compounds, and 139 targets. The herbs are depicted as pink squares. The purple rectangle shapes represent compounds. The yellow triangle shapes represent FMP targets. The lines represent the relationship between the compounds and target nodes.

The herb-compound-target network of the FMPs was constructed from these 108 active compounds and 139 targets (Supplement 2). This network contained 251 nodes and 406 edges. In this network, quercetin, luteolin, kaempferol, beta-sitosterol, stigmasterol, berberine, (2R)-5,7-dihydroxy-2-(4-hydroxyphenyl)chroman-4-one, campesterol, poriferast-5-en-3beta-ol, and isocorypalmine were the top 10 most important active compounds based on topological analysis. This suggests that these ten compounds play the most vital roles in FMPs. Furthermore, AR, CYP19A1, HMGCR, CYP51A1, NPC1L1, ACHE, NR1H3, ABCC1, and CYP1B1 were identified as important targets (Figure 3).

### 3.3. FMP-UC Targets Network

**Figure 4:**
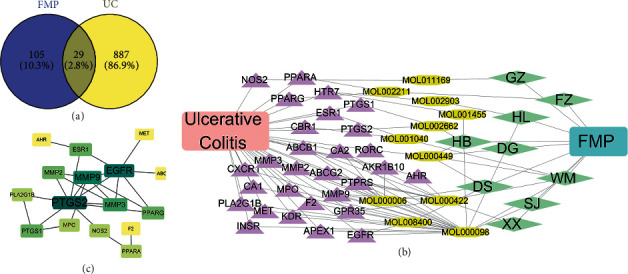
FMP-UC target network. (a) Distribution of FMP and UC targets. (b) FMP-compound-target-UC network, purple triangles representing shared targets between FMP and UC, Earth yellow octagons representing FMP compounds, and grass green representing herbs. (c) FMP-UC PPI network.

From the DisGeNET, Genecards, and TTD databases, 916 UC-related targets were identified (Supplementary 3). Twenty-nine targets were shared between FMPs and UC identified by the Venny 2.0 online tool: HTR7, ABCB1, GPR35, F2, PPARG, PPARA, PTGS2, EGFR, PTGS1, APEX1, RORC, MET, KDR, PTPRS, MPO, CA1, CA2, MMP2, MMP3, MMP9, PLA2G1B, AHR, INSR, AKR1B10, CBR1, CXCR1, ABCG2, ESR1, and NOS2 (Figure 4(a)). As shown in Figure 4(b), the FMP-UC network had 51 nodes and 100 edges. Topology analysis indicated that MMP9, ABCB1, PTPRS, CA2, MMP2, ABCG2, and EGFR were pivotal targets in the FMP-potential target-UC target network.

Twenty-nine targets revealed 25 PPIs and 16 targets were involved in the FMP-UC PPI network that could be co-expressed or interacted with each other. These 16 targets were suggested to have strong interactions. In Figure 3(c), the higher the degree value of the target, the darker the color shown and the more proteins it connected. The top five targets were MMP9, MMP3, ESR1, PTGS1, and PPARA. These targets are involved in oxidative stress, adhesion, inflammation, repair and reconstruction, endothelial growth, mass transfer, and neurotransmitter response, among other processes.

### 3.4. GO and KEGG Enrichment Results

**Figure 5:**
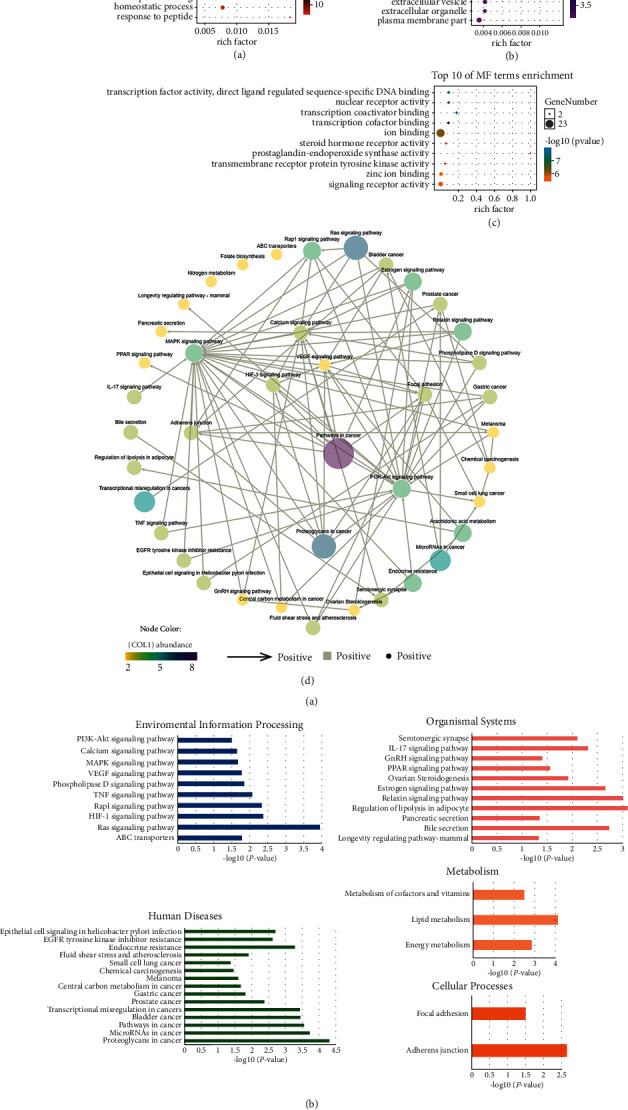
GO and KEGG enrichment results. (a) Top 10 BP enrichment. (b) Top 10 MF enrichment. (c) Top 10 CC enrichment. (d) KEGG pathway enrichment. This graph represents the interaction between enrichment paths, and the arrows indicate the upstream and downstream signal relationships between the pathways. (e) KEGG pathway distribution diagram.

During GO analysis, we obtained 1,536 GO terms enriched in biological processes (BPs), molecular function (MF), and cellular component (CC) (Supplement 4). The top 10 BP terms contained cellular responses to oxygen-containing compounds, responses to oxygen-containing compounds, and cellular responses to chemical stimuli, among others. (Figure 5(a)). The top 10 MF terms included “transcription factor activity,” “direct ligand-regulated sequence-specific DNA binding,” and “nuclear receptor activity” (Figure 5(b)). The top 10 CC terms included “extracellular region,” “vesicle,” and “receptor complex” (Figure 5(c)).

KEGG enrichment analysis showed that these 29 targets contributed to 41 pathways (*p* < 0.05), including pathways for cancer, epithelial cell signaling in *Helicobacter pylori* infection, VEGF signaling pathway, and adherence junction (Supplement 5). These 41 pathways were closely linked to the protective effects of FMPs against conditions related to UC, including inflammation, permeability, adherence, and cancer (Figure 5(d), (e)).

### 3.5. Effect of FMPs on Symptoms of AA-Induced UC

**Figure 6:**
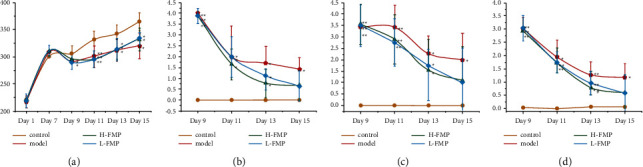
Dynamic changes in the characteristic manifestations of each group of rats. (a) Bodyweight changes. (b) Rectal blood changes. (c) Diarrhea changes. (d) DAI score. ^*∗∗*^*p* < 0.001 compared with the control group, ^*∗*^*p* < 0.05 compared with the control group, #*p* < 0.05 compared with the control group.

The DAI score reflects the progression and recovery of UC. After treatment with 4% AA, all rats showed rectal bleeding, diarrhea, and various degrees of weight loss on day 9. The DAI scores of the H-FMP and L-FMP rats on day 9 were significantly increased, suggesting that UC was successfully induced in rats (*p* < 0.001). Over the course of the experiment, on day 15, the bodyweights of the model rats significantly decreased (*p* < 0.05), and diarrhea and rectal bleeding were detected (*p* < 0.05). The DAI scores of the model rats and control rats were significantly different (*p* < 0.05), and H-FMP and L-FMP treatment eased the symptoms of the defecation syndromes (*p* < 0.05). The DAI scores of the H-FMP and L-FMP groups were lower than those of the model group (*p* < 0.05). The dynamic changes in the syndrome are shown in Figure 6. Over the course of the experiment, three rats were sacrificed in the model group, one in the H-FMP group and two in the L-FMP group.

### 3.6. Effects of FMPs on Histopathologic Changes

**Figure 7:**
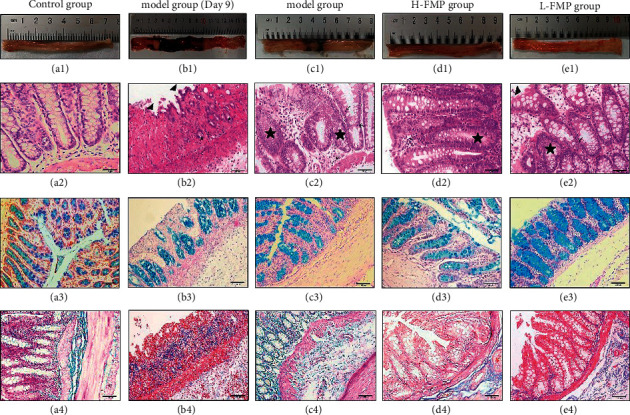
FMP relieved the histopathologic damage of AA-induced UC rats. (A1–E1) Macroscopic pathologic observations by the naked eye. (A2–E2) H&E pathological staining of colon tissue from each group of rats. Arrow symbols indicate the infiltration of neutrophils and mononuclear inflammatory cells; triangle symbols show the disappearance, distortion, and exfoliation of epithelial tissue; pentagram symbols represent crypt structure changes. (A3–E3) AB-PAS pathological staining of colon tissue from each group of rats; in AB-PAS staining, various glycoproteins of glycogen neutral mucins were purple-red. Acidic mucins, proteoglycans, and hyaluronic acid were blue. (A4–E4) Masson pathological staining colon tissue from each group of rats. Fibrous tissue was stained blue, and the cells, cytoplasm, erythrocytes, muscle tissue, eosinophilic granules, and connective tissue were stained red.

In the control group, the colon tissue maintained a non-diseased state and a smooth surface without any incrassation or congestion (Figure 7(a)1). After AA enema, the rat colon showed marked local swelling, congestion, and anabrosis, and the lesion area was extended over 2 cm^2^, which suggested that the models were successfully induced (Figure 7(b)1). At the end of the experiment, the rat colon in the model group still showed visible signs of local congestion, swelling, incrassation, and anabrosis, although the lesion area was reduced (Figure 7(c)1). In the two-dosage FMP treatment groups, the colonic mucosa exhibited only a small amount of local congestion and the colon tissue was essentially restored (Figure 7(d)1, 7(e)1).

H&E staining results indicated that the colonic epithelium structure of the control group remained intact and the crypts were well-organized. The submucosal and muscular structures were clear and the inner circular and outer longitudinal muscle layers were arranged neatly (Figure 7(a)2). After modeling, H&E staining showed sharply demarcated ulcerations, the rat colonic epithelium structure had disappeared, and the focal areas of extensive mucosal distortion were exfoliated. The crypts had disappeared and atrophied, and the submucosa was infiltrated by the mucosa (Figure 7(b)2). The colonic tissue in the model group rats also showed partial loss of colonic epithelium cells and the crypts exhibited structural changes, branches, distortion, and basal thickening. Plasma cells increased in the basal mucosal layer (Figure 7(c)2). In the H-FMP group, the colonic epithelium was restored, the crypts were arranged closely, and plentiful mucus and lymphocytes were maintained (Figure 7(d)2). In the L-FMP group, lymphocyte infiltration and crypt morphological alterations were observed in the colon tissue (Figure 7(e)2).

AB-PAS histopathologic staining indicated that AA enema caused a reduction in rat colon goblet cells and reduced mucus in the model group compared with the control group. After FMP treatment, the number of goblet cells in the H-FMP and L-FMP colons increased, and the large intestinal glands showed increased evidence of mucus (Figure 7(a)3–7(e)3). Masson histopathologic staining indicated that the UC rat submucosa had different degrees of incrassation. The submucosa and lamina propria layers had incrassated and uneven lower boundaries, which contained capillary fibroblasts. H-FMP and L-FMP decreased the number of capillary fibroblasts in the UC rats (Figure7(a)4–7(e)4).

### 3.7. Effects of FMPs on Inflammatory Cytokines

**Figure 8:**
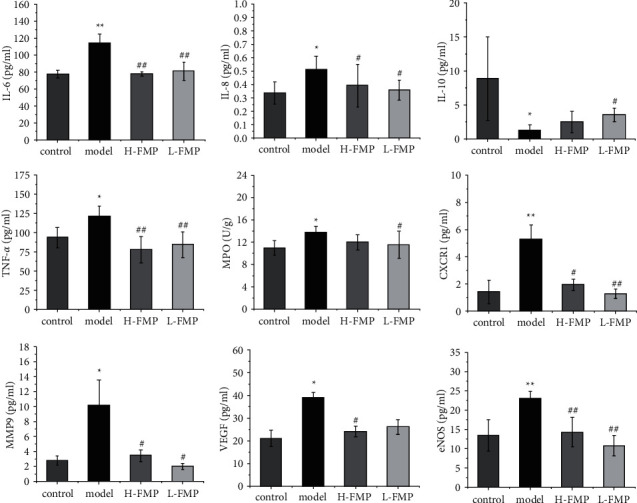
Effect of FMP on serum inflammation cytokines and predicted targets. ^*∗*^*p* < 0.05, ^*∗∗*^*p* < 0.001 compared with the control group and #*p* < 0.05, ##*p* < 0.001 compared with the model group.

FMP-UC network analysis predicted that inflammation could be a potential mechanism, and ongoing inflammation is part of the pathogenesis of UC. Therefore, inflammation-related targets and UC landmark inflammatory factors were verified, including the serum concentrations of IL-6, IL-10, TNF-*α*, IL-8, and colonic concentrations of MPO, CXCR1, and MMP9. As shown in Figure 8, compared with the control group, the administration of AA significantly increased rat serum concentrations of IL-6, IL-8, and TNF-*α* (*p* < 0.001,*p* < 0.05, *p* < 0.05); promoted MPO, CXCR1, and MMP9 expressions in the colon tissue (*p* < 0.05, *p* < 0.001, *p* < 0.05); and reduced the serum concentration of IL-10 in model rats (*p* < 0.05). Compared with the model group, the serum concentrations of IL-6, IL-8, TNF-*α*, and colonic concentrations of MPO, CXCR1, and MMP9 in the H-FMP group were significantly decreased (*p* < 0.001, *p* < 0.05, *p* < 0.001, *p* < 0.05, *p* < 0.05, *p* < 0.05). The serum concentration of IL-10 showed an increased tendency in the H-FMP group compared with the model group (*p* < 0.05). After low-dose FMP treatment, the serum concentrations of IL-6, IL-8, and TNF-*α* (*p* < 0.001, *p* < 0.05, *p* < 0.001) and colonic concentrations of MPO, CXCR1, and MMP9 (*p* < 0.05, *p* < 0.001, *p* < 0.05) were markedly reduced compared with those of the model group rats. In addition, the serum concentrations of IL-10 in L-FMP were evaluated (*p* < 0.05).

### 3.8. FMPs Prevent AA-Induced UC in Rats via Permeability and VEGF Pathways

Based on the above results, the potential pathways of FMPs against UC were significantly enriched for the permeability and VEGF signaling pathways, effects of FMPs on colonic VEGF, and eNOS level changes, and the VEGF pathway regulation is a potential mechanism. The colonic concentrations of VEGF and eNOS were significantly higher for the model group than for the control group (*p* < 0.05, *p* < 0.001). Compared with the model group, the high-dose FMP group showed markedly reduced colonic VEGF and eNOS expressions (*p* < 0.05, *p* < 0.001), and the low-dose FMP group showed significantly reduced colonic VEGF and eNOS expressions (*p* < 0.05, *p* < 0.001) (Figure 8).

**Figure 9:**
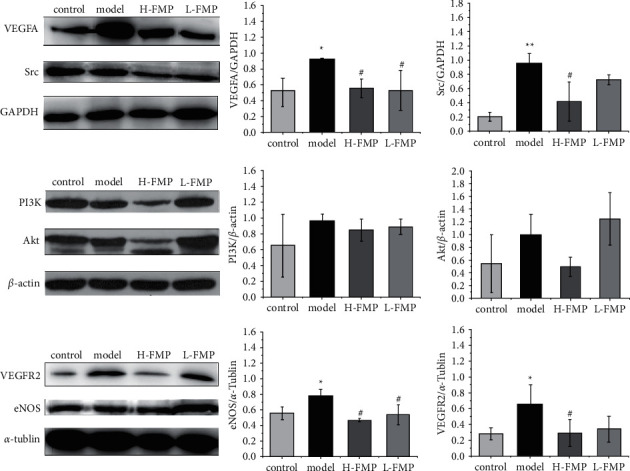
The effects of two FMP dosages on VEGFA, VEGFR2, Src, PI3k, Akt, and eNOS in colon tissue in AA-induced UC rats. Data are expressed as the mean ± SEM (*n* = 3). ^*∗*^*p* < 0.05, ^*∗∗*^*p* < 0.001 compared with the control group, and #*p* < 0.05 compared with the model group. The thresholds of detection were as follows: VEGFA, 25 kDa; VEGFR2, 200 kDa; Src, 55 kDa; PI3K, 80 kDa; Akt, 60 kDa; eNOS, 130 kDa; GAPDH, 37 kDa; *a*-tubulin, 55 kDa; and *ß*-actin, 42 kDa.

For the VEGF pathway changes, the colonic VEGFA, VAGFR2, Src, and eNOS concentrations were significantly overexpressed in acute colitis rats (*p* < 0.05, *p* < 0.05, *p* < 0.001, *p* < 0.05, respectively) and the PI3K concentrations showed an upward trend (*p* < 0.05), while the Akt level showed a decrease. High-dose FMP reduced colonic VEGFA, VEGFR2, eNOS, and Src protein expressions (*p* < 0.05, *p* < 0.05, *p* < 0.05, *p* < 0.05, respectively). Low-dose FMP decreased colonic VEGFA and eNOS protein expressions (*p* < 0.05, *p* < 0.05). These results indicate that FMPs may prevent colitis damage via the VEGF signaling pathway (Figure 9).

## 4. Discussion

Clinical studies have confirmed that FMPs are effective for treating UC in China; however, the protective mechanism of FMPs against UC requires further exploration. Therefore, we used a network pharmacology method to identify targets shared between UC and FMPs and explored the protective effect of FMPs. The 108 active compounds in FMPs were flavonoids, natural phytosterols, and isoquinoline alkaloids, including quercetin, luteolin, kaempferol, *ß*-sitosterol, and berberine. These compounds play roles in regulating cytokine concentrations and inflammation, restoring colon damage, and altering the gut. It has been shown that quercetin [29], luteolin [30], and berberine [31] suppress inflammatory damage in UC organoids; dietary kaempferol intake restores colon mucosal damage and inhibits MPO expression [32]; *ß*-sitosterol reduces weight loss, decreases proinflammatory factors, and resists pathogenic bacteria in UC mice [33].

For the UC pathogenesis, the activation of immune cells and persistent imbalance of inflammatory cytokines lead to chronic intestinal inflammation and repeated mucous stimulation. Inflammation-related biomarkers should be explored to identify new effective UC therapies. Our predicted FM-UC targets, such as MPO, CXCR1, MMP-9, and inflammatory cytokine biomarkers, namely TNF-*α*, IL-6, IL-8, and IL-10, are associated with inflammatory responses. TNF-*α* induces the apoptosis of epithelial cells and disrupts the epithelial barrier; the monoclonal antibodies of TNF-*α* golimumab and adalimumab have been successively used in clinical studies [34]. IL-6 is a pleiotropic cytokine that anticipates innate and adaptive immune responses and is evaluated in both the blood and colonic mucosa of patients and is positively associated with UC activity [35]. Serum MMP9 concentrations are markedly higher in active UC; specifically, MMP9 is positively correlated with serum IL-6 concentrations and platelet and leukocyte counts in UC [36]. Mutation or functional loss of IL-10 leads to UC severity and IL-10 KO induces spontaneous chronic colitis [37]. As a neutrophil-activating cytokine, IL-8, can be generated by endotheliocytes, suggesting a pathological grade in UC [38]. CXCR1, a receptor for IL-8, constitutes the primary mechanism of neutrophil recruitment, and its overexpression is observed in colon tissue during UC, which may increase the risk of colon cancer [39]. Activated neutrophils release MPO in an inflammatory environment, and MPO in colon tissue fragments can be used to evaluate inflammation in UC [40]. This study indicated that anti-inflammation is one of the mechanisms by which FMPs alleviate AA-induced colitis in rats by regulating the above cytokine markers by reducing serum TNF-*α*, IL-6, and IL-8 concentrations and colonic MPO, MMP-9, and CXCR1 concentrations. In addition, histological observations suggest that FMPs aid goblet cell recovery and inflammatory infiltration mitigation.

Permeability changes are strongly associated with UC pathology; crucial endothelial damage, microvascular permeability (mVP), perivascular edema, and epithelial hypoxia precede epithelial barrier dysfunction progressing to erosions, ulceration, and inflammation [41]. mVP has proven to be a crucial factor; anatomical observation and functional vascular changes have been observed during ulcer healing and progression from UC toward colorectal carcinoma [42, 43]. VEGF and its receptors have emerged as principal drivers that mediate mVP and endothelial permeability, which are considered major factors that predispose patients to UC [44]. Evidence shows that VEGF increases in the serum and tissue during the onset of UC, activates mVP, and has detrimental effects on the colon barrier [45]. VEGF has a dual role in colon tissue; overexpression mediates the recruitment of inflammatory cells and enhances the expression of costimulatory molecules; likewise, it seems to have immunosuppressive effects on tumor growth in colorectal carcinoma [46]. NO synthase is best known for its role in endothelium-mediated relaxation of mVP. Definitive evidence determines the activity of eNOS, which increases mVP to macromolecules in response to inflammatory agents. eNOS-derived NO causes hyperpermeability in response to VEGF for multiple diseases [47]. Studies have shown that serum eNOS is markedly induced in UC [48] and involved in nitrosative stress and UC-associated carcinogenesis [49], and the loss of eNOS is protective in a dextran sodium sulfate model of colitis [50]. Our predicted targets and enriched KEGG signaling pathways all indicated that FMPs may have protective effects on UC by influencing mVP, especially by regulating the VEGF, NO synthase, and VEGF signaling pathways. Our results reinforced the idea that colonic VEGF and eNOS levels were higher than normal in AA-induced colitis; 900 mg/kg and 450 mg/kg of FMPs could inhibit the increase in VEGFA and eNOS concentrations, respectively. Thus, our predicted targets, VEGF and NOS, were targets of FMP treatment, and FMPs could alleviate colitis in the model by suppressing VEGF and eNOS.

The VEGF pathway directly regulates the mVP. VEGFR-2 is the major mediator of VEGF-driven responses in endothelial cells and is considered a crucial signal transducer for both physiological and pathological permeability [51]. Binding leads to the activation of Src, whereas activation of the PI3K-Akt pathway leads to increased eNOS expression, stimulating mVP augmentation [52]. Our results provide evidence that the VEGF signaling pathway was activated in the AA-induced colitis model; VEGFA, VEGFR2, Src, and eNOS protein expression levels were increased through a cascade reaction; and PI3K was overexpressed. This is the first study to show that FMPs can inhibit the activation of the VEGF-PI3k/Akt-eNOS signaling pathway and downregulate VEGFA, VEGFR2, Src, and eNOS colonic protein concentrations. FMPs showed the potential of reducing the expression of PI3K, and the influence on Akt requires further study. Changes in PI3K/ AKT respond to various upstream protein regulations, which have different regulatory trends, ultimately leading to an increase in Akt concentrations in this study. In addition, 900 mg/kg of FMPs had a better effect on the regulation of the VEGF-PI3K/Akt-eNOS signaling pathway than 450 mg/kg of FMPs.

This study showed evidence of the effects of FMPs in an acute UC model. Further studies are required to confirm that FMP administration can reverse chronic colitis fibrosis or colorectal cancer induced by chronic UC. A chronic UC disease model may be needed to observe this mechanism. The safety of long-term FMP treatment will be the focus of our future study. This study may support permeability alterations that require further exploration for their roles in gastrointestinal diseases.

## 5. Conclusion

The FMP-UC network pharmacology suggested that FMP treats UC by regulating inflammation, oxidative stress, permeability, and endothelial recovery. FMP relieved typical symptoms of UC, repaired colon tissue damage via anti-inflammatory responses, and regulated VEGF-PI3K/AKT-eNOS signaling pathway-mediated microvascular permeability. Based on the efficacy of FMP, this may represent a useful alternative strategy for treating UC.

## Data Availability

Please contact the first or corresponding author for data support. Network pharmacology data will be uploaded as supplements 2, 3, and 4.

## Abbreviations

AA: Acetic acid
BC: Betweenness centrality
BCA: Bicinchonic acid
BP: Biological process
CC: Cellular component
CXCR1: C-X-C chemokine receptor type 1
DAI: Disease active index
DC:Degree centralityDG: Degree centralityDG:Radix Angelicae Sinensis
DL: Drug-likeness
DS: Radix Codcnopsitis Pilosulas
eNOS: Endothelial nitric oxide synthase
FMP: Fructus Mume Pills
FZ: Radix Aconiti Praeparata;
GJ: Rhizoma Zingiberis
GO: Gene ontology
GZ: Ramulus Cinnamomi;
HB: Cortex Phellodendri
HL: Phizoma Coptidis
H-FMP: High-dose FMP
L-6: Interleukin-6
IL-8: Interleukin-8
IL-10: Interleukin-10
KEGG: Kyoto Encyclopedia of Genes and Genomes
L-FMP: Low-dose FMP
OB:Bio availabilityOD:Optical DensityMF: Bio availabilityOD:Optical DensityMF:Molecular function
MMP9: Matrix metalloproteinase-9
MPO: Myeloperoxidase
mVP: Microascular permeability
PPI: Protein-protein interaction
*p*-value: Probability value
PVDF: Polyvinylidene fluoride
SJ: Pericarpium Zanthoxyli
TNF-*α*: Tumor necrosis factor-*α*
UC: Ulcerative Colitis
UPLC: Ultra performance liquid chromatograph
VEGF: Vascular endothelial growth factor
VEGFR2: VEGF receptor 2
WM:Fructus MumeXX: Fructus MumeXX:Herba Asar.

## Supplementary Materials

Supplementary Materials Supplement 1 FMP Quality Control Methods and Results. Supplement 2 FMP Active Compounds and Targets Supplement 3 Ulcerative Colitis Targets Supplement 4 GO Enrichment Result Supplement 5 KEGG Enrichment Results.
