# Supplementary material for: Mechanism of Fructus Mume Pills Underlying Their Protective Effects in Rats with Acetic Acid-Inducedulcerative Colitis via the Regulation of Inflammatory Cytokines and the VEGF-PI3K/Akt-eNOS Signaling Pathway
Source: Evid Based Complement Alternat Med. 2022 May 2;2022:4621131. doi: 10.1155/2022/4621131 (PMC9129976; doi:10.1155/2022/4621131)
Supplement: Supplementary Materials — Supplement 1 FMP Quality Control Methods and Results. Supplement 2 FMP Active Compounds and Targets Supplement 3 Ulcerative Colitis Targets Supplement 4 GO Enrichment Result Supplement 5 KEGG Enrichment Results. [file 4621131.f1.zip › 4621131.f1/Supplement 1 FMP Quality Control Methods and Results.docx]

**FMP quality control materials and reagents**

FMP was obtained from Sichuan Neautus Traditional Chinese Medicine Co., Ltd. (Sichuan China). The FMP quality control standard reference substances citric acid monohydrate (100396-201603, 100%), berberine hydrochloride (110713-201804, 86.7%), ferulic acid (110773-201915, 99.4%), cinnamic acid (110786-201604, 98.8%), phellodendron hydrochloride (111895-201303, 100%), α-asarum ether (100298-201203, 100%), β-asarum ether (112018-201601, 96.8%), and 6-gingerol (111833-201806, 99.9%) were purchased from the National Institution of Food and Drug Control. Coptisine hydrochloride (MUST-1905211, 99.58%) was purchased from Chengdu Must Bio-technology Co., Ltd., hydroxy-α-sanshool (RP200528, 98.34%) was obtained from Chengdu Refmedic Biotechnology Co., Ltd., lobetyolin (PU0058-0010, 99%) was purchased from Chengdu Push Bio-technology Co., Ltd., benzoylaconine (DST190619-055), benzoyl neoaconitine (DST1911029-056), and benzoyl hypoaconitine (DST191103-057) were obtained from Chengdu Desite Biotechnology Co., Ltd. Other chemicals were chromatographically pure and were purchased from commercial sources.

**FMP quality control method and results**

The quality of FMP was analysed by a Waters e2695 Ultra performance liquid chromatograph (UPLC), Waters 2489 ultraviolet detector, and Waters 2998 PAD detector (Waters, U.S.A.). Citric acid, berberine hydrochloride, ferulic acid, cinnamic acid, phellodendron hydrochloride, α-asarum ether, β-asarum ether, 6-gingerol, coptisine hydrochloride, hydroxy-α-sanshool, lobetyolin, benzoylaconine, benzoyl neoaconitine, and benzoyl hypoaconitine were selected as characteristic quality control markers for the ten herbs according to Chinese pharmacopoeia 2020, and the content of citric acid monohydrate should not be less than 6%. The wavelength and retention time of the corresponding characteristic substance in aqueous FMP should be correlated to those of the standard substances. The first eleven standard substances were dissolved in absolute methanol. FMP detection sample solution was prepared by mixing 0.5 g of FMP powder in 50 ml of ultrapure water, heating reflux in a 100ºC water bath for 60 min, and then replenishing the lost mass. Ten microlitres of FMP extraction solution was filtered with a 0.22-μm Millipore filter and injected into a Waters SymmetryShield C18 (4.6×250 mm, 5 µm). Linear elution was performed with acetonitrile (A) and 0.1% phosphoric acid (B). The linear gradient elution was as follows: 0~10 min, acetonitrile 1%, 0.1% phosphoric acid 99%; 10~20 min, acetonitrile 2%→12%, 0.1% phosphoric acid 98%→88%; 20~30 min, acetonitrile 12%→20%, 0.1% phosphoric acid 88%→80%; 30~40 min, acetonitrile 20%→30%, 0.1% phosphoric acid 80%→70%; 40~50 min, acetonitrile 30%→40%, 0.1% phosphoric acid 70%→60%; 50~60 min, acetonitrile 40%→70%, 0.1% phosphoric acid 60%→30%; 60~70 min, acetonitrile 70%→20%, 0.1% phosphoric acid 30%→80%; 70~75 min, acetonitrile 20%→1%, 0.1% phosphoric acid→99%→75 min, 80%→80%. The flow velocity was set to 0.8 ml/min, the wavelength was 285 nm, and the column temperature was 27ºC.

# To investigate whether this detection condition was reliable, we investigated the stability, precision, and repetition and ensured the quality of the FMP powder. The UPLC results showed that the retention times of citric acid, phellodendron hydrochloride, coptisine hydrochloride, berberine hydrochloride, ferulic acid, lobetyolin, cinnamic acid, hydroxy-α-sanshool, 6-gingerol, β-asarum ether, and α-asarum ether in FMP were 6.3 min, 14.09 min, 30.53 min, 44.97 min, 48.12 min, 53.75 min, 62.48 min, 68.08 min, 68.38 min, 69.90 min, and 71.68 min. The retention times were consistent with those of the standard reference substances, and the absorption waves were consistent with those of the standard reference substances (Fig. 1A, B) The content of citric acid was approximately 56.47 mg/g, and the RSD was 2.97%.

# Benzoylaconine, benzoyl neoaconitine, and benzoyl hypoaconitine standard substances were dissolved in dichloromethane and isopropanol (1:1). FMP samples for benzoylaconine, benzoyl neoaconitine, benzoyl hypoaconitine detection were ultrasonically extracted in isopropanol and ethyl acetate (1:1) for 30 min. Ten microlitres of FMP extracting solution was filtered with a 0.22-μm Millipore filter and injected into a Waters SymmetryShield C18 (4.6×250 mm, 5 μm). The mobile phase was acetonitrile: tetrahydrofuran (25:15, A) and 0.1 mol/L ammonium acetate (B), and the linear gradient elution was performed as 0~48 min, pump A 15%→26%, pump B 85%→74%; 48~49 min, pump A 26%→35%, pump B 74%→65%; 49~58 min, pump A 35%, pump B 65%; 58~65 min, pump A 35%→15%, pump B 865%→85%. The wavelength for determination was 235 nm, the flow velocity was 0.8 ml/min, and the column temperature was 25℃. The retention times of benzoyl neoaconitine, benzoyl hypoaconitine, and benzoylaconine in FMP were 17.26 min, 22.71 min, and 47.05 min, respectively, at a wavelength of 235 nm (Fig. 1C, D).

**
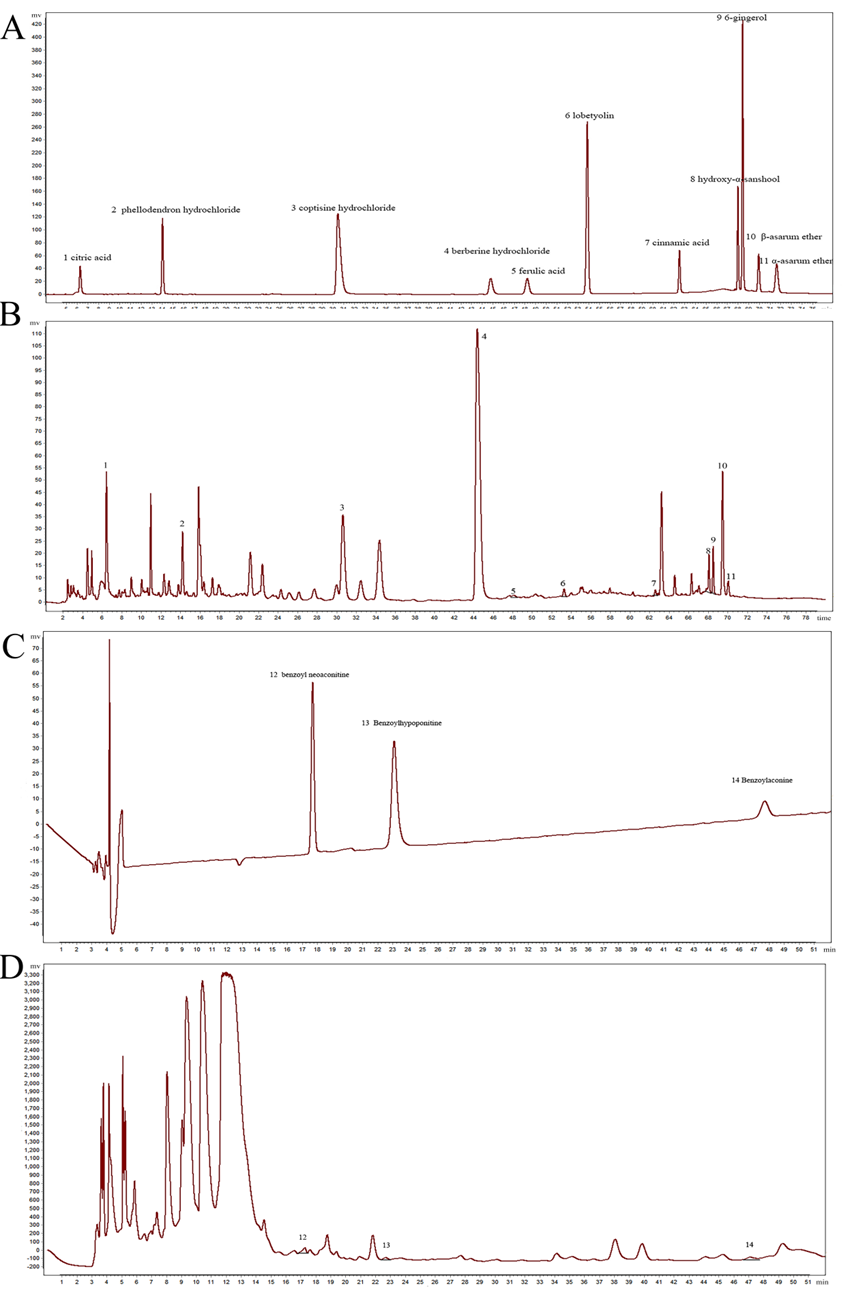
**

Fig.1 UPLC profiles of standards and FMP aqueous extract (A and B were detected at 285 nm, and C and D were detected at 235 nm).
