# Supplementary material for: Mechanism of Fructus Mume Pills Underlying Their Protective Effects in Rats with Acetic Acid-Inducedulcerative Colitis via the Regulation of Inflammatory Cytokines and the VEGF-PI3K/Akt-eNOS Signaling Pathway
Source: Evid Based Complement Alternat Med. 2022 May 2;2022:4621131. doi: 10.1155/2022/4621131 (PMC9129976; doi:10.1155/2022/4621131)
Supplement: Supplementary Materials — Supplement 1 FMP Quality Control Methods and Results. Supplement 2 FMP Active Compounds and Targets Supplement 3 Ulcerative Colitis Targets Supplement 4 GO Enrichment Result Supplement 5 KEGG Enrichment Results. [file 4621131.f1.zip › 4621131.f1/Supplement 2 FMP Active Compounds and Targets.pdf]

Table 1 summary list of FMP compounds

Durg name	Symbol	Compound
WM	MOL001040	(2R)-5,7-dihydroxy-2-(4-hydroxyphenyl)chroman-4-one
	MOL000358	beta-sitosterol
	MOL000422	kaempferol
	MOL000449	Stigmasterol
	MOL005043	campest-5-en-3beta-ol
	MOL008601	Methyl arachidonate
	MOL000953	CLR
	MOL000098	quercetin
HL	MOL001454	berberine
	MOL013352	Obacunone
	MOL002894	berberrubine
	MOL002897	epiberberine
	MOL002903	(R)-Canadine
	MOL002904	Berlambine
	MOL002907	Corchoroside A_qt
	MOL000622	Magnograndiolide
	MOL000762	Palmidin A
	MOL000785	palmatine
	MOL000098	quercetin
	MOL001458	coptisine
	MOL002668	Worenine
	MOL008647	Moupinamide
DS	MOL001006	poriferasta-7,22E-dien-3beta-ol
	MOL002140	Perlolyrine
	MOL002879	Diop
	MOL003036	ZINC03978781
	MOL000449	Stigmasterol
	MOL003896	7-Methoxy-2-methyl isoflavone
	MOL004355	Spinasterol
	MOL004492	Chrysanthemaxanthin
	MOL005321	Frutinone A
	MOL000006	luteolin
	MOL006554	Taraxerol
	MOL006774	stigmast-7-enol
	MOL007059	3-beta-Hydroxymethyllenetanshiquinone
	MOL007514	methyl icosa-11,14-dienoate
	MOL008391	5alpha-Stigmastan-3,6-dione
	MOL008393	7-(beta-Xylosyl)cephalomannine_qt
	MOL008397	Daturilin
	MOL008400	glycitein
	MOL008406	Spinoside A
	MOL008407	R,13R,14S,17R)-17-[(E,2R,5S)-5-ethyl-6-methylhept-3-en-2-yl]-10,13-dimethyl-1,2,4,7,8,9,11,12,14,15,16,17-dodecahydrocyclopenta[a]phenantl
	MOL008411	11-Hydroxyrankinidine
DG	MOL000358	beta-sitosterol
	MOL000449	Stigmasterol
GJ	MOL002464	1-Monolinolein
	MOL002501	3-[(E)-but-2-enyl]-2-methyl-4-oxo-1-cyclopent-2-enyl] (1R,3R)-3-[(E)-3-methoxy-2-methyl-3-oxoprop-1-enyl]-2,2-dimethylcyclopropane-1-carbo
	MOL002514	Sexangularetin
	MOL000358	beta-sitosterol
	MOL000359	sitosterol
GZ	MOL001736	(-)-taxifolin
	MOL000358	beta-sitosterol
	MOL000359	sitosterol
	MOL000492	(+)-catechin
	MOL000073	ent-Epicatechin
	MOL004576	taxifolin
	MOL011169	Peroxyergosterol
SJ	MOL013271	Kokusaginin
	MOL002663	Skimmianin
	MOL002881	Diosmetin
	MOL000358	beta-sitosterol
	MOL000098	quercetin
XX	MOL012140	4,9-dimethoxy-1-vinyl-$b-carboline
	MOL012141	Caribine
	MOL001460	Cryptopin
	MOL001558	sesamin
	MOL002501	3-[(E)-but-2-enyl]-2-methyl-4-oxo-1-cyclopent-2-enyl] (1R,3R)-3-[(E)-3-methoxy-2-methyl-3-oxoprop-1-enyl]-2,2-dimethylcyclopropane-1-carbo
	MOL002962	(3S)-7-hydroxy-3-(2,3,4-trimethoxyphenyl)chroman-4-one
	MOL000422	kaempferol
	MOL009849	ZINC05223929
FZ	MOL002211	11,14-eicosadienoic acid
	MOL002388	Delphin_qt
	MOL002392	Deltoin
	MOL002393	Demethyldelavaine A
	MOL002394	Demethyldelavaine B
	MOL002395	Deoxyandrographolide
	MOL002397	karakoline
	MOL002398	Karanjin
	MOL002401	Neokadsuranic acid B
	MOL002406	2,7-Dideacetyl-2,7-dibenzoyl-taxayunnanine F
	MOL002410	benzoylnapelline
	MOL002415	6-Demethyldesoline
	MOL002416	deoxyaconitine
	MOL002419	(R)-Norcoclaurine
	MOL002421	ignavine
	MOL002422	isotalatizidine
	MOL002423	jesaconitine
	MOL002433	:hyl-17-[(E,2R)-6-methyl-7-[(2R,3R,4S,5S,6R)-3,4,5-trihydroxy-6-[[(2R,3R,4S,5S,6R)-3,4,5-trihydroxy-6-(hydroxymethyl)oxan-2-yl]oxymethyl]c
	MOL002434	Carnosifloside I_qt
	MOL000359	sitosterol
	MOL000538	hypaconitine
HB	MOL001454	berberine
	MOL001458	coptisine
	MOL002636	Kihadalactone A
	MOL013352	Obacunone
	MOL002641	Phellavin_qt
	MOL002643	delta 7-stigmastenol
	MOL002644	Phellopterin
	MOL002651	Dehydrotanshinone II A
	MOL002652	delta7-Dehydrosophoramine
	MOL002656	dihydroniloticin
	MOL002659	kihadanin A
	MOL002660	niloticin
	MOL002662	rutaecarpine
	MOL002663	Skimmianin
	MOL002666	Chelerythrine

MOL000449	Stigmasterol
MOL002668	Worenine
MOL002670	Cavidine
MOL002671	Candletoxin A
MOL002672	Hericenone H
MOL002673	Hispidone
MOL000358	beta-sitosterol
MOL000622	Magnograndiolide
MOL000762	Palmidin A
MOL000785	palmatine
MOL000787	Fumarine
MOL000790	Isocorypalmine
MOL000098	quercetin
MOL001131	phellamurin qt
MOL001455	(S)-Canadine
MOL001771	poriferast-5-en-3beta-ol
MOL002894	berberrubine
MOL005438	campesterol
MOL006401	melianone
MOL006413	phellochin
MOL006422	thalifendine

Table 2 FMP targets of active compounds

Compounds	Targets
MOL011169	NOS2
MOL009849	MCL1
MOL008407	CTSD
MOL008400	EGFR
MOL006774	AR
MOL005438	AR
MOL005438	NR1H3
MOL005438	HMGCR
MOL005438	CYP51A1
MOL005438	NPC1L1
MOL005438	CYP17A1
MOL005043	AR
MOL005043	NR1H3
MOL005043	HMGCR
MOL005043	CYP51A1
MOL005043	NPC1L1
MOL005043	CYP17A1
MOL004355	AR
MOL003036	AR
MOL003036	NPC1L1
MOL003036	HMGCR
MOL003036	CYP51A1
MOL003036	CYP19A1
MOL002903	DRD1
MOL002903	DRD2
MOL002903	F3
MOL002903	HTR7
MOL002897	UBA2
MOL002897	ACHE
MOL002897	SAE1
MOL002881	ABCC1
MOL002881	CYP1B1
MOL002670	F3
MOL002670	DRD1
MOL002666	ACHE
MOL002666	PRKCE
MOL002662	PTGS1
MOL002662	PTGS2
MOL002643	AR
MOL002514	AKR1B1
MOL002419	DRD2
MOL002419	DRD4
MOL002211	PPARG
MOL002211	PPARA
MOL002211	PPARD
MOL002211	FABP4
MOL002211	FABP3
MOL001771	AR
MOL001771	HMGCR
MOL001771	CYP51A1
MOL001771	NPC1L1
MOL001771	NR1H3
MOL001771	CYP19A1
MOL001558	MCL1
MOL001455	DRD1
MOL001455	DRD2
MOL001455	F3
MOL001455	HTR7
MOL001454	HTR2B
MOL001454	BCHE
MOL001454	ADRA2C
MOL001454	ADRA2B
MOL001454	CHRM1
MOL001454	ACHE
MOL001454	SIGMAR1
MOL001454	CYP2D6
MOL001040	CYP19A1
MOL001040	CA7
MOL001040	ABCC1
MOL001040	HSD17B1
MOL001040	CA12
MOL001040	SHBG
MOL001040	CA4
MOL001040	CYP1B1
MOL001040	CBR1
MOL001040	ESR1
MOL001040	ESR2
MOL000953	AR
MOL000953	NPC1L1
MOL000953	CYP17A1
MOL000953	NR1H3
MOL000953	HMGCR
MOL000953	CYP51A1
MOL000790	DRD1

MOL000790	DRD2
MOL000790	DRD3
MOL000790	SIGMAR1
MOL000790	HTR1A
MOL000790	F3
MOL000790	DRD4
MOL000785	ACHE
MOL000449	AR
MOL000449	NPC1L1
MOL000449	HMGCR
MOL000449	CYP51A1
MOL000449	CYP19A1
MOL000449	RORC
MOL000422	NOX4
MOL000422	AKR1B1
MOL000422	XDH
MOL000422	TYR
MOL000422	FLT3
MOL000422	CA2
MOL000422	ALOX5
MOL000422	CA7
MOL000422	HSD17B2
MOL000422	ABCC1
MOL000422	HSD17B1
MOL000422	AHR
MOL000422	CA12
MOL000422	ESRRA
MOL000422	ABCB1
MOL000422	CYP1B1
MOL000422	ABCG2
MOL000422	ADORA1
MOL000422	CA4
MOL000422	ACHE
MOL000422	MAOA
MOL000422	GLO1
MOL000422	SYK
MOL000422	GSK3B
MOL000422	MMP9
MOL000422	MMP2
MOL000422	ALOX15
MOL000422	ALOX12
MOL000422	PTPRS
MOL000422	ADORA2A
MOL000422	CDK5R1
MOL000422	CCNB3
MOL000422	CDK5
MOL000422	CDK1
MOL000422	CCNB1
MOL000422	CCNB2
MOL000422	ARG1
MOL000422	GPR35
MOL000422	ESR2
MOL000422	DAPK1
MOL000422	MPG
MOL000422	SLC22A12
MOL000359	AR
MOL000359	HMGCR
MOL000359	CYP51A1
MOL000359	NPC1L1
MOL000359	NR1H3
MOL000358	AR
MOL000358	HMGCR
MOL000358	CYP51A1
MOL000358	NPC1L1
MOL000358	NR1H3
MOL000358	CYP19A1
MOL000098	NOX4
MOL000098	AVPR2
MOL000098	AKR1B1
MOL000098	XDH
MOL000098	MAOA
MOL000098	IGF1R
MOL000098	FLT3
MOL000098	CYP19A1
MOL000098	EGFR
MOL000098	F2
MOL000098	CA2
MOL000098	PIM1
MOL000098	ALOX5
MOL000098	AURKB
MOL000098	DRD4
MOL000098	ADORA1
MOL000098	CA7
MOL000098	GLO1
MOL000098	MPO
MOL000098	PIK3R1
MOL000098	ADORA2A
MOL000098	DAPK1
MOL000098	PYGL
MOL000098	CA1
MOL000098	GSK3B
MOL000098	SRC
MOL000098	PTK2
MOL000098	HSD17B2
MOL000098	KDR
MOL000098	MMP13
MOL000098	MMP3
MOL000098	CA3
MOL000098	ALOX15
MOL000098	ABCC1
MOL000098	PLK1
MOL000098	CA6
MOL000098	MMP9
MOL000098	CA12
MOL000098	MMP2
MOL000098	PKN1
MOL000098	CA14
MOL000098	CA9
MOL000098	CSNK2A1

MOL000098	ALOX12
MOL000098	MET
MOL000098	CA4
MOL000098	NEK2
MOL000098	CXCR1
MOL000098	CAMK2B
MOL000098	ALK
MOL000098	AKT1
MOL000098	ABCB1
MOL000098	NEK6
MOL000098	PLA2G1B
MOL000098	CA5A
MOL000098	BACE1
MOL000098	CYP1B1
MOL000098	AXL
MOL000098	ABCG2
MOL000098	NUAK1
MOL000098	AKR1C2
MOL000098	AKR1C1
MOL000098	AKR1C3
MOL000098	AKR1C4
MOL000098	CA13
MOL000098	AKR1A1
MOL000098	GPR35
MOL000098	SYK
MOL000098	MAPT
MOL000098	KDM4E
MOL000098	TOP2A
MOL000098	INSR
MOL000098	ACHE
MOL000098	MYLK
MOL000098	PIK3CG
MOL000098	APEX1
MOL000098	CDK5R1
MOL000098	CDK5
MOL000098	CDK1
MOL000098	CCNB1
MOL000098	CCNB2
MOL000098	CCNB3
MOL000098	ARG1
MOL000098	PTPRS
MOL000098	ESR2
MOL000098	MPG
MOL000098	SLC22A12
MOL000098	CDK6
MOL000098	CDK2
MOL000006	NOX4
MOL000006	AKR1B1
MOL000006	CDK5R1
MOL000006	XDH
MOL000006	MAOA
MOL000006	FLT3
MOL000006	CA2
MOL000006	CDK5
MOL000006	CDK1
MOL000006	CCNB1
MOL000006	CCNB2
MOL000006	CCNB3
MOL000006	ALOX5
MOL000006	ADORA1
MOL000006	CA7
MOL000006	GLO1
MOL000006	APP
MOL000006	SYK
MOL000006	GSK3B
MOL000006	PARP1
MOL000006	TTR
MOL000006	MMP9
MOL000006	CA12
MOL000006	MMP2
MOL000006	CA4
MOL000006	MMP12
MOL000006	CD38
MOL000006	CYP1B1
MOL000006	ABCG2
MOL000006	AKR1B10
MOL000006	TNKS2
MOL000006	TNKS
MOL000006	TOP1
MOL000006	ARG1
MOL000006	PTPRS
MOL000006	ABCC1
MOL000006	HSD17B1
MOL000006	ACHE
MOL000006	CDK6
MOL000006	ABCB1
