# Supplementary material for: Mechanism of Fructus Mume Pills Underlying Their Protective Effects in Rats with Acetic Acid-Inducedulcerative Colitis via the Regulation of Inflammatory Cytokines and the VEGF-PI3K/Akt-eNOS Signaling Pathway
Source: Evid Based Complement Alternat Med. 2022 May 2;2022:4621131. doi: 10.1155/2022/4621131 (PMC9129976; doi:10.1155/2022/4621131)
Supplement: Supplementary Materials — Supplement 1 FMP Quality Control Methods and Results. Supplement 2 FMP Active Compounds and Targets Supplement 3 Ulcerative Colitis Targets Supplement 4 GO Enrichment Result Supplement 5 KEGG Enrichment Results. [file 4621131.f1.zip › 4621131.f1/Supplement 3 Ulcerative Colitis Targets.pdf]

Table 1 summary of UC targets

Gene ID	Species	Target name
POP5	Homo sapiens	POP5 homolog, ribonuclease P/MRP subunit(POP5)
POP7	Homo sapiens	POP7 homolog, ribonuclease P/MRP subunit(POP7)
TEX41	Homo sapiens	testis expressed 41 (non-protein coding)(TEX41)
CLDN2	Homo sapiens	claudin 2(CLDN2)
F11R	Homo sapiens	F11 receptor(F11R)
CLDN1	Homo sapiens	claudin 1(CLDN1)
RABGEF1	Homo sapiens	RAB guanine nucleotide exchange factor 1(RABGEF1)
KIAA1109	Homo sapiens	KIAA1109(KIAA1109)
HTR7	Homo sapiens	5-hydroxytryptamine receptor 7(HTR7)
LURAP1L	Homo sapiens	leucine rich adaptor protein 1 like(LURAP1L)
UBASH3A	Homo sapiens	ubiquitin associated and SH3 domain containing A(UBASH3A)
GSTK1	Homo sapiens	glutathione S-transferase kappa 1(GSTK1)
FNBP1	Homo sapiens	formin binding protein 1(FNBP1)
FLJ31356	Homo sapiens	uncharacterized protein FLJ31356(FLJ31356)
SLC5A8	Homo sapiens	solute carrier family 5 member 8(SLC5A8)
CLDN4	Homo sapiens	claudin 4(CLDN4)
DDX39A	Homo sapiens	DExD-box helicase 39A(DDX39A)
MIR196A2	Homo sapiens	microRNA 196a-2(MIR196A2)
CLDN7	Homo sapiens	claudin 7(CLDN7)
TXNIP	Homo sapiens	thioredoxin interacting protein(TXNIP)
TBPL1	Homo sapiens	TATA-box binding protein like 1(TBPL1)
PADI4	Homo sapiens	peptidyl arginine deiminase 4(PADI4)
SKAP2	Homo sapiens	src kinase associated phosphoprotein 2(SKAP2)
IDO1	Homo sapiens	indoleamine 2,3-dioxygenase 1(IDO1)
ABCB1	Homo sapiens	ATP binding cassette subfamily B member 1(ABCB1)
ABCB6	Homo sapiens	[illegible] binding cassette subfamily B member 6 (Langereis blood group)(ABC
MIR196B	Homo sapiens	microRNA 196b(MIR196B)
FPR2	Homo sapiens	formyl peptide receptor 2(FPR2)
PLA2G6	Homo sapiens	phospholipase A2 group VI(PLA2G6)
PLA2G7	Homo sapiens	phospholipase A2 group VII(PLA2G7)
HSD11B2	Homo sapiens	hydroxysteroid 11-beta dehydrogenase 2(HSD11B2)
CYP2B6	Homo sapiens	cytochrome P450 family 2 subfamily B member 6(CYP2B6)
IGF2-AS	Homo sapiens	IGF2 antisense RNA(IGF2-AS)
COX2	Homo sapiens	cytochrome c oxidase subunit II(COX2)
COX1	Homo sapiens	cytochrome c oxidase subunit I(COX1)
AMZ1	Homo sapiens	archaelysin family metallopeptidase 1(AMZ1)
LYN	Homo sapiens	LYN proto-oncogene, Src family tyrosine kinase(LYN)
GSTM1	Homo sapiens	glutathione S-transferase mu 1(GSTM1)
GPR12	Homo sapiens	G protein-coupled receptor 12(GPR12)
TSLP	Homo sapiens	thymic stromal lymphopoietin(TSLP)
NR0B1	Homo sapiens	nuclear receptor subfamily 0 group B member 1(NR0B1)
LYZ	Homo sapiens	lysozyme(LYZ)
NR0B2	Homo sapiens	nuclear receptor subfamily 0 group B member 2(NR0B2)
PGA5	Homo sapiens	pepsinogen 5, group I (pepsinogen A)(PGA5)
FERMT1	Homo sapiens	fermitin family member 1(FERMT1)
FASN	Homo sapiens	fatty acid synthase(FASN)
PSMG1	Homo sapiens	proteasome assembly chaperone 1(PSMG1)
PKIG	Homo sapiens	protein kinase (cAMP-dependent, catalytic) inhibitor gamma(PKIG)
HLA-DRB3	Homo sapiens	major histocompatibility complex, class II, DR beta 3(HLA-DRB3)
NOX1	Homo sapiens	NADPH oxidase 1(NOX1)
GPR18	Homo sapiens	G protein-coupled receptor 18(GPR18)
HLA-DRB1	Homo sapiens	major histocompatibility complex, class II, DR beta 1(HLA-DRB1)
LITAF	Homo sapiens	lipopolysaccharide induced TNF factor(LITAF)
PANX1	Homo sapiens	pannexin 1(PANX1)
PANX2	Homo sapiens	pannexin 2(PANX2)
RPLP2	Homo sapiens	ribosomal protein lateral stalk subunit P2(RPLP2)
CTLA4	Homo sapiens	cytotoxic T-lymphocyte associated protein 4(CTLA4)
JAK2	Homo sapiens	Janus kinase 2(JAK2)
RIPK2	Homo sapiens	receptor interacting serine/threonine kinase 2(RIPK2)
GPR35	Homo sapiens	G protein-coupled receptor 35(GPR35)
DUSP1	Homo sapiens	dual specificity phosphatase 1(DUSP1)
SPHK1	Homo sapiens	sphingosine kinase 1(SPHK1)

FOS	Homo sapiens	Fos proto-oncogene, AP-1 transcription factor subunit(FOS)
F2	Homo sapiens	coagulation factor II, thrombin(F2)
F5	Homo sapiens	coagulation factor V(F5)
PPARG	Homo sapiens	peroxisome proliferator activated receptor gamma(PPARG)
PPARA	Homo sapiens	peroxisome proliferator activated receptor alpha(PPARA)
CHST2	Homo sapiens	carbohydrate sulfotransferase 2(CHST2)
CRB1	Homo sapiens	crumbs 1, cell polarity complex component(CRB1)
MYRF	Homo sapiens	myelin regulatory factor(MYRF)
IDUA	Homo sapiens	iduronidase, alpha-L-(IDUA)
GSTP1	Homo sapiens	glutathione S-transferase pi 1(GSTP1)
ODC1	Homo sapiens	ornithine decarboxylase 1(ODC1)
HPP1	Homo sapiens	hyperpigmentation, progressive, 1(HPP1)
KCNA3	Homo sapiens	potassium voltage-gated channel subfamily A member 3(KCNA3)
CCNDBP1	Homo sapiens	cyclin D1 binding protein 1(CCNDBP1)
PTGS2	Homo sapiens	prostaglandin-endoperoxide synthase 2(PTGS2)
EGFR	Homo sapiens	epidermal growth factor receptor(EGFR)
PTGS1	Homo sapiens	prostaglandin-endoperoxide synthase 1(PTGS1)
NTF3	Homo sapiens	neurotrophin 3(NTF3)
SPP1	Homo sapiens	secreted phosphoprotein 1(SPP1)
STAT4	Homo sapiens	signal transducer and activator of transcription 4(STAT4)
FLNB	Homo sapiens	filamin B(FLNB)
STAT6	Homo sapiens	signal transducer and activator of transcription 6(STAT6)
STX2	Homo sapiens	syntaxin 2(STX2)
LINC00994	Homo sapiens	long intergenic non-protein coding RNA 994(LINC00994)
P2RY13	Homo sapiens	purinergic receptor P2Y13(P2RY13)
EGLN3	Homo sapiens	egl-9 family hypoxia inducible factor 3(EGLN3)
SLC16A1	Homo sapiens	solute carrier family 16 member 1(SLC16A1)
STAT1	Homo sapiens	signal transducer and activator of transcription 1(STAT1)
P2RY14	Homo sapiens	purinergic receptor P2Y14(P2RY14)
STAT3	Homo sapiens	signal transducer and activator of transcription 3(STAT3)
GPR55	Homo sapiens	G protein-coupled receptor 55(GPR55)
FABP2	Homo sapiens	fatty acid binding protein 2(FABP2)
LRG1	Homo sapiens	leucine rich alpha-2-glycoprotein 1(LRG1)
LYRM4	Homo sapiens	LYR motif containing 4(LYRM4)
APEX1	Homo sapiens	apurinic/apyrimidinic endodeoxyribonuclease 1(APEX1)
NCAPD3	Homo sapiens	non-SMC condensin II complex subunit D3(NCAPD3)
KCCAT333	Homo sapiens	renal clear cell carcinoma-associated transcript 333(KCCAT333)
ADGRL2	Homo sapiens	adhesion G protein-coupled receptor L2(ADGRL2)
LINC01475	Homo sapiens	long intergenic non-protein coding RNA 1475(LINC01475)
GPR65	Homo sapiens	G protein-coupled receptor 65(GPR65)
REG1A	Homo sapiens	regenerating family member 1 alpha(REG1A)
ATN1	Homo sapiens	atrophin 1(ATN1)
RORC	Homo sapiens	RAR related orphan receptor C(RORC)
MSI1	Homo sapiens	musashi RNA binding protein 1(MSI1)
DEFB1	Homo sapiens	defensin beta 1(DEFB1)
CXCL13	Homo sapiens	C-X-C motif chemokine ligand 13(CXCL13)
APEH	Homo sapiens	acylaminoacyl-peptide hydrolase(APEH)
HNMT	Homo sapiens	histamine N-methyltransferase(HNMT)
SAT1	Homo sapiens	spermidine/spermine N1-acetyltransferase 1(SAT1)
CXCL16	Homo sapiens	C-X-C motif chemokine ligand 16(CXCL16)
CTGF	Homo sapiens	connective tissue growth factor(CTGF)
MFSD13A	Homo sapiens	major facilitator superfamily domain containing 13A(MFSD13A)
HPGDS	Homo sapiens	hematopoietic prostaglandin D synthase(HPGDS)
ADORA3	Homo sapiens	adenosine A3 receptor(ADORA3)
TOM1	Homo sapiens	target of myb1 membrane trafficking protein(TOM1)
LEPR	Homo sapiens	leptin receptor(LEPR)
MAP3K8	Homo sapiens	mitogen-activated protein kinase kinase kinase 8(MAP3K8)
IFNA13	Homo sapiens	interferon alpha 13(IFNA13)
S100A1	Homo sapiens	S100 calcium binding protein A1(S100A1)
FAM118A	Homo sapiens	family with sequence similarity 118 member A(FAM118A)
MUL1	Homo sapiens	mitochondrial E3 ubiquitin protein ligase 1(MUL1)
FOXP3	Homo sapiens	forkhead box P3(FOXP3)
ATXN2L	Homo sapiens	ataxin 2 like(ATXN2L)
SLC9A3	Homo sapiens	solute carrier family 9 member A3(SLC9A3)

MAF	Homo sapiens	MAF bZIP transcription factor(MAF)
MSH2	Homo sapiens	mutS homolog 2(MSH2)
SMO	Homo sapiens	smoothened, frizzled class receptor(SMO)
SLC9A8	Homo sapiens	solute carrier family 9 member A8(SLC9A8)
MSH5	Homo sapiens	mutS homolog 5(MSH5)
NAAA	Homo sapiens	N-acylethanolamine acid amidase(NAAA)
S100A9	Homo sapiens	S100 calcium binding protein A9(S100A9)
S100A8	Homo sapiens	S100 calcium binding protein A8(S100A8)
LINC01250	Homo sapiens	long intergenic non-protein coding RNA 1250(LINC01250)
CREM	Homo sapiens	cAMP responsive element modulator(CREM)
GSTT1	Homo sapiens	glutathione S-transferase theta 1(GSTT1)
THADA	Homo sapiens	THADA, armadillo repeat containing(THADA)
TNFRSF11A	Homo sapiens	TNF receptor superfamily member 11a(TNFRSF11A)
FOXO3	Homo sapiens	forkhead box O3(FOXO3)
FHIT	Homo sapiens	fragile histidine triad(FHIT)
ACAT2	Homo sapiens	acetyl-CoA acetyltransferase 2(ACAT2)
SLC9A1	Homo sapiens	solute carrier family 9 member A1(SLC9A1)
HSP90B1	Homo sapiens	heat shock protein 90 beta family member 1(HSP90B1)
SOCS3	Homo sapiens	suppressor of cytokine signaling 3(SOCS3)
SBNO2	Homo sapiens	strawberry notch homolog 2(SBNO2)
SOCS1	Homo sapiens	suppressor of cytokine signaling 1(SOCS1)
RAP1A	Homo sapiens	RAP1A, member of RAS oncogene family(RAP1A)
TNFRSF17	Homo sapiens	TNF receptor superfamily member 17(TNFRSF17)
TNFRSF14	Homo sapiens	TNF receptor superfamily member 14(TNFRSF14)
IGLL2P	Homo sapiens	immunoglobulin lambda like polypeptide 2, pseudogene(IGLL2P)
DEFA6	Homo sapiens	defensin alpha 6(DEFA6)
TNFRSF9	Homo sapiens	TNF receptor superfamily member 9(TNFRSF9)
DEFA5	Homo sapiens	defensin alpha 5(DEFA5)
AICDA	Homo sapiens	activation induced cytidine deaminase(AICDA)
NFKB1	Homo sapiens	nuclear factor kappa B subunit 1(NFKB1)
NFKB2	Homo sapiens	nuclear factor kappa B subunit 2(NFKB2)
CXCL10	Homo sapiens	C-X-C motif chemokine ligand 10(CXCL10)
CXCL12	Homo sapiens	C-X-C motif chemokine ligand 12(CXCL12)
PTPRC	Homo sapiens	protein tyrosine phosphatase, receptor type C(PTPRC)
ARPC2	Homo sapiens	actin related protein 2/3 complex subunit 2(ARPC2)
MDM2	Homo sapiens	MDM2 proto-oncogene(MDM2)
LCN2	Homo sapiens	lipocalin 2(LCN2)
FGFR1OP	Homo sapiens	FGFR1 oncogene partner(FGFR1OP)
HLA-DRA	Homo sapiens	major histocompatibility complex, class II, DR alpha(HLA-DRA)
MYD88	Homo sapiens	myeloid differentiation primary response 88(MYD88)
NFE2L2	Homo sapiens	nuclear factor, erythroid 2 like 2(NFE2L2)
ITGB1	Homo sapiens	integrin subunit beta 1(ITGB1)
FLT1	Homo sapiens	fms related tyrosine kinase 1(FLT1)
ITGAM	Homo sapiens	integrin subunit alpha M(ITGAM)
LRRK2	Homo sapiens	leucine rich repeat kinase 2(LRRK2)
FHL2	Homo sapiens	four and a half LIM domains 2(FHL2)
ARRB2	Homo sapiens	arrestin beta 2(ARRB2)
ITGAE	Homo sapiens	integrin subunit alpha E(ITGAE)
ITGAL	Homo sapiens	integrin subunit alpha L(ITGAL)
WISP1	Homo sapiens	WNT1 inducible signaling pathway protein 1(WISP1)
TNFSF13B	Homo sapiens	tumor necrosis factor superfamily member 13b(TNFSF13B)
FCGR3A	Homo sapiens	Fc fragment of IgG receptor IIIa(FCGR3A)
FCGR3B	Homo sapiens	Fc fragment of IgG receptor IIIb(FCGR3B)
LOC102723407	Homo sapiens	and immunoglobulin domain-containing-like protein IGHV4OR15-8(L[illegible]
mTOR	Homo sapiens	mechanistic target of rapamycin(MTOR)
ITGAX	Homo sapiens	integrin subunit alpha X(ITGAX)
ITGB7	Homo sapiens	integrin subunit beta 7(ITGB7)
ACP1	Homo sapiens	acid phosphatase 1, soluble(ACP1)
CTNNBL1	Homo sapiens	catenin beta like 1(CTNNBL1)
MBL2	Homo sapiens	mannose binding lectin 2(MBL2)
HSP90AA1	Homo sapiens	heat shock protein 90 alpha family class A member 1(HSP90AA1)
KSR1	Homo sapiens	kinase suppressor of ras 1(KSR1)
ITGA4	Homo sapiens	integrin subunit alpha 4(ITGA4)
AHSA1	Homo sapiens	activator of Hsp90 ATPase activity 1(AHSA1)

HTR3A	Homo sapiens	5-hydroxytryptamine receptor 3A(HTR3A)
TNFRSF1B	Homo sapiens	TNF receptor superfamily member 1B(TNFRSF1B)
TNFRSF1A	Homo sapiens	TNF receptor superfamily member 1A(TNFRSF1A)
RRM2B	Homo sapiens	bonucleotide reductase regulatory TP53 inducible subunit M2B(RRM2I
TMBIM1	Homo sapiens	transmembrane BAX inhibitor motif containing 1(TMBIM1)
BPI	Homo sapiens	bactericidal/permeability-increasing protein(BPI)
DSG2	Homo sapiens	desmoglein 2(DSG2)
OR10A4	Homo sapiens	olfactory receptor family 10 subfamily A member 4(OR10A4)
MET	Homo sapiens	MET proto-oncogene, receptor tyrosine kinase(MET)
BIRC2	Homo sapiens	baculoviral IAP repeat containing 2(BIRC2)
BIRC3	Homo sapiens	baculoviral IAP repeat containing 3(BIRC3)
LRP5	Homo sapiens	LDL receptor related protein 5(LRP5)
NTN1	Homo sapiens	netrin 1(NTN1)
LRP6	Homo sapiens	LDL receptor related protein 6(LRP6)
CCL8	Homo sapiens	C-C motif chemokine ligand 8(CCL8)
CCL5	Homo sapiens	C-C motif chemokine ligand 5(CCL5)
BAHD1	Homo sapiens	bromo adjacent homology domain containing 1(BAHD1)
CCL4	Homo sapiens	C-C motif chemokine ligand 4(CCL4)
CCL3	Homo sapiens	C-C motif chemokine ligand 3(CCL3)
CCL2	Homo sapiens	C-C motif chemokine ligand 2(CCL2)
E2F4	Homo sapiens	E2F transcription factor 4(E2F4)
SH2B3	Homo sapiens	SH2B adaptor protein 3(SH2B3)
LINC00598	Homo sapiens	long intergenic non-protein coding RNA 598(LINC00598)
ERAP2	Homo sapiens	endoplasmic reticulum aminopeptidase 2(ERAP2)
GZMA	Homo sapiens	granzyme A(GZMA)
ERAP1	Homo sapiens	endoplasmic reticulum aminopeptidase 1(ERAP1)
GZMB	Homo sapiens	granzyme B(GZMB)
TPD52L1	Homo sapiens	tumor protein D52-like 1(TPD52L1)
LOC105369230	Homo sapiens	A class II histocompatibility antigen, DRB1-7 beta chain(LOC1053692
ST2	Homo sapiens	suppression of tumorigenicity 2(ST2)
NELL1	Homo sapiens	neural EGFL like 1(NELL1)
TSPAN14	Homo sapiens	tetraspanin 14(TSPAN14)
FCGR2A	Homo sapiens	Fc fragment of IgG receptor IIa(FCGR2A)
SST	Homo sapiens	somatostatin(SST)
IL2RA	Homo sapiens	interleukin 2 receptor subunit alpha(IL2RA)
STUB1	Homo sapiens	STIP1 homology and U-box containing protein 1(STUB1)
P4HB	Homo sapiens	prolyl 4-hydroxylase subunit beta(P4HB)
PDCD1	Homo sapiens	programmed cell death 1(PDCD1)
PTPN2	Homo sapiens	protein tyrosine phosphatase, non-receptor type 2(PTPN2)
TNFRSF6B	Homo sapiens	TNF receptor superfamily member 6b(TNFRSF6B)
HHIP	Homo sapiens	hedgehog interacting protein(HHIP)
SLC23A1	Homo sapiens	solute carrier family 23 member 1(SLC23A1)
ICAM2	Homo sapiens	intercellular adhesion molecule 2(ICAM2)
KEAP1	Homo sapiens	kelch like ECH associated protein 1(KEAP1)
IKZF1	Homo sapiens	IKAROS family zinc finger 1(IKZF1)
UBE2L3	Homo sapiens	ubiquitin conjugating enzyme E2 L3(UBE2L3)
ICAM1	Homo sapiens	intercellular adhesion molecule 1(ICAM1)
LGALS4	Homo sapiens	galectin 4(LGALS4)
LGALS3	Homo sapiens	galectin 3(LGALS3)
RASSF1	Homo sapiens	Ras association domain family member 1(RASSF1)
HERC2	Homo sapiens	IECT and RLD domain containing E3 ubiquitin protein ligase 2(HERC2
KDR	Homo sapiens	kinase insert domain receptor(KDR)
IL12B	Homo sapiens	interleukin 12B(IL12B)
KIF21B	Homo sapiens	kinesin family member 21B(KIF21B)
LGALS9	Homo sapiens	galectin 9(LGALS9)
EPHB2	Homo sapiens	EPH receptor B2(EPHB2)
BSN	Homo sapiens	bassoon presynaptic cytomatrix protein(BSN)
SLC12A9	Homo sapiens	solute carrier family 12 member 9(SLC12A9)
ARSA	Homo sapiens	arylsulfatase A(ARSA)
SFMBT1	Homo sapiens	Scm-like with four mbt domains 1(SFMBT1)
LINC01185	Homo sapiens	long intergenic non-protein coding RNA 1185(LINC01185)
TRAF3IP2-AS1	Homo sapiens	TRAF3IP2 antisense RNA 1(TRAF3IP2-AS1)
MIF	Homo sapiens	crophage migration inhibitory factor (glycosylation-inhibiting factor)(M
SFRP1	Homo sapiens	secreted frizzled related protein 1(SFRP1)

SFRP2	Homo sapiens	secreted frizzled related protein 2(SFRP2)
SLPI	Homo sapiens	secretory leukocyte peptidase inhibitor(SLPI)
CCNY	Homo sapiens	cyclin Y(CCNY)
MIP	Homo sapiens	major intrinsic protein of lens fiber(MIP)
SUB1	Homo sapiens	SUB1 homolog, transcriptional regulator(SUB1)
CD226	Homo sapiens	CD226 molecule(CD226)
TP53	Homo sapiens	tumor protein p53(TP53)
DEFB103A	Homo sapiens	defensin beta 103A(DEFB103A)
DEFB103B	Homo sapiens	defensin beta 103B(DEFB103B)
TGFA	Homo sapiens	transforming growth factor alpha(TGFA)
MST1R	Homo sapiens	macrophage stimulating 1 receptor(MST1R)
RTF1	Homo sapiens	RTF1 homolog, Paf1/RNA polymerase II complex component(RTF1)
SEC14L2	Homo sapiens	SEC14 like lipid binding 2(SEC14L2)
IGHD3-10	Homo sapiens	immunoglobulin heavy diversity 3-10(IGHD3-10)
APOH	Homo sapiens	apolipoprotein H(APOH)
CD19	Homo sapiens	CD19 molecule(CD19)
KCNN1	Homo sapiens	potassium calcium-activated channel subfamily N member 1(KCNN1)
APOE	Homo sapiens	apolipoprotein E(APOE)
CD14	Homo sapiens	CD14 molecule(CD14)
STAT5A	Homo sapiens	signal transducer and activator of transcription 5A(STAT5A)
DEFB104B	Homo sapiens	defensin beta 104B(DEFB104B)
TEL1-TNFRSF6	Homo sapiens	TEL1-TNFRSF6B readthrough (NMD candidate)(RTEL1-TNFRSF6B
EGR1	Homo sapiens	early growth response 1(EGR1)
XBP1	Homo sapiens	X-box binding protein 1(XBP1)
DEFB104A	Homo sapiens	defensin beta 104A(DEFB104A)
TNFSF15	Homo sapiens	tumor necrosis factor superfamily member 15(TNFSF15)
REG4	Homo sapiens	regenerating family member 4(REG4)
SLC10A2	Homo sapiens	solute carrier family 10 member 2(SLC10A2)
XRCC3	Homo sapiens	X-ray repair cross complementing 3(XRCC3)
IGF2	Homo sapiens	insulin like growth factor 2(IGF2)
XRCC1	Homo sapiens	X-ray repair cross complementing 1(XRCC1)
CELA1	Homo sapiens	chymotrypsin like elastase family member 1(CELA1)
ATP4A	Homo sapiens	ATPase H+/K+ transporting alpha subunit(ATP4A)
AIMP2	Homo sapiens	acyl tRNA synthetase complex interacting multifunctional protein 2(AI
TG	Homo sapiens	thyroglobulin(TG)
CD209	Homo sapiens	CD209 molecule(CD209)
WNK1	Homo sapiens	WNK lysine deficient protein kinase 1(WNK1)
LPAR6	Homo sapiens	lysophosphatidic acid receptor 6(LPAR6)
CD28	Homo sapiens	CD28 molecule(CD28)
SDC1	Homo sapiens	syndecan 1(SDC1)
PAEP	Homo sapiens	progestagen associated endometrial protein(PAEP)
CD24	Homo sapiens	CD24 molecule(CD24)
IL21	Homo sapiens	interleukin 21(IL21)
IL22	Homo sapiens	interleukin 22(IL22)
CD40	Homo sapiens	CD40 molecule(CD40)
ECM1	Homo sapiens	extracellular matrix protein 1(ECM1)
IL20	Homo sapiens	interleukin 20(IL20)
PTPRS	Homo sapiens	protein tyrosine phosphatase, receptor type S(PTPRS)
IL26	Homo sapiens	interleukin 26(IL26)
IL24	Homo sapiens	interleukin 24(IL24)
HNF4G	Homo sapiens	hepatocyte nuclear factor 4 gamma(HNF4G)
IL27	Homo sapiens	interleukin 27(IL27)
MLN	Homo sapiens	motilin(MLN)
MYDGF	Homo sapiens	myeloid derived growth factor(MYDGF)
HNF4A	Homo sapiens	hepatocyte nuclear factor 4 alpha(HNF4A)
CCR9	Homo sapiens	C-C motif chemokine receptor 9(CCR9)
MUC12	Homo sapiens	mucin 12, cell surface associated(MUC12)
DHX16	Homo sapiens	DEAH-box helicase 16(DHX16)
MUC13	Homo sapiens	mucin 13, cell surface associated(MUC13)
CCR7	Homo sapiens	C-C motif chemokine receptor 7(CCR7)
CCR6	Homo sapiens	C-C motif chemokine receptor 6(CCR6)
RAC1	Homo sapiens	3 botulinum toxin substrate 1 (rho family, small GTP binding protein R
CCR5	Homo sapiens	C-C motif chemokine receptor 5 (gene/pseudogene)(CCR5)
CD34	Homo sapiens	CD34 molecule(CD34)

CD33	Homo sapiens	CD33 molecule(CD33)
CCR3	Homo sapiens	C-C motif chemokine receptor 3(CCR3)
ACAD8	Homo sapiens	acyl-CoA dehydrogenase family member 8(ACAD8)
IL10	Homo sapiens	interleukin 10(IL10)
IL11	Homo sapiens	interleukin 11(IL11)
DBNL	Homo sapiens	drebrin like(DBNL)
IL15	Homo sapiens	interleukin 15(IL15)
NXPE1	Homo sapiens	neurexophilin and PC-esterase domain family member 1(NXPE1)
SFTPD	Homo sapiens	surfactant protein D(SFTPD)
MST1	Homo sapiens	macrophage stimulating 1(MST1)
IL13	Homo sapiens	interleukin 13(IL13)
IL18	Homo sapiens	interleukin 18(IL18)
IL19	Homo sapiens	interleukin 19(IL19)
IL16	Homo sapiens	interleukin 16(IL16)
COMMD1	Homo sapiens	copper metabolism domain containing 1(COMMD1)
OSMR	Homo sapiens	oncostatin M receptor(OSMR)
C5orf56	Homo sapiens	chromosome 5 open reading frame 56(C5orf56)
IL1A	Homo sapiens	interleukin 1 alpha(IL1A)
NR5A2	Homo sapiens	nuclear receptor subfamily 5 group A member 2(NR5A2)
BANK1	Homo sapiens	B-cell scaffold protein with ankyrin repeats 1(BANK1)
IL1B	Homo sapiens	interleukin 1 beta(IL1B)
CD47	Homo sapiens	CD47 molecule(CD47)
CD44	Homo sapiens	CD44 molecule (Indian blood group)(CD44)
ARHGEF6	Homo sapiens	Rac/Cdc42 guanine nucleotide exchange factor 6(ARHGEF6)
NEDD9	Homo sapiens	[illegible]ural precursor cell expressed, developmentally down-regulated 9(NED[illegible]
RELA	Homo sapiens	RELA proto-oncogene, NF-kB subunit(RELA)
WG	Homo sapiens	Wegener granulomatosis(WG)
C5orf66	Homo sapiens	chromosome 5 open reading frame 66(C5orf66)
PAK1	Homo sapiens	p21 (RAC1) activated kinase 1(PAK1)
NFIL3	Homo sapiens	nuclear factor, interleukin 3 regulated(NFIL3)
MGAT5	Homo sapiens	[illegible] (alpha-1,6-)-glycoprotein beta-1,6-N-acetyl-glucosaminyltransferase([illegible]
HSF2	Homo sapiens	heat shock transcription factor 2(HSF2)
ORMDL3	Homo sapiens	ORMDL sphingolipid biosynthesis regulator 3(ORMDL3)
MICA	Homo sapiens	MHC class I polypeptide-related sequence A(MICA)
CD55	Homo sapiens	CD55 molecule (Cromer blood group)(CD55)
MICB	Homo sapiens	MHC class I polypeptide-related sequence B(MICB)
IL33	Homo sapiens	interleukin 33(IL33)
CD74	Homo sapiens	CD74 molecule(CD74)
IL37	Homo sapiens	interleukin 37(IL37)
IL34	Homo sapiens	interleukin 34(IL34)
MYO9B	Homo sapiens	myosin IXB(MYO9B)
MIB1	Homo sapiens	mindbomb E3 ubiquitin protein ligase 1(MIB1)
VEGFA	Homo sapiens	vascular endothelial growth factor A(VEGFA)
TRBV20OR9-2	Homo sapiens	[illegible] cell receptor beta variable 20/OR9-2 (non-functional)(TRBV20OR9-2[illegible]
PER3	Homo sapiens	period circadian clock 3(PER3)
SGSM3	Homo sapiens	small G protein signaling modulator 3(SGSM3)
IL17F	Homo sapiens	interleukin 17F(IL17F)
IL17D	Homo sapiens	interleukin 17D(IL17D)
CD68	Homo sapiens	CD68 molecule(CD68)
IL17C	Homo sapiens	interleukin 17C(IL17C)
IL17B	Homo sapiens	interleukin 17B(IL17B)
IL18R1	Homo sapiens	interleukin 18 receptor 1(IL18R1)
IL17A	Homo sapiens	interleukin 17A(IL17A)
MAD2L1	Homo sapiens	MAD2 mitotic arrest deficient-like 1 (yeast)(MAD2L1)
CD86	Homo sapiens	CD86 molecule(CD86)
CD80	Homo sapiens	CD80 molecule(CD80)
MIR26B	Homo sapiens	microRNA 26b(MIR26B)
BUB1B	Homo sapiens	BUB1 mitotic checkpoint serine/threonine kinase B(BUB1B)
PRDM1	Homo sapiens	PR/SET domain 1(PRDM1)
NR3C1	Homo sapiens	nuclear receptor subfamily 3 group C member 1(NR3C1)
ETS1	Homo sapiens	ETS proto-oncogene 1, transcription factor(ETS1)
MPO	Homo sapiens	myeloperoxidase(MPO)
PLB1	Homo sapiens	phospholipase B1(PLB1)
CELSR3	Homo sapiens	cadherin EGF LAG seven-pass G-type receptor 3(CELSR3)

CCND1	Homo sapiens	cyclin D1(CCND1)
DMBT1	Homo sapiens	deleted in malignant brain tumors 1(DMBT1)
PLAU	Homo sapiens	plasminogen activator, urokinase(PLAU)
MPZ	Homo sapiens	myelin protein zero(MPZ)
DUOXA2	Homo sapiens	dual oxidase maturation factor 2(DUOXA2)
RAG2	Homo sapiens	recombination activating 2(RAG2)
IL6R	Homo sapiens	interleukin 6 receptor(IL6R)
TGM2	Homo sapiens	transglutaminase 2(TGM2)
PDGFRB	Homo sapiens	platelet derived growth factor receptor beta(PDGFRB)
ZGPAT	Homo sapiens	zinc finger CCCH-type and G-patch domain containing(ZGPAT)
IL4R	Homo sapiens	interleukin 4 receptor(IL4R)
UBE4A	Homo sapiens	ubiquitination factor E4A(UBE4A)
CDKAL1	Homo sapiens	CDK5 regulatory subunit associated protein 1 like 1(CDKAL1)
MUC3A	Homo sapiens	mucin 3A, cell surface associated(MUC3A)
MR1	Homo sapiens	major histocompatibility complex, class I-related(MR1)
ERN1	Homo sapiens	endoplasmic reticulum to nucleus signaling 1(ERN1)
SCNN1G	Homo sapiens	sodium channel epithelial 1 gamma subunit(SCNN1G)
PROCR	Homo sapiens	protein C receptor(PROCR)
SCNN1B	Homo sapiens	sodium channel epithelial 1 beta subunit(SCNN1B)
MASP2	Homo sapiens	mannan binding lectin serine peptidase 2(MASP2)
CALCA	Homo sapiens	calcitonin related polypeptide alpha(CALCA)
ARHGEF28	Homo sapiens	Rho guanine nucleotide exchange factor 28(ARHGEF28)
FUT2	Homo sapiens	fucosyltransferase 2(FUT2)
ACACA	Homo sapiens	acetyl-CoA carboxylase alpha(ACACA)
FUT3	Homo sapiens	fucosyltransferase 3 (Lewis blood group)(FUT3)
NCKIPSD	Homo sapiens	NCK interacting protein with SH3 domain(NCKIPSD)
IRAK1	Homo sapiens	interleukin 1 receptor associated kinase 1(IRAK1)
IRAK2	Homo sapiens	interleukin 1 receptor associated kinase 2(IRAK2)
TAC1	Homo sapiens	tachykinin precursor 1(TAC1)
BRINP3	Homo sapiens	BMP/retinoic acid inducible neural specific 3(BRINP3)
GCKR	Homo sapiens	glucokinase regulator(GCKR)
VCAM1	Homo sapiens	vascular cell adhesion molecule 1(VCAM1)
SYT1	Homo sapiens	synaptotagmin 1(SYT1)
VDR	Homo sapiens	vitamin D (1,25- dihydroxyvitamin D3) receptor(VDR)
RARRES3	Homo sapiens	retinoic acid receptor responder 3(RARRES3)
RARRES2	Homo sapiens	retinoic acid receptor responder 2(RARRES2)
SLC52A1	Homo sapiens	solute carrier family 52 member 1(SLC52A1)
BMP7	Homo sapiens	bone morphogenetic protein 7(BMP7)
IL2	Homo sapiens	interleukin 2(IL2)
GNAO1	Homo sapiens	G protein subunit alpha o1(GNAO1)
IL4	Homo sapiens	interleukin 4(IL4)
ISG20	Homo sapiens	interferon stimulated exonuclease gene 20(ISG20)
GH1	Homo sapiens	growth hormone 1(GH1)
IL3	Homo sapiens	interleukin 3(IL3)
H1F0	Homo sapiens	H1 histone family member 0(H1F0)
IL6	Homo sapiens	interleukin 6(IL6)
IL5	Homo sapiens	interleukin 5(IL5)
IL17REL	Homo sapiens	interleukin 17 receptor E like(IL17REL)
NAT1	Homo sapiens	N-acetyltransferase 1(NAT1)
NAT2	Homo sapiens	N-acetyltransferase 2(NAT2)
IL7	Homo sapiens	interleukin 7(IL7)
IL9	Homo sapiens	interleukin 9(IL9)
IGHV3OR16-7	Homo sapiens	mmunoglobulin heavy variable 3/OR16-7 (pseudogene)(IGHV3OR16-7
ALPI	Homo sapiens	alkaline phosphatase, intestinal(ALPI)
TCF4	Homo sapiens	transcription factor 4(TCF4)
IL7R	Homo sapiens	interleukin 7 receptor(IL7R)
MUC20	Homo sapiens	mucin 20, cell surface associated(MUC20)
MAST3	Homo sapiens	microtubule associated serine/threonine kinase 3(MAST3)
TNFAIP3	Homo sapiens	TNF alpha induced protein 3(TNFAIP3)
MTR	Homo sapiens	5-methyltetrahydrofolate-homocysteine methyltransferase(MTR)
LAMC2	Homo sapiens	laminin subunit gamma 2(LAMC2)
HMGB1	Homo sapiens	high mobility group box 1(HMGB1)
CA1	Homo sapiens	carbonic anhydrase 1(CA1)
CYP26B1	Homo sapiens	cytochrome P450 family 26 subfamily B member 1(CYP26B1)

CA2	Homo sapiens	carbonic anhydrase 2(CA2)
MIR193A	Homo sapiens	microRNA 193a(MIR193A)
MMP7	Homo sapiens	matrix metallopeptidase 7(MMP7)
MMP1	Homo sapiens	matrix metallopeptidase 1(MMP1)
EED	Homo sapiens	embryonic ectoderm development(EED)
DDX58	Homo sapiens	DExD/H-box helicase 58(DDX58)
MMP2	Homo sapiens	matrix metallopeptidase 2(MMP2)
MMP3	Homo sapiens	matrix metallopeptidase 3(MMP3)
TBC1D9	Homo sapiens	TBC1 domain family member 9(TBC1D9)
IRAK3	Homo sapiens	interleukin 1 receptor associated kinase 3(IRAK3)
MMP9	Homo sapiens	matrix metallopeptidase 9(MMP9)
YWHAZ	Homo sapiens	[illegible]-monooxygenase/tryptophan 5-monooxygenase activation protein zeta([illegible]
ACTA2	Homo sapiens	actin, alpha 2, smooth muscle, aorta(ACTA2)
ACTA1	Homo sapiens	actin, alpha 1, skeletal muscle(ACTA1)
VCAN	Homo sapiens	versican(VCAN)
ELF3	Homo sapiens	E74 like ETS transcription factor 3(ELF3)
IRF4	Homo sapiens	interferon regulatory factor 4(IRF4)
MYOD1	Homo sapiens	myogenic differentiation 1(MYOD1)
GORASP1	Homo sapiens	golgi reassembly stacking protein 1(GORASP1)
IRF1	Homo sapiens	interferon regulatory factor 1(IRF1)
CAT	Homo sapiens	catalase(CAT)
CHI3L1	Homo sapiens	chitinase 3 like 1(CHI3L1)
IRF5	Homo sapiens	interferon regulatory factor 5(IRF5)
LDAH	Homo sapiens	lipid droplet associated hydrolase(LDAH)
PLA2G1B	Homo sapiens	phospholipase A2 group IB(PLA2G1B)
DNAH8	Homo sapiens	dynein axonemal heavy chain 8(DNAH8)
SLC3A2	Homo sapiens	solute carrier family 3 member 2(SLC3A2)
ASAP2	Homo sapiens	ArfGAP with SH3 domain, ankyrin repeat and PH domain 2(ASAP2)
CLCN2	Homo sapiens	chloride voltage-gated channel 2(CLCN2)
CRYZ	Homo sapiens	crystallin zeta(CRYZ)
IL1RL1	Homo sapiens	interleukin 1 receptor like 1(IL1RL1)
CNR1	Homo sapiens	cannabinoid receptor 1(CNR1)
BMS1	Homo sapiens	BMS1, ribosome biogenesis factor(BMS1)
MXI1	Homo sapiens	MAX interactor 1, dimerization protein(MXI1)
MKNK2	Homo sapiens	MAP kinase interacting serine/threonine kinase 2(MKNK2)
RPP14	Homo sapiens	ribonuclease P/MRP subunit p14(RPP14)
ATOH1	Homo sapiens	atonal bHLH transcription factor 1(ATOH1)
CHGA	Homo sapiens	chromogranin A(CHGA)
IGAN1	Homo sapiens	IgA nephropathy(IGAN1)
EGF	Homo sapiens	epidermal growth factor(EGF)
EYA4	Homo sapiens	EYA transcriptional coactivator and phosphatase 4(EYA4)
PLA2G2A	Homo sapiens	phospholipase A2 group IIA(PLA2G2A)
LAMB1	Homo sapiens	laminin subunit beta 1(LAMB1)
S100B	Homo sapiens	S100 calcium binding protein B(S100B)
SOD2	Homo sapiens	superoxide dismutase 2, mitochondrial(SOD2)
SUOX	Homo sapiens	sulfite oxidase(SUOX)
MAPK14	Homo sapiens	mitogen-activated protein kinase 14(MAPK14)
IRGM	Homo sapiens	immunity related GTPase M(IRGM)
PNKD	Homo sapiens	paroxysmal nonkinesigenic dyskinesia(PNKD)
CD6	Homo sapiens	CD6 molecule(CD6)
GLB1	Homo sapiens	galactosidase beta 1(GLB1)
GALM	Homo sapiens	galactose mutarotase(GALM)
KRAS	Homo sapiens	KRAS proto-oncogene, GTPase(KRAS)
ACKR1	Homo sapiens	atypical chemokine receptor 1 (Duffy blood group)(ACKR1)
RBM45	Homo sapiens	RNA binding motif protein 45(RBM45)
VIP	Homo sapiens	vasoactive intestinal peptide(VIP)
OTUD3	Homo sapiens	OTU deubiquitinase 3(OTUD3)
MAML2	Homo sapiens	mastermind like transcriptional coactivator 2(MAML2)
SERPINE1	Homo sapiens	serpin family E member 1(SERPINE1)
SLCO6A1	Homo sapiens	solute carrier organic anion transporter family member 6A1(SLCO6A1)
ENO1	Homo sapiens	enolase 1(ENO1)
BACH2	Homo sapiens	BTB domain and CNC homolog 2(BACH2)
ZMIZ1	Homo sapiens	zinc finger MIZ-type containing 1(ZMIZ1)
NAMPT	Homo sapiens	nicotinamide phosphoribosyltransferase(NAMPT)

PROK2	Homo sapiens	prokineticin 2(PROK2)
PRKCB	Homo sapiens	protein kinase C beta(PRKCB)
SERPINF1	Homo sapiens	serpin family F member 1(SERPINF1)
EIM	Homo sapiens	Infantile myoclonic epilepsy(EIM)
LGALS14	Homo sapiens	galectin 14(LGALS14)
FNDC3A	Homo sapiens	fibronectin type III domain containing 3A(FNDC3A)
RNF128	Homo sapiens	ring finger protein 128, E3 ubiquitin protein ligase(RNF128)
BIN1	Homo sapiens	bridging integrator 1(BIN1)
PARD3	Homo sapiens	par-3 family cell polarity regulator(PARD3)
PRKCQ	Homo sapiens	protein kinase C theta(PRKCQ)
CFB	Homo sapiens	complement factor B(CFB)
CDKN2B-AS1	Homo sapiens	CDKN2B antisense RNA 1(CDKN2B-AS1)
MEFV	Homo sapiens	Mediterranean fever(MEFV)
MIR214	Homo sapiens	microRNA 214(MIR214)
RIC8B	Homo sapiens	RIC8 guanine nucleotide exchange factor B(RIC8B)
TERT	Homo sapiens	telomerase reverse transcriptase(TERT)
SERPINH1	Homo sapiens	serpin family H member 1(SERPINH1)
PLCG2	Homo sapiens	phospholipase C gamma 2(PLCG2)
S100A12	Homo sapiens	S100 calcium binding protein A12(S100A12)
HMOX1	Homo sapiens	heme oxygenase 1(HMOX1)
PROM1	Homo sapiens	prominin 1(PROM1)
IGHA1	Homo sapiens	immunoglobulin heavy constant alpha 1(IGHA1)
NKX2-3	Homo sapiens	NK2 homeobox 3(NKX2-3)
FZD1	Homo sapiens	frizzled class receptor 1(FZD1)
GSDMB	Homo sapiens	gasdermin B(GSDMB)
ST14	Homo sapiens	suppression of tumorigenicity 14(ST14)
FZD3	Homo sapiens	frizzled class receptor 3(FZD3)
NDFIP1	Homo sapiens	Nedd4 family interacting protein 1(NDFIP1)
ACE	Homo sapiens	angiotensin I converting enzyme(ACE)
FZD5	Homo sapiens	frizzled class receptor 5(FZD5)
FZD4	Homo sapiens	frizzled class receptor 4(FZD4)
PLK2	Homo sapiens	polo like kinase 2(PLK2)
SETD1A	Homo sapiens	SET domain containing 1A(SETD1A)
GP2	Homo sapiens	glycoprotein 2(GP2)
MTHFR	Homo sapiens	methylenetetrahydrofolate reductase(MTHFR)
NFATC2	Homo sapiens	nuclear factor of activated T-cells 2(NFATC2)
BRAF	Homo sapiens	B-Raf proto-oncogene, serine/threonine kinase(BRAF)
NFATC1	Homo sapiens	nuclear factor of activated T-cells 1(NFATC1)
NR1D2	Homo sapiens	nuclear receptor subfamily 1 group D member 2(NR1D2)
PRRC2A	Homo sapiens	proline rich coiled-coil 2A(PRRC2A)
FIBP	Homo sapiens	FGF1 intracellular binding protein(FIBP)
TCN2	Homo sapiens	transcobalamin 2(TCN2)
TOLLIP	Homo sapiens	toll interacting protein(TOLLIP)
REL	Homo sapiens	REL proto-oncogene, NF-kB subunit(REL)
ITPA	Homo sapiens	inosine triphosphatase(ITPA)
DENND1B	Homo sapiens	DENN domain containing 1B(DENND1B)
CDKN1A	Homo sapiens	cyclin dependent kinase inhibitor 1A(CDKN1A)
SERPINA1	Homo sapiens	serpin family A member 1(SERPINA1)
MIR629	Homo sapiens	microRNA 629(MIR629)
GLI1	Homo sapiens	GLI family zinc finger 1(GLI1)
SLC6A4	Homo sapiens	solute carrier family 6 member 4(SLC6A4)
IFIH1	Homo sapiens	interferon induced with helicase C domain 1(IFIH1)
TIMP1	Homo sapiens	TIMP metallopeptidase inhibitor 1(TIMP1)
IGHV3-69-1	Homo sapiens	immunoglobulin heavy variable 3-69-1 (pseudogene)(IGHV3-69-1)
ZNF365	Homo sapiens	zinc finger protein 365(ZNF365)
HAVCR2	Homo sapiens	hepatitis A virus cellular receptor 2(HAVCR2)
ANXA1	Homo sapiens	annexin A1(ANXA1)
IFNGR2	Homo sapiens	interferon gamma receptor 2 (interferon gamma transducer 1)(IFNGR2)
TACR2	Homo sapiens	tachykinin receptor 2(TACR2)
TET2	Homo sapiens	tet methylcytosine dioxygenase 2(TET2)
TACR1	Homo sapiens	tachykinin receptor 1(TACR1)
PAX5	Homo sapiens	paired box 5(PAX5)
TERF2	Homo sapiens	telomeric repeat binding factor 2(TERF2)
CIT	Homo sapiens	citron rho-interacting serine/threonine kinase(CIT)

KAT2A	Homo sapiens	lysine acetyltransferase 2A(KAT2A)
AFTPH	Homo sapiens	aftiphilin(AFTPH)
HLA-DQB2	Homo sapiens	major histocompatibility complex, class II, DQ beta 2(HLA-DQB2)
GAS7	Homo sapiens	growth arrest specific 7(GAS7)
HLA-DQB1	Homo sapiens	major histocompatibility complex, class II, DQ beta 1(HLA-DQB1)
ENG	Homo sapiens	endoglin(ENG)
NPSR1	Homo sapiens	neuropeptide S receptor 1(NPSR1)
CCL11	Homo sapiens	C-C motif chemokine ligand 11(CCL11)
SF3B6	Homo sapiens	splicing factor 3b subunit 6(SF3B6)
ANKRD55	Homo sapiens	ankyrin repeat domain 55(ANKRD55)
USO1	Homo sapiens	USO1 vesicle transport factor(USO1)
NR1I2	Homo sapiens	nuclear receptor subfamily 1 group I member 2(NR1I2)
ADCY3	Homo sapiens	adenylate cyclase 3(ADCY3)
ADCY7	Homo sapiens	adenylate cyclase 7(ADCY7)
CCL25	Homo sapiens	C-C motif chemokine ligand 25(CCL25)
CCL24	Homo sapiens	C-C motif chemokine ligand 24(CCL24)
AOC1	Homo sapiens	amine oxidase, copper containing 1(AOC1)
TIGAR	Homo sapiens	TP53 induced glycolysis regulatory phosphatase(TIGAR)
CCL21	Homo sapiens	C-C motif chemokine ligand 21(CCL21)
HSPA1L	Homo sapiens	heat shock protein family A (Hsp70) member 1 like(HSPA1L)
MGMT	Homo sapiens	O-6-methylguanine-DNA methyltransferase(MGMT)
CCL20	Homo sapiens	C-C motif chemokine ligand 20(CCL20)
CDKN2A	Homo sapiens	cyclin dependent kinase inhibitor 2A(CDKN2A)
IL10RB	Homo sapiens	interleukin 10 receptor subunit beta(IL10RB)
CARD8	Homo sapiens	caspase recruitment domain family member 8(CARD8)
NR1H2	Homo sapiens	nuclear receptor subfamily 1 group H member 2(NR1H2)
IL10RA	Homo sapiens	interleukin 10 receptor subunit alpha(IL10RA)
CARD9	Homo sapiens	caspase recruitment domain family member 9(CARD9)
NR1H4	Homo sapiens	nuclear receptor subfamily 1 group H member 4(NR1H4)
HIPK1	Homo sapiens	homeodomain interacting protein kinase 1(HIPK1)
C17orf67	Homo sapiens	chromosome 17 open reading frame 67(C17orf67)
LAMTOR2	Homo sapiens	endosomal/lysosomal adaptor, MAPK and MTOR activator 2(LAMTO
HSPA1B	Homo sapiens	heat shock protein family A (Hsp70) member 1B(HSPA1B)
CCL26	Homo sapiens	C-C motif chemokine ligand 26(CCL26)
HSPA1A	Homo sapiens	heat shock protein family A (Hsp70) member 1A(HSPA1A)
RNU1-4	Homo sapiens	RNA, U1 small nuclear 4(RNU1-4)
PUS10	Homo sapiens	pseudouridylate synthase 10(PUS10)
RNU1-1	Homo sapiens	RNA, U1 small nuclear 1(RNU1-1)
TPMT	Homo sapiens	thiopurine S-methyltransferase(TPMT)
TUSC3	Homo sapiens	tumor suppressor candidate 3(TUSC3)
MIR146A	Homo sapiens	microRNA 146a(MIR146A)
AHR	Homo sapiens	aryl hydrocarbon receptor(AHR)
PARK7	Homo sapiens	Parkinsonism associated deglycase(PARK7)
BEST2	Homo sapiens	bestrophin 2(BEST2)
FGF2	Homo sapiens	fibroblast growth factor 2(FGF2)
TNF	Homo sapiens	tumor necrosis factor(TNF)
CLU	Homo sapiens	clusterin(CLU)
IGHG3	Homo sapiens	immunoglobulin heavy constant gamma 3 (G3m marker)(IGHG3)
NPEPPS	Homo sapiens	aminopeptidase puromycin sensitive(NPEPPS)
FGF7	Homo sapiens	fibroblast growth factor 7(FGF7)
CDH3	Homo sapiens	cadherin 3(CDH3)
CDH1	Homo sapiens	cadherin 1(CDH1)
GRAP2	Homo sapiens	GRB2-related adaptor protein 2(GRAP2)
RNF19A	Homo sapiens	ring finger protein 19A, RBR E3 ubiquitin protein ligase(RNF19A)
CHP1	Homo sapiens	calcineurin like EF-hand protein 1(CHP1)
IL13RA2	Homo sapiens	interleukin 13 receptor subunit alpha 2(IL13RA2)
SLC15A1	Homo sapiens	solute carrier family 15 member 1(SLC15A1)
CMM	Homo sapiens	cutaneous malignant melanoma/dysplastic nevus(CMM)
MAP2K2	Homo sapiens	mitogen-activated protein kinase kinase 2(MAP2K2)
TPM3	Homo sapiens	tropomyosin 3(TPM3)
SEMA6D	Homo sapiens	semaphorin 6D(SEMA6D)
MAGI2	Homo sapiens	:ane associated guanylate kinase, WW and PDZ domain containing 2(M
PGD	Homo sapiens	phosphogluconate dehydrogenase(PGD)
HIC1	Homo sapiens	HIC ZBTB transcriptional repressor 1(HIC1)

TRAIP	Homo sapiens	TRAF interacting protein(TRAIP)
IAPP	Homo sapiens	islet amyloid polypeptide(IAPP)
FKBP5	Homo sapiens	FK506 binding protein 5(FKBP5)
NOTCH1	Homo sapiens	notch 1(NOTCH1)
ASMT	Homo sapiens	acetylserotonin O-methyltransferase(ASMT)
KNG1	Homo sapiens	kininogen 1(KNG1)
MIR4728	Homo sapiens	microRNA 4728(MIR4728)
CLEC7A	Homo sapiens	C-type lectin domain family 7 member A(CLEC7A)
PI3	Homo sapiens	peptidase inhibitor 3(PI3)
MAP2K7	Homo sapiens	mitogen-activated protein kinase kinase 7(MAP2K7)
ZNF300	Homo sapiens	zinc finger protein 300(ZNF300)
CD163	Homo sapiens	CD163 molecule(CD163)
CD160	Homo sapiens	CD160 molecule(CD160)
BW35	Homo sapiens	Body weight QTL 35(BW35)
DUPD1	Homo sapiens	ıl specificity phosphatase and pro isomerase domain containing 1(DUPI
INS-IGF2	Homo sapiens	INS-IGF2 readthrough(INS-IGF2)
ALB	Homo sapiens	albumin(ALB)
BCL3	Homo sapiens	B-cell CLL/lymphoma 3(BCL3)
BCL2	Homo sapiens	BCL2, apoptosis regulator(BCL2)
REEP6	Homo sapiens	receptor accessory protein 6(REEP6)
RNF186	Homo sapiens	ring finger protein 186(RNF186)
SLC26A3	Homo sapiens	solute carrier family 26 member 3(SLC26A3)
PTPN22	Homo sapiens	protein tyrosine phosphatase, non-receptor type 22(PTPN22)
LSP1	Homo sapiens	lymphocyte-specific protein 1(LSP1)
DUSP16	Homo sapiens	dual specificity phosphatase 16(DUSP16)
TREM1	Homo sapiens	triggering receptor expressed on myeloid cells 1(TREM1)
FCAR	Homo sapiens	Fc fragment of IgA receptor(FCAR)
GAL3ST2	Homo sapiens	galactose-3-O-sulfotransferase 2(GAL3ST2)
LOC390714	Homo sapiens	immunoglobulin heavy chain variable region(LOC390714)
MSH5-SAPCD1	Homo sapiens	MSH5-SAPCD1 readthrough (NMD candidate)(MSH5-SAPCD1)
UGT1A1	Homo sapiens	UDP glucuronosyltransferase family 1 member A1(UGT1A1)
DNMT3A	Homo sapiens	DNA methyltransferase 3 alpha(DNMT3A)
MEPE	Homo sapiens	matrix extracellular phosphoglycoprotein(MEPE)
APOA4	Homo sapiens	apolipoprotein A4(APOA4)
NGF	Homo sapiens	nerve growth factor(NGF)
YDJC	Homo sapiens	YdjC homolog (bacterial)(YDJC)
ADRA2A	Homo sapiens	adrenoceptor alpha 2A(ADRA2A)
ZFP90	Homo sapiens	ZFP90 zinc finger protein(ZFP90)
MEP1A	Homo sapiens	meprin A subunit alpha(MEP1A)
LURAP1L-AS1	Homo sapiens	LURAP1L antisense RNA 1(LURAP1L-AS1)
PECAM1	Homo sapiens	platelet and endothelial cell adhesion molecule 1(PECAM1)
CRH	Homo sapiens	corticotropin releasing hormone(CRH)
LCT	Homo sapiens	lactase(LCT)
CRK	Homo sapiens	CRK proto-oncogene, adaptor protein(CRK)
CRP	Homo sapiens	C-reactive protein(CRP)
CX3CR1	Homo sapiens	C-X3-C motif chemokine receptor 1(CX3CR1)
LINC00484	Homo sapiens	long intergenic non-protein coding RNA 484(LINC00484)
NOD1	Homo sapiens	nucleotide binding oligomerization domain containing 1(NOD1)
NOD2	Homo sapiens	nucleotide binding oligomerization domain containing 2(NOD2)
CYP3A4	Homo sapiens	cytochrome P450 family 3 subfamily A member 4(CYP3A4)
CYP3A5	Homo sapiens	cytochrome P450 family 3 subfamily A member 5(CYP3A5)
MIR499A	Homo sapiens	microRNA 499a(MIR499A)
HDAC7	Homo sapiens	histone deacetylase 7(HDAC7)
UGT1A7	Homo sapiens	UDP glucuronosyltransferase family 1 member A7(UGT1A7)
JAZF1	Homo sapiens	JAZF zinc finger 1(JAZF1)
SMOX	Homo sapiens	spermine oxidase(SMOX)
PLCL1	Homo sapiens	phospholipase C like 1(PLCL1)
IBD22	Homo sapiens	Inflammatory bowel disease-22(IBD22)
PTPN11	Homo sapiens	protein tyrosine phosphatase, non-receptor type 11(PTPN11)
CFLAR	Homo sapiens	CASP8 and FADD like apoptosis regulator(CFLAR)
IBD24	Homo sapiens	Inflammatory bowel disease-24(IBD24)
RAD51	Homo sapiens	RAD51 recombinase(RAD51)
APC	Homo sapiens	APC, WNT signaling pathway regulator(APC)
LEP	Homo sapiens	leptin(LEP)

LOC107984148	Homo sapiens	beta-defensin 104A(LOC107984148)
FAAH	Homo sapiens	fatty acid amide hydrolase(FAAH)
VIPR1	Homo sapiens	vasoactive intestinal peptide receptor 1(VIPR1)
IL1RN	Homo sapiens	interleukin 1 receptor antagonist(IL1RN)
TRAF3IP2	Homo sapiens	TRAF3 interacting protein 2(TRAF3IP2)
AQP8	Homo sapiens	aquaporin 8(AQP8)
IL23R	Homo sapiens	interleukin 23 receptor(IL23R)
HDAC11	Homo sapiens	histone deacetylase 11(HDAC11)
GDE1	Homo sapiens	glycerophosphodiester phosphodiesterase 1(GDE1)
ANTXR2	Homo sapiens	anthrax toxin receptor 2(ANTXR2)
SOX2	Homo sapiens	SRY-box 2(SOX2)
CHEK1	Homo sapiens	checkpoint kinase 1(CHEK1)
PTGDS	Homo sapiens	prostaglandin D2 synthase(PTGDS)
B2M	Homo sapiens	beta-2-microglobulin(B2M)
ICOSLG	Homo sapiens	inducible T-cell costimulator ligand(ICOSLG)
IL15RA	Homo sapiens	interleukin 15 receptor subunit alpha(IL15RA)
ACSL1	Homo sapiens	acyl-CoA synthetase long-chain family member 1(ACSL1)
IL1R1	Homo sapiens	interleukin 1 receptor type 1(IL1R1)
SLC11A1	Homo sapiens	solute carrier family 11 member 1(SLC11A1)
NFKBIL1	Homo sapiens	NFKB inhibitor like 1(NFKBIL1)
IL1R2	Homo sapiens	interleukin 1 receptor type 2(IL1R2)
CMC1	Homo sapiens	C-X9-C motif containing 1(CMC1)
SLC11A2	Homo sapiens	solute carrier family 11 member 2(SLC11A2)
SLC6A14	Homo sapiens	solute carrier family 6 member 14(SLC6A14)
HLA-B	Homo sapiens	major histocompatibility complex, class I, B(HLA-B)
KRT8	Homo sapiens	keratin 8(KRT8)
KRT7	Homo sapiens	keratin 7(KRT7)
HLA-A	Homo sapiens	major histocompatibility complex, class I, A(HLA-A)
CRNKL1	Homo sapiens	crooked neck pre-mRNA splicing factor 1(CRNKL1)
RUNX3	Homo sapiens	runt related transcription factor 3(RUNX3)
HLA-G	Homo sapiens	major histocompatibility complex, class I, G(HLA-G)
IL23A	Homo sapiens	interleukin 23 subunit alpha(IL23A)
ZNF831	Homo sapiens	zinc finger protein 831(ZNF831)
CFTR	Homo sapiens	cystic fibrosis transmembrane conductance regulator(CFTR)
SLC22A4	Homo sapiens	solute carrier family 22 member 4(SLC22A4)
SLC22A5	Homo sapiens	solute carrier family 22 member 5(SLC22A5)
KLK1	Homo sapiens	kallikrein 1(KLK1)
SP140	Homo sapiens	SP140 nuclear body protein(SP140)
EBI3	Homo sapiens	Epstein-Barr virus induced 3(EBI3)
TWIST1	Homo sapiens	twist family bHLH transcription factor 1(TWIST1)
CRHR1	Homo sapiens	corticotropin releasing hormone receptor 1(CRHR1)
CRHR2	Homo sapiens	corticotropin releasing hormone receptor 2(CRHR2)
CYP27B1	Homo sapiens	cytochrome P450 family 27 subfamily B member 1(CYP27B1)
DPH5	Homo sapiens	diphthamide biosynthesis 5(DPH5)
MAGEC2	Homo sapiens	MAGE family member C2(MAGEC2)
CDR3	Homo sapiens	Cerebellar degeneration-related autoantigen-3(CDR3)
INSR	Homo sapiens	insulin receptor(INSR)
MIR200C	Homo sapiens	microRNA 200c(MIR200C)
UBAC2	Homo sapiens	UBA domain containing 2(UBAC2)
LACC1	Homo sapiens	laccase domain containing 1(LACC1)
VNN1	Homo sapiens	vanin 1(VNN1)
AKR1B10	Homo sapiens	aldo-keto reductase family 1 member B10(AKR1B10)
SLC44A4	Homo sapiens	solute carrier family 44 member 4(SLC44A4)
IFNA1	Homo sapiens	interferon alpha 1(IFNA1)
MAGT1	Homo sapiens	magnesium transporter 1(MAGT1)
KRT20	Homo sapiens	keratin 20(KRT20)
CX3CL1	Homo sapiens	C-X3-C motif chemokine ligand 1(CX3CL1)
MIR195	Homo sapiens	microRNA 195(MIR195)
MIR192	Homo sapiens	microRNA 192(MIR192)
CCHCR1	Homo sapiens	coiled-coil alpha-helical rod protein 1(CCHCR1)
HRAS	Homo sapiens	HRas proto-oncogene, GTPase(HRAS)
PSD	Homo sapiens	pleckstrin and Sec7 domain containing(PSD)
IGHD3-9	Homo sapiens	immunoglobulin heavy diversity 3-9(IGHD3-9)
CBR1	Homo sapiens	carbonyl reductase 1(CBR1)

GPBAR1	Homo sapiens	G protein-coupled bile acid receptor 1(GPBAR1)
MIR21	Homo sapiens	microRNA 21(MIR21)
TYK2	Homo sapiens	tyrosine kinase 2(TYK2)
IL17RD	Homo sapiens	interleukin 17 receptor D(IL17RD)
CBFA2T3	Homo sapiens	CBFA2/RUNX1 translocation partner 3(CBFA2T3)
TLR1	Homo sapiens	toll like receptor 1(TLR1)
SLC22A23	Homo sapiens	solute carrier family 22 member 23(SLC22A23)
LOC105379528	Homo sapiens	uncharacterized LOC105379528(LOC105379528)
CCKBR	Homo sapiens	cholecystokinin B receptor(CCKBR)
CEACAM6	Homo sapiens	carcinoembryonic antigen related cell adhesion molecule 6(CEACAM6)
CEACAM5	Homo sapiens	carcinoembryonic antigen related cell adhesion molecule 5(CEACAM5)
TLR9	Homo sapiens	toll like receptor 9(TLR9)
TFF3	Homo sapiens	trefoil factor 3(TFF3)
CLDN18	Homo sapiens	claudin 18(CLDN18)
TFF2	Homo sapiens	trefoil factor 2(TFF2)
TLR7	Homo sapiens	toll like receptor 7(TLR7)
TFF1	Homo sapiens	trefoil factor 1(TFF1)
TLR6	Homo sapiens	toll like receptor 6(TLR6)
ALDOB	Homo sapiens	aldolase, fructose-bisphosphate B(ALDOB)
TLR5	Homo sapiens	toll like receptor 5(TLR5)
TLR4	Homo sapiens	toll like receptor 4(TLR4)
TLR3	Homo sapiens	toll like receptor 3(TLR3)
TLR2	Homo sapiens	toll like receptor 2(TLR2)
GUCY2C	Homo sapiens	guanylate cyclase 2C(GUCY2C)
PTGER4	Homo sapiens	prostaglandin E receptor 4(PTGER4)
CXCR4	Homo sapiens	C-X-C motif chemokine receptor 4(CXCR4)
CXCR6	Homo sapiens	C-X-C motif chemokine receptor 6(CXCR6)
NPL	Homo sapiens	N-acetylneuraminate pyruvate lyase(NPL)
UQCR10	Homo sapiens	ubiquinol-cytochrome c reductase, complex III subunit X(UQCR10)
NT5E	Homo sapiens	5'-nucleotidase ecto(NT5E)
CXCR1	Homo sapiens	C-X-C motif chemokine receptor 1(CXCR1)
CXCR3	Homo sapiens	C-X-C motif chemokine receptor 3(CXCR3)
CXCR2	Homo sapiens	C-X-C motif chemokine receptor 2(CXCR2)
ADAD1	Homo sapiens	adenosine deaminase domain containing 1(ADAD1)
CORIN	Homo sapiens	corin, serine peptidase(CORIN)
DUOX2	Homo sapiens	dual oxidase 2(DUOX2)
LAIR1	Homo sapiens	leukocyte associated immunoglobulin like receptor 1(LAIR1)
NQO1	Homo sapiens	NAD(P)H quinone dehydrogenase 1(NQO1)
CDX2	Homo sapiens	caudal type homeobox 2(CDX2)
MLH1	Homo sapiens	mutL homolog 1(MLH1)
PDZK1IP1	Homo sapiens	PDZK1 interacting protein 1(PDZK1IP1)
LILRB4	Homo sapiens	leukocyte immunoglobulin like receptor B4(LILRB4)
SLURP1	Homo sapiens	secreted LY6/PLAUR domain containing 1(SLURP1)
CD40LG	Homo sapiens	CD40 ligand(CD40LG)
SAA1	Homo sapiens	serum amyloid A1(SAA1)
F2RL1	Homo sapiens	F2R like trypsin receptor 1(F2RL1)
FAS	Homo sapiens	Fas cell surface death receptor(FAS)
CTNNB1	Homo sapiens	catenin beta 1(CTNNB1)
APBA1	Homo sapiens	amyloid beta precursor protein binding family A member 1(APBA1)
GHRL	Homo sapiens	ghrelin and obestatin prepropeptide(GHRL)
CCL4L1	Homo sapiens	C-C motif chemokine ligand 4 like 1(CCL4L1)
CCL4L2	Homo sapiens	C-C motif chemokine ligand 4 like 2(CCL4L2)
IL18RAP	Homo sapiens	interleukin 18 receptor accessory protein(IL18RAP)
MIR155	Homo sapiens	microRNA 155(MIR155)
ARHGDIA	Homo sapiens	Rho GDP dissociation inhibitor alpha(ARHGDIA)
MIR150	Homo sapiens	microRNA 150(MIR150)
GBAP1	Homo sapiens	glucosylceramidase beta pseudogene 1(GBAP1)
GHSR	Homo sapiens	growth hormone secretagogue receptor(GHSR)
HGF	Homo sapiens	hepatocyte growth factor(HGF)
TUFM	Homo sapiens	Tu translation elongation factor, mitochondrial(TUFM)
PRTN3	Homo sapiens	proteinase 3(PRTN3)
IL21-AS1	Homo sapiens	IL21 antisense RNA 1(IL21-AS1)
ABCG2	Homo sapiens	TP binding cassette subfamily G member 2 (Junior blood group)(ABCG
ISYNA1	Homo sapiens	inositol-3-phosphate synthase 1(ISYNA1)

CLEC16A	Homo sapiens	C-type lectin domain family 16 member A(CLEC16A)
LPL	Homo sapiens	lipoprotein lipase(LPL)
MIR31	Homo sapiens	microRNA 31(MIR31)
CSF2RA	Homo sapiens	colony stimulating factor 2 receptor alpha subunit(CSF2RA)
CUZD1	Homo sapiens	CUB and zona pellucida like domains 1(CUZD1)
NLRP3	Homo sapiens	NLR family pyrin domain containing 3(NLRP3)
RIPK1	Homo sapiens	receptor interacting serine/threonine kinase 1(RIPK1)
XRCC6P5	Homo sapiens	X-ray repair cross complementing 6 pseudogene 5(XRCC6P5)
TGFB2	Homo sapiens	transforming growth factor beta 2(TGFB2)
TGFB1	Homo sapiens	transforming growth factor beta 1(TGFB1)
SMAD3	Homo sapiens	SMAD family member 3(SMAD3)
TGFB3	Homo sapiens	transforming growth factor beta 3(TGFB3)
HSPA4	Homo sapiens	heat shock protein family A (Hsp70) member 4(HSPA4)
IBD2	Homo sapiens	inflammatory bowel disease 2(IBD2)
IDH1	Homo sapiens	isocitrate dehydrogenase (NADP(+)) 1, cytosolic(IDH1)
IBD5	Homo sapiens	inflammatory bowel disease 5(IBD5)
IBD6	Homo sapiens	inflammatory bowel disease 6(IBD6)
IBD3	Homo sapiens	inflammatory bowel disease 3(IBD3)
BTNL2	Homo sapiens	butyrophilin like 2(BTNL2)
HSPA2	Homo sapiens	heat shock protein family A (Hsp70) member 2(HSPA2)
TRPV1	Homo sapiens	ansient receptor potential cation channel subfamily V member 1(TRPV
ESR1	Homo sapiens	estrogen receptor 1(ESR1)
SMAD5	Homo sapiens	SMAD family member 5(SMAD5)
SMAD7	Homo sapiens	SMAD family member 7(SMAD7)
NFKBIA	Homo sapiens	NFKB inhibitor alpha(NFKBIA)
NR4A1	Homo sapiens	nuclear receptor subfamily 4 group A member 1(NR4A1)
DAP	Homo sapiens	death associated protein(DAP)
AMACR	Homo sapiens	alpha-methylacyl-CoA racemase(AMACR)
SELL	Homo sapiens	selectin L(SELL)
LINC00824	Homo sapiens	long intergenic non-protein coding RNA 824(LINC00824)
ATG16L1	Homo sapiens	autophagy related 16 like 1(ATG16L1)
DLG5	Homo sapiens	discs large MAGUK scaffold protein 5(DLG5)
BRWD1	Homo sapiens	bromodomain and WD repeat domain containing 1(BRWD1)
CXCL6	Homo sapiens	C-X-C motif chemokine ligand 6(CXCL6)
CXCL8	Homo sapiens	C-X-C motif chemokine ligand 8(CXCL8)
CXCL1	Homo sapiens	C-X-C motif chemokine ligand 1(CXCL1)
CXCL5	Homo sapiens	C-X-C motif chemokine ligand 5(CXCL5)
CASP3	Homo sapiens	caspase 3(CASP3)
CASP1	Homo sapiens	caspase 1(CASP1)
MADCAM1	Homo sapiens	mucosal vascular addressin cell adhesion molecule 1(MADCAM1)
SCYL1	Homo sapiens	SCY1 like pseudokinase 1(SCYL1)
ABCC4	Homo sapiens	ATP binding cassette subfamily C member 4(ABCC4)
PROSER1	Homo sapiens	proline and serine rich 1(PROSER1)
DCC	Homo sapiens	DCC netrin 1 receptor(DCC)
H2AFX	Homo sapiens	H2A histone family member X(H2AFX)
SAFB	Homo sapiens	scaffold attachment factor B(SAFB)
TGFBR2	Homo sapiens	transforming growth factor beta receptor 2(TGFBR2)
DNM2	Homo sapiens	dynamin 2(DNM2)
MIR126	Homo sapiens	microRNA 126(MIR126)
MIR122	Homo sapiens	microRNA 122(MIR122)
IFNG	Homo sapiens	interferon gamma(IFNG)
CDH11	Homo sapiens	cadherin 11(CDH11)
ZPBP2	Homo sapiens	zona pellucida binding protein 2(ZPBP2)
LTA	Homo sapiens	lymphotoxin alpha(LTA)
CDH13	Homo sapiens	cadherin 13(CDH13)
TERF2IP	Homo sapiens	TERF2 interacting protein(TERF2IP)
DEFB4A	Homo sapiens	defensin beta 4A(DEFB4A)
DEFB4B	Homo sapiens	defensin beta 4B(DEFB4B)
LTF	Homo sapiens	lactotransferrin(LTF)
USP14	Homo sapiens	ubiquitin specific peptidase 14(USP14)
RGS14	Homo sapiens	regulator of G-protein signaling 14(RGS14)
HPGD	Homo sapiens	hydroxyprostaglandin dehydrogenase 15-(NAD)(HPGD)
POLDIP2	Homo sapiens	DNA polymerase delta interacting protein 2(POLDIP2)
USP12	Homo sapiens	ubiquitin specific peptidase 12(USP12)

CAMK2A	Homo sapiens	calcium/calmodulin dependent protein kinase II alpha(CAMK2A)
PON1	Homo sapiens	paraoxonase 1(PON1)
NXF1	Homo sapiens	nuclear RNA export factor 1(NXF1)
MUC2	Homo sapiens	mucin 2, oligomeric mucus/gel-forming(MUC2)
WNT11	Homo sapiens	Wnt family member 11(WNT11)
PPP2R3C	Homo sapiens	protein phosphatase 2 regulatory subunit B"gamma(PPP2R3C)
GNA12	Homo sapiens	G protein subunit alpha 12(GNA12)
S1PR1	Homo sapiens	sphingosine-1-phosphate receptor 1(S1PR1)
MAPK1	Homo sapiens	mitogen-activated protein kinase 1(MAPK1)
HES1	Homo sapiens	hes family bHLH transcription factor 1(HES1)
MUC4	Homo sapiens	mucin 4, cell surface associated(MUC4)
TCF7L2	Homo sapiens	transcription factor 7 like 2(TCF7L2)
NOS2	Homo sapiens	nitric oxide synthase 2(NOS2)
NOS3	Homo sapiens	nitric oxide synthase 3(NOS3)
B3GAT1	Homo sapiens	beta-1,3-glucuronyltransferase 1(B3GAT1)
MIR145	Homo sapiens	microRNA 145(MIR145)
RPS6KB1	Homo sapiens	ribosomal protein S6 kinase B1(RPS6KB1)
MIR143	Homo sapiens	microRNA 143(MIR143)
MIR141	Homo sapiens	microRNA 141(MIR141)
CDK2AP2	Homo sapiens	cyclin dependent kinase 2 associated protein 2(CDK2AP2)
PMP22	Homo sapiens	peripheral myelin protein 22(PMP22)
BAX	Homo sapiens	BCL2 associated X, apoptosis regulator(BAX)
