# Supplementary material for: Mechanism of Fructus Mume Pills Underlying Their Protective Effects in Rats with Acetic Acid-Inducedulcerative Colitis via the Regulation of Inflammatory Cytokines and the VEGF-PI3K/Akt-eNOS Signaling Pathway
Source: Evid Based Complement Alternat Med. 2022 May 2;2022:4621131. doi: 10.1155/2022/4621131 (PMC9129976; doi:10.1155/2022/4621131)
Supplement: Supplementary Materials — Supplement 1 FMP Quality Control Methods and Results. Supplement 2 FMP Active Compounds and Targets Supplement 3 Ulcerative Colitis Targets Supplement 4 GO Enrichment Result Supplement 5 KEGG Enrichment Results. [file 4621131.f1.zip › 4621131.f1/Supplement 4 GO enrichment results.pdf]

Summmary of GO enrichment results in FMP-UC

GO ID	GO Term	Ontology	GeneRatio	P-value	FDR	gene ID
GO:1901701	cellular response to oxygen-containing compound	BP	17	6.30E-14	1.52E-10	ENSG00000005381;ENSG00000007171;ENSG00000073756;ENSG00000087245;ENSG00000091831;ENSG00000100823;ENSG00000100985;ENSG00000104267;ENSG00000105976;ENSG00000106546;ENSG00000132170;ENSG00000143365;ENSG00000146648;ENSG00000149968;ENSG00000170890;ENSG00000171105;ENSG00000198074
GO:1901700	response to oxygen-containing compound	BP	18	1.31E-12	1.07E-09	ENSG00000005381;ENSG00000007171;ENSG00000073756;ENSG00000087245;ENSG00000091831;ENSG00000100823;ENSG00000100985;ENSG00000104267;ENSG00000105976;ENSG00000106546;ENSG00000132170;ENSG00000143365;ENSG00000146648;ENSG00000149968;ENSG00000170890;ENSG00000171105;ENSG00000186951;ENSG00000198074
GO:0070887	cellular response to chemical stimulus	BP	23	1.33E-12	1.07E-09	ENSG00000005381;ENSG00000007171;ENSG00000073756;ENSG00000087245;ENSG00000091831;ENSG00000095303;ENSG00000100823;ENSG00000100985;ENSG00000104267;ENSG00000105976;ENSG00000106546;ENSG00000128052;ENSG00000132170;ENSG00000133742;ENSG00000143365;ENSG00000146648;ENSG00000149968;ENSG00000163464;ENSG00000170890;ENSG00000171105;ENSG00000178623;ENSG00000186951;ENSG00000198074
GO:0042221	response to chemical	BP	25	2.90E-11	1.75E-08	ENSG00000005381;ENSG00000007171;ENSG00000073756;ENSG00000085563;ENSG00000087245;ENSG00000091831;ENSG00000095303;ENSG00000100823;ENSG00000100985;ENSG00000104267;ENSG00000105976;ENSG00000106546;ENSG00000118777;ENSG00000128052;ENSG00000132170;ENSG00000133742;ENSG00000143365;ENSG00000146648;ENSG00000149968;ENSG00000163464;ENSG00000170890;ENSG00000171105;ENSG00000178623;ENSG00000186951;ENSG00000198074
GO:0010033	response to organic substance	BP	21	2.60E-10	1.26E-07	ENSG00000005381;ENSG00000007171;ENSG00000073756;ENSG00000087245;ENSG00000091831;ENSG00000100823;ENSG00000100985;ENSG00000104267;ENSG00000106546;ENSG00000128052;ENSG00000132170;ENSG00000133742;ENSG00000143365;ENSG00000146648;ENSG00000149968;ENSG00000163464;ENSG00000170890;ENSG00000171105;ENSG00000178623;ENSG00000186951;ENSG00000198074
GO:0065008	regulation of biological quality	BP	22	9.47E-10	3.46E-07	ENSG00000005381;ENSG00000007171;ENSG00000073756;ENSG00000085563;ENSG00000091831;ENSG00000095303;ENSG00000100823;ENSG00000104267;ENSG00000105426;ENSG00000105976;ENSG00000118777;ENSG00000128052;ENSG00000132170;ENSG00000146648;ENSG00000148680;ENSG00000163464;ENSG00000170890;ENSG00000171105;ENSG00000178623;ENSG00000180210;ENSG00000186951;ENSG00000198074
GO:0071310	cellular response to organic substance	BP	19	1.04E-09	3.46E-07	ENSG00000007171;ENSG00000073756;ENSG00000087245;ENSG00000091831;ENSG00000100823;ENSG00000100985;ENSG00000104267;ENSG00000106546;ENSG00000128052;ENSG00000132170;ENSG00000133742;ENSG00000143365;ENSG00000146648;ENSG00000149968;ENSG00000163464;ENSG00000170890;ENSG00000171105;ENSG00000178623;ENSG00000186951
GO:0042493	response to drug	BP	13	1.15E-09	3.46E-07	ENSG00000007171;ENSG00000073756;ENSG00000085563;ENSG00000100823;ENSG00000104267;ENSG00000105976;ENSG00000106546;ENSG00000118777;ENSG00000128052;ENSG00000132170;ENSG00000146648;ENSG00000149968;ENSG00000186951
GO:0042592	homeostatic process	BP	16	2.57E-09	6.45E-07	ENSG00000005381;ENSG00000007171;ENSG00000073756;ENSG00000091831;ENSG00000100823;ENSG00000104267;ENSG00000105976;ENSG00000118777;ENSG00000128052;ENSG00000132170;ENSG00000146648;ENSG00000163464;ENSG00000170890;ENSG00000171105;ENSG00000178623;ENSG00000180210
GO:1901652	response to peptide	BP	10	2.66E-09	6.45E-07	ENSG00000073756;ENSG00000087245;ENSG00000100823;ENSG00000100985;ENSG00000104267;ENSG00000132170;ENSG00000149968;ENSG00000170890;ENSG00000171105;ENSG00000186951
GO:0034614	cellular response to reactive oxygen species	BP	7	5.70E-09	1.25E-06	ENSG00000005381;ENSG00000087245;ENSG00000100823;ENSG00000100985;ENSG00000105976;ENSG00000146648;ENSG00000149968
GO:0048511	rhythmic process	BP	8	7.54E-09	1.43E-06	ENSG00000007171;ENSG00000091831;ENSG00000106546;ENSG00000132170;ENSG00000143365;ENSG00000146648;ENSG00000148680;ENSG00000186951
GO:0032101	regulation of response to external stimulus	BP	12	8.20E-09	1.43E-06	ENSG00000007171;ENSG00000073756;ENSG00000091831;ENSG00000100985;ENSG00000105426;ENSG00000105976;ENSG00000128052;ENSG00000132170;ENSG00000146648;ENSG00000149968;ENSG00000180210;ENSG00000186951
GO:0010243	response to organonitrogen compound	BP	12	8.28E-09	1.43E-06	ENSG00000073756;ENSG00000087245;ENSG00000100823;ENSG00000100985;ENSG00000104267;ENSG00000106546;ENSG00000132170;ENSG00000146648;ENSG00000149968;ENSG00000170890;ENSG00000171105;ENSG00000186951
GO:0007623	circadian rhythm	BP	7	1.77E-08	2.85E-06	ENSG00000007171;ENSG00000106546;ENSG00000132170;ENSG00000143365;ENSG00000146648;ENSG00000148680;ENSG00000186951
GO:0006979	response to oxidative stress	BP	9	2.28E-08	3.33E-06	ENSG00000005381;ENSG00000073756;ENSG00000087245;ENSG00000095303;ENSG00000100823;ENSG00000100985;ENSG00000105976;ENSG00000146648;ENSG00000149968
GO:1901699	cellular response to nitrogen compound	BP	10	2.42E-08	3.33E-06	ENSG00000073756;ENSG00000087245;ENSG00000100823;ENSG00000104267;ENSG00000106546;ENSG00000132170;ENSG00000146648;ENSG00000149968;ENSG00000170890;ENSG00000171105
GO:1901698	response to nitrogen compound	BP	12	2.48E-08	3.33E-06	ENSG00000073756;ENSG00000087245;ENSG00000100823;ENSG00000100985;ENSG00000104267;ENSG00000106546;ENSG00000132170;ENSG00000146648;ENSG00000149968;ENSG00000170890;ENSG00000171105;ENSG00000186951
GO:0000302	response to reactive oxygen species	BP	7	4.91E-08	6.25E-06	ENSG00000005381;ENSG00000087245;ENSG00000100823;ENSG00000100985;ENSG00000105976;ENSG00000146648;ENSG00000149968
GO:0048878	chemical homeostasis	BP	12	6.32E-08	7.61E-06	ENSG00000091831;ENSG00000104267;ENSG00000105976;ENSG00000118777;ENSG00000128052;ENSG00000132170;ENSG00000146648;ENSG00000163464;ENSG00000170890;ENSG00000171105;ENSG00000178623;ENSG00000180210

GO:0035690	cellular response to drug	BP	8	6.67E-08	7.61E-06	ENSG00000007171;ENSG00000073756;ENSG00000100823;ENSG00000105976;ENSG00000106546;ENSG00000128052;ENSG00000146648;ENSG00000149968
GO:0009636	response to toxic substance	BP	9	6.92E-08	7.61E-06	ENSG00000005381;ENSG00000073756;ENSG00000095303;ENSG00000100823;ENSG00000105976;ENSG00000106546;ENSG00000128052;ENSG00000186951;ENSG00000198074
GO:0050727	regulation of inflammatory response	BP	9	8.03E-08	8.45E-06	ENSG00000007171;ENSG00000073756;ENSG00000091831;ENSG00000100985;ENSG00000132170;ENSG00000146648;ENSG00000149968;ENSG00000180210;ENSG00000186951
GO:0097237	cellular response to toxic substance	BP	7	9.76E-08	9.83E-06	ENSG00000005381;ENSG00000073756;ENSG00000095303;ENSG00000100823;ENSG00000105976;ENSG00000128052;ENSG00000198074
GO:0010035	response to inorganic substance	BP	9	1.09E-07	1.05E-05	ENSG00000005381;ENSG00000073756;ENSG00000100823;ENSG00000100985;ENSG00000104267;ENSG00000105976;ENSG00000128052;ENSG00000146648;ENSG00000149968
GO:0009725	response to hormone	BP	11	1.12E-07	1.05E-05	ENSG00000007171;ENSG00000073756;ENSG00000091831;ENSG00000100823;ENSG00000104267;ENSG00000132170;ENSG00000143365;ENSG00000146648;ENSG00000170890;ENSG00000171105;ENSG00000186951
GO:0008283	cell proliferation	BP	15	1.18E-07	1.06E-05	ENSG00000007171;ENSG00000073756;ENSG00000085563;ENSG00000087245;ENSG00000091831;ENSG00000095303;ENSG00000100985;ENSG00000105976;ENSG00000106546;ENSG00000128052;ENSG00000132170;ENSG00000146648;ENSG00000170890;ENSG00000171105;ENSG00000180210
GO:0071417	cellular response to organonitrogen compound	BP	9	1.25E-07	1.08E-05	ENSG00000073756;ENSG00000087245;ENSG00000100823;ENSG00000104267;ENSG00000106546;ENSG00000132170;ENSG00000146648;ENSG00000170890;ENSG00000171105
GO:0072593	reactive oxygen species metabolic process	BP	7	1.73E-07	1.45E-05	ENSG00000005381;ENSG00000007171;ENSG00000073756;ENSG00000146648;ENSG00000149968;ENSG00000171105;ENSG00000180210
GO:0051716	cellular response to stimulus	BP	26	2.19E-07	1.76E-05	ENSG00000005381;ENSG00000007171;ENSG00000073756;ENSG00000087245;ENSG00000091831;ENSG00000095303;ENSG00000100823;ENSG00000100985;ENSG00000104267;ENSG00000105426;ENSG00000105976;ENSG00000106546;ENSG00000128052;ENSG00000132170;ENSG00000133742;ENSG00000143365;ENSG00000146648;ENSG00000148680;ENSG00000149968;ENSG00000163464;ENSG00000170890;ENSG00000171105;ENSG00000178623;ENSG00000180210;ENSG00000186951:ENSG00000198074
GO:0009719	response to endogenous stimulus	BP	13	2.95E-07	2.30E-05	ENSG00000007171;ENSG00000073756;ENSG00000087245;ENSG00000091831;ENSG00000100823;ENSG00000104267;ENSG00000106546;ENSG00000132170;ENSG00000143365;ENSG00000146648;ENSG00000170890;ENSG00000171105;ENSG00000186951
GO:0050896	response to stimulus	BP	28	3.13E-07	2.36E-05	ENSG00000005381;ENSG00000007171;ENSG00000073756;ENSG00000085563;ENSG00000087245;ENSG00000091831;ENSG00000095303;ENSG00000100823;ENSG00000100985;ENSG00000104267;ENSG00000105426;ENSG00000105976;ENSG00000106546;ENSG00000118777;ENSG00000128052;ENSG00000132170;ENSG00000133742;ENSG00000143365;ENSG00000146648;ENSG00000148680;ENSG00000149968;ENSG00000163464;ENSG00000170890;ENSG00000171105;ENSG00000178623:ENSG00000180210:ENSG00000186951:ENSG00000198074
GO:0071495	cellular response to endogenous stimulus	BP	12	3.77E-07	2.73E-05	ENSG00000073756;ENSG00000087245;ENSG00000091831;ENSG00000100823;ENSG00000104267;ENSG00000106546;ENSG00000132170;ENSG00000143365;ENSG00000146648;ENSG00000170890;ENSG00000171105;ENSG00000186951
GO:0034599	cellular response to oxidative stress	BP	7	3.92E-07	2.73E-05	ENSG00000005381;ENSG00000087245;ENSG00000100823;ENSG00000100985;ENSG00000105976;ENSG00000146648;ENSG00000149968
GO:0009891	positive regulation of biosynthetic process	BP	14	3.95E-07	2.73E-05	ENSG00000007171;ENSG00000073756;ENSG00000091831;ENSG00000100823;ENSG00000105976;ENSG00000106546;ENSG00000128052;ENSG00000132170;ENSG00000143365;ENSG00000146648;ENSG00000170890;ENSG00000171105;ENSG00000180210;ENSG00000186951
GO:0019371	cyclooxygenase pathway	BP	3	4.51E-07	3.03E-05	ENSG00000073756;ENSG00000095303;ENSG00000159228
GO:0019221	cytokine-mediated signaling pathway	BP	10	5.98E-07	3.91E-05	ENSG00000007171;ENSG00000073756;ENSG00000087245;ENSG00000100985;ENSG00000132170;ENSG00000133742;ENSG00000143365;ENSG00000149968;ENSG00000163464;ENSG00000178623
GO:0009888	tissue development	BP	14	6.19E-07	3.94E-05	ENSG00000073756;ENSG00000087245;ENSG00000091831;ENSG00000100985;ENSG00000104267;ENSG00000105426;ENSG00000105976;ENSG00000128052;ENSG00000132170;ENSG00000143365;ENSG00000146648;ENSG00000159228;ENSG00000171105;ENSG00000186951
GO:0042127	regulation of cell proliferation	BP	13	6.79E-07	4.14E-05	ENSG00000007171;ENSG00000073756;ENSG00000087245;ENSG00000091831;ENSG00000095303;ENSG00000100985;ENSG00000106546;ENSG00000128052;ENSG00000132170;ENSG00000146648;ENSG00000170890;ENSG00000171105;ENSG00000180210
GO:0032870	cellular response to hormone stimulus	BP	9	7.01E-07	4.14E-05	ENSG00000091831;ENSG00000100823;ENSG00000104267;ENSG00000132170;ENSG00000143365;ENSG00000146648;ENSG00000170890;ENSG00000171105;ENSG00000186951
GO:0006954	inflammatory response	BP	10	7.01E-07	4.14E-05	ENSG00000007171;ENSG00000073756;ENSG00000091831;ENSG00000095303;ENSG00000100985;ENSG00000132170;ENSG00000146648;ENSG00000149968;ENSG00000180210;ENSG00000186951
GO:0032963	collagen metabolic process	BP	5	8.64E-07	4.98E-05	ENSG00000087245;ENSG00000100985;ENSG00000132170;ENSG00000149968;ENSG00000180210

GO:0033993	response to lipid	BP	10	9.64E-07	5.43E-05	ENSG00000005381;ENSG00000007171;ENSG00000073756;ENSG00000091831;ENSG00000104267;ENSG00000106546;ENSG00000132170;ENSG00000143365;ENSG00000146648;ENSG00000186951
GO:0019725	cellular homeostasis	BP	10	1.02E-06	5.59E-05	ENSG00000005381;ENSG00000007171;ENSG00000091831;ENSG00000100823;ENSG00000104267;ENSG00000118777;ENSG00000163464;ENSG00000170890;ENSG00000178623;ENSG00000180210
GO:0046456	icosanoid biosynthetic process	BP	4	1.16E-06	6.26E-05	ENSG00000073756;ENSG00000095303;ENSG00000159228;ENSG00000170890
GO:0071407	cellular response to organic cyclic compound	BP	8	1.26E-06	6.63E-05	ENSG00000073756;ENSG00000091831;ENSG00000100823;ENSG00000106546;ENSG00000132170;ENSG00000143365;ENSG00000146648;ENSG00000186951
GO:0055114	oxidation-reduction process	BP	10	1.61E-06	8.11E-05	ENSG00000005381;ENSG00000007171;ENSG00000073756;ENSG00000095303;ENSG00000100823;ENSG00000132170;ENSG00000159228;ENSG00000171105;ENSG00000186951;ENSG00000198074
GO:0009605	response to external stimulus	BP	15	1.61E-06	8.11E-05	ENSG00000005381;ENSG00000007171;ENSG00000073756;ENSG00000091831;ENSG00000100985;ENSG00000105426;ENSG00000105976;ENSG00000128052;ENSG00000132170;ENSG00000146648;ENSG00000149968;ENSG00000163464;ENSG00000170890;ENSG00000180210;ENSG00000186951
GO:0048545	response to steroid hormone	BP	7	1.79E-06	8.83E-05	ENSG00000073756;ENSG00000091831;ENSG00000104267;ENSG00000132170;ENSG00000143365;ENSG00000146648;ENSG00000186951
GO:0031347	regulation of defense response	BP	10	1.91E-06	9.26E-05	ENSG00000007171;ENSG00000073756;ENSG00000091831;ENSG00000100985;ENSG00000105426;ENSG00000132170;ENSG00000146648;ENSG00000149968;ENSG00000180210;ENSG00000186951
GO:0050801	ion homeostasis	BP	9	1.95E-06	9.26E-05	ENSG00000091831;ENSG00000104267;ENSG00000118777;ENSG00000128052;ENSG00000146648;ENSG00000163464;ENSG00000170890;ENSG00000178623;ENSG00000180210
GO:0062013	positive regulation of small molecule metabolic process	BP	5	2.38E-06	0.00011063	ENSG00000007171;ENSG00000073756;ENSG00000132170;ENSG00000171105;ENSG00000186951
GO:0043434	response to peptide hormone	BP	7	3.04E-06	0.00013888	ENSG00000073756;ENSG00000100823;ENSG00000104267;ENSG00000132170;ENSG00000170890;ENSG00000171105;ENSG00000186951
GO:0017144	drug metabolic process	BP	9	3.16E-06	0.00014163	ENSG00000005381;ENSG00000007171;ENSG00000073756;ENSG00000146648;ENSG00000149968;ENSG00000159228;ENSG00000171105;ENSG00000186951;ENSG00000198074
GO:0032879	regulation of localization	BP	15	4.89E-06	0.00021509	ENSG00000007171;ENSG00000073756;ENSG00000085563;ENSG00000100823;ENSG00000100985;ENSG00000104267;ENSG00000105976;ENSG00000128052;ENSG00000132170;ENSG00000146648;ENSG00000170890;ENSG00000171105;ENSG00000178623;ENSG00000180210;ENSG00000186951
GO:0006629	lipid metabolic process	BP	11	5.10E-06	0.00022042	ENSG00000073756;ENSG00000091831;ENSG00000095303;ENSG00000132170;ENSG00000143365;ENSG00000146648;ENSG00000159228;ENSG00000170890;ENSG00000180210;ENSG00000186951;ENSG00000198074
GO:0015711	organic anion transport	BP	7	5.52E-06	0.00023423	ENSG00000007171;ENSG00000085563;ENSG00000104267;ENSG00000132170;ENSG00000133742;ENSG00000170890;ENSG00000186951
GO:0007166	cell surface receptor signaling pathway	BP	16	5.65E-06	0.0002358	ENSG00000007171;ENSG00000073756;ENSG00000087245;ENSG00000091831;ENSG00000100985;ENSG00000105976;ENSG00000128052;ENSG00000132170;ENSG00000133742;ENSG00000143365;ENSG00000146648;ENSG00000149968;ENSG00000163464;ENSG00000171105;ENSG00000178623;ENSG00000180210
GO:0080134	regulation of response to stress	BP	12	5.77E-06	0.0002364	ENSG00000007171;ENSG00000073756;ENSG00000085563;ENSG00000091831;ENSG00000100985;ENSG00000105426;ENSG00000105976;ENSG00000132170;ENSG00000146648;ENSG00000149968;ENSG00000180210;ENSG00000186951
GO:0048660	regulation of smooth muscle cell proliferation	BP	5	6.42E-06	0.00025335	ENSG00000073756;ENSG00000087245;ENSG00000100985;ENSG00000132170;ENSG00000146648
GO:0044281	small molecule metabolic process	BP	13	6.47E-06	0.00025335	ENSG00000005381;ENSG00000007171;ENSG00000073756;ENSG00000095303;ENSG00000118777;ENSG00000132170;ENSG00000133742;ENSG00000143365;ENSG00000159228;ENSG00000170890;ENSG00000171105;ENSG00000186951;ENSG00000198074
GO:0014070	response to organic cyclic compound	BP	9	6.57E-06	0.00025335	ENSG00000073756;ENSG00000091831;ENSG00000100823;ENSG00000104267;ENSG00000106546;ENSG00000132170;ENSG00000143365;ENSG00000146648;ENSG00000186951
GO:0006952	defense response	BP	13	6.60E-06	0.00025335	ENSG00000005381;ENSG00000007171;ENSG00000073756;ENSG00000091831;ENSG00000095303;ENSG00000100985;ENSG00000105426;ENSG00000132170;ENSG00000146648;ENSG00000149968;ENSG00000170890;ENSG00000180210;ENSG00000186951
GO:0048659	smooth muscle cell proliferation	BP	5	6.77E-06	0.00025583	ENSG00000073756;ENSG00000087245;ENSG00000100985;ENSG00000132170;ENSG00000146648

GO:0006082	organic acid metabolic process	BP	10	7.69E-06	0.00028205	ENSG00000005381;ENSG00000007171;ENSG00000073756;ENSG00000095303;ENSG00000118777;ENSG00000132170;ENSG00000159228;ENSG00000170890;ENSG00000171105;ENSG00000186951
GO:0008217	regulation of blood pressure	BP	5	7.70E-06	0.00028205	ENSG00000007171;ENSG00000073756;ENSG00000095303;ENSG00000132170;ENSG00000186951
GO:0048145	regulation of fibroblast proliferation	BP	4	8.42E-06	0.00030403	ENSG00000091831;ENSG00000132170;ENSG00000146648;ENSG00000170890
GO:0048144	fibroblast proliferation	BP	4	8.80E-06	0.00031302	ENSG00000091831;ENSG00000132170;ENSG00000146648;ENSG00000170890
GO:0071345	cellular response to cytokine stimulus	BP	10	8.94E-06	0.00031348	ENSG00000007171;ENSG00000073756;ENSG00000087245;ENSG00000100985;ENSG00000132170;ENSG00000133742;ENSG00000143365;ENSG00000149968;ENSG00000163464;ENSG00000178623
GO:0055080	cation homeostasis	BP	8	9.24E-06	0.00031914	ENSG00000091831;ENSG00000104267;ENSG00000118777;ENSG00000146648;ENSG00000163464;ENSG00000170890;ENSG00000178623;ENSG00000180210
GO:0072330	monocarboxylic acid biosynthetic process	BP	6	9.41E-06	0.00032055	ENSG00000073756;ENSG00000095303;ENSG00000159228;ENSG00000170890;ENSG00000171105;ENSG00000186951
GO:0007165	signal transduction	BP	22	9.61E-06	0.0003211	ENSG00000007171;ENSG00000073756;ENSG00000087245;ENSG00000091831;ENSG00000100985;ENSG00000104267;ENSG00000105426;ENSG00000105976;ENSG00000106546;ENSG00000128052;ENSG00000132170;ENSG00000133742;ENSG00000143365;ENSG00000146648;ENSG00000148680;ENSG00000149968;ENSG00000163464;ENSG00000170890;ENSG00000171105;ENSG00000178623;ENSG00000180210;ENSG00000186951
GO:0001516	prostaglandin biosynthetic process	BP	3	9.82E-06	0.0003211	ENSG00000073756;ENSG00000095303;ENSG00000159228
GO:0046457	prostanoid biosynthetic process	BP	3	9.82E-06	0.0003211	ENSG00000073756;ENSG00000095303;ENSG00000159228
GO:2000377	regulation of reactive oxygen species metabolic process	BP	5	1.03E-05	0.00033252	ENSG00000073756;ENSG00000146648;ENSG00000149968;ENSG00000171105;ENSG00000180210
GO:0098771	inorganic ion homeostasis	BP	8	1.04E-05	0.00033252	ENSG00000091831;ENSG00000104267;ENSG00000118777;ENSG00000146648;ENSG00000163464;ENSG00000170890;ENSG00000178623;ENSG00000180210
GO:0150076	neuroinflammatory response	BP	4	1.13E-05	0.00035621	ENSG00000073756;ENSG00000100985;ENSG00000146648;ENSG00000149968
GO:0032431	activation of phospholipase A2 activity	BP	2	1.22E-05	0.00037859	ENSG00000146648;ENSG00000170890
GO:0051049	regulation of transport	BP	12	1.24E-05	0.00037873	ENSG00000007171;ENSG00000073756;ENSG00000085563;ENSG00000100985;ENSG00000104267;ENSG00000132170;ENSG00000146648;ENSG00000170890;ENSG00000171105;ENSG00000178623;ENSG00000180210;ENSG00000186951
GO:0008015	blood circulation	BP	7	1.27E-05	0.0003851	ENSG00000007171;ENSG00000073756;ENSG00000095303;ENSG00000132170;ENSG00000146648;ENSG00000148680;ENSG00000186951
GO:0006950	response to stress	BP	18	1.31E-05	0.00039092	ENSG00000005381;ENSG00000007171;ENSG00000073756;ENSG00000085563;ENSG00000087245;ENSG00000091831;ENSG00000095303;ENSG00000100823;ENSG00000100985;ENSG00000104267;ENSG00000105426;ENSG00000105976;ENSG00000132170;ENSG00000146648;ENSG00000149968;ENSG00000170890;ENSG00000180210;ENSG00000186951
GO:0015908	fatty acid transport	BP	4	1.33E-05	0.00039259	ENSG00000007171;ENSG00000132170;ENSG00000170890;ENSG00000186951
GO:0008284	positive regulation of cell proliferation	BP	9	1.37E-05	0.00039822	ENSG00000073756;ENSG00000087245;ENSG00000091831;ENSG00000100985;ENSG00000128052;ENSG00000146648;ENSG00000170890;ENSG00000171105;ENSG00000180210
GO:0003013	circulatory system process	BP	7	1.41E-05	0.0004024	ENSG00000007171;ENSG00000073756;ENSG00000095303;ENSG00000132170;ENSG00000146648;ENSG00000148680;ENSG00000186951
GO:0016477	cell migration	BP	11	1.41E-05	0.0004024	ENSG00000073756;ENSG00000100823;ENSG00000100985;ENSG00000105976;ENSG00000128052;ENSG00000132170;ENSG00000146648;ENSG00000163464;ENSG00000170890;ENSG00000171105;ENSG00000180210
GO:0045923	positive regulation of fatty acid metabolic process	BP	3	1.46E-05	0.00041094	ENSG00000073756;ENSG00000132170;ENSG00000186951

GO:0034762	regulation of transmembrane transport	BP	7	1.54E-05	0.00042904	ENSG00000085563;ENSG00000100985;ENSG00000104267;ENSG00000170890;ENSG00000171105;ENSG00000178623;ENSG00000180210
GO:0031328	positive regulation of cellular biosynthetic process	BP	12	1.64E-05	0.00045103	ENSG00000007171;ENSG00000073756;ENSG00000091831;ENSG00000100823;ENSG00000105976;ENSG00000106546;ENSG00000132170;ENSG00000143365;ENSG00000146648;ENSG00000170890;ENSG00000171105;ENSG00000186951
GO:0034097	response to cytokine	BP	10	1.69E-05	0.00045884	ENSG00000007171;ENSG00000073756;ENSG00000087245;ENSG00000100985;ENSG00000132170;ENSG00000133742;ENSG00000143365;ENSG00000149968;ENSG00000163464;ENSG00000178623
GO:2000379	positive regulation of reactive oxygen species metabolic process	BP	4	1.86E-05	0.00050122	ENSG00000073756;ENSG00000146648;ENSG00000171105;ENSG00000180210
GO:0051173	positive regulation of nitrogen compound metabolic process	BP	15	1.99E-05	0.00052879	ENSG00000007171;ENSG00000073756;ENSG00000091831;ENSG00000100823;ENSG00000100985;ENSG00000105976;ENSG00000106546;ENSG00000128052;ENSG00000132170;ENSG00000143365;ENSG00000146648;ENSG00000170890;ENSG00000171105;ENSG00000180210;ENSG00000186951
GO:0010891	negative regulation of sequestering of triglyceride	BP	2	2.03E-05	0.00053448	ENSG00000132170;ENSG00000186951
GO:1901570	fatty acid derivative biosynthetic process	BP	4	2.07E-05	0.00053825	ENSG00000073756;ENSG00000095303;ENSG00000159228;ENSG00000170890
GO:0006631	fatty acid metabolic process	BP	6	2.09E-05	0.00053825	ENSG00000073756;ENSG00000095303;ENSG00000132170;ENSG00000159228;ENSG00000170890;ENSG00000186951
GO:0006811	ion transport	BP	11	2.21E-05	0.00055988	ENSG00000007171;ENSG00000073756;ENSG00000085563;ENSG00000100985;ENSG00000104267;ENSG00000132170;ENSG00000133742;ENSG00000170890;ENSG00000178623;ENSG00000180210;ENSG00000186951
GO:0048661	positive regulation of smooth muscle cell proliferation	BP	4	2.22E-05	0.00055988	ENSG00000073756;ENSG00000087245;ENSG00000100985;ENSG00000146648
GO:0051186	cofactor metabolic process	BP	7	2.68E-05	0.00066762	ENSG00000005381;ENSG00000073756;ENSG00000146648;ENSG00000149968;ENSG00000171105;ENSG00000186951;ENSG00000198074
GO:0006820	anion transport	BP	7	2.79E-05	0.00068799	ENSG00000007171;ENSG00000085563;ENSG00000104267;ENSG00000132170;ENSG00000133742;ENSG00000170890;ENSG00000186951
GO:0010871	negative regulation of receptor biosynthetic process	BP	2	3.05E-05	0.00073133	ENSG00000132170;ENSG00000186951
GO:0032430	positive regulation of phospholipase A2 activity	BP	2	3.05E-05	0.00073133	ENSG00000146648;ENSG00000170890
GO:0033002	muscle cell proliferation	BP	5	3.08E-05	0.00073133	ENSG00000073756;ENSG00000087245;ENSG00000100985;ENSG00000132170;ENSG00000146648
GO:0006690	icosanoid metabolic process	BP	4	3.08E-05	0.00073133	ENSG00000073756;ENSG00000095303;ENSG00000159228;ENSG00000170890
GO:0031325	positive regulation of cellular metabolic process	BP	15	3.22E-05	0.00075558	ENSG00000007171;ENSG00000073756;ENSG00000091831;ENSG00000100823;ENSG00000100985;ENSG00000105976;ENSG00000106546;ENSG00000128052;ENSG00000132170;ENSG00000143365;ENSG00000146648;ENSG00000170890;ENSG00000171105;ENSG00000180210;ENSG00000186951
GO:0038083	peptidyl-tyrosine autophosphorylation	BP	3	3.27E-05	0.00075802	ENSG00000128052;ENSG00000146648;ENSG00000171105
GO:0023052	signaling	BP	22	3.34E-05	0.00075802	ENSG00000007171;ENSG00000073756;ENSG00000087245;ENSG00000091831;ENSG00000100985;ENSG00000104267;ENSG00000105426;ENSG00000105976;ENSG00000106546;ENSG00000128052;ENSG00000132170;ENSG00000133742;ENSG00000143365;ENSG00000146648;ENSG00000148680;ENSG00000149968;ENSG00000163464;ENSG00000170890;ENSG00000171105;ENSG00000178623;ENSG00000180210;ENSG00000186951
GO:0048870	cell motility	BP	11	3.36E-05	0.00075802	ENSG00000073756;ENSG00000100823;ENSG00000100985;ENSG00000105976;ENSG00000128052;ENSG00000132170;ENSG00000146648;ENSG00000163464;ENSG00000170890;ENSG00000171105;ENSG00000180210
GO:0051674	localization of cell	BP	11	3.36E-05	0.00075802	ENSG00000073756;ENSG00000100823;ENSG00000100985;ENSG00000105976;ENSG00000128052;ENSG00000132170;ENSG00000146648;ENSG00000163464;ENSG00000170890;ENSG00000171105;ENSG00000180210
GO:1990748	cellular detoxification	BP	4	3.38E-05	0.00075802	ENSG00000005381;ENSG00000073756;ENSG00000095303;ENSG00000198074

GO:0048518	positive regulation of biological process	BP	21	3.44E-05	0.00076334	ENSG00000007171;ENSG00000073756;ENSG00000085563;ENSG00000087245;ENSG00000091831;ENSG00000100823;ENSG00000100985;ENSG00000104267;ENSG00000105426;ENSG00000105976;ENSG00000106546;ENSG00000128052;ENSG00000132170;ENSG00000143365;ENSG00000146648;ENSG00000149968;ENSG00000170890;ENSG00000171105;ENSG00000178623;ENSG00000180210;ENSG00000186951
GO:1903409	reactive oxygen species biosynthetic process	BP	4	3.49E-05	0.00076444	ENSG00000005381;ENSG00000007171;ENSG00000073756;ENSG00000171105
GO:0007154	cell communication	BP	22	3.51E-05	0.00076444	ENSG00000007171;ENSG00000073756;ENSG00000087245;ENSG00000091831;ENSG00000100985;ENSG00000104267;ENSG00000105426;ENSG00000105976;ENSG00000106546;ENSG00000128052;ENSG00000132170;ENSG00000133742;ENSG00000143365;ENSG00000146648;ENSG00000148680;ENSG00000149968;ENSG00000163464;ENSG00000170890;ENSG00000171105;ENSG00000178623;ENSG00000180210;ENSG00000186951
GO:0032787	monocarboxylic acid metabolic process	BP	7	3.85E-05	0.00083085	ENSG00000073756;ENSG00000095303;ENSG00000132170;ENSG00000159228;ENSG00000170890;ENSG00000171105;ENSG00000186951
GO:0071383	cellular response to steroid hormone stimulus	BP	5	3.97E-05	0.00085047	ENSG00000091831;ENSG00000132170;ENSG00000143365;ENSG00000146648;ENSG00000186951
GO:0010941	regulation of cell death	BP	11	4.05E-05	0.00086009	ENSG00000005381;ENSG00000073756;ENSG00000091831;ENSG00000100823;ENSG00000100985;ENSG00000105976;ENSG00000128052;ENSG00000132170;ENSG00000146648;ENSG00000149968;ENSG00000186951
GO:0055065	metal ion homeostasis	BP	7	4.11E-05	0.00086446	ENSG00000091831;ENSG00000118777;ENSG00000146648;ENSG00000163464;ENSG00000170890;ENSG00000178623;ENSG00000180210
GO:0071396	cellular response to lipid	BP	7	4.19E-05	0.00087322	ENSG00000007171;ENSG00000091831;ENSG00000106546;ENSG00000132170;ENSG00000143365;ENSG00000146648;ENSG00000186951
GO:0030003	cellular cation homeostasis	BP	7	4.39E-05	0.00090705	ENSG00000091831;ENSG00000104267;ENSG00000118777;ENSG00000163464;ENSG00000170890;ENSG00000178623;ENSG00000180210
GO:0014066	regulation of phosphatidylinositol 3-kinase signaling	BP	4	4.67E-05	0.00095784	ENSG00000128052;ENSG00000146648;ENSG00000171105;ENSG00000180210
GO:0006692	prostanoid metabolic process	BP	3	4.86E-05	0.00097909	ENSG00000073756;ENSG00000095303;ENSG00000159228
GO:0006693	prostaglandin metabolic process	BP	3	4.86E-05	0.00097909	ENSG00000073756;ENSG00000095303;ENSG00000159228
GO:0006873	cellular ion homeostasis	BP	7	4.99E-05	0.0009973	ENSG00000091831;ENSG00000104267;ENSG00000118777;ENSG00000163464;ENSG00000170890;ENSG00000178623;ENSG00000180210
GO:0046394	carboxylic acid biosynthetic process	BP	6	5.07E-05	0.00099808	ENSG00000073756;ENSG00000095303;ENSG00000159228;ENSG00000170890;ENSG00000171105;ENSG00000186951
GO:0043436	oxoacid metabolic process	BP	9	5.07E-05	0.00099808	ENSG00000005381;ENSG00000007171;ENSG00000073756;ENSG00000095303;ENSG00000132170;ENSG00000159228;ENSG00000170890;ENSG00000171105;ENSG00000186951
GO:0016053	organic acid biosynthetic process	BP	6	5.13E-05	0.00100023	ENSG00000073756;ENSG00000095303;ENSG00000159228;ENSG00000170890;ENSG00000171105;ENSG00000186951
GO:0062012	regulation of small molecule metabolic process	BP	6	5.19E-05	0.00100395	ENSG00000007171;ENSG00000073756;ENSG00000132170;ENSG00000143365;ENSG00000171105;ENSG00000186951
GO:0043491	protein kinase B signaling	BP	5	5.58E-05	0.00107131	ENSG00000091831;ENSG00000105976;ENSG00000128052;ENSG00000146648;ENSG00000171105
GO:0098754	detoxification	BP	4	5.66E-05	0.00107273	ENSG00000005381;ENSG00000073756;ENSG00000095303;ENSG00000198074
GO:0010887	negative regulation of cholesterol storage	BP	2	5.68E-05	0.00107273	ENSG00000132170;ENSG00000186951
GO:0010647	positive regulation of cell communication	BP	11	5.77E-05	0.00108093	ENSG00000073756;ENSG00000091831;ENSG00000100985;ENSG00000104267;ENSG00000105976;ENSG00000128052;ENSG00000146648;ENSG00000170890;ENSG00000171105;ENSG00000178623;ENSG00000180210
GO:0030574	collagen catabolic process	BP	3	5.81E-05	0.00108093	ENSG00000087245;ENSG00000100985;ENSG00000149968

GO:0010557	positive regulation of macromolecule biosynthetic process	BP	11	5.88E-05	0.0010853	ENSG00000091831;ENSG00000100823;ENSG00000105976;ENSG00000106546;ENSG00000128052;ENSG00000132170;ENSG00000143365;ENSG00000146648;ENSG00000170890;ENSG00000171105;ENSG00000186951
GO:0023056	positive regulation of signaling	BP	11	5.99E-05	0.00109727	ENSG00000073756;ENSG00000091831;ENSG00000100985;ENSG00000104267;ENSG00000105976;ENSG00000128052;ENSG00000146648;ENSG00000170890;ENSG00000171105;ENSG00000178623;ENSG00000180210
GO:0010632	regulation of epithelial cell migration	BP	5	6.05E-05	0.00110059	ENSG00000073756;ENSG00000100985;ENSG00000105976;ENSG00000128052;ENSG00000132170
GO:0045834	positive regulation of lipid metabolic process	BP	4	6.13E-05	0.00110073	ENSG00000073756;ENSG00000132170;ENSG00000180210;ENSG00000186951
GO:0030334	regulation of cell migration	BP	8	6.14E-05	0.00110073	ENSG00000073756;ENSG00000100823;ENSG00000100985;ENSG00000105976;ENSG00000128052;ENSG00000132170;ENSG00000146648;ENSG00000171105
GO:0045935	positive regulation of nucleobase-containing compound metabolic process	BP	11	6.33E-05	0.00112574	ENSG00000007171;ENSG00000091831;ENSG00000100823;ENSG00000105976;ENSG00000106546;ENSG00000132170;ENSG00000143365;ENSG00000146648;ENSG00000170890;ENSG00000171105;ENSG00000186951
GO:0007566	embryo implantation	BP	3	6.51E-05	0.00113242	ENSG00000073756;ENSG00000087245;ENSG00000100985
GO:0019369	arachidonic acid metabolic process	BP	3	6.51E-05	0.00113242	ENSG00000073756;ENSG00000095303;ENSG00000159228
GO:0048146	positive regulation of fibroblast proliferation	BP	3	6.51E-05	0.00113242	ENSG00000091831;ENSG00000146648;ENSG00000170890
GO:0014074	response to purine-containing compound	BP	4	6.62E-05	0.00113596	ENSG00000073756;ENSG00000100823;ENSG00000106546;ENSG00000132170
GO:0040008	regulation of growth	BP	7	6.62E-05	0.00113596	ENSG00000005381;ENSG00000105426;ENSG00000132170;ENSG00000146648;ENSG00000171105;ENSG00000180210;ENSG00000186951
GO:0016999	antibiotic metabolic process	BP	4	6.79E-05	0.00115258	ENSG00000005381;ENSG00000146648;ENSG00000149968;ENSG00000198074
GO:0009056	catabolic process	BP	13	6.81E-05	0.00115258	ENSG00000005381;ENSG00000007171;ENSG00000087245;ENSG00000100823;ENSG00000100985;ENSG00000105976;ENSG00000128052;ENSG00000146648;ENSG00000149968;ENSG00000170890;ENSG00000171105;ENSG00000186951;ENSG00000198074
GO:0009410	response to xenobiotic stimulus	BP	5	6.87E-05	0.00115375	ENSG00000095303;ENSG00000106546;ENSG00000132170;ENSG00000143365;ENSG00000146648
GO:0043269	regulation of ion transport	BP	7	6.97E-05	0.00116306	ENSG00000073756;ENSG00000085563;ENSG00000100985;ENSG00000104267;ENSG00000170890;ENSG00000178623;ENSG00000180210
GO:0009628	response to abiotic stimulus	BP	9	7.20E-05	0.00119273	ENSG00000005381;ENSG00000007171;ENSG00000073756;ENSG00000085563;ENSG00000087245;ENSG00000104267;ENSG00000132170;ENSG00000146648;ENSG00000186951
GO:0006636	unsaturated fatty acid biosynthetic process	BP	3	7.65E-05	0.00124256	ENSG00000073756;ENSG00000095303;ENSG00000159228
GO:0042743	hydrogen peroxide metabolic process	BP	3	7.65E-05	0.00124256	ENSG00000005381;ENSG00000146648;ENSG00000149968
GO:1904645	response to amyloid-beta	BP	3	7.65E-05	0.00124256	ENSG00000087245;ENSG00000100985;ENSG00000149968
GO:0030522	intracellular receptor signaling pathway	BP	5	8.13E-05	0.00131074	ENSG00000091831;ENSG00000106546;ENSG00000132170;ENSG00000143365;ENSG00000186951
GO:0048522	positive regulation of cellular process	BP	19	8.32E-05	0.0013292	ENSG00000007171;ENSG00000073756;ENSG00000087245;ENSG00000091831;ENSG00000100823;ENSG00000100985;ENSG00000104267;ENSG00000105976;ENSG00000106546;ENSG00000128052;ENSG00000132170;ENSG00000143365;ENSG00000146648;ENSG00000149968;ENSG00000170890;ENSG00000171105;ENSG00000178623;ENSG00000180210;ENSG00000186951
GO:0009653	anatomical structure morphogenesis	BP	13	8.35E-05	0.0013292	ENSG00000073756;ENSG00000087245;ENSG00000091831;ENSG00000100985;ENSG00000104267;ENSG00000105426;ENSG00000105976;ENSG00000128052;ENSG00000132170;ENSG00000146648;ENSG00000171105;ENSG00000180210;ENSG00000186951

GO:1903508	positive regulation of nucleic acid-templated transcription	BP	10	8.69E-05	0.00137253	ENSG00000091831;ENSG00000100823;ENSG00000105976;ENSG00000106546;ENSG00000132170;ENSG00000143365;ENSG00000146648;ENSG00000170890;ENSG00000171105;ENSG00000186951
GO:1902680	positive regulation of RNA biosynthetic process	BP	10	8.74E-05	0.00137253	ENSG00000091831;ENSG00000100823;ENSG00000105976;ENSG00000106546;ENSG00000132170;ENSG00000143365;ENSG00000146648;ENSG00000170890;ENSG00000171105;ENSG00000186951
GO:0010518	positive regulation of phospholipase activity	BP	3	8.92E-05	0.00139285	ENSG00000091831;ENSG00000146648;ENSG00000170890
GO:0014065	phosphatidylinositol 3-kinase signaling	BP	4	9.10E-05	0.00140304	ENSG00000128052;ENSG00000146648;ENSG00000171105;ENSG00000180210
GO:2001225	regulation of chloride transport	BP	2	9.11E-05	0.00140304	ENSG00000085563;ENSG00000104267
GO:0008219	cell death	BP	12	9.25E-05	0.00141598	ENSG00000005381;ENSG00000073756;ENSG00000091831;ENSG00000100823;ENSG00000100985;ENSG00000105976;ENSG00000106546;ENSG00000128052;ENSG00000132170;ENSG00000146648;ENSG00000149968;ENSG00000186951
GO:0040007	growth	BP	8	9.35E-05	0.00142179	ENSG00000005381;ENSG00000091831;ENSG00000105426;ENSG00000132170;ENSG00000146648;ENSG00000171105;ENSG00000180210;ENSG00000186951
GO:2000145	regulation of cell motility	BP	8	9.47E-05	0.00142293	ENSG00000073756;ENSG00000100823;ENSG00000100985;ENSG00000105976;ENSG00000128052;ENSG00000132170;ENSG00000146648;ENSG00000171105
GO:0009893	positive regulation of metabolic process	BP	15	9.47E-05	0.00142293	ENSG00000007171;ENSG00000073756;ENSG00000091831;ENSG00000100823;ENSG00000100985;ENSG00000105976;ENSG00000106546;ENSG00000128052;ENSG00000132170;ENSG00000143365;ENSG00000146648;ENSG00000170890;ENSG00000171105;ENSG00000180210;ENSG00000186951
GO:0007204	positive regulation of cytosolic calcium ion concentration	BP	5	9.84E-05	0.00146098	ENSG00000091831;ENSG00000163464;ENSG00000170890;ENSG00000178623;ENSG00000180210
GO:0032922	circadian regulation of gene expression	BP	3	9.84E-05	0.00146098	ENSG00000106546;ENSG00000143365;ENSG00000186951
GO:0072507	divalent inorganic cation homeostasis	BP	6	0.0001	0.00147868	ENSG00000091831;ENSG00000146648;ENSG00000163464;ENSG00000170890;ENSG00000178623;ENSG00000180210
GO:0010634	positive regulation of epithelial cell migration	BP	4	0.000104	0.00153195	ENSG00000073756;ENSG00000100985;ENSG00000105976;ENSG00000128052
GO:0006633	fatty acid biosynthetic process	BP	4	0.000107	0.00154673	ENSG00000073756;ENSG00000095303;ENSG00000159228;ENSG00000170890
GO:0015718	monocarboxylic acid transport	BP	4	0.000107	0.00154673	ENSG00000007171;ENSG00000132170;ENSG00000170890;ENSG00000186951
GO:0040011	locomotion	BP	11	0.000107	0.00154673	ENSG00000073756;ENSG00000100823;ENSG00000100985;ENSG00000105976;ENSG00000128052;ENSG00000132170;ENSG00000146648;ENSG00000163464;ENSG00000170890;ENSG00000171105;ENSG00000180210
GO:0150077	regulation of neuroinflammatory response	BP	3	0.000108	0.0015493	ENSG00000073756;ENSG00000100985;ENSG00000149968
GO:0051050	positive regulation of transport	BP	8	0.00011	0.00156669	ENSG00000073756;ENSG00000085563;ENSG00000104267;ENSG00000132170;ENSG00000146648;ENSG00000170890;ENSG00000171105;ENSG00000180210
GO:0060429	epithelium development	BP	9	0.000111	0.00157189	ENSG00000091831;ENSG00000100985;ENSG00000104267;ENSG00000105426;ENSG00000105976;ENSG00000128052;ENSG00000132170;ENSG00000146648;ENSG00000159228
GO:0071375	cellular response to peptide hormone stimulus	BP	5	0.000112	0.00157189	ENSG00000100823;ENSG00000104267;ENSG00000132170;ENSG00000170890;ENSG00000171105
GO:0009892	negative regulation of metabolic process	BP	14	0.00012	0.00167147	ENSG00000007171;ENSG00000073756;ENSG00000091831;ENSG00000100823;ENSG00000100985;ENSG00000105976;ENSG00000106546;ENSG00000128052;ENSG00000132170;ENSG00000143365;ENSG00000146648;ENSG00000149968;ENSG00000180210;ENSG00000186951
GO:0032429	regulation of phospholipase A2 activity	BP	2	0.000133	0.0018534	ENSG00000146648;ENSG00000170890

GO:0030335	positive regulation of cell migration	BP	6	0.000138	0.001906	ENSG00000073756;ENSG00000100985;ENSG00000105976;ENSG00000128052;ENSG00000146648;ENSG00000171105
GO:0051254	positive regulation of RNA metabolic process	BP	10	0.00014	0.0019225	ENSG00000091831;ENSG00000100823;ENSG00000105976;ENSG00000106546;ENSG00000132170;ENSG00000143365;ENSG00000146648;ENSG00000170890;ENSG00000171105;ENSG00000186951
GO:0051897	positive regulation of protein kinase B signaling	BP	4	0.000145	0.00197487	ENSG00000091831;ENSG00000105976;ENSG00000146648;ENSG00000171105
GO:0044283	small molecule biosynthetic process	BP	7	0.000146	0.00197756	ENSG00000005381;ENSG00000073756;ENSG00000095303;ENSG00000159228;ENSG00000170890;ENSG00000171105;ENSG00000186951
GO:0010517	regulation of phospholipase activity	BP	3	0.000147	0.00199295	ENSG00000091831;ENSG00000146648;ENSG00000170890
GO:0010631	epithelial cell migration	BP	5	0.00015	0.0020123	ENSG00000073756;ENSG00000100985;ENSG00000105976;ENSG00000128052;ENSG00000132170
GO:0071466	cellular response to xenobiotic stimulus	BP	4	0.000151	0.0020123	ENSG00000095303;ENSG00000106546;ENSG00000143365;ENSG00000146648
GO:0090132	epithelium migration	BP	5	0.000156	0.00205829	ENSG00000073756;ENSG00000100985;ENSG00000105976;ENSG00000128052;ENSG00000132170
GO:0010745	negative regulation of macrophage derived foam cell differentiation	BP	2	0.000157	0.00205829	ENSG00000132170;ENSG00000186951
GO:0010889	regulation of sequestering of triglyceride	BP	2	0.000157	0.00205829	ENSG00000132170;ENSG00000186951
GO:0047484	regulation of response to osmotic stress	BP	2	0.000157	0.00205829	ENSG00000073756;ENSG00000085563
GO:0001101	response to acid chemical	BP	5	0.000158	0.00205991	ENSG00000073756;ENSG00000087245;ENSG00000128052;ENSG00000132170;ENSG00000146648
GO:0060193	positive regulation of lipase activity	BP	3	0.00016	0.00206157	ENSG00000091831;ENSG00000146648;ENSG00000170890
GO:0051239	regulation of multicellular organismal process	BP	14	0.00016	0.00206157	ENSG00000007171;ENSG00000073756;ENSG00000091831;ENSG00000100985;ENSG00000104267;ENSG00000105426;ENSG00000105976;ENSG00000128052;ENSG00000132170;ENSG00000146648;ENSG00000171105;ENSG00000178623;ENSG00000180210;ENSG00000186951
GO:0051270	regulation of cellular component movement	BP	8	0.000162	0.0020777	ENSG00000073756;ENSG00000100823;ENSG00000100985;ENSG00000105976;ENSG00000128052;ENSG00000132170;ENSG00000146648;ENSG00000171105
GO:0030855	epithelial cell differentiation	BP	7	0.000164	0.00208573	ENSG00000091831;ENSG00000100985;ENSG00000105426;ENSG00000105976;ENSG00000128052;ENSG00000132170;ENSG00000159228
GO:1901568	fatty acid derivative metabolic process	BP	4	0.000167	0.00210041	ENSG00000073756;ENSG00000095303;ENSG00000159228;ENSG00000170890
GO:0051480	regulation of cytosolic calcium ion concentration	BP	5	0.000167	0.00210041	ENSG00000091831;ENSG00000163464;ENSG00000170890;ENSG00000178623;ENSG00000180210
GO:0090130	tissue migration	BP	5	0.000169	0.00211626	ENSG00000073756;ENSG00000100985;ENSG00000105976;ENSG00000128052;ENSG00000132170
GO:0010604	positive regulation of macromolecule metabolic process	BP	14	0.000171	0.00212509	ENSG00000073756;ENSG00000091831;ENSG00000100823;ENSG00000100985;ENSG00000105976;ENSG00000106546;ENSG00000128052;ENSG00000132170;ENSG00000143365;ENSG00000146648;ENSG00000170890;ENSG00000171105;ENSG00000180210;ENSG00000186951
GO:2000147	positive regulation of cell motility	BP	6	0.000171	0.00212509	ENSG00000073756;ENSG00000100985;ENSG00000105976;ENSG00000128052;ENSG00000146648;ENSG00000171105
GO:0040012	regulation of locomotion	BP	8	0.000172	0.00212805	ENSG00000073756;ENSG00000100823;ENSG00000100985;ENSG00000105976;ENSG00000128052;ENSG00000132170;ENSG00000146648;ENSG00000171105

GO:0055082	cellular chemical homeostasis	BP	7	0.00018	0.00221078	ENSG00000091831;ENSG00000104267;ENSG00000118777;ENSG00000163464;ENSG00000170890;ENSG00000178623;ENSG00000180210
GO:0048015	phosphatidylinositol-mediated signaling	BP	4	0.000187	0.00228093	ENSG00000128052;ENSG00000146648;ENSG00000171105;ENSG00000180210
GO:0043627	response to estrogen	BP	3	0.000188	0.00228093	ENSG00000091831;ENSG00000104267;ENSG00000132170
GO:0032103	positive regulation of response to external stimulus	BP	5	0.000189	0.00228246	ENSG00000073756;ENSG00000105976;ENSG00000128052;ENSG00000146648;ENSG00000180210
GO:1902533	positive regulation of intracellular signal transduction	BP	8	0.00019	0.00228246	ENSG00000091831;ENSG00000105976;ENSG00000128052;ENSG00000146648;ENSG00000170890;ENSG00000171105;ENSG00000178623;ENSG00000180210
GO:0043401	steroid hormone mediated signaling pathway	BP	4	0.000191	0.00228626	ENSG00000091831;ENSG00000132170;ENSG00000143365;ENSG00000186951
GO:0006875	cellular metal ion homeostasis	BP	6	0.000195	0.00231177	ENSG00000091831;ENSG00000118777;ENSG00000163464;ENSG00000170890;ENSG00000178623;ENSG00000180210
GO:0045454	cell redox homeostasis	BP	3	0.000195	0.00231177	ENSG00000005381;ENSG00000007171;ENSG00000100823
GO:0033554	cellular response to stress	BP	11	0.000197	0.00231767	ENSG00000005381;ENSG00000073756;ENSG00000087245;ENSG00000100823;ENSG00000100985;ENSG00000104267;ENSG00000105426;ENSG00000105976;ENSG00000132170;ENSG00000146648;ENSG00000149968
GO:0051272	positive regulation of cellular component movement	BP	6	0.000198	0.00231767	ENSG00000073756;ENSG00000100985;ENSG00000105976;ENSG00000128052;ENSG00000146648;ENSG00000171105
GO:0048017	inositol lipid-mediated signaling	BP	4	0.000198	0.00231767	ENSG00000128052;ENSG00000146648;ENSG00000171105;ENSG00000180210
GO:0019752	carboxylic acid metabolic process	BP	8	0.0002	0.00232104	ENSG00000007171;ENSG00000073756;ENSG00000095303;ENSG00000132170;ENSG00000159228;ENSG00000170890;ENSG00000171105;ENSG00000186951
GO:2000026	regulation of multicellular organismal development	BP	11	0.000201	0.00232104	ENSG00000073756;ENSG00000091831;ENSG00000100985;ENSG00000104267;ENSG00000105426;ENSG00000128052;ENSG00000132170;ENSG00000146648;ENSG00000171105;ENSG00000180210;ENSG00000186951
GO:0006801	superoxide metabolic process	BP	3	0.000202	0.00232104	ENSG00000005381;ENSG00000007171;ENSG00000146648
GO:0042310	vasoconstriction	BP	3	0.000202	0.00232104	ENSG00000073756;ENSG00000146648;ENSG00000148680
GO:0044255	cellular lipid metabolic process	BP	8	0.000208	0.00236884	ENSG00000073756;ENSG00000095303;ENSG00000132170;ENSG00000146648;ENSG00000159228;ENSG00000170890;ENSG00000186951;ENSG00000198074
GO:0034764	positive regulation of transmembrane transport	BP	4	0.00021	0.00236884	ENSG00000085563;ENSG00000104267;ENSG00000171105;ENSG00000180210
GO:0010885	regulation of cholesterol storage	BP	2	0.000212	0.00236884	ENSG00000132170;ENSG00000186951
GO:0046321	positive regulation of fatty acid oxidation	BP	2	0.000212	0.00236884	ENSG00000132170;ENSG00000186951
GO:2001028	positive regulation of endothelial cell chemotaxis	BP	2	0.000212	0.00236884	ENSG00000105976;ENSG00000128052
GO:0006869	lipid transport	BP	5	0.000216	0.00240856	ENSG00000007171;ENSG00000085563;ENSG00000132170;ENSG00000170890;ENSG00000186951
GO:0007565	female pregnancy	BP	4	0.000222	0.00246138	ENSG00000073756;ENSG00000087245;ENSG00000091831;ENSG00000100985

GO:0040017	positive regulation of locomotion	BP	6	0.000228	0.00252048	ENSG00000073756;ENSG00000100985;ENSG00000105976;ENSG00000128052;ENSG00000146648;ENSG00000171105
GO:0072359	circulatory system development	BP	8	0.000234	0.0025695	ENSG00000073756;ENSG00000087245;ENSG00000106546;ENSG00000128052;ENSG00000132170;ENSG00000146648;ENSG00000171105;ENSG00000186951
GO:0044093	positive regulation of molecular function	BP	10	0.000236	0.00258164	ENSG00000007171;ENSG00000085563;ENSG00000091831;ENSG00000100985;ENSG00000105976;ENSG00000132170;ENSG00000146648;ENSG00000170890;ENSG00000171105;ENSG00000180210
GO:0035556	intracellular signal transduction	BP	13	0.000239	0.00260666	ENSG00000007171;ENSG00000073756;ENSG00000091831;ENSG00000100985;ENSG00000105976;ENSG00000106546;ENSG00000128052;ENSG00000146648;ENSG00000163464;ENSG00000170890;ENSG00000171105;ENSG00000178623;ENSG00000180210
GO:0010562	positive regulation of phosphorus metabolic process	BP	8	0.000245	0.00264082	ENSG00000007171;ENSG00000073756;ENSG00000100985;ENSG00000128052;ENSG00000146648;ENSG00000170890;ENSG00000171105;ENSG00000180210
GO:0045937	positive regulation of phosphate metabolic process	BP	8	0.000245	0.00264082	ENSG00000007171;ENSG00000073756;ENSG00000100985;ENSG00000128052;ENSG00000146648;ENSG00000170890;ENSG00000171105;ENSG00000180210
GO:0006809	nitric oxide biosynthetic process	BP	3	0.000251	0.00267893	ENSG00000007171;ENSG00000073756;ENSG00000171105
GO:1904705	regulation of vascular smooth muscle cell proliferation	BP	3	0.000251	0.00267893	ENSG00000087245;ENSG00000100985;ENSG00000132170
GO:1990874	vascular smooth muscle cell proliferation	BP	3	0.000251	0.00267893	ENSG00000087245;ENSG00000100985;ENSG00000132170
GO:0006928	movement of cell or subcellular component	BP	11	0.000264	0.00280078	ENSG00000073756;ENSG00000100823;ENSG00000100985;ENSG00000105976;ENSG00000128052;ENSG00000132170;ENSG00000146648;ENSG00000163464;ENSG00000170890;ENSG00000171105;ENSG00000180210
GO:0009612	response to mechanical stimulus	BP	4	0.00027	0.00285213	ENSG00000005381;ENSG00000073756;ENSG00000132170;ENSG00000146648
GO:1901653	cellular response to peptide	BP	5	0.000273	0.00286381	ENSG00000100823;ENSG00000104267;ENSG00000132170;ENSG00000170890;ENSG00000171105
GO:0010878	cholesterol storage	BP	2	0.000273	0.00286381	ENSG00000132170;ENSG00000186951
GO:0071229	cellular response to acid chemical	BP	4	0.000275	0.00286451	ENSG00000087245;ENSG00000128052;ENSG00000132170;ENSG00000146648
GO:0097756	negative regulation of blood vessel diameter	BP	3	0.000278	0.00289124	ENSG00000073756;ENSG00000146648;ENSG00000148680
GO:0007186	G protein-coupled receptor signaling pathway	BP	9	0.000281	0.00290399	ENSG00000091831;ENSG00000104267;ENSG00000105976;ENSG00000132170;ENSG00000148680;ENSG00000163464;ENSG00000171105;ENSG00000178623;ENSG00000180210
GO:0046209	nitric oxide metabolic process	BP	3	0.000288	0.00296374	ENSG00000007171;ENSG00000073756;ENSG00000171105
GO:0006367	transcription initiation from RNA polymerase II promoter	BP	4	0.000294	0.00301187	ENSG00000091831;ENSG00000132170;ENSG00000143365;ENSG00000186951
GO:0019216	regulation of lipid metabolic process	BP	5	0.000295	0.00301187	ENSG00000073756;ENSG00000132170;ENSG00000143365;ENSG00000180210;ENSG00000186951
GO:0060850	regulation of transcription involved in cell fate commitment	BP	2	0.000307	0.00311132	ENSG00000132170;ENSG00000143365
GO:0006970	response to osmotic stress	BP	3	0.000307	0.00311132	ENSG00000073756;ENSG00000085563;ENSG00000146648
GO:0045893	positive regulation of transcription, DNA-templated	BP	9	0.000315	0.0031699	ENSG00000091831;ENSG00000105976;ENSG00000106546;ENSG00000132170;ENSG00000143365;ENSG00000146648;ENSG00000170890;ENSG00000171105;ENSG00000186951

GO:0022617	extracellular matrix disassembly	BP	3	0.000317	0.00317321	ENSG00000087245;ENSG00000100985;ENSG00000149968
GO:2001057	reactive nitrogen species metabolic process	BP	3	0.000317	0.00317321	ENSG00000007171;ENSG00000073756;ENSG00000171105
GO:0070482	response to oxygen levels	BP	5	0.000319	0.00317321	ENSG00000007171;ENSG00000073756;ENSG00000087245;ENSG00000132170;ENSG00000186951
GO:0019217	regulation of fatty acid metabolic process	BP	3	0.000328	0.00324896	ENSG00000073756;ENSG00000132170;ENSG00000186951
GO:0071241	cellular response to inorganic substance	BP	4	0.000331	0.00326521	ENSG00000073756;ENSG00000100985;ENSG00000146648;ENSG00000149968
GO:0010594	regulation of endothelial cell migration	BP	4	0.000336	0.00330568	ENSG00000073756;ENSG00000105976;ENSG00000128052;ENSG00000132170
GO:1903798	regulation of production of miRNAs involved in gene silencing by miRNA	BP	2	0.000343	0.00336151	ENSG00000091831;ENSG00000146648
GO:0014068	positive regulation of phosphatidylinositol 3-kinase signaling	BP	3	0.000349	0.00340297	ENSG00000128052;ENSG00000171105;ENSG00000180210
GO:0010876	lipid localization	BP	5	0.000351	0.00341283	ENSG00000007171;ENSG00000085563;ENSG00000132170;ENSG00000170890;ENSG00000186951
GO:0065007	biological regulation	BP	28	0.000356	0.00344817	ENSG00000005381;ENSG00000007171;ENSG00000073756;ENSG00000085563;ENSG00000087245;ENSG00000091831;ENSG00000095303;ENSG00000100823;ENSG00000100985;ENSG00000104267;ENSG00000105426;ENSG00000105976;ENSG00000106546;ENSG00000118777;ENSG00000128052;ENSG00000132170;ENSG00000133742;ENSG00000143365;ENSG00000146648;ENSG00000148680;ENSG00000149968;ENSG00000163464;ENSG00000170890;ENSG00000171105;ENSG00000178623;ENSG00000180210;ENSG00000186951;ENSG00000198074
GO:0048584	positive regulation of response to stimulus	BP	12	0.000362	0.00349156	ENSG00000073756;ENSG00000091831;ENSG00000100985;ENSG00000105426;ENSG00000105976;ENSG00000128052;ENSG00000146648;ENSG00000149968;ENSG00000170890;ENSG00000171105;ENSG00000178623;ENSG00000180210
GO:0035239	tube morphogenesis	BP	7	0.00037	0.00354629	ENSG00000073756;ENSG00000087245;ENSG00000091831;ENSG00000105976;ENSG00000128052;ENSG00000132170;ENSG00000146648
GO:0033273	response to vitamin	BP	3	0.000371	0.00354629	ENSG00000073756;ENSG00000132170;ENSG00000146648
GO:0045595	regulation of cell differentiation	BP	10	0.000374	0.00355999	ENSG00000073756;ENSG00000100985;ENSG00000104267;ENSG00000105426;ENSG00000128052;ENSG00000132170;ENSG00000143365;ENSG00000146648;ENSG00000180210;ENSG00000186951
GO:0045780	positive regulation of bone resorption	BP	2	0.000381	0.00357048	ENSG00000104267;ENSG00000146648
GO:0046852	positive regulation of bone remodeling	BP	2	0.000381	0.00357048	ENSG00000104267;ENSG00000146648
GO:0060965	negative regulation of gene silencing by miRNA	BP	2	0.000381	0.00357048	ENSG00000091831;ENSG00000132170
GO:0070920	regulation of production of small RNA involved in gene silencing by RNA	BP	2	0.000381	0.00357048	ENSG00000091831;ENSG00000146648
GO:0001558	regulation of cell growth	BP	5	0.000382	0.00357048	ENSG00000105426;ENSG00000132170;ENSG00000146648;ENSG00000180210;ENSG00000186951
GO:0044706	multi-multicellular organism process	BP	4	0.000388	0.0036128	ENSG00000073756;ENSG00000087245;ENSG00000091831;ENSG00000100985
GO:0060191	regulation of lipase activity	BP	3	0.000394	0.00364984	ENSG00000091831;ENSG00000146648;ENSG00000170890
GO:0007200	phospholipase C-activating G protein-coupled receptor signaling pathway	BP	3	0.000418	0.0038264	ENSG00000091831;ENSG00000178623;ENSG00000180210

GO:0050793	regulation of developmental process	BP	12	0.000418	0.0038264	ENSG00000073756;ENSG00000091831;ENSG00000100985;ENSG00000104267;ENSG00000105426;ENSG00000128052;ENSG00000132170;ENSG00000143365;ENSG00000146648;ENSG00000171105;ENSG00000180210;ENSG00000186951
GO:0048608	reproductive structure development	BP	5	0.00042	0.0038264	ENSG00000073756;ENSG00000091831;ENSG00000132170;ENSG00000146648;ENSG00000171105
GO:0010869	regulation of receptor biosynthetic process	BP	2	0.000421	0.0038264	ENSG00000132170;ENSG00000186951
GO:0071498	cellular response to fluid shear stress	BP	2	0.000421	0.0038264	ENSG00000073756;ENSG00000104267
GO:1901655	cellular response to ketone	BP	3	0.00043	0.00389422	ENSG00000106546;ENSG00000132170;ENSG00000146648
GO:0061458	reproductive system development	BP	5	0.000437	0.00394456	ENSG00000073756;ENSG00000091831;ENSG00000132170;ENSG00000146648;ENSG00000171105
GO:0030730	sequestering of triglyceride	BP	2	0.000462	0.00412766	ENSG00000132170;ENSG00000186951
GO:0060149	negative regulation of posttranscriptional gene silencing	BP	2	0.000462	0.00412766	ENSG00000091831;ENSG00000132170
GO:0060967	negative regulation of gene silencing by RNA	BP	2	0.000462	0.00412766	ENSG00000091831;ENSG00000132170
GO:0051179	localization	BP	20	0.000481	0.00427849	ENSG00000005381;ENSG00000007171;ENSG00000073756;ENSG00000085563;ENSG00000091831;ENSG00000100823;ENSG00000100985;ENSG00000104267;ENSG00000105976;ENSG00000118777;ENSG00000128052;ENSG00000132170;ENSG00000133742;ENSG00000146648;ENSG00000163464;ENSG00000170890;ENSG00000171105;ENSG00000178623;ENSG00000180210;ENSG00000186951
GO:0043062	extracellular structure organization	BP	5	0.000493	0.00436506	ENSG00000005381;ENSG00000087245;ENSG00000100985;ENSG00000128052;ENSG00000149968
GO:0051704	multi-organism process	BP	12	0.000503	0.00444493	ENSG00000005381;ENSG00000007171;ENSG00000073756;ENSG00000087245;ENSG00000091831;ENSG00000100985;ENSG00000105976;ENSG00000128052;ENSG00000146648;ENSG00000170890;ENSG00000171105;ENSG00000180210
GO:0051195	negative regulation of cofactor metabolic process	BP	2	0.000506	0.00444514	ENSG00000149968;ENSG00000186951
GO:1902531	regulation of intracellular signal transduction	BP	10	0.000507	0.00444514	ENSG00000073756;ENSG00000091831;ENSG00000100985;ENSG00000105976;ENSG00000128052;ENSG00000146648;ENSG00000170890;ENSG00000171105;ENSG00000178623;ENSG00000180210
GO:0009755	hormone-mediated signaling pathway	BP	4	0.000517	0.00451534	ENSG00000091831;ENSG00000132170;ENSG00000143365;ENSG00000186951
GO:0051896	regulation of protein kinase B signaling	BP	4	0.000532	0.00461748	ENSG00000091831;ENSG00000105976;ENSG00000146648;ENSG00000171105
GO:0001667	ameboidal-type cell migration	BP	5	0.000533	0.00461748	ENSG00000073756;ENSG00000100985;ENSG00000105976;ENSG00000128052;ENSG00000132170
GO:0019932	second-messenger-mediated signaling	BP	5	0.000543	0.00469046	ENSG00000007171;ENSG00000106546;ENSG00000128052;ENSG00000146648;ENSG00000163464
GO:1900371	regulation of purine nucleotide biosynthetic process	BP	3	0.00055	0.00473056	ENSG00000007171;ENSG00000171105;ENSG00000186951
GO:2001026	regulation of endothelial cell chemotaxis	BP	2	0.000552	0.00473056	ENSG00000105976;ENSG00000128052
GO:0006874	cellular calcium ion homeostasis	BP	5	0.000553	0.00473056	ENSG00000091831;ENSG00000163464;ENSG00000170890;ENSG00000178623;ENSG00000180210
GO:0030808	regulation of nucleotide biosynthetic process	BP	3	0.000565	0.00481273	ENSG00000007171;ENSG00000171105;ENSG00000186951

GO:0001676	long-chain fatty acid metabolic process	BP	3	0.00058	0.00492184	ENSG00000073756;ENSG00000095303;ENSG00000159228
GO:0042752	regulation of circadian rhythm	BP	3	0.000595	0.00503228	ENSG00000132170;ENSG00000143365;ENSG00000186951
GO:0010888	negative regulation of lipid storage	BP	2	0.000599	0.00504808	ENSG00000132170;ENSG00000186951
GO:0042180	cellular ketone metabolic process	BP	4	0.000604	0.00507335	ENSG00000073756;ENSG00000132170;ENSG00000186951;ENSG00000198074
GO:0043067	regulation of programmed cell death	BP	9	0.00061	0.00507335	ENSG00000005381;ENSG00000073756;ENSG00000091831;ENSG00000100823;ENSG00000100985;ENSG00000105976;ENSG00000128052;ENSG00000132170;ENSG00000146648
GO:0010646	regulation of cell communication	BP	14	0.00061	0.00507335	ENSG00000007171;ENSG00000073756;ENSG00000091831;ENSG00000100985;ENSG00000104267;ENSG00000105426;ENSG00000105976;ENSG00000128052;ENSG00000132170;ENSG00000146648;ENSG00000170890;ENSG00000171105;ENSG00000178623;ENSG00000180210
GO:0051193	regulation of cofactor metabolic process	BP	3	0.00061	0.00507335	ENSG00000149968;ENSG00000171105;ENSG00000186951
GO:0055074	calcium ion homeostasis	BP	5	0.000626	0.00518201	ENSG00000091831;ENSG00000163464;ENSG00000170890;ENSG00000178623;ENSG00000180210
GO:0009894	regulation of catabolic process	BP	7	0.00064	0.00526885	ENSG00000007171;ENSG00000100823;ENSG00000105976;ENSG00000128052;ENSG00000146648;ENSG00000171105;ENSG00000186951
GO:0010906	regulation of glucose metabolic process	BP	3	0.000642	0.00526885	ENSG00000143365;ENSG00000171105;ENSG00000186951
GO:0007169	transmembrane receptor protein tyrosine kinase signaling pathway	BP	6	0.000643	0.00526885	ENSG00000087245;ENSG00000100985;ENSG00000105976;ENSG00000128052;ENSG00000146648;ENSG00000171105
GO:0051924	regulation of calcium ion transport	BP	4	0.000647	0.00528742	ENSG00000073756;ENSG00000170890;ENSG00000178623;ENSG00000180210
GO:0045926	negative regulation of growth	BP	4	0.000656	0.00534006	ENSG00000005381;ENSG00000105426;ENSG00000132170;ENSG00000186951
GO:0060964	regulation of gene silencing by miRNA	BP	3	0.000658	0.00534006	ENSG00000091831;ENSG00000132170;ENSG00000146648
GO:0051241	negative regulation of multicellular organismal process	BP	8	0.00067	0.00541761	ENSG00000073756;ENSG00000100985;ENSG00000105426;ENSG00000132170;ENSG00000146648;ENSG00000178623;ENSG00000180210;ENSG00000186951
GO:0043542	endothelial cell migration	BP	4	0.000674	0.00541828	ENSG00000073756;ENSG00000105976;ENSG00000128052;ENSG00000132170
GO:0098869	cellular oxidant detoxification	BP	3	0.000674	0.00541828	ENSG00000005381;ENSG00000073756;ENSG00000095303
GO:0048583	regulation of response to stimulus	BP	16	0.000681	0.00545128	ENSG00000007171;ENSG00000073756;ENSG00000085563;ENSG00000091831;ENSG00000100985;ENSG00000105426;ENSG00000105976;ENSG00000128052;ENSG00000132170;ENSG00000146648;ENSG00000149968;ENSG00000170890;ENSG00000171105;ENSG00000178623;ENSG00000180210;ENSG00000186951
GO:0023051	regulation of signaling	BP	14	0.000694	0.00554058	ENSG00000007171;ENSG00000073756;ENSG00000091831;ENSG00000100985;ENSG00000104267;ENSG00000105426;ENSG00000105976;ENSG00000128052;ENSG00000132170;ENSG00000146648;ENSG00000170890;ENSG00000171105;ENSG00000178623;ENSG00000180210
GO:1905208	negative regulation of cardiocyte differentiation	BP	2	0.000699	0.00556594	ENSG00000146648;ENSG00000186951
GO:0033559	unsaturated fatty acid metabolic process	BP	3	0.000708	0.00557625	ENSG00000073756;ENSG00000095303;ENSG00000159228
GO:0060147	regulation of posttranscriptional gene silencing	BP	3	0.000708	0.00557625	ENSG00000091831;ENSG00000132170;ENSG00000146648

GO:0060966	regulation of gene silencing by RNA	BP	3	0.000708	0.00557625	ENSG00000091831;ENSG00000132170;ENSG00000146648
GO:0042136	neurotransmitter biosynthetic process	BP	3	0.000725	0.00569271	ENSG00000007171;ENSG00000073756;ENSG00000171105
GO:0032409	regulation of transporter activity	BP	4	0.00073	0.00571089	ENSG00000085563;ENSG00000100985;ENSG00000132170;ENSG00000178623
GO:0065009	regulation of molecular function	BP	13	0.000747	0.00581262	ENSG00000007171;ENSG00000073756;ENSG00000085563;ENSG00000091831;ENSG00000100985;ENSG00000105976;ENSG00000132170;ENSG00000146648;ENSG00000170890;ENSG00000171105;ENSG00000178623;ENSG00000180210;ENSG00000186951
GO:0001934	positive regulation of protein phosphorylation	BP	7	0.000747	0.00581262	ENSG00000073756;ENSG00000100985;ENSG00000128052;ENSG00000146648;ENSG00000170890;ENSG00000171105;ENSG00000180210
GO:0031667	response to nutrient levels	BP	5	0.00075	0.00581482	ENSG00000005381;ENSG00000073756;ENSG00000132170;ENSG00000146648;ENSG00000186951
GO:0009967	positive regulation of signal transduction	BP	9	0.000758	0.00585402	ENSG00000091831;ENSG00000100985;ENSG00000105976;ENSG00000128052;ENSG00000146648;ENSG00000170890;ENSG00000171105;ENSG00000178623;ENSG00000180210
GO:0045446	endothelial cell differentiation	BP	3	0.00076	0.00585402	ENSG00000105426;ENSG00000105976;ENSG00000128052
GO:0044419	interspecies interaction between organisms	BP	7	0.000772	0.00592985	ENSG00000005381;ENSG00000007171;ENSG00000105976;ENSG00000128052;ENSG00000146648;ENSG00000171105;ENSG00000180210
GO:0032868	response to insulin	BP	4	0.000778	0.00595896	ENSG00000132170;ENSG00000170890;ENSG00000171105;ENSG00000186951
GO:0072503	cellular divalent inorganic cation homeostasis	BP	5	0.000791	0.00603393	ENSG00000091831;ENSG00000163464;ENSG00000170890;ENSG00000178623;ENSG00000180210
GO:0016049	cell growth	BP	5	0.000798	0.00606781	ENSG00000105426;ENSG00000132170;ENSG00000146648;ENSG00000180210;ENSG00000186951
GO:0051172	negative regulation of nitrogen compound metabolic process	BP	11	0.000801	0.00607309	ENSG00000007171;ENSG00000073756;ENSG00000091831;ENSG00000100823;ENSG00000100985;ENSG00000106546;ENSG00000132170;ENSG00000143365;ENSG00000146648;ENSG00000180210;ENSG00000186951
GO:1990776	response to angiotensin	BP	2	0.000808	0.00609971	ENSG00000073756;ENSG00000104267
GO:0006810	transport	BP	17	0.000812	0.00609971	ENSG00000005381;ENSG00000007171;ENSG00000073756;ENSG00000085563;ENSG00000100985;ENSG00000104267;ENSG00000105976;ENSG00000118777;ENSG00000132170;ENSG00000133742;ENSG00000146648;ENSG00000163464;ENSG00000170890;ENSG00000171105;ENSG00000178623;ENSG00000180210;ENSG00000186951
GO:0006805	xenobiotic metabolic process	BP	3	0.000814	0.00609971	ENSG00000095303;ENSG00000106546;ENSG00000143365
GO:0010595	positive regulation of endothelial cell migration	BP	3	0.000814	0.00609971	ENSG00000073756;ENSG00000105976;ENSG00000128052
GO:0051128	regulation of cellular component organization	BP	11	0.000854	0.00637446	ENSG00000091831;ENSG00000100985;ENSG00000105426;ENSG00000105976;ENSG00000128052;ENSG00000132170;ENSG00000146648;ENSG00000149968;ENSG00000171105;ENSG00000180210;ENSG00000186951
GO:0006352	DNA-templated transcription, initiation	BP	4	0.000862	0.00641223	ENSG00000091831;ENSG00000132170;ENSG00000143365;ENSG00000186951
GO:0046320	regulation of fatty acid oxidation	BP	2	0.000865	0.0064151	ENSG00000132170;ENSG00000186951
GO:0048585	negative regulation of response to stimulus	BP	9	0.000913	0.00674785	ENSG00000073756;ENSG00000091831;ENSG00000100985;ENSG00000105426;ENSG00000105976;ENSG00000132170;ENSG00000146648;ENSG00000180210;ENSG00000186951
GO:0002227	innate immune response in mucosa	BP	2	0.000923	0.00674785	ENSG00000007171;ENSG00000170890

GO:0010743	regulation of macrophage derived foam cell differentiation	BP	2	0.000923	0.00674785	ENSG00000132170;ENSG00000186951
GO:0032800	receptor biosynthetic process	BP	2	0.000923	0.00674785	ENSG00000132170;ENSG00000186951
GO:0035767	endothelial cell chemotaxis	BP	2	0.000923	0.00674785	ENSG00000105976;ENSG00000128052
GO:1900542	regulation of purine nucleotide metabolic process	BP	3	0.000931	0.0067834	ENSG00000007171;ENSG00000171105;ENSG00000186951
GO:0032501	multicellular organismal process	BP	21	0.000951	0.00691058	ENSG00000005381;ENSG00000007171;ENSG00000073756;ENSG00000087245;ENSG00000091831;ENSG00000095303;ENSG00000100985;ENSG00000104267;ENSG00000105426;ENSG00000105976;ENSG00000106546;ENSG00000128052;ENSG00000132170;ENSG00000143365;ENSG00000146648;ENSG00000148680;ENSG00000170890;ENSG00000171105;ENSG00000178623;ENSG00000180210;ENSG00000186951
GO:0048523	negative regulation of cellular process	BP	16	0.000987	0.00714883	ENSG00000005381;ENSG00000073756;ENSG00000091831;ENSG00000100823;ENSG00000100985;ENSG00000105426;ENSG00000105976;ENSG00000106546;ENSG00000128052;ENSG00000132170;ENSG00000143365;ENSG00000146648;ENSG00000149968;ENSG00000178623;ENSG00000180210;ENSG00000186951
GO:0045596	negative regulation of cell differentiation	BP	6	0.00099	0.00714883	ENSG00000100985;ENSG00000105426;ENSG00000132170;ENSG00000146648;ENSG00000180210;ENSG00000186951
GO:0009991	response to extracellular stimulus	BP	5	0.000995	0.00714883	ENSG00000005381;ENSG00000073756;ENSG00000132170;ENSG00000146648;ENSG00000186951
GO:0042327	positive regulation of phosphorylation	BP	7	0.000996	0.00714883	ENSG00000073756;ENSG00000100985;ENSG00000128052;ENSG00000146648;ENSG00000170890;ENSG00000171105;ENSG00000180210
GO:0006140	regulation of nucleotide metabolic process	BP	3	0.001014	0.00721624	ENSG00000007171;ENSG00000171105;ENSG00000186951
GO:0032355	response to estradiol	BP	3	0.001014	0.00721624	ENSG00000073756;ENSG00000091831;ENSG00000146648
GO:0046683	response to organophosphorus	BP	3	0.001014	0.00721624	ENSG00000073756;ENSG00000100823;ENSG00000106546
GO:0010605	negative regulation of macromolecule metabolic process	BP	12	0.001027	0.00728396	ENSG00000007171;ENSG00000073756;ENSG00000091831;ENSG00000100823;ENSG00000100985;ENSG00000106546;ENSG00000128052;ENSG00000132170;ENSG00000143365;ENSG00000146648;ENSG00000180210;ENSG00000186951
GO:0060548	negative regulation of cell death	BP	7	0.001038	0.00734031	ENSG00000005381;ENSG00000073756;ENSG00000100985;ENSG00000105976;ENSG00000128052;ENSG00000146648;ENSG00000186951
GO:0051234	establishment of localization	BP	17	0.001066	0.00751997	ENSG00000005381;ENSG00000007171;ENSG00000073756;ENSG00000085563;ENSG00000100985;ENSG00000104267;ENSG00000105976;ENSG00000118777;ENSG00000132170;ENSG00000133742;ENSG00000146648;ENSG00000163464;ENSG00000170890;ENSG00000171105;ENSG00000178623;ENSG00000180210;ENSG00000186951
GO:0045598	regulation of fat cell differentiation	BP	3	0.00108	0.00759261	ENSG00000073756;ENSG00000132170;ENSG00000143365
GO:0035296	regulation of tube diameter	BP	3	0.001102	0.00770531	ENSG00000073756;ENSG00000146648;ENSG00000148680
GO:0097746	regulation of blood vessel diameter	BP	3	0.001102	0.00770531	ENSG00000073756;ENSG00000146648;ENSG00000148680
GO:0045907	positive regulation of vasoconstriction	BP	2	0.001111	0.00774464	ENSG00000073756;ENSG00000146648
GO:0010648	negative regulation of cell communication	BP	8	0.001119	0.00777827	ENSG00000073756;ENSG00000091831;ENSG00000100985;ENSG00000105426;ENSG00000105976;ENSG00000132170;ENSG00000146648;ENSG00000178623
GO:0010675	regulation of cellular carbohydrate metabolic process	BP	3	0.001125	0.00779635	ENSG00000143365;ENSG00000171105;ENSG00000186951
GO:0023057	negative regulation of signaling	BP	8	0.001138	0.00786756	ENSG00000073756;ENSG00000091831;ENSG00000100985;ENSG00000105426;ENSG00000105976;ENSG00000132170;ENSG00000146648;ENSG00000178623

GO:0012501	programmed cell death	BP	10	0.001142	0.00787098	ENSG00000005381;ENSG00000073756;ENSG00000091831;ENSG00000100823;ENSG00000100985;ENSG00000105976;ENSG00000106546;ENSG00000128052;ENSG00000132170;ENSG00000146648
GO:0035295	tube development	BP	7	0.001149	0.00789292	ENSG00000073756;ENSG00000087245;ENSG00000091831;ENSG00000105976;ENSG00000128052;ENSG00000132170;ENSG00000146648
GO:0007263	nitric oxide mediated signal transduction	BP	2	0.001177	0.00802168	ENSG00000007171;ENSG00000146648
GO:0051968	positive regulation of synaptic transmission, glutamatergic	BP	2	0.001177	0.00802168	ENSG00000073756;ENSG00000146648
GO:0071392	cellular response to estradiol stimulus	BP	2	0.001177	0.00802168	ENSG00000091831;ENSG00000146648
GO:0019220	regulation of phosphate metabolic process	BP	9	0.001204	0.00818408	ENSG00000007171;ENSG00000073756;ENSG00000100985;ENSG00000128052;ENSG00000146648;ENSG00000170890;ENSG00000171105;ENSG00000180210;ENSG00000186951
GO:0051174	regulation of phosphorus metabolic process	BP	9	0.001213	0.00822189	ENSG00000007171;ENSG00000073756;ENSG00000100985;ENSG00000128052;ENSG00000146648;ENSG00000170890;ENSG00000171105;ENSG00000180210;ENSG00000186951
GO:0003158	endothelium development	BP	3	0.001243	0.00839696	ENSG00000105426;ENSG00000105976;ENSG00000128052
GO:0009895	negative regulation of catabolic process	BP	4	0.001285	0.00865723	ENSG00000007171;ENSG00000105976;ENSG00000146648;ENSG00000186951
GO:0043467	regulation of generation of precursor metabolites and energy	BP	3	0.001292	0.00865723	ENSG00000007171;ENSG00000171105;ENSG00000186951
GO:0050880	regulation of blood vessel size	BP	3	0.001292	0.00865723	ENSG00000073756;ENSG00000146648;ENSG00000148680
GO:0010742	macrophage derived foam cell differentiation	BP	2	0.001315	0.00870475	ENSG00000132170;ENSG00000186951
GO:0034405	response to fluid shear stress	BP	2	0.001315	0.00870475	ENSG00000073756;ENSG00000104267
GO:0045730	respiratory burst	BP	2	0.001315	0.00870475	ENSG00000005381;ENSG00000171105
GO:0090077	foam cell differentiation	BP	2	0.001315	0.00870475	ENSG00000132170;ENSG00000186951
GO:0035150	regulation of tube size	BP	3	0.001317	0.00870475	ENSG00000073756;ENSG00000146648;ENSG00000148680
GO:0031324	negative regulation of cellular metabolic process	BP	11	0.001329	0.00876147	ENSG00000073756;ENSG00000091831;ENSG00000100823;ENSG00000100985;ENSG00000105976;ENSG00000106546;ENSG00000132170;ENSG00000143365;ENSG00000149968;ENSG00000180210;ENSG00000186951
GO:0019359	nicotinamide nucleotide biosynthetic process	BP	3	0.001342	0.00880042	ENSG00000073756;ENSG00000171105;ENSG00000186951
GO:0019363	pyridine nucleotide biosynthetic process	BP	3	0.001342	0.00880042	ENSG00000073756;ENSG00000171105;ENSG00000186951
GO:0072525	pyridine-containing compound biosynthetic process	BP	3	0.00142	0.00917985	ENSG00000073756;ENSG00000171105;ENSG00000186951
GO:0048513	animal organ development	BP	13	0.001421	0.00917985	ENSG00000073756;ENSG00000087245;ENSG00000091831;ENSG00000100985;ENSG00000104267;ENSG00000105426;ENSG00000105976;ENSG00000128052;ENSG00000132170;ENSG00000143365;ENSG00000146648;ENSG00000171105;ENSG00000186951
GO:0048519	negative regulation of biological process	BP	17	0.001449	0.00917985	ENSG00000005381;ENSG00000007171;ENSG00000073756;ENSG00000091831;ENSG00000100823;ENSG00000100985;ENSG00000105426;ENSG00000105976;ENSG00000106546;ENSG00000128052;ENSG00000132170;ENSG00000143365;ENSG00000146648;ENSG00000149968;ENSG00000178623;ENSG00000180210;ENSG00000186951

GO:0002148	hypochlorous acid metabolic process	BP	1	0.001453	0.00917985	ENSG00000005381
GO:0002149	hypochlorous acid biosynthetic process	BP	1	0.001453	0.00917985	ENSG00000005381
GO:0010335	response to non-ionic osmotic stress	BP	1	0.001453	0.00917985	ENSG00000073756
GO:0051838	cytolysis by host of symbiont cells	BP	1	0.001453	0.00917985	ENSG00000180210
GO:0071471	cellular response to non-ionic osmotic stress	BP	1	0.001453	0.00917985	ENSG00000073756
GO:0072366	regulation of cellular ketone metabolic process by positive regulation of transcription from	BP	1	0.001453	0.00917985	ENSG00000186951
GO:0097698	telomere maintenance via base-excision repair	BP	1	0.001453	0.00917985	ENSG00000100823
GO:1904456	negative regulation of neuronal action potential	BP	1	0.001453	0.00917985	ENSG00000178623
GO:1990268	response to gold nanoparticle	BP	1	0.001453	0.00917985	ENSG00000005381
GO:2000230	negative regulation of pancreatic stellate cell proliferation	BP	1	0.001453	0.00917985	ENSG00000132170
GO:0032094	response to food	BP	2	0.001461	0.00917985	ENSG00000005381;ENSG00000186951
GO:0090050	positive regulation of cell migration involved in sprouting angiogenesis	BP	2	0.001461	0.00917985	ENSG00000073756;ENSG00000128052
GO:0015849	organic acid transport	BP	4	0.001465	0.00917985	ENSG00000007171;ENSG00000132170;ENSG00000170890;ENSG00000186951
GO:0046942	carboxylic acid transport	BP	4	0.001465	0.00917985	ENSG00000007171;ENSG00000132170;ENSG00000170890;ENSG00000186951
GO:0002446	neutrophil mediated immunity	BP	5	0.001474	0.00921359	ENSG00000005381;ENSG00000100985;ENSG00000163464;ENSG00000170890;ENSG00000180210
GO:0060326	cell chemotaxis	BP	4	0.001496	0.00932901	ENSG00000105976;ENSG00000128052;ENSG00000163464;ENSG00000170890
GO:0001890	placenta development	BP	3	0.001529	0.00945845	ENSG00000073756;ENSG00000132170;ENSG00000146648
GO:0060968	regulation of gene silencing	BP	3	0.001529	0.00945845	ENSG00000091831;ENSG00000132170;ENSG00000146648
GO:0071236	cellular response to antibiotic	BP	3	0.001529	0.00945845	ENSG00000100823;ENSG00000105976;ENSG00000106546
GO:0044237	cellular metabolic process	BP	25	0.00154	0.00950504	ENSG00000005381;ENSG00000007171;ENSG00000073756;ENSG00000087245;ENSG00000091831;ENSG00000095303;ENSG00000100823;ENSG00000100985;ENSG00000105426;ENSG00000105976;ENSG00000106546;ENSG00000118777;ENSG00000128052;ENSG00000132170;ENSG00000133742;ENSG00000143365;ENSG00000146648;ENSG00000149968;ENSG00000159228;ENSG00000163464;ENSG00000170890;ENSG00000171105;ENSG00000180210;ENSG00000186951;ENSG00000198074
GO:0060969	negative regulation of gene silencing	BP	2	0.001614	0.00993238	ENSG00000091831;ENSG00000132170
GO:0010907	positive regulation of glucose metabolic process	BP	2	0.001693	0.01026293	ENSG00000171105;ENSG00000186951

GO:0015701	bicarbonate transport	BP	2	0.001693	0.01026293	ENSG00000104267;ENSG00000133742
GO:0030810	positive regulation of nucleotide biosynthetic process	BP	2	0.001693	0.01026293	ENSG00000007171;ENSG00000171105
GO:0045124	regulation of bone resorption	BP	2	0.001693	0.01026293	ENSG00000104267;ENSG00000146648
GO:0071276	cellular response to cadmium ion	BP	2	0.001693	0.01026293	ENSG00000100985;ENSG00000146648
GO:1900373	positive regulation of purine nucleotide biosynthetic process	BP	2	0.001693	0.01026293	ENSG00000007171;ENSG00000171105
GO:0043535	regulation of blood vessel endothelial cell migration	BP	3	0.001701	0.01028645	ENSG00000073756;ENSG00000128052;ENSG00000132170
GO:0002385	mucosal immune response	BP	2	0.001774	0.01067363	ENSG00000007171;ENSG00000170890
GO:0010863	positive regulation of phospholipase C activity	BP	2	0.001774	0.01067363	ENSG00000091831;ENSG00000146648
GO:0043271	negative regulation of ion transport	BP	3	0.001791	0.01075262	ENSG00000073756;ENSG00000100985;ENSG00000178623
GO:0045944	positive regulation of transcription by RNA polymerase II	BP	7	0.001801	0.01078419	ENSG00000091831;ENSG00000105976;ENSG00000106546;ENSG00000132170;ENSG00000146648;ENSG00000170890;ENSG00000186951
GO:0044092	negative regulation of molecular function	BP	7	0.001826	0.01090572	ENSG00000073756;ENSG00000091831;ENSG00000100985;ENSG00000105976;ENSG00000132170;ENSG00000178623;ENSG00000186951
GO:0002376	immune system process	BP	13	0.001856	0.01105638	ENSG00000005381;ENSG00000007171;ENSG00000091831;ENSG00000100985;ENSG00000104267;ENSG00000105426;ENSG00000106546;ENSG00000128052;ENSG00000132170;ENSG00000143365;ENSG00000163464;ENSG00000170890;ENSG00000180210
GO:0032965	regulation of collagen biosynthetic process	BP	2	0.001941	0.01150918	ENSG00000132170;ENSG00000180210
GO:1900274	regulation of phospholipase C activity	BP	2	0.001941	0.01150918	ENSG00000091831;ENSG00000146648
GO:0032502	developmental process	BP	18	0.001987	0.01175277	ENSG00000005381;ENSG00000073756;ENSG00000087245;ENSG00000091831;ENSG00000100823;ENSG00000100985;ENSG00000104267;ENSG00000105426;ENSG00000105976;ENSG00000106546;ENSG00000128052;ENSG00000132170;ENSG00000143365;ENSG00000146648;ENSG00000159228;ENSG00000171105;ENSG00000180210;ENSG00000186951
GO:0002251	organ or tissue specific immune response	BP	2	0.002028	0.01193376	ENSG00000007171;ENSG00000170890
GO:0032309	icosanoid secretion	BP	2	0.002028	0.01193376	ENSG00000007171;ENSG00000170890
GO:0042133	neurotransmitter metabolic process	BP	3	0.002047	0.01198983	ENSG00000007171;ENSG00000073756;ENSG00000171105
GO:0045931	positive regulation of mitotic cell cycle	BP	3	0.002047	0.01198983	ENSG00000100823;ENSG00000146648;ENSG00000171105
GO:0034105	positive regulation of tissue remodeling	BP	2	0.002116	0.01227376	ENSG00000104267;ENSG00000146648
GO:0035196	production of miRNAs involved in gene silencing by miRNA	BP	2	0.002116	0.01227376	ENSG00000091831;ENSG00000146648
GO:0045776	negative regulation of blood pressure	BP	2	0.002116	0.01227376	ENSG00000007171;ENSG00000186951

GO:1904707	positive regulation of vascular smooth muscle cell proliferation	BP	2	0.002116	0.01227376	ENSG00000087245;ENSG00000100985
GO:0003018	vascular process in circulatory system	BP	3	0.002148	0.01243345	ENSG00000073756;ENSG00000146648;ENSG00000148680
GO:0050728	negative regulation of inflammatory response	BP	3	0.002183	0.01260294	ENSG00000132170;ENSG00000180210;ENSG00000186951
GO:0002444	myeloid leukocyte mediated immunity	BP	5	0.002194	0.01261393	ENSG00000005381;ENSG00000100985;ENSG00000163464;ENSG00000170890;ENSG00000180210
GO:0010628	positive regulation of gene expression	BP	9	0.002195	0.01261393	ENSG00000091831;ENSG00000105976;ENSG00000106546;ENSG00000132170;ENSG00000143365;ENSG00000146648;ENSG00000170890;ENSG00000171105;ENSG00000186951
GO:0061614	pri-miRNA transcription by RNA polymerase II	BP	2	0.002206	0.01264428	ENSG00000132170;ENSG00000186951
GO:0030154	cell differentiation	BP	14	0.002219	0.01269193	ENSG00000073756;ENSG00000087245;ENSG00000091831;ENSG00000100985;ENSG00000104267;ENSG00000105426;ENSG00000105976;ENSG00000128052;ENSG00000132170;ENSG00000143365;ENSG00000146648;ENSG00000159228;ENSG00000180210;ENSG00000186951
GO:0001666	response to hypoxia	BP	4	0.002231	0.0126998	ENSG00000007171;ENSG00000073756;ENSG00000087245;ENSG00000186951
GO:0031401	positive regulation of protein modification process	BP	7	0.002231	0.0126998	ENSG00000073756;ENSG00000100985;ENSG00000128052;ENSG00000146648;ENSG00000170890;ENSG00000171105;ENSG00000180210
GO:0010038	response to metal ion	BP	4	0.002273	0.01290712	ENSG00000073756;ENSG00000100985;ENSG00000104267;ENSG00000146648
GO:0018108	peptidyl-tyrosine phosphorylation	BP	4	0.002294	0.01299765	ENSG00000105976;ENSG00000128052;ENSG00000146648;ENSG00000171105
GO:0048771	tissue remodeling	BP	3	0.002324	0.01313732	ENSG00000087245;ENSG00000104267;ENSG00000146648
GO:0051098	regulation of binding	BP	4	0.002337	0.0131798	ENSG00000100985;ENSG00000105976;ENSG00000132170;ENSG00000186951
GO:0042325	regulation of phosphorylation	BP	8	0.002351	0.01322564	ENSG00000073756;ENSG00000100985;ENSG00000128052;ENSG00000146648;ENSG00000170890;ENSG00000171105;ENSG00000180210;ENSG00000186951
GO:0018212	peptidyl-tyrosine modification	BP	4	0.002359	0.01323644	ENSG00000105976;ENSG00000128052;ENSG00000146648;ENSG00000171105
GO:0032102	negative regulation of response to external stimulus	BP	4	0.002381	0.01323644	ENSG00000105426;ENSG00000132170;ENSG00000180210;ENSG00000186951
GO:0010712	regulation of collagen metabolic process	BP	2	0.002391	0.01323644	ENSG00000132170;ENSG00000180210
GO:0035272	exocrine system development	BP	2	0.002391	0.01323644	ENSG00000146648;ENSG00000171105
GO:0046850	regulation of bone remodeling	BP	2	0.002391	0.01323644	ENSG00000104267;ENSG00000146648
GO:0071715	icosanoid transport	BP	2	0.002391	0.01323644	ENSG00000007171;ENSG00000170890
GO:1901571	fatty acid derivative transport	BP	2	0.002391	0.01323644	ENSG00000007171;ENSG00000170890
GO:0030198	extracellular matrix organization	BP	4	0.002425	0.01339372	ENSG00000087245;ENSG00000100985;ENSG00000128052;ENSG00000149968

GO:0032270	positive regulation of cellular protein metabolic process	BP	8	0.002467	0.01342636	ENSG00000073756;ENSG00000100985;ENSG00000128052;ENSG00000132170;ENSG00000146648;ENSG00000170890;ENSG00000171105;ENSG00000180210
GO:0036293	response to decreased oxygen levels	BP	4	0.00247	0.01342636	ENSG00000007171;ENSG00000073756;ENSG00000087245;ENSG00000186951
GO:0031050	dsRNA processing	BP	2	0.002487	0.01342636	ENSG00000091831;ENSG00000146648
GO:0035987	endodermal cell differentiation	BP	2	0.002487	0.01342636	ENSG00000087245;ENSG00000100985
GO:0045429	positive regulation of nitric oxide biosynthetic process	BP	2	0.002487	0.01342636	ENSG00000073756;ENSG00000171105
GO:0045981	positive regulation of nucleotide metabolic process	BP	2	0.002487	0.01342636	ENSG00000007171;ENSG00000171105
GO:0070918	production of small RNA involved in gene silencing by RNA	BP	2	0.002487	0.01342636	ENSG00000091831;ENSG00000146648
GO:1900087	positive regulation of G1/S transition of mitotic cell cycle	BP	2	0.002487	0.01342636	ENSG00000100823;ENSG00000146648
GO:1900544	positive regulation of purine nucleotide metabolic process	BP	2	0.002487	0.01342636	ENSG00000007171;ENSG00000171105
GO:1904407	positive regulation of nitric oxide metabolic process	BP	2	0.002487	0.01342636	ENSG00000073756;ENSG00000171105
GO:0042981	regulation of apoptotic process	BP	8	0.002512	0.01353505	ENSG00000005381;ENSG00000073756;ENSG00000091831;ENSG00000100823;ENSG00000100985;ENSG00000128052;ENSG00000132170;ENSG00000146648
GO:0043534	blood vessel endothelial cell migration	BP	3	0.002547	0.01366154	ENSG00000073756;ENSG00000128052;ENSG00000132170
GO:0051099	positive regulation of binding	BP	3	0.002547	0.01366154	ENSG00000100985;ENSG00000105976;ENSG00000132170
GO:0016043	cellular component organization	BP	18	0.002609	0.01395107	ENSG00000005381;ENSG00000007171;ENSG00000085563;ENSG00000087245;ENSG00000091831;ENSG00000100823;ENSG00000100985;ENSG00000105426;ENSG00000105976;ENSG00000128052;ENSG00000132170;ENSG00000146648;ENSG00000149968;ENSG00000170890;ENSG00000171105;ENSG00000178623;ENSG00000180210;ENSG00000186951
GO:0019362	pyridine nucleotide metabolic process	BP	3	0.002624	0.01395107	ENSG00000073756;ENSG00000171105;ENSG00000186951
GO:0035821	modification of morphology or physiology of other organism	BP	3	0.002624	0.01395107	ENSG00000007171;ENSG00000171105;ENSG00000180210
GO:0046496	nicotinamide nucleotide metabolic process	BP	3	0.002624	0.01395107	ENSG00000073756;ENSG00000171105;ENSG00000186951
GO:0001503	ossification	BP	4	0.002678	0.01410488	ENSG00000073756;ENSG00000087245;ENSG00000100985;ENSG00000146648
GO:0010883	regulation of lipid storage	BP	2	0.002683	0.01410488	ENSG00000132170;ENSG00000186951
GO:0071320	cellular response to cAMP	BP	2	0.002683	0.01410488	ENSG00000100823;ENSG00000106546
GO:2001258	negative regulation of cation channel activity	BP	2	0.002683	0.01410488	ENSG00000100985;ENSG00000178623
GO:0051130	positive regulation of cellular component organization	BP	7	0.002704	0.01410488	ENSG00000091831;ENSG00000100985;ENSG00000105976;ENSG00000128052;ENSG00000132170;ENSG00000149968;ENSG00000171105

GO:0050806	positive regulation of synaptic transmission	BP	3	0.002742	0.01410488	ENSG00000073756;ENSG00000104267;ENSG00000146648
GO:0055085	transmembrane transport	BP	8	0.002761	0.01410488	ENSG00000085563;ENSG00000100985;ENSG00000104267;ENSG00000118777;ENSG00000170890;ENSG00000171105;ENSG00000178623;ENSG00000180210
GO:0006953	acute-phase response	BP	2	0.002783	0.01410488	ENSG00000073756;ENSG00000180210
GO:0010524	positive regulation of calcium ion transport into cytosol	BP	2	0.002783	0.01410488	ENSG00000170890;ENSG00000180210
GO:0032964	collagen biosynthetic process	BP	2	0.002783	0.01410488	ENSG00000132170;ENSG00000180210
GO:0006915	apoptotic process	BP	9	0.002792	0.01410488	ENSG00000005381;ENSG00000073756;ENSG00000091831;ENSG00000100823;ENSG00000100985;ENSG00000106546;ENSG00000128052;ENSG00000132170;ENSG00000146648
GO:0030308	negative regulation of cell growth	BP	3	0.002823	0.01410488	ENSG00000105426;ENSG00000132170;ENSG00000186951
GO:0072524	pyridine-containing compound metabolic process	BP	3	0.002823	0.01410488	ENSG00000073756;ENSG00000171105;ENSG00000186951
GO:0009165	nucleotide biosynthetic process	BP	4	0.002824	0.01410488	ENSG00000007171;ENSG00000073756;ENSG00000171105;ENSG00000186951
GO:0048731	system development	BP	15	0.002858	0.01410488	ENSG00000073756;ENSG00000087245;ENSG00000091831;ENSG00000100985;ENSG00000104267;ENSG00000105426;ENSG00000105976;ENSG00000106546;ENSG00000128052;ENSG00000132170;ENSG00000143365;ENSG00000146648;ENSG00000171105;ENSG00000180210;ENSG00000186951
GO:0010565	regulation of cellular ketone metabolic process	BP	3	0.002864	0.01410488	ENSG00000073756;ENSG00000132170;ENSG00000186951
GO:0006286	base-excision repair, base-free sugar-phosphate removal	BP	1	0.002904	0.01410488	ENSG00000100823
GO:0006714	sesquiterpenoid metabolic process	BP	1	0.002904	0.01410488	ENSG00000198074
GO:0016107	sesquiterpenoid catabolic process	BP	1	0.002904	0.01410488	ENSG00000198074
GO:0016487	farnesol metabolic process	BP	1	0.002904	0.01410488	ENSG00000198074
GO:0016488	farnesol catabolic process	BP	1	0.002904	0.01410488	ENSG00000198074
GO:0038112	interleukin-8-mediated signaling pathway	BP	1	0.002904	0.01410488	ENSG00000163464
GO:0043006	activation of phospholipase A2 activity by calcium-mediated signaling	BP	1	0.002904	0.01410488	ENSG00000146648
GO:0051801	cytolysis in other organism involved in symbiotic interaction	BP	1	0.002904	0.01410488	ENSG00000180210
GO:0060694	regulation of cholesterol transporter activity	BP	1	0.002904	0.01410488	ENSG00000132170
GO:0071306	cellular response to vitamin E	BP	1	0.002904	0.01410488	ENSG00000132170
GO:0072343	pancreatic stellate cell proliferation	BP	1	0.002904	0.01410488	ENSG00000132170

GO:0072363	regulation of glycolytic process by positive regulation of transcription from RNA polymerase II	BP	1	0.002904	0.01410488	ENSG00000186951
GO:0072364	regulation of cellular ketone metabolic process by regulation of transcription from RNA polymerase II	BP	1	0.002904	0.01410488	ENSG00000186951
GO:0072369	regulation of lipid transport by positive regulation of transcription from RNA polymerase II	BP	1	0.002904	0.01410488	ENSG00000186951
GO:0090088	regulation of oligopeptide transport	BP	1	0.002904	0.01410488	ENSG00000104267
GO:0090089	regulation of dipeptide transport	BP	1	0.002904	0.01410488	ENSG00000104267
GO:0090362	positive regulation of platelet-derived growth factor production	BP	1	0.002904	0.01410488	ENSG00000073756
GO:0099040	ceramide translocation	BP	1	0.002904	0.01410488	ENSG00000085563
GO:1901558	response to metformin	BP	1	0.002904	0.01410488	ENSG00000132170
GO:1904613	cellular response to 2,3,7,8-tetrachlorodibenzodioxine	BP	1	0.002904	0.01410488	ENSG00000106546
GO:1904881	cellular response to hydrogen sulfide	BP	1	0.002904	0.01410488	ENSG00000128052
GO:1990267	response to transition metal nanoparticle	BP	1	0.002904	0.01410488	ENSG00000005381
GO:2000229	regulation of pancreatic stellate cell proliferation	BP	1	0.002904	0.01410488	ENSG00000132170
GO:2000878	positive regulation of oligopeptide transport	BP	1	0.002904	0.01410488	ENSG00000104267
GO:2000880	positive regulation of dipeptide transport	BP	1	0.002904	0.01410488	ENSG00000104267
GO:2001148	regulation of dipeptide transmembrane transport	BP	1	0.002904	0.01410488	ENSG00000104267
GO:2001150	positive regulation of dipeptide transmembrane transport	BP	1	0.002904	0.01410488	ENSG00000104267
GO:1901293	nucleoside phosphate biosynthetic process	BP	4	0.002949	0.01429592	ENSG00000007171;ENSG00000073756;ENSG00000171105;ENSG00000186951
GO:0010959	regulation of metal ion transport	BP	4	0.002975	0.01439076	ENSG00000073756;ENSG00000170890;ENSG00000178623;ENSG00000180210
GO:0071248	cellular response to metal ion	BP	3	0.003074	0.01484105	ENSG00000073756;ENSG00000100985;ENSG00000146648
GO:0006796	phosphate-containing compound metabolic process	BP	12	0.003087	0.01487323	ENSG00000007171;ENSG00000073756;ENSG00000100823;ENSG00000100985;ENSG00000105426;ENSG00000105976;ENSG00000128052;ENSG00000146648;ENSG00000170890;ENSG00000171105;ENSG00000180210;ENSG00000186951
GO:0009966	regulation of signal transduction	BP	12	0.003101	0.01491478	ENSG00000073756;ENSG00000091831;ENSG00000100985;ENSG00000105426;ENSG00000105976;ENSG00000128052;ENSG00000132170;ENSG00000146648;ENSG00000170890;ENSG00000171105;ENSG00000178623;ENSG00000180210
GO:0007267	cell-cell signaling	BP	8	0.003137	0.01505409	ENSG00000007171;ENSG00000073756;ENSG00000091831;ENSG00000104267;ENSG00000105426;ENSG00000105976;ENSG00000146648;ENSG00000148680

GO:0003006	developmental process involved in reproduction	BP	5	0.003163	0.01514932	ENSG00000073756;ENSG00000091831;ENSG00000132170;ENSG00000146648;ENSG00000171105
GO:0044403	symbiont process	BP	6	0.003195	0.01525704	ENSG00000005381;ENSG00000105976;ENSG00000128052;ENSG00000146648;ENSG00000171105;ENSG00000180210
GO:0001706	endoderm formation	BP	2	0.003204	0.01525704	ENSG00000087245;ENSG00000100985
GO:0006109	regulation of carbohydrate metabolic process	BP	3	0.003204	0.01525704	ENSG00000143365;ENSG00000171105;ENSG00000186951
GO:0043069	negative regulation of programmed cell death	BP	6	0.00321	0.01525704	ENSG00000005381;ENSG00000073756;ENSG00000100985;ENSG00000105976;ENSG00000128052;ENSG00000146648
GO:0043112	receptor metabolic process	BP	3	0.003248	0.01540696	ENSG00000132170;ENSG00000163464;ENSG00000186951
GO:0009968	negative regulation of signal transduction	BP	7	0.003264	0.01545255	ENSG00000073756;ENSG00000091831;ENSG00000100985;ENSG00000105426;ENSG00000105976;ENSG00000132170;ENSG00000146648
GO:0050789	regulation of biological process	BP	26	0.003281	0.01549949	ENSG00000005381;ENSG00000007171;ENSG00000073756;ENSG00000085563;ENSG00000087245;ENSG00000091831;ENSG00000095303;ENSG00000100823;ENSG00000100985;ENSG00000104267;ENSG00000105426;ENSG00000105976;ENSG00000106546;ENSG00000128052;ENSG00000132170;ENSG00000133742;ENSG00000143365;ENSG00000146648;ENSG00000148680;ENSG00000149968;ENSG00000163464;ENSG00000170890;ENSG00000171105;ENSG00000178623;ENSG00000180210:ENSG00000186951
GO:0006793	phosphorus metabolic process	BP	12	0.003291	0.01551943	ENSG00000007171;ENSG00000073756;ENSG00000100823;ENSG00000100985;ENSG00000105426;ENSG00000105976;ENSG00000128052;ENSG00000146648;ENSG00000170890;ENSG00000171105;ENSG00000180210;ENSG00000186951
GO:0017001	antibiotic catabolic process	BP	2	0.003313	0.0155916	ENSG00000005381;ENSG00000198074
GO:0006733	oxidoreduction coenzyme metabolic process	BP	3	0.003338	0.01567781	ENSG00000073756;ENSG00000171105;ENSG00000186951
GO:0050794	regulation of cellular process	BP	25	0.003419	0.01599032	ENSG00000005381;ENSG00000007171;ENSG00000073756;ENSG00000087245;ENSG00000091831;ENSG00000095303;ENSG00000100823;ENSG00000100985;ENSG00000104267;ENSG00000105426;ENSG00000105976;ENSG00000106546;ENSG00000128052;ENSG00000132170;ENSG00000133742;ENSG00000143365;ENSG00000146648;ENSG00000148680;ENSG00000149968;ENSG00000163464;ENSG00000170890;ENSG00000171105;ENSG00000178623;ENSG00000180210;ENSG00000186951
GO:0019229	regulation of vasoconstriction	BP	2	0.003424	0.01599032	ENSG00000073756;ENSG00000146648
GO:1900015	regulation of cytokine production involved in inflammatory response	BP	2	0.003424	0.01599032	ENSG00000007171;ENSG00000180210
GO:0042742	defense response to bacterium	BP	4	0.00346	0.01612718	ENSG00000005381;ENSG00000007171;ENSG00000170890;ENSG00000180210
GO:0046324	regulation of glucose import	BP	2	0.003537	0.01645399	ENSG00000170890;ENSG00000171105
GO:0048869	cellular developmental process	BP	14	0.003548	0.01647466	ENSG00000073756;ENSG00000087245;ENSG00000091831;ENSG00000100985;ENSG00000104267;ENSG00000105426;ENSG00000105976;ENSG00000128052;ENSG00000132170;ENSG00000143365;ENSG00000146648;ENSG00000159228;ENSG00000180210;ENSG00000186951
GO:0002673	regulation of acute inflammatory response	BP	3	0.003615	0.01669002	ENSG00000073756;ENSG00000132170;ENSG00000180210
GO:0006006	glucose metabolic process	BP	3	0.003615	0.01669002	ENSG00000143365;ENSG00000171105;ENSG00000186951
GO:0031099	regeneration	BP	3	0.003615	0.01669002	ENSG00000105426;ENSG00000132170;ENSG00000146648
GO:1902808	positive regulation of cell cycle G1/S phase transition	BP	2	0.003652	0.01682541	ENSG00000100823;ENSG00000146648
GO:0050673	epithelial cell proliferation	BP	4	0.003663	0.01683344	ENSG00000091831;ENSG00000128052;ENSG00000132170;ENSG00000146648

GO:0071840	cellular component organization or biogenesis	BP	18	0.003667	0.01683344	ENSG00000005381;ENSG00000007171;ENSG00000085563;ENSG00000087245;ENSG00000091831;ENSG00000100823;ENSG00000100985;ENSG00000105426;ENSG00000105976;ENSG00000128052;ENSG00000132170;ENSG00000146648;ENSG00000149968;ENSG00000170890;ENSG00000171105;ENSG00000178623;ENSG00000180210;ENSG00000186951
GO:1901654	response to ketone	BP	3	0.003711	0.01700149	ENSG00000106546;ENSG00000132170;ENSG00000146648
GO:0051247	positive regulation of protein metabolic process	BP	8	0.003724	0.01703112	ENSG00000073756;ENSG00000100985;ENSG00000128052;ENSG00000132170;ENSG00000146648;ENSG00000170890;ENSG00000171105;ENSG00000180210
GO:0009889	regulation of biosynthetic process	BP	14	0.003743	0.01708565	ENSG00000007171;ENSG00000073756;ENSG00000091831;ENSG00000100823;ENSG00000105976;ENSG00000106546;ENSG00000128052;ENSG00000132170;ENSG00000143365;ENSG00000146648;ENSG00000170890;ENSG00000171105;ENSG00000180210;ENSG00000186951
GO:0002534	cytokine production involved in inflammatory response	BP	2	0.003768	0.01710088	ENSG00000007171;ENSG00000180210
GO:0010676	positive regulation of cellular carbohydrate metabolic process	BP	2	0.003768	0.01710088	ENSG00000171105;ENSG00000186951
GO:0043388	positive regulation of DNA binding	BP	2	0.003768	0.01710088	ENSG00000100985;ENSG00000132170
GO:0045453	bone resorption	BP	2	0.003886	0.01758876	ENSG00000104267;ENSG00000146648
GO:0010629	negative regulation of gene expression	BP	9	0.00389	0.01758876	ENSG00000007171;ENSG00000091831;ENSG00000100823;ENSG00000106546;ENSG00000128052;ENSG00000132170;ENSG00000143365;ENSG00000146648;ENSG00000186951
GO:1903428	positive regulation of reactive oxygen species biosynthetic process	BP	2	0.004006	0.01807848	ENSG00000073756;ENSG00000171105
GO:0007268	chemical synaptic transmission	BP	5	0.0041	0.01843315	ENSG00000073756;ENSG00000104267;ENSG00000105426;ENSG00000146648;ENSG00000148680
GO:0098916	anterograde trans-synaptic signaling	BP	5	0.0041	0.01843315	ENSG00000073756;ENSG00000104267;ENSG00000105426;ENSG00000146648;ENSG00000148680
GO:0002064	epithelial cell development	BP	3	0.004109	0.01843979	ENSG00000091831;ENSG00000105426;ENSG00000105976
GO:0060135	maternal process involved in female pregnancy	BP	2	0.004127	0.01848872	ENSG00000073756;ENSG00000091831
GO:0007584	response to nutrient	BP	3	0.004212	0.01883374	ENSG00000073756;ENSG00000132170;ENSG00000146648
GO:0045600	positive regulation of fat cell differentiation	BP	2	0.00425	0.01883534	ENSG00000073756;ENSG00000132170
GO:1905207	regulation of cardiocyte differentiation	BP	2	0.00425	0.01883534	ENSG00000146648;ENSG00000186951
GO:0043393	regulation of protein binding	BP	3	0.004264	0.01883534	ENSG00000100985;ENSG00000105976;ENSG00000186951
GO:0099537	trans-synaptic signaling	BP	5	0.004338	0.01883534	ENSG00000073756;ENSG00000104267;ENSG00000105426;ENSG00000146648;ENSG00000148680
GO:0016095	polyprenol catabolic process	BP	1	0.004353	0.01883534	ENSG00000198074
GO:0032227	negative regulation of synaptic transmission, dopaminergic	BP	1	0.004353	0.01883534	ENSG00000073756
GO:0060523	prostate epithelial cord elongation	BP	1	0.004353	0.01883534	ENSG00000091831

GO:0070494	regulation of thrombin-activated receptor signaling pathway	BP	1	0.004353	0.01883534	ENSG00000105976
GO:0070495	negative regulation of thrombin-activated receptor signaling pathway	BP	1	0.004353	0.01883534	ENSG00000105976
GO:0072361	regulation of glycolytic process by regulation of transcription from RNA polymerase II promoter	BP	1	0.004353	0.01883534	ENSG00000186951
GO:0090360	platelet-derived growth factor production	BP	1	0.004353	0.01883534	ENSG00000073756
GO:0090361	regulation of platelet-derived growth factor production	BP	1	0.004353	0.01883534	ENSG00000073756
GO:0098502	DNA dephosphorylation	BP	1	0.004353	0.01883534	ENSG00000100823
GO:0098679	regulation of carbohydrate catabolic process by regulation of transcription from RNA polymerase II	BP	1	0.004353	0.01883534	ENSG00000186951
GO:0098758	response to interleukin-8	BP	1	0.004353	0.01883534	ENSG00000163464
GO:0098759	cellular response to interleukin-8	BP	1	0.004353	0.01883534	ENSG00000163464
GO:1902722	positive regulation of prolactin secretion	BP	1	0.004353	0.01883534	ENSG00000146648
GO:1904880	response to hydrogen sulfide	BP	1	0.004353	0.01883534	ENSG00000128052
GO:1903672	positive regulation of sprouting angiogenesis	BP	2	0.004375	0.01889989	ENSG00000073756;ENSG00000128052
GO:0032869	cellular response to insulin stimulus	BP	3	0.004424	0.01907404	ENSG00000132170;ENSG00000170890;ENSG00000171105
GO:0090407	organophosphate biosynthetic process	BP	5	0.004436	0.01909298	ENSG00000007171;ENSG00000073756;ENSG00000170890;ENSG00000171105;ENSG00000186951
GO:0099536	synaptic signaling	BP	5	0.004485	0.01913901	ENSG00000073756;ENSG00000104267;ENSG00000105426;ENSG00000146648;ENSG00000148680
GO:0048732	gland development	BP	4	0.004487	0.01913901	ENSG00000091831;ENSG00000105976;ENSG00000146648;ENSG00000171105
GO:0050804	modulation of chemical synaptic transmission	BP	4	0.004487	0.01913901	ENSG00000073756;ENSG00000104267;ENSG00000105426;ENSG00000146648
GO:0001885	endothelial cell development	BP	2	0.004502	0.01913901	ENSG00000105426;ENSG00000105976
GO:0046323	glucose import	BP	2	0.004502	0.01913901	ENSG00000170890;ENSG00000171105
GO:0046686	response to cadmium ion	BP	2	0.004502	0.01913901	ENSG00000100985;ENSG00000146648
GO:1905953	negative regulation of lipid localization	BP	2	0.004502	0.01913901	ENSG00000132170;ENSG00000186951
GO:0099177	regulation of trans-synaptic signaling	BP	4	0.004521	0.01918439	ENSG00000073756;ENSG00000104267;ENSG00000105426;ENSG00000146648

GO:0010001	glial cell differentiation	BP	3	0.004532	0.01919296	ENSG00000132170;ENSG00000146648;ENSG00000180210
GO:0009887	animal organ morphogenesis	BP	6	0.004538	0.01919296	ENSG00000087245;ENSG00000091831;ENSG00000104267;ENSG00000146648;ENSG00000171105;ENSG00000186951
GO:0007167	enzyme linked receptor protein signaling pathway	BP	6	0.004558	0.01924433	ENSG00000087245;ENSG00000100985;ENSG00000105976;ENSG00000128052;ENSG00000146648;ENSG00000171105
GO:0043281	regulation of cysteine-type endopeptidase activity involved in apoptotic process	BP	3	0.004586	0.01930571	ENSG00000073756;ENSG00000100985;ENSG00000132170
GO:0006816	calcium ion transport	BP	4	0.004589	0.01930571	ENSG00000073756;ENSG00000170890;ENSG00000178623;ENSG00000180210
GO:0002573	myeloid leukocyte differentiation	BP	3	0.004753	0.01995605	ENSG00000100985;ENSG00000104267;ENSG00000132170
GO:0071230	cellular response to amino acid stimulus	BP	2	0.00476	0.01995605	ENSG00000087245;ENSG00000146648
GO:0051093	negative regulation of developmental process	BP	6	0.004847	0.02028516	ENSG00000100985;ENSG00000105426;ENSG00000132170;ENSG00000146648;ENSG00000180210;ENSG00000186951
GO:0015909	long-chain fatty acid transport	BP	2	0.004892	0.02040173	ENSG00000132170;ENSG00000170890
GO:0042698	ovulation cycle	BP	2	0.004892	0.02040173	ENSG00000091831;ENSG00000146648
GO:0019722	calcium-mediated signaling	BP	3	0.004923	0.02049548	ENSG00000128052;ENSG00000146648;ENSG00000163464
GO:0031670	cellular response to nutrient	BP	2	0.005025	0.02088559	ENSG00000073756;ENSG00000132170
GO:0022414	reproductive process	BP	7	0.005079	0.02107321	ENSG00000073756;ENSG00000087245;ENSG00000091831;ENSG00000100985;ENSG00000132170;ENSG00000146648;ENSG00000171105
GO:0000003	reproduction	BP	7	0.005134	0.02126669	ENSG00000073756;ENSG00000087245;ENSG00000091831;ENSG00000100985;ENSG00000132170;ENSG00000146648;ENSG00000171105
GO:0045444	fat cell differentiation	BP	3	0.005155	0.0213001	ENSG00000073756;ENSG00000132170;ENSG00000143365
GO:0045428	regulation of nitric oxide biosynthetic process	BP	2	0.00516	0.0213001	ENSG00000073756;ENSG00000171105
GO:0001568	blood vessel development	BP	5	0.005222	0.02152128	ENSG00000073756;ENSG00000087245;ENSG00000106546;ENSG00000128052;ENSG00000132170
GO:0043085	positive regulation of catalytic activity	BP	7	0.005265	0.02166144	ENSG00000007171;ENSG00000091831;ENSG00000132170;ENSG00000146648;ENSG00000170890;ENSG00000171105;ENSG00000180210
GO:0046777	protein autophosphorylation	BP	3	0.005334	0.02190457	ENSG00000128052;ENSG00000146648;ENSG00000171105
GO:0019915	lipid storage	BP	2	0.005435	0.02220744	ENSG00000132170;ENSG00000186951
GO:0051926	negative regulation of calcium ion transport	BP	2	0.005435	0.02220744	ENSG00000073756;ENSG00000178623
GO:1903524	positive regulation of blood circulation	BP	2	0.005435	0.02220744	ENSG00000073756;ENSG00000146648

GO:0034765	regulation of ion transmembrane transport	BP	4	0.005467	0.02229941	ENSG00000085563;ENSG00000100985;ENSG00000178623;ENSG00000180210
GO:0006110	regulation of glycolytic process	BP	2	0.005575	0.02258841	ENSG00000171105;ENSG00000186951
GO:0031640	killing of cells of other organism	BP	2	0.005575	0.02258841	ENSG00000007171;ENSG00000180210
GO:0044364	disruption of cells of other organism	BP	2	0.005575	0.02258841	ENSG00000007171;ENSG00000180210
GO:1900182	positive regulation of protein localization to nucleus	BP	2	0.005575	0.02258841	ENSG00000073756;ENSG00000180210
GO:0003008	system process	BP	9	0.005707	0.02292281	ENSG00000007171;ENSG00000073756;ENSG00000095303;ENSG00000132170;ENSG00000146648;ENSG00000148680;ENSG00000171105;ENSG00000178623;ENSG00000186951
GO:0030811	regulation of nucleotide catabolic process	BP	2	0.005716	0.02292281	ENSG00000171105;ENSG00000186951
GO:1903201	regulation of oxidative stress-induced cell death	BP	2	0.005716	0.02292281	ENSG00000105976;ENSG00000149968
GO:0001878	response to yeast	BP	1	0.005799	0.02292281	ENSG00000005381
GO:0019087	transformation of host cell by virus	BP	1	0.005799	0.02292281	ENSG00000171105
GO:0032849	positive regulation of cellular pH reduction	BP	1	0.005799	0.02292281	ENSG00000104267
GO:0033590	response to cobalamin	BP	1	0.005799	0.02292281	ENSG00000146648
GO:0033594	response to hydroxyisoflavone	BP	1	0.005799	0.02292281	ENSG00000146648
GO:0035633	maintenance of permeability of blood-brain barrier	BP	1	0.005799	0.02292281	ENSG00000073756
GO:0060737	prostate gland morphogenetic growth	BP	1	0.005799	0.02292281	ENSG00000091831
GO:0060750	epithelial cell proliferation involved in mammary gland duct elongation	BP	1	0.005799	0.02292281	ENSG00000091831
GO:0072367	regulation of lipid transport by regulation of transcription from RNA polymerase II promoter	BP	1	0.005799	0.02292281	ENSG00000186951
GO:0099039	sphingolipid translocation	BP	1	0.005799	0.02292281	ENSG00000085563
GO:1901529	positive regulation of anion channel activity	BP	1	0.005799	0.02292281	ENSG00000085563
GO:1904612	response to 2,3,7,8-tetrachlorodibenzodioxine	BP	1	0.005799	0.02292281	ENSG00000106546
GO:0001932	regulation of protein phosphorylation	BP	7	0.005857	0.02304746	ENSG00000073756;ENSG00000100985;ENSG00000128052;ENSG00000146648;ENSG00000170890;ENSG00000171105;ENSG00000180210
GO:0010827	regulation of glucose transmembrane transport	BP	2	0.00586	0.02304746	ENSG00000170890;ENSG00000171105

GO:0071260	cellular response to mechanical stimulus	BP	2	0.00586	0.02304746	ENSG00000073756;ENSG00000146648
GO:0090087	regulation of peptide transport	BP	5	0.005981	0.02346461	ENSG00000007171;ENSG00000073756;ENSG00000104267;ENSG00000146648;ENSG00000170890
GO:0031016	pancreas development	BP	2	0.006004	0.02346461	ENSG00000105976;ENSG00000171105
GO:0032413	negative regulation of ion transmembrane transporter activity	BP	2	0.006004	0.02346461	ENSG00000100985;ENSG00000178623
GO:0051966	regulation of synaptic transmission, glutamatergic	BP	2	0.006004	0.02346461	ENSG00000073756;ENSG00000146648
GO:0019318	hexose metabolic process	BP	3	0.00602	0.02348784	ENSG00000143365;ENSG00000171105;ENSG00000186951
GO:0051701	interaction with host	BP	3	0.006216	0.02421354	ENSG00000105976;ENSG00000146648;ENSG00000171105
GO:0001944	vasculature development	BP	5	0.006261	0.02434897	ENSG00000073756;ENSG00000087245;ENSG00000106546;ENSG00000128052;ENSG00000132170
GO:0043536	positive regulation of blood vessel endothelial cell migration	BP	2	0.006299	0.02437961	ENSG00000073756;ENSG00000128052
GO:0045685	regulation of glial cell differentiation	BP	2	0.006299	0.02437961	ENSG00000132170;ENSG00000180210
GO:0045913	positive regulation of carbohydrate metabolic process	BP	2	0.006299	0.02437961	ENSG00000171105;ENSG00000186951
GO:0032412	regulation of ion transmembrane transporter activity	BP	3	0.006349	0.02453324	ENSG00000085563;ENSG00000100985;ENSG00000178623
GO:0051240	positive regulation of multicellular organismal process	BP	8	0.006397	0.0246791	ENSG00000073756;ENSG00000100985;ENSG00000104267;ENSG00000105976;ENSG00000128052;ENSG00000132170;ENSG00000146648;ENSG00000180210
GO:0031100	animal organ regeneration	BP	2	0.006449	0.02483995	ENSG00000132170;ENSG00000146648
GO:0048871	multicellular organismal homeostasis	BP	4	0.006538	0.02514293	ENSG00000073756;ENSG00000104267;ENSG00000105976;ENSG00000146648
GO:0072358	cardiovascular system development	BP	5	0.00655	0.02515002	ENSG00000073756;ENSG00000087245;ENSG00000106546;ENSG00000128052;ENSG00000132170
GO:0007492	endoderm development	BP	2	0.0066	0.02526209	ENSG00000087245;ENSG00000100985
GO:0050918	positive chemotaxis	BP	2	0.0066	0.02526209	ENSG00000105976;ENSG00000128052
GO:2000116	regulation of cysteine-type endopeptidase activity	BP	3	0.006619	0.02529625	ENSG00000073756;ENSG00000100985;ENSG00000132170
GO:0070838	divalent metal ion transport	BP	4	0.00667	0.02544822	ENSG00000073756;ENSG00000170890;ENSG00000178623;ENSG00000180210
GO:0090049	regulation of cell migration involved in sprouting angiogenesis	BP	2	0.006753	0.02572552	ENSG00000073756;ENSG00000128052
GO:0072511	divalent inorganic cation transport	BP	4	0.006849	0.02600724	ENSG00000073756;ENSG00000170890;ENSG00000178623;ENSG00000180210

GO:0080135	regulation of cellular response to stress	BP	5	0.006849	0.02600724	ENSG00000073756;ENSG00000105426;ENSG00000105976;ENSG00000146648;ENSG00000149968
GO:0022898	regulation of transmembrane transporter activity	BP	3	0.006897	0.02606811	ENSG00000085563;ENSG00000100985;ENSG00000178623
GO:0042058	regulation of epidermal growth factor receptor signaling pathway	BP	2	0.006908	0.02606811	ENSG00000100985;ENSG00000146648
GO:0043470	regulation of carbohydrate catabolic process	BP	2	0.006908	0.02606811	ENSG00000171105;ENSG00000186951
GO:0061844	antimicrobial humoral immune response mediated by antimicrobial peptide	BP	2	0.006908	0.02606811	ENSG00000170890;ENSG00000180210
GO:0007611	learning or memory	BP	3	0.007038	0.02639105	ENSG00000073756;ENSG00000146648;ENSG00000171105
GO:0002532	production of molecular mediator involved in inflammatory response	BP	2	0.007064	0.02639105	ENSG00000007171;ENSG00000180210
GO:0090066	regulation of anatomical structure size	BP	4	0.007076	0.02639105	ENSG00000073756;ENSG00000105426;ENSG00000146648;ENSG00000148680
GO:0006955	immune response	BP	10	0.007101	0.02639105	ENSG00000005381;ENSG00000007171;ENSG00000091831;ENSG00000100985;ENSG00000105426;ENSG00000132170;ENSG00000143365;ENSG00000163464;ENSG00000170890;ENSG00000180210
GO:0009617	response to bacterium	BP	5	0.007191	0.02639105	ENSG00000005381;ENSG00000007171;ENSG00000073756;ENSG00000170890;ENSG00000180210
GO:0009058	biosynthetic process	BP	17	0.007208	0.02639105	ENSG00000005381;ENSG00000007171;ENSG00000073756;ENSG00000091831;ENSG00000095303;ENSG00000100823;ENSG00000105976;ENSG00000106546;ENSG00000128052;ENSG00000132170;ENSG00000143365;ENSG00000146648;ENSG00000159228;ENSG00000170890;ENSG00000171105;ENSG00000180210;ENSG00000186951
GO:0034121	regulation of toll-like receptor signaling pathway	BP	2	0.007222	0.02639105	ENSG00000091831;ENSG00000105426
GO:0010750	positive regulation of nitric oxide mediated signal transduction	BP	1	0.007244	0.02639105	ENSG00000146648
GO:0015670	carbon dioxide transport	BP	1	0.007244	0.02639105	ENSG00000104267
GO:0031394	positive regulation of prostaglandin biosynthetic process	BP	1	0.007244	0.02639105	ENSG00000073756
GO:0032847	regulation of cellular pH reduction	BP	1	0.007244	0.02639105	ENSG00000104267
GO:0035442	dipeptide transmembrane transport	BP	1	0.007244	0.02639105	ENSG00000104267
GO:0038033	positive regulation of endothelial cell chemotaxis by VEGF-activated vascular endothelial growth	BP	1	0.007244	0.02639105	ENSG00000128052
GO:0042938	dipeptide transport	BP	1	0.007244	0.02639105	ENSG00000104267
GO:0060751	branch elongation involved in mammary gland duct branching	BP	1	0.007244	0.02639105	ENSG00000091831
GO:0071284	cellular response to lead ion	BP	1	0.007244	0.02639105	ENSG00000073756
GO:0080184	response to phenylpropanoid	BP	1	0.007244	0.02639105	ENSG00000146648

GO:0098501	polynucleotide dephosphorylation	BP	1	0.007244	0.02639105	ENSG00000100823
GO:0098908	regulation of neuronal action potential	BP	1	0.007244	0.02639105	ENSG00000178623
GO:1900738	positive regulation of phospholipase C-activating G protein-coupled receptor signaling pathway	BP	1	0.007244	0.02639105	ENSG00000180210
GO:1902219	negative regulation of intrinsic apoptotic signaling pathway in response to osmotic stress	BP	1	0.007244	0.02639105	ENSG00000073756
GO:1905098	negative regulation of guanyl-nucleotide exchange factor activity	BP	1	0.007244	0.02639105	ENSG00000105976
GO:2000697	negative regulation of epithelial cell differentiation involved in kidney development	BP	1	0.007244	0.02639105	ENSG00000100985
GO:0043406	positive regulation of MAP kinase activity	BP	3	0.007399	0.02691496	ENSG00000146648;ENSG00000170890;ENSG00000171105
GO:0042475	odontogenesis of dentin-containing tooth	BP	2	0.007542	0.0271823	ENSG00000104267;ENSG00000186951
GO:0043154	negative regulation of cysteine-type endopeptidase activity involved in apoptotic process	BP	2	0.007542	0.0271823	ENSG00000073756;ENSG00000100985
GO:0048013	ephrin receptor signaling pathway	BP	2	0.007542	0.0271823	ENSG00000087245;ENSG00000100985
GO:1903035	negative regulation of response to wounding	BP	2	0.007542	0.0271823	ENSG00000105426;ENSG00000180210
GO:2001169	regulation of ATP biosynthetic process	BP	2	0.007542	0.0271823	ENSG00000171105;ENSG00000186951
GO:0009108	coenzyme biosynthetic process	BP	3	0.007546	0.0271823	ENSG00000073756;ENSG00000171105;ENSG00000186951
GO:0022603	regulation of anatomical structure morphogenesis	BP	6	0.007551	0.0271823	ENSG00000073756;ENSG00000091831;ENSG00000105426;ENSG00000128052;ENSG00000132170;ENSG00000180210
GO:0002526	acute inflammatory response	BP	3	0.007621	0.02739189	ENSG00000073756;ENSG00000132170;ENSG00000180210
GO:0032410	negative regulation of transporter activity	BP	2	0.007705	0.02752955	ENSG00000100985;ENSG00000178623
GO:0034103	regulation of tissue remodeling	BP	2	0.007705	0.02752955	ENSG00000104267;ENSG00000146648
GO:0051196	regulation of coenzyme metabolic process	BP	2	0.007705	0.02752955	ENSG00000171105;ENSG00000186951
GO:0070542	response to fatty acid	BP	2	0.007705	0.02752955	ENSG00000073756;ENSG00000132170
GO:0048856	anatomical structure development	BP	16	0.007739	0.02761253	ENSG00000073756;ENSG00000087245;ENSG00000091831;ENSG00000100985;ENSG00000104267;ENSG00000105426;ENSG00000105976;ENSG00000106546;ENSG00000128052;ENSG00000132170;ENSG00000143365;ENSG00000146648;ENSG00000159228;ENSG00000171105;ENSG00000180210;ENSG00000186951
GO:1904063	negative regulation of cation transmembrane transport	BP	2	0.007869	0.0280333	ENSG00000100985;ENSG00000178623
GO:0050790	regulation of catalytic activity	BP	9	0.007891	0.02807201	ENSG00000007171;ENSG00000073756;ENSG00000091831;ENSG00000100985;ENSG00000132170;ENSG00000146648;ENSG00000170890;ENSG00000171105;ENSG00000180210

GO:0046849	bone remodeling	BP	2	0.008035	0.02849784	ENSG00000104267;ENSG00000146648
GO:1901184	regulation of ERBB signaling pathway	BP	2	0.008035	0.02849784	ENSG00000100985;ENSG00000146648
GO:0048708	astrocyte differentiation	BP	2	0.008202	0.02900622	ENSG00000146648;ENSG00000180210
GO:1901992	positive regulation of mitotic cell cycle phase transition	BP	2	0.008202	0.02900622	ENSG00000100823;ENSG00000146648
GO:0043270	positive regulation of ion transport	BP	3	0.008231	0.0290677	ENSG00000085563;ENSG00000170890;ENSG00000180210
GO:0044262	cellular carbohydrate metabolic process	BP	3	0.00831	0.02925872	ENSG00000143365;ENSG00000171105;ENSG00000186951
GO:0045927	positive regulation of growth	BP	3	0.00831	0.02925872	ENSG00000146648;ENSG00000171105;ENSG00000180210
GO:0008152	metabolic process	BP	25	0.008327	0.02927796	ENSG00000005381;ENSG00000007171;ENSG00000073756;ENSG00000087245;ENSG00000091831;ENSG00000095303;ENSG00000100823;ENSG00000100985;ENSG00000105426;ENSG00000105976;ENSG00000106546;ENSG00000118777;ENSG00000128052;ENSG00000132170;ENSG00000133742;ENSG00000143365;ENSG00000146648;ENSG00000149968;ENSG00000159228;ENSG00000163464;ENSG00000170890;ENSG00000171105;ENSG00000180210;ENSG00000186951;ENSG00000198074
GO:0097306	cellular response to alcohol	BP	2	0.008371	0.02931081	ENSG00000106546;ENSG00000132170
GO:1900407	regulation of cellular response to oxidative stress	BP	2	0.008371	0.02931081	ENSG00000105976;ENSG00000149968
GO:0071702	organic substance transport	BP	10	0.008373	0.02931081	ENSG00000007171;ENSG00000073756;ENSG00000085563;ENSG00000104267;ENSG00000132170;ENSG00000133742;ENSG00000146648;ENSG00000170890;ENSG00000171105;ENSG00000186951
GO:0031348	negative regulation of defense response	BP	3	0.008627	0.02960276	ENSG00000132170;ENSG00000180210;ENSG00000186951
GO:0048646	anatomical structure formation involved in morphogenesis	BP	6	0.00866	0.02960276	ENSG00000073756;ENSG00000087245;ENSG00000100985;ENSG00000128052;ENSG00000132170;ENSG00000186951
GO:0001957	intramembranous ossification	BP	1	0.008687	0.02960276	ENSG00000087245
GO:0016115	terpenoid catabolic process	BP	1	0.008687	0.02960276	ENSG00000198074
GO:0036072	direct ossification	BP	1	0.008687	0.02960276	ENSG00000087245
GO:0042373	vitamin K metabolic process	BP	1	0.008687	0.02960276	ENSG00000159228
GO:0051970	negative regulation of transmission of nerve impulse	BP	1	0.008687	0.02960276	ENSG00000178623
GO:0060526	prostate glandular acinus morphogenesis	BP	1	0.008687	0.02960276	ENSG00000091831
GO:0060527	prostate epithelial cord arborization involved in prostate glandular acinus morphogenesis	BP	1	0.008687	0.02960276	ENSG00000091831
GO:0060745	mammary gland branching involved in pregnancy	BP	1	0.008687	0.02960276	ENSG00000091831
GO:0070141	response to UV-A	BP	1	0.008687	0.02960276	ENSG00000146648

GO:0070945	neutrophil mediated killing of gram-negative bacterium	BP	1	0.008687	0.02960276	ENSG00000180210
GO:0110112	regulation of lipid transporter activity	BP	1	0.008687	0.02960276	ENSG00000132170
GO:1900019	regulation of protein kinase C activity	BP	1	0.008687	0.02960276	ENSG00000146648
GO:1900020	positive regulation of protein kinase C activity	BP	1	0.008687	0.02960276	ENSG00000146648
GO:1900736	regulation of phospholipase C-activating G protein-coupled receptor signaling pathway	BP	1	0.008687	0.02960276	ENSG00000180210
GO:1902218	regulation of intrinsic apoptotic signaling pathway in response to osmotic stress	BP	1	0.008687	0.02960276	ENSG00000073756
GO:2001268	negative regulation of cysteine-type endopeptidase activity involved in apoptotic signaling pathway	BP	1	0.008687	0.02960276	ENSG00000100985
GO:0036473	cell death in response to oxidative stress	BP	2	0.008713	0.02960276	ENSG00000105976;ENSG00000149968
GO:0043255	regulation of carbohydrate biosynthetic process	BP	2	0.008713	0.02960276	ENSG00000171105;ENSG00000186951
GO:0044070	regulation of anion transport	BP	2	0.008713	0.02960276	ENSG00000085563;ENSG00000104267
GO:0002521	leukocyte differentiation	BP	4	0.008921	0.03026779	ENSG00000100985;ENSG00000104267;ENSG00000132170;ENSG00000143365
GO:0007275	multicellular organism development	BP	15	0.009013	0.0305349	ENSG00000073756;ENSG00000087245;ENSG00000091831;ENSG00000100985;ENSG00000104267;ENSG00000105426;ENSG00000105976;ENSG00000106546;ENSG00000128052;ENSG00000132170;ENSG00000143365;ENSG00000146648;ENSG00000171105;ENSG00000180210;ENSG00000186951
GO:0051591	response to cAMP	BP	2	0.009062	0.03061537	ENSG00000100823;ENSG00000106546
GO:2000117	negative regulation of cysteine-type endopeptidase activity	BP	2	0.009062	0.03061537	ENSG00000073756;ENSG00000100985
GO:0022411	cellular component disassembly	BP	4	0.009084	0.03064608	ENSG00000087245;ENSG00000100985;ENSG00000149968;ENSG00000171105
GO:0008585	female gonad development	BP	2	0.009239	0.03108198	ENSG00000091831;ENSG00000171105
GO:0032637	interleukin-8 production	BP	2	0.009239	0.03108198	ENSG00000007171;ENSG00000170890
GO:0005996	monosaccharide metabolic process	BP	3	0.009368	0.03142869	ENSG00000143365;ENSG00000171105;ENSG00000186951
GO:0031334	positive regulation of protein complex assembly	BP	3	0.009368	0.03142869	ENSG00000091831;ENSG00000105976;ENSG00000149968
GO:0002009	morphogenesis of an epithelium	BP	4	0.009582	0.03195782	ENSG00000091831;ENSG00000104267;ENSG00000105976;ENSG00000146648
GO:0010817	regulation of hormone levels	BP	4	0.009582	0.03195782	ENSG00000007171;ENSG00000091831;ENSG00000146648;ENSG00000198074
GO:0050900	leukocyte migration	BP	4	0.009582	0.03195782	ENSG00000100985;ENSG00000163464;ENSG00000170890;ENSG00000180210

GO:0002042	cell migration involved in sprouting angiogenesis	BP	2	0.009596	0.03195782	ENSG00000073756;ENSG00000128052
GO:0070301	cellular response to hydrogen peroxide	BP	2	0.009596	0.03195782	ENSG00000100823;ENSG00000105976
GO:0006468	protein phosphorylation	BP	8	0.009605	0.03195782	ENSG00000073756;ENSG00000100985;ENSG00000105976;ENSG00000128052;ENSG00000146648;ENSG00000170890;ENSG00000171105;ENSG00000180210
GO:0019395	fatty acid oxidation	BP	2	0.009778	0.03248941	ENSG00000132170;ENSG00000186951
GO:0016310	phosphorylation	BP	9	0.009813	0.03256316	ENSG00000073756;ENSG00000100985;ENSG00000105976;ENSG00000128052;ENSG00000146648;ENSG00000170890;ENSG00000171105;ENSG00000180210;ENSG00000186951
GO:0034766	negative regulation of ion transmembrane transport	BP	2	0.009961	0.03280027	ENSG00000100985;ENSG00000178623
GO:0035249	synaptic transmission, glutamatergic	BP	2	0.009961	0.03280027	ENSG00000073756;ENSG00000146648
GO:0044089	positive regulation of cellular component biogenesis	BP	4	0.009981	0.03280027	ENSG00000091831;ENSG00000105976;ENSG00000128052;ENSG00000149968
GO:0031017	exocrine pancreas development	BP	1	0.010128	0.03280027	ENSG00000171105
GO:0034164	negative regulation of toll-like receptor 9 signaling pathway	BP	1	0.010128	0.03280027	ENSG00000105426
GO:0038089	positive regulation of cell migration by vascular endothelial growth factor signaling pathway	BP	1	0.010128	0.03280027	ENSG00000128052
GO:0060331	negative regulation of response to interferon-gamma	BP	1	0.010128	0.03280027	ENSG00000132170
GO:0060336	negative regulation of interferon-gamma-mediated signaling pathway	BP	1	0.010128	0.03280027	ENSG00000132170
GO:0060687	regulation of branching involved in prostate gland morphogenesis	BP	1	0.010128	0.03280027	ENSG00000091831
GO:0071455	cellular response to hyperoxia	BP	1	0.010128	0.03280027	ENSG00000132170
GO:1901299	negative regulation of hydrogen peroxide-mediated programmed cell death	BP	1	0.010128	0.03280027	ENSG00000105976
GO:1905097	regulation of guanyl-nucleotide exchange factor activity	BP	1	0.010128	0.03280027	ENSG00000105976
GO:2001280	positive regulation of unsaturated fatty acid biosynthetic process	BP	1	0.010128	0.03280027	ENSG00000073756
GO:0032091	negative regulation of protein binding	BP	2	0.010145	0.03280027	ENSG00000105976;ENSG00000186951
GO:0034440	lipid oxidation	BP	2	0.010145	0.03280027	ENSG00000132170;ENSG00000186951
GO:0046545	development of primary female sexual characteristics	BP	2	0.010145	0.03280027	ENSG00000091831;ENSG00000171105
GO:0070098	chemokine-mediated signaling pathway	BP	2	0.010145	0.03280027	ENSG00000163464;ENSG00000178623

GO:1902882	regulation of response to oxidative stress	BP	2	0.010145	0.03280027	ENSG00000105976;ENSG00000149968
GO:0007507	heart development	BP	4	0.010156	0.03280027	ENSG00000132170;ENSG00000146648;ENSG00000171105;ENSG00000186951
GO:1902532	negative regulation of intracellular signal transduction	BP	4	0.010156	0.03280027	ENSG00000073756;ENSG00000091831;ENSG00000100985;ENSG00000105976
GO:0010522	regulation of calcium ion transport into cytosol	BP	2	0.010331	0.03331987	ENSG00000170890;ENSG00000180210
GO:0001775	cell activation	BP	7	0.010369	0.03339757	ENSG00000005381;ENSG00000100985;ENSG00000106546;ENSG00000143365;ENSG00000146648;ENSG00000163464;ENSG00000180210
GO:0031329	regulation of cellular catabolic process	BP	5	0.010383	0.03340005	ENSG00000100823;ENSG00000105976;ENSG00000128052;ENSG00000171105;ENSG00000186951
GO:0090068	positive regulation of cell cycle process	BP	3	0.010411	0.03344649	ENSG00000100823;ENSG00000146648;ENSG00000171105
GO:0006164	purine nucleotide biosynthetic process	BP	3	0.010501	0.03369065	ENSG00000007171;ENSG00000171105;ENSG00000186951
GO:2001243	negative regulation of intrinsic apoptotic signaling pathway	BP	2	0.010518	0.0336995	ENSG00000073756;ENSG00000100985
GO:0051091	positive regulation of DNA-binding transcription factor activity	BP	3	0.010592	0.03389073	ENSG00000091831;ENSG00000132170;ENSG00000170890
GO:0043410	positive regulation of MAPK cascade	BP	4	0.010631	0.03397246	ENSG00000128052;ENSG00000146648;ENSG00000170890;ENSG00000171105
GO:0018193	peptidyl-amino acid modification	BP	6	0.010652	0.03399228	ENSG00000007171;ENSG00000073756;ENSG00000105976;ENSG00000128052;ENSG00000146648;ENSG00000171105
GO:1903426	regulation of reactive oxygen species biosynthetic process	BP	2	0.010707	0.03412383	ENSG00000073756;ENSG00000171105
GO:1904659	glucose transmembrane transport	BP	2	0.010897	0.03468487	ENSG00000170890;ENSG00000171105
GO:0006897	endocytosis	BP	5	0.011029	0.03505661	ENSG00000105976;ENSG00000132170;ENSG00000146648;ENSG00000163464;ENSG00000171105
GO:1901989	positive regulation of cell cycle phase transition	BP	2	0.011089	0.03520294	ENSG00000100823;ENSG00000146648
GO:0033674	positive regulation of kinase activity	BP	4	0.011121	0.03525762	ENSG00000146648;ENSG00000170890;ENSG00000171105;ENSG00000180210
GO:0042063	gliogenesis	BP	3	0.011237	0.03555964	ENSG00000132170;ENSG00000146648;ENSG00000180210
GO:0001525	angiogenesis	BP	4	0.011246	0.03555964	ENSG00000073756;ENSG00000087245;ENSG00000128052;ENSG00000132170
GO:0006939	smooth muscle contraction	BP	2	0.011283	0.03562977	ENSG00000073756;ENSG00000148680
GO:0001816	cytokine production	BP	5	0.011361	0.03583232	ENSG00000007171;ENSG00000073756;ENSG00000105426;ENSG00000170890;ENSG00000180210
GO:0050890	cognition	BP	3	0.011426	0.03596266	ENSG00000073756;ENSG00000146648;ENSG00000171105

GO:0001547	antral ovarian follicle growth	BP	1	0.011566	0.03596266	ENSG00000091831
GO:0001955	blood vessel maturation	BP	1	0.011566	0.03596266	ENSG00000087245
GO:0008627	intrinsic apoptotic signaling pathway in response to osmotic stress	BP	1	0.011566	0.03596266	ENSG00000073756
GO:0009750	response to fructose	BP	1	0.011566	0.03596266	ENSG00000073756
GO:0016093	polyprenol metabolic process	BP	1	0.011566	0.03596266	ENSG00000198074
GO:0035095	behavioral response to nicotine	BP	1	0.011566	0.03596266	ENSG00000186951
GO:0051715	cytolysis in other organism	BP	1	0.011566	0.03596266	ENSG00000180210
GO:0070459	prolactin secretion	BP	1	0.011566	0.03596266	ENSG00000146648
GO:0110095	cellular detoxification of aldehyde	BP	1	0.011566	0.03596266	ENSG00000198074
GO:1903961	positive regulation of anion transmembrane transport	BP	1	0.011566	0.03596266	ENSG00000085563
GO:0072522	purine-containing compound biosynthetic process	BP	3	0.011616	0.03607113	ENSG00000007171;ENSG00000171105;ENSG00000186951
GO:0033138	positive regulation of peptidyl-serine phosphorylation	BP	2	0.011674	0.03615718	ENSG00000073756;ENSG00000146648
GO:1903578	regulation of ATP metabolic process	BP	2	0.011674	0.03615718	ENSG00000171105;ENSG00000186951
GO:1903507	negative regulation of nucleic acid-templated transcription	BP	6	0.011823	0.03657401	ENSG00000091831;ENSG00000100823;ENSG00000106546;ENSG00000132170;ENSG00000143365;ENSG00000186951
GO:0006096	glycolytic process	BP	2	0.011872	0.03658257	ENSG00000171105;ENSG00000186951
GO:1990868	response to chemokine	BP	2	0.011872	0.03658257	ENSG00000163464;ENSG00000178623
GO:1990869	cellular response to chemokine	BP	2	0.011872	0.03658257	ENSG00000163464;ENSG00000178623
GO:1902679	negative regulation of RNA biosynthetic process	BP	6	0.011905	0.03663804	ENSG00000091831;ENSG00000100823;ENSG00000106546;ENSG00000132170;ENSG00000143365;ENSG00000186951
GO:0006757	ATP generation from ADP	BP	2	0.012071	0.03696134	ENSG00000171105;ENSG00000186951
GO:0008645	hexose transmembrane transport	BP	2	0.012071	0.03696134	ENSG00000170890;ENSG00000171105
GO:0048640	negative regulation of developmental growth	BP	2	0.012071	0.03696134	ENSG00000105426;ENSG00000186951
GO:0050829	defense response to Gram-negative bacterium	BP	2	0.012071	0.03696134	ENSG00000007171;ENSG00000180210

GO:0016101	diterpenoid metabolic process	BP	2	0.012272	0.03749397	ENSG00000146648;ENSG00000198074
GO:0007610	behavior	BP	4	0.012276	0.03749397	ENSG00000073756;ENSG00000146648;ENSG00000171105;ENSG00000186951
GO:0006821	chloride transport	BP	2	0.012474	0.03790758	ENSG00000085563;ENSG00000104267
GO:0015749	monosaccharide transmembrane transport	BP	2	0.012474	0.03790758	ENSG00000170890;ENSG00000171105
GO:0042303	molting cycle	BP	2	0.012474	0.03790758	ENSG00000073756;ENSG00000146648
GO:0042633	hair cycle	BP	2	0.012474	0.03790758	ENSG00000073756;ENSG00000146648
GO:0042866	pyruvate biosynthetic process	BP	2	0.012678	0.03847835	ENSG00000171105;ENSG00000186951
GO:0044057	regulation of system process	BP	4	0.012745	0.03863472	ENSG00000073756;ENSG00000146648;ENSG00000178623;ENSG00000186951
GO:0071704	organic substance metabolic process	BP	24	0.012785	0.03868914	ENSG00000005381;ENSG00000007171;ENSG00000073756;ENSG00000087245;ENSG00000091831;ENSG00000095303;ENSG00000100823;ENSG00000100985;ENSG00000105426;ENSG00000105976;ENSG00000106546;ENSG00000118777;ENSG00000128052;ENSG00000132170;ENSG00000143365;ENSG00000146648;ENSG00000149968;ENSG00000159228;ENSG00000163464;ENSG00000170890;ENSG00000171105;ENSG00000180210;ENSG00000186951;ENSG00000198074
GO:0034219	carbohydrate transmembrane transport	BP	2	0.012883	0.03868914	ENSG00000170890;ENSG00000171105
GO:0043200	response to amino acid	BP	2	0.012883	0.03868914	ENSG00000087245;ENSG00000146648
GO:0030097	hemopoiesis	BP	5	0.012972	0.03868914	ENSG00000100985;ENSG00000104267;ENSG00000128052;ENSG00000132170;ENSG00000143365
GO:0008300	isoprenoid catabolic process	BP	1	0.013003	0.03868914	ENSG00000198074
GO:0010359	regulation of anion channel activity	BP	1	0.013003	0.03868914	ENSG00000085563
GO:0010727	negative regulation of hydrogen peroxide metabolic process	BP	1	0.013003	0.03868914	ENSG00000149968
GO:0030638	polyketide metabolic process	BP	1	0.013003	0.03868914	ENSG00000198074
GO:0032687	negative regulation of interferon-alpha production	BP	1	0.013003	0.03868914	ENSG00000105426
GO:0042908	xenobiotic transport	BP	1	0.013003	0.03868914	ENSG00000118777
GO:0044597	daunorubicin metabolic process	BP	1	0.013003	0.03868914	ENSG00000198074
GO:0044598	doxorubicin metabolic process	BP	1	0.013003	0.03868914	ENSG00000198074
GO:0060267	positive regulation of respiratory burst	BP	1	0.013003	0.03868914	ENSG00000171105
GO:0070944	neutrophil mediated killing of bacterium	BP	1	0.013003	0.03868914	ENSG00000180210

GO:1900122	positive regulation of receptor binding	BP	1	0.013003	0.03868914	ENSG00000100985
GO:0046660	female sex differentiation	BP	2	0.013089	0.03889861	ENSG00000091831;ENSG00000171105
GO:0007173	epidermal growth factor receptor signaling pathway	BP	2	0.013298	0.03942016	ENSG00000100985;ENSG00000146648
GO:0030593	neutrophil chemotaxis	BP	2	0.013298	0.03942016	ENSG00000163464;ENSG00000170890
GO:0071496	cellular response to external stimulus	BP	3	0.013413	0.03966424	ENSG00000073756;ENSG00000132170;ENSG00000146648
GO:1903829	positive regulation of cellular protein localization	BP	3	0.013413	0.03966424	ENSG00000073756;ENSG00000146648;ENSG00000180210
GO:1900180	regulation of protein localization to nucleus	BP	2	0.013507	0.03989468	ENSG00000073756;ENSG00000180210
GO:0001655	urogenital system development	BP	3	0.013622	0.04013485	ENSG00000091831;ENSG00000100985;ENSG00000104267
GO:1904062	regulation of cation transmembrane transport	BP	3	0.013622	0.04013485	ENSG00000100985;ENSG00000178623;ENSG00000180210
GO:0072521	purine-containing compound metabolic process	BP	4	0.013647	0.0401614	ENSG00000007171;ENSG00000118777;ENSG00000171105;ENSG00000186951
GO:0046677	response to antibiotic	BP	3	0.013727	0.04034641	ENSG00000100823;ENSG00000105976;ENSG00000106546
GO:0043066	negative regulation of apoptotic process	BP	5	0.013889	0.04072131	ENSG00000005381;ENSG00000073756;ENSG00000100985;ENSG00000128052;ENSG00000146648
GO:0007613	memory	BP	2	0.013931	0.04072131	ENSG00000073756;ENSG00000171105
GO:0022612	gland morphogenesis	BP	2	0.013931	0.04072131	ENSG00000091831;ENSG00000146648
GO:0046031	ADP metabolic process	BP	2	0.013931	0.04072131	ENSG00000171105;ENSG00000186951
GO:0051188	cofactor biosynthetic process	BP	3	0.013939	0.04072131	ENSG00000073756;ENSG00000171105;ENSG00000186951
GO:0071902	positive regulation of protein serine/threonine kinase activity	BP	3	0.014045	0.04098314	ENSG00000146648;ENSG00000170890;ENSG00000171105
GO:0031349	positive regulation of defense response	BP	4	0.014222	0.04133103	ENSG00000073756;ENSG00000091831;ENSG00000105426;ENSG00000146648
GO:0032147	activation of protein kinase activity	BP	3	0.014368	0.04133103	ENSG00000146648;ENSG00000170890;ENSG00000171105
GO:0010749	regulation of nitric oxide mediated signal transduction	BP	1	0.014438	0.04133103	ENSG00000146648
GO:0030647	aminoglycoside antibiotic metabolic process	BP	1	0.014438	0.04133103	ENSG00000198074
GO:0031284	positive regulation of guanylate cyclase activity	BP	1	0.014438	0.04133103	ENSG00000007171

GO:0032000	positive regulation of fatty acid beta-oxidation	BP	1	0.014438	0.04133103	ENSG00000186951
GO:0048671	negative regulation of collateral sprouting	BP	1	0.014438	0.04133103	ENSG00000105426
GO:0051901	positive regulation of mitochondrial depolarization	BP	1	0.014438	0.04133103	ENSG00000128052
GO:0070943	neutrophil mediated killing of symbiont cell	BP	1	0.014438	0.04133103	ENSG00000180210
GO:0090271	positive regulation of fibroblast growth factor production	BP	1	0.014438	0.04133103	ENSG00000073756
GO:0106049	regulation of cellular response to osmotic stress	BP	1	0.014438	0.04133103	ENSG00000073756
GO:0110096	cellular response to aldehyde	BP	1	0.014438	0.04133103	ENSG00000198074
GO:1903799	negative regulation of production of miRNAs involved in gene silencing by miRNA	BP	1	0.014438	0.04133103	ENSG00000091831
GO:1903800	positive regulation of production of miRNAs involved in gene silencing by miRNA	BP	1	0.014438	0.04133103	ENSG00000146648
GO:1990535	neuron projection maintenance	BP	1	0.014438	0.04133103	ENSG00000171105
GO:2001214	positive regulation of vasculogenesis	BP	1	0.014438	0.04133103	ENSG00000128052
GO:0010721	negative regulation of cell development	BP	3	0.014476	0.04139181	ENSG00000105426;ENSG00000180210;ENSG00000186951
GO:0048638	regulation of developmental growth	BP	3	0.014585	0.04160486	ENSG00000105426;ENSG00000171105;ENSG00000186951
GO:0051235	maintenance of location	BP	3	0.014585	0.04160486	ENSG00000132170;ENSG00000180210;ENSG00000186951
GO:0044087	regulation of cellular component biogenesis	BP	5	0.014619	0.04165236	ENSG00000091831;ENSG00000105426;ENSG00000105976;ENSG00000128052;ENSG00000149968
GO:0010822	positive regulation of mitochondrion organization	BP	2	0.014795	0.04205417	ENSG00000100985;ENSG00000128052
GO:0051817	modification of morphology or physiology of other organism involved in symbiotic interaction	BP	2	0.014795	0.04205417	ENSG00000171105;ENSG00000180210
GO:0051336	regulation of hydrolase activity	BP	6	0.014962	0.04247862	ENSG00000073756;ENSG00000091831;ENSG00000100985;ENSG00000132170;ENSG00000146648;ENSG00000170890
GO:0006721	terpenoid metabolic process	BP	2	0.015014	0.04247862	ENSG00000146648;ENSG00000198074
GO:0032368	regulation of lipid transport	BP	2	0.015014	0.04247862	ENSG00000132170;ENSG00000186951
GO:0046717	acid secretion	BP	2	0.015014	0.04247862	ENSG00000007171;ENSG00000170890
GO:0043405	regulation of MAP kinase activity	BP	3	0.015137	0.04277481	ENSG00000146648;ENSG00000170890;ENSG00000171105

GO:0034763	negative regulation of transmembrane transport	BP	2	0.015235	0.04300323	ENSG00000100985;ENSG00000178623
GO:0048534	hematopoietic or lymphoid organ development	BP	5	0.015257	0.04301387	ENSG00000100985;ENSG00000104267;ENSG00000128052;ENSG00000132170;ENSG00000143365
GO:0042476	odontogenesis	BP	2	0.015458	0.04352937	ENSG00000104267;ENSG00000186951
GO:0007568	aging	BP	3	0.015586	0.04377456	ENSG00000005381;ENSG00000073756;ENSG00000100823
GO:0051101	regulation of DNA binding	BP	2	0.015681	0.04377456	ENSG00000100985;ENSG00000132170
GO:0051928	positive regulation of calcium ion transport	BP	2	0.015681	0.04377456	ENSG00000170890;ENSG00000180210
GO:0046903	secretion	BP	7	0.015742	0.04377456	ENSG00000005381;ENSG00000007171;ENSG00000100985;ENSG00000104267;ENSG00000146648;ENSG00000163464;ENSG00000170890
GO:0031323	regulation of cellular metabolic process	BP	16	0.015767	0.04377456	ENSG00000007171;ENSG00000073756;ENSG00000091831;ENSG00000100823;ENSG00000100985;ENSG00000105976;ENSG00000106546;ENSG00000128052;ENSG00000132170;ENSG00000143365;ENSG00000146648;ENSG00000149968;ENSG00000170890;ENSG00000171105;ENSG00000180210;ENSG00000186951
GO:0016042	lipid catabolic process	BP	3	0.015814	0.04377456	ENSG00000170890;ENSG00000186951;ENSG00000198074
GO:0051338	regulation of transferase activity	BP	5	0.015853	0.04377456	ENSG00000132170;ENSG00000146648;ENSG00000170890;ENSG00000171105;ENSG00000180210
GO:0010960	magnesium ion homeostasis	BP	1	0.01587	0.04377456	ENSG00000146648
GO:0031392	regulation of prostaglandin biosynthetic process	BP	1	0.01587	0.04377456	ENSG00000073756
GO:0031915	positive regulation of synaptic plasticity	BP	1	0.01587	0.04377456	ENSG00000073756
GO:0035627	ceramide transport	BP	1	0.01587	0.04377456	ENSG00000085563
GO:0035672	oligopeptide transmembrane transport	BP	1	0.01587	0.04377456	ENSG00000104267
GO:0045713	low-density lipoprotein particle receptor biosynthetic process	BP	1	0.01587	0.04377456	ENSG00000132170
GO:0051918	negative regulation of fibrinolysis	BP	1	0.01587	0.04377456	ENSG00000180210
GO:0060068	vagina development	BP	1	0.01587	0.04377456	ENSG00000091831
GO:0060346	bone trabecula formation	BP	1	0.01587	0.04377456	ENSG00000087245
GO:0060525	prostate glandular acinus development	BP	1	0.01587	0.04377456	ENSG00000091831
GO:0060736	prostate gland growth	BP	1	0.01587	0.04377456	ENSG00000091831
GO:0006165	nucleoside diphosphate phosphorylation	BP	2	0.015907	0.04382441	ENSG00000171105;ENSG00000186951

GO:0000165	MAPK cascade	BP	5	0.015974	0.04395997	ENSG00000105976;ENSG00000128052;ENSG00000146648;ENSG00000170890;ENSG00000171105
GO:0051253	negative regulation of RNA metabolic process	BP	6	0.016044	0.04410153	ENSG00000091831;ENSG00000100823;ENSG00000106546;ENSG00000132170;ENSG00000143365;ENSG00000186951
GO:0021987	cerebral cortex development	BP	2	0.016133	0.04429735	ENSG00000105426;ENSG00000146648
GO:0010638	positive regulation of organelle organization	BP	4	0.016277	0.04464276	ENSG00000100985;ENSG00000105976;ENSG00000128052;ENSG00000171105
GO:0046939	nucleotide phosphorylation	BP	2	0.016361	0.04477088	ENSG00000171105;ENSG00000186951
GO:1990266	neutrophil migration	BP	2	0.016361	0.04477088	ENSG00000163464;ENSG00000170890
GO:0009135	purine nucleoside diphosphate metabolic process	BP	2	0.01659	0.04529607	ENSG00000171105;ENSG00000186951
GO:0009179	purine ribonucleoside diphosphate metabolic process	BP	2	0.01659	0.04529607	ENSG00000171105;ENSG00000186951
GO:0023014	signal transduction by protein phosphorylation	BP	5	0.016651	0.04538014	ENSG00000105976;ENSG00000128052;ENSG00000146648;ENSG00000170890;ENSG00000171105
GO:0006935	chemotaxis	BP	4	0.016678	0.04538014	ENSG00000105976;ENSG00000128052;ENSG00000163464;ENSG00000170890
GO:0051347	positive regulation of transferase activity	BP	4	0.016678	0.04538014	ENSG00000146648;ENSG00000170890;ENSG00000171105;ENSG00000180210
GO:0032496	response to lipopolysaccharide	BP	3	0.016744	0.04550927	ENSG00000005381;ENSG00000007171;ENSG00000073756
GO:0001704	formation of primary germ layer	BP	2	0.016821	0.04563904	ENSG00000087245;ENSG00000100985
GO:0042330	taxis	BP	4	0.016921	0.04563904	ENSG00000105976;ENSG00000128052;ENSG00000163464;ENSG00000170890
GO:0009185	ribonucleoside diphosphate metabolic process	BP	2	0.017053	0.04563904	ENSG00000171105;ENSG00000186951
GO:0009790	embryo development	BP	5	0.017156	0.04563904	ENSG00000087245;ENSG00000100985;ENSG00000128052;ENSG00000146648;ENSG00000171105
GO:0048589	developmental growth	BP	4	0.017166	0.04563904	ENSG00000091831;ENSG00000105426;ENSG00000171105;ENSG00000186951
GO:0002674	negative regulation of acute inflammatory response	BP	1	0.017301	0.04563904	ENSG00000132170
GO:0006857	oligopeptide transport	BP	1	0.017301	0.04563904	ENSG00000104267
GO:0031282	regulation of guanylate cyclase activity	BP	1	0.017301	0.04563904	ENSG00000007171
GO:0033197	response to vitamin E	BP	1	0.017301	0.04563904	ENSG00000132170
GO:0045899	positive regulation of RNA polymerase II transcriptional preinitiation complex assembly	BP	1	0.017301	0.04563904	ENSG00000091831

GO:0051549	positive regulation of keratinocyte migration	BP	1	0.017301	0.04563904	ENSG00000100985
GO:0051712	positive regulation of killing of cells of other organism	BP	1	0.017301	0.04563904	ENSG00000007171
GO:0051974	negative regulation of telomerase activity	BP	1	0.017301	0.04563904	ENSG00000132170
GO:0060100	positive regulation of phagocytosis, engulfment	BP	1	0.017301	0.04563904	ENSG00000132170
GO:0060442	branching involved in prostate gland morphogenesis	BP	1	0.017301	0.04563904	ENSG00000091831
GO:0070493	thrombin-activated receptor signaling pathway	BP	1	0.017301	0.04563904	ENSG00000105976
GO:0070942	neutrophil mediated cytotoxicity	BP	1	0.017301	0.04563904	ENSG00000180210
GO:0090336	positive regulation of brown fat cell differentiation	BP	1	0.017301	0.04563904	ENSG00000073756
GO:0090557	establishment of endothelial intestinal barrier	BP	1	0.017301	0.04563904	ENSG00000105426
GO:0099151	regulation of postsynaptic density assembly	BP	1	0.017301	0.04563904	ENSG00000105426
GO:1900402	regulation of carbohydrate metabolic process by regulation of transcription from RNA polymerase II	BP	1	0.017301	0.04563904	ENSG00000186951
GO:1901298	regulation of hydrogen peroxide-mediated programmed cell death	BP	1	0.017301	0.04563904	ENSG00000105976
GO:1904181	positive regulation of membrane depolarization	BP	1	0.017301	0.04563904	ENSG00000128052
GO:1904321	response to forskolin	BP	1	0.017301	0.04563904	ENSG00000106546
GO:1904322	cellular response to forskolin	BP	1	0.017301	0.04563904	ENSG00000106546
GO:1905155	positive regulation of membrane invagination	BP	1	0.017301	0.04563904	ENSG00000132170
GO:1905461	positive regulation of vascular associated smooth muscle cell apoptotic process	BP	1	0.017301	0.04563904	ENSG00000132170
GO:0001505	regulation of neurotransmitter levels	BP	3	0.01734	0.04569313	ENSG00000007171;ENSG00000073756;ENSG00000171105
GO:0098542	defense response to other organism	BP	4	0.017497	0.04605455	ENSG00000005381;ENSG00000007171;ENSG00000170890;ENSG00000180210
GO:0071621	granulocyte chemotaxis	BP	2	0.017522	0.04607079	ENSG00000163464;ENSG00000170890
GO:0098657	import into cell	BP	5	0.017606	0.04624099	ENSG00000105976;ENSG00000132170;ENSG00000146648;ENSG00000163464;ENSG00000171105
GO:0000902	cell morphogenesis	BP	5	0.017671	0.04636115	ENSG00000105426;ENSG00000105976;ENSG00000128052;ENSG00000146648;ENSG00000180210

GO:0035195	gene silencing by miRNA	BP	3	0.017704	0.04639862	ENSG00000091831;ENSG00000132170;ENSG00000146648
GO:0008584	male gonad development	BP	2	0.017996	0.04706099	ENSG00000091831;ENSG00000171105
GO:0034754	cellular hormone metabolic process	BP	2	0.017996	0.04706099	ENSG00000091831;ENSG00000198074
GO:0050678	regulation of epithelial cell proliferation	BP	3	0.018072	0.04720943	ENSG00000128052;ENSG00000132170;ENSG00000146648
GO:0016192	vesicle-mediated transport	BP	8	0.018145	0.04734913	ENSG00000005381;ENSG00000100985;ENSG00000105976;ENSG00000132170;ENSG00000146648;ENSG00000163464;ENSG00000171105;ENSG00000180210
GO:0046546	development of primary male sexual characteristics	BP	2	0.018235	0.04753179	ENSG00000091831;ENSG00000171105
GO:0048514	blood vessel morphogenesis	BP	4	0.018426	0.04759123	ENSG00000073756;ENSG00000087245;ENSG00000128052;ENSG00000132170
GO:0038127	ERBB signaling pathway	BP	2	0.018475	0.04759123	ENSG00000100985;ENSG00000146648
GO:0002520	immune system development	BP	5	0.018596	0.04759123	ENSG00000100985;ENSG00000104267;ENSG00000128052;ENSG00000132170;ENSG00000143365
GO:0009411	response to UV	BP	2	0.018717	0.04759123	ENSG00000073756;ENSG00000146648
GO:1903670	regulation of sprouting angiogenesis	BP	2	0.018717	0.04759123	ENSG00000073756;ENSG00000128052
GO:0002679	respiratory burst involved in defense response	BP	1	0.01873	0.04759123	ENSG00000005381
GO:0007494	midgut development	BP	1	0.01873	0.04759123	ENSG00000146648
GO:0031622	positive regulation of fever generation	BP	1	0.01873	0.04759123	ENSG00000073756
GO:0032096	negative regulation of response to food	BP	1	0.01873	0.04759123	ENSG00000186951
GO:0032099	negative regulation of appetite	BP	1	0.01873	0.04759123	ENSG00000186951
GO:0034163	regulation of toll-like receptor 9 signaling pathway	BP	1	0.01873	0.04759123	ENSG00000105426
GO:0043247	telomere maintenance in response to DNA damage	BP	1	0.01873	0.04759123	ENSG00000100823
GO:0045759	negative regulation of action potential	BP	1	0.01873	0.04759123	ENSG00000178623
GO:0045820	negative regulation of glycolytic process	BP	1	0.01873	0.04759123	ENSG00000186951
GO:0046415	urate metabolic process	BP	1	0.01873	0.04759123	ENSG00000118777
GO:0048681	negative regulation of axon regeneration	BP	1	0.01873	0.04759123	ENSG00000105426

GO:0050872	white fat cell differentiation	BP	1	0.01873	0.04759123	ENSG00000132170
GO:0051770	positive regulation of nitric-oxide synthase biosynthetic process	BP	1	0.01873	0.04759123	ENSG00000128052
GO:0061000	negative regulation of dendritic spine development	BP	1	0.01873	0.04759123	ENSG00000105426
GO:0061029	eyelid development in camera-type eye	BP	1	0.01873	0.04759123	ENSG00000146648
GO:0061052	negative regulation of cell growth involved in cardiac muscle cell development	BP	1	0.01873	0.04759123	ENSG00000186951
GO:1904424	regulation of GTP binding	BP	1	0.01873	0.04759123	ENSG00000105976
GO:2000194	regulation of female gonad development	BP	1	0.01873	0.04759123	ENSG00000171105
GO:2001279	regulation of unsaturated fatty acid biosynthetic process	BP	1	0.01873	0.04759123	ENSG00000073756
GO:0035194	posttranscriptional gene silencing by RNA	BP	3	0.018821	0.0477724	ENSG00000091831;ENSG00000132170;ENSG00000146648
GO:0016441	posttranscriptional gene silencing	BP	3	0.018947	0.04802448	ENSG00000091831;ENSG00000132170;ENSG00000146648
GO:0014013	regulation of gliogenesis	BP	2	0.01896	0.04802448	ENSG00000132170;ENSG00000180210
GO:0002237	response to molecule of bacterial origin	BP	3	0.019074	0.04821323	ENSG00000005381;ENSG00000007171;ENSG00000073756
GO:0031331	positive regulation of cellular catabolic process	BP	3	0.019074	0.04821323	ENSG00000128052;ENSG00000171105;ENSG00000186951
GO:0033135	regulation of peptidyl-serine phosphorylation	BP	2	0.01945	0.04897833	ENSG00000073756;ENSG00000146648
GO:0042542	response to hydrogen peroxide	BP	2	0.01945	0.04897833	ENSG00000100823;ENSG00000105976
GO:0050921	positive regulation of chemotaxis	BP	2	0.01945	0.04897833	ENSG00000105976;ENSG00000128052
GO:0045765	regulation of angiogenesis	BP	3	0.019458	0.04897833	ENSG00000073756;ENSG00000128052;ENSG00000132170
GO:0001889	liver development	BP	2	0.019697	0.04934707	ENSG00000105976;ENSG00000146648
GO:0007612	learning	BP	2	0.019697	0.04934707	ENSG00000073756;ENSG00000171105
GO:0042177	negative regulation of protein catabolic process	BP	2	0.019697	0.04934707	ENSG00000007171;ENSG00000146648
GO:0006720	isoprenoid metabolic process	BP	2	0.019945	0.04934707	ENSG00000146648;ENSG00000198074
GO:0035023	regulation of Rho protein signal transduction	BP	2	0.019945	0.04934707	ENSG00000105976;ENSG00000178623

GO:2000241	regulation of reproductive process	BP	2	0.019945	0.04934707	ENSG00000091831;ENSG00000171105
GO:0001886	endothelial cell morphogenesis	BP	1	0.020156	0.04934707	ENSG00000105976
GO:0010421	hydrogen peroxide-mediated programmed cell death	BP	1	0.020156	0.04934707	ENSG00000105976
GO:0019755	one-carbon compound transport	BP	1	0.020156	0.04934707	ENSG00000104267
GO:0031620	regulation of fever generation	BP	1	0.020156	0.04934707	ENSG00000073756
GO:0032230	positive regulation of synaptic transmission, GABAergic	BP	1	0.020156	0.04934707	ENSG00000104267
GO:0034374	low-density lipoprotein particle remodeling	BP	1	0.020156	0.04934707	ENSG00000005381
GO:0036295	cellular response to increased oxygen levels	BP	1	0.020156	0.04934707	ENSG00000132170
GO:0038166	angiotensin-activated signaling pathway	BP	1	0.020156	0.04934707	ENSG00000104267
GO:0044130	negative regulation of growth of symbiont in host	BP	1	0.020156	0.04934707	ENSG00000005381
GO:0048712	negative regulation of astrocyte differentiation	BP	1	0.020156	0.04934707	ENSG00000180210
GO:0051547	regulation of keratinocyte migration	BP	1	0.020156	0.04934707	ENSG00000100985
GO:0051873	killing by host of symbiont cells	BP	1	0.020156	0.04934707	ENSG00000180210
GO:0060099	regulation of phagocytosis, engulfment	BP	1	0.020156	0.04934707	ENSG00000132170
GO:0061430	bone trabecula morphogenesis	BP	1	0.020156	0.04934707	ENSG00000087245
GO:0061478	response to platelet aggregation inhibitor	BP	1	0.020156	0.04934707	ENSG00000106546
GO:0090269	fibroblast growth factor production	BP	1	0.020156	0.04934707	ENSG00000073756
GO:0090270	regulation of fibroblast growth factor production	BP	1	0.020156	0.04934707	ENSG00000073756
GO:0097468	programmed cell death in response to reactive oxygen species	BP	1	0.020156	0.04934707	ENSG00000105976
GO:0099150	regulation of postsynaptic specialization assembly	BP	1	0.020156	0.04934707	ENSG00000105426
GO:0150052	regulation of postsynapse assembly	BP	1	0.020156	0.04934707	ENSG00000105426
GO:2001212	regulation of vasculogenesis	BP	1	0.020156	0.04934707	ENSG00000128052

GO:0032880	regulation of protein localization	BP	5	0.020187	0.04934707	ENSG00000007171;ENSG00000073756;ENSG00000146648;ENSG00000170890;ENSG00000180210
GO:0048729	tissue morphogenesis	BP	4	0.020196	0.04934707	ENSG00000091831;ENSG00000104267;ENSG00000105976;ENSG00000146648
GO:0045861	negative regulation of proteolysis	BP	3	0.020238	0.04940028	ENSG00000073756;ENSG00000100985;ENSG00000180210
GO:0008643	carbohydrate transport	BP	2	0.020446	0.0497571	ENSG00000170890;ENSG00000171105
GO:0019730	antimicrobial humoral response	BP	2	0.020446	0.0497571	ENSG00000170890;ENSG00000180210
GO:0061008	hepaticobiliary system development	BP	2	0.020446	0.0497571	ENSG00000105976;ENSG00000146648
GO:0009611	response to wounding	BP	4	0.020562	0.04998933	ENSG00000105426;ENSG00000146648;ENSG00000180210;ENSG00000186951
GO:0055123	digestive system development	BP	2	0.020698	0.05027005	ENSG00000146648;ENSG00000171105
GO:0006090	pyruvate metabolic process	BP	2	0.021207	0.0511804	ENSG00000171105;ENSG00000186951
GO:1903169	regulation of calcium ion transmembrane transport	BP	2	0.021207	0.0511804	ENSG00000178623;ENSG00000180210
GO:0006898	receptor-mediated endocytosis	BP	3	0.021305	0.0511804	ENSG00000146648;ENSG00000163464;ENSG00000171105
GO:0031399	regulation of protein modification process	BP	7	0.021423	0.0511804	ENSG00000073756;ENSG00000100985;ENSG00000128052;ENSG00000146648;ENSG00000170890;ENSG00000171105;ENSG00000180210
GO:0006754	ATP biosynthetic process	BP	2	0.021463	0.0511804	ENSG00000171105;ENSG00000186951
GO:0009132	nucleoside diphosphate metabolic process	BP	2	0.021463	0.0511804	ENSG00000171105;ENSG00000186951
GO:0030238	male sex determination	BP	1	0.021581	0.0511804	ENSG00000171105
GO:0030812	negative regulation of nucleotide catabolic process	BP	1	0.021581	0.0511804	ENSG00000186951
GO:0032105	negative regulation of response to extracellular stimulus	BP	1	0.021581	0.0511804	ENSG00000186951
GO:0032108	negative regulation of response to nutrient levels	BP	1	0.021581	0.0511804	ENSG00000186951
GO:0032310	prostaglandin secretion	BP	1	0.021581	0.0511804	ENSG00000007171
GO:0032688	negative regulation of interferon-beta production	BP	1	0.021581	0.0511804	ENSG00000105426
GO:0034310	primary alcohol catabolic process	BP	1	0.021581	0.0511804	ENSG00000198074
GO:0035635	entry of bacterium into host cell	BP	1	0.021581	0.0511804	ENSG00000105976

GO:0044126	regulation of growth of symbiont in host	BP	1	0.021581	0.0511804	ENSG00000005381
GO:0044146	negative regulation of growth of symbiont involved in interaction with host	BP	1	0.021581	0.0511804	ENSG00000005381
GO:0051198	negative regulation of coenzyme metabolic process	BP	1	0.021581	0.0511804	ENSG00000186951
GO:0051709	regulation of killing of cells of other organism	BP	1	0.021581	0.0511804	ENSG00000007171
GO:0051917	regulation of fibrinolysis	BP	1	0.021581	0.0511804	ENSG00000180210
GO:0070571	negative regulation of neuron projection regeneration	BP	1	0.021581	0.0511804	ENSG00000105426
GO:1901386	negative regulation of voltage-gated calcium channel activity	BP	1	0.021581	0.0511804	ENSG00000178623
GO:1902894	negative regulation of pri-miRNA transcription by RNA polymerase II	BP	1	0.021581	0.0511804	ENSG00000186951
GO:1904889	regulation of excitatory synapse assembly	BP	1	0.021581	0.0511804	ENSG00000105426
GO:1905153	regulation of membrane invagination	BP	1	0.021581	0.0511804	ENSG00000132170
GO:0034767	positive regulation of ion transmembrane transport	BP	2	0.021721	0.05146161	ENSG00000085563;ENSG00000180210
GO:0008277	regulation of G protein-coupled receptor signaling pathway	BP	2	0.02198	0.05197312	ENSG00000105976;ENSG00000180210
GO:0097530	granulocyte migration	BP	2	0.02198	0.05197312	ENSG00000163464;ENSG00000170890
GO:0051384	response to glucocorticoid	BP	2	0.02224	0.05253695	ENSG00000073756;ENSG00000146648
GO:0009117	nucleotide metabolic process	BP	4	0.02265	0.05345438	ENSG00000007171;ENSG00000073756;ENSG00000171105;ENSG00000186951
GO:0002443	leukocyte mediated immunity	BP	5	0.022701	0.0535227	ENSG00000005381;ENSG00000100985;ENSG00000163464;ENSG00000170890;ENSG00000180210
GO:0006732	coenzyme metabolic process	BP	3	0.022964	0.05360821	ENSG00000073756;ENSG00000171105;ENSG00000186951
GO:0001660	fever generation	BP	1	0.023003	0.05360821	ENSG00000073756
GO:0017014	protein nitrosylation	BP	1	0.023003	0.05360821	ENSG00000007171
GO:0018119	peptidyl-cysteine S-nitrosylation	BP	1	0.023003	0.05360821	ENSG00000007171
GO:0022038	corpus callosum development	BP	1	0.023003	0.05360821	ENSG00000105426
GO:0044144	modulation of growth of symbiont involved in interaction with host	BP	1	0.023003	0.05360821	ENSG00000005381

GO:0045898	regulation of RNA polymerase II transcriptional preinitiation complex assembly	BP	1	0.023003	0.05360821	ENSG00000091831
GO:0045986	negative regulation of smooth muscle contraction	BP	1	0.023003	0.05360821	ENSG00000073756
GO:0071391	cellular response to estrogen stimulus	BP	1	0.023003	0.05360821	ENSG00000091831
GO:0071732	cellular response to nitric oxide	BP	1	0.023003	0.05360821	ENSG00000149968
GO:0090141	positive regulation of mitochondrial fission	BP	1	0.023003	0.05360821	ENSG00000128052
GO:2001170	negative regulation of ATP biosynthetic process	BP	1	0.023003	0.05360821	ENSG00000186951
GO:0048754	branching morphogenesis of an epithelial tube	BP	2	0.023028	0.05361359	ENSG00000091831;ENSG00000105976
GO:0002252	immune effector process	BP	6	0.023127	0.05379194	ENSG00000005381;ENSG00000100985;ENSG00000143365;ENSG00000163464;ENSG00000170890;ENSG00000180210
GO:0044248	cellular catabolic process	BP	8	0.023359	0.05428003	ENSG00000005381;ENSG00000007171;ENSG00000100823;ENSG00000105976;ENSG00000128052;ENSG00000171105;ENSG00000186951;ENSG00000198074
GO:0006753	nucleoside phosphate metabolic process	BP	4	0.023441	0.05441798	ENSG00000007171;ENSG00000073756;ENSG00000171105;ENSG00000186951
GO:0010468	regulation of gene expression	BP	13	0.023522	0.05452441	ENSG00000007171;ENSG00000091831;ENSG00000100823;ENSG00000105976;ENSG00000106546;ENSG00000128052;ENSG00000132170;ENSG00000143365;ENSG00000146648;ENSG00000170890;ENSG00000171105;ENSG00000180210;ENSG00000186951
GO:0031047	gene silencing by RNA	BP	3	0.023532	0.05452441	ENSG00000091831;ENSG00000132170;ENSG00000146648
GO:0045934	negative regulation of nucleobase-containing compound metabolic process	BP	6	0.023651	0.05474891	ENSG00000091831;ENSG00000100823;ENSG00000106546;ENSG00000132170;ENSG00000143365;ENSG00000186951
GO:0002224	toll-like receptor signaling pathway	BP	2	0.023828	0.05484208	ENSG00000091831;ENSG00000105426
GO:0030260	entry into host cell	BP	2	0.023828	0.05484208	ENSG00000105976;ENSG00000146648
GO:0044409	entry into host	BP	2	0.023828	0.05484208	ENSG00000105976;ENSG00000146648
GO:0046661	male sex differentiation	BP	2	0.023828	0.05484208	ENSG00000091831;ENSG00000171105
GO:0051806	entry into cell of other organism involved in symbiotic interaction	BP	2	0.023828	0.05484208	ENSG00000105976;ENSG00000146648
GO:0051828	entry into other organism involved in symbiotic interaction	BP	2	0.023828	0.05484208	ENSG00000105976;ENSG00000146648
GO:0000187	activation of MAPK activity	BP	2	0.024097	0.055011	ENSG00000170890;ENSG00000171105
GO:0008360	regulation of cell shape	BP	2	0.024097	0.055011	ENSG00000128052;ENSG00000180210
GO:0009206	purine ribonucleoside triphosphate biosynthetic process	BP	2	0.024367	0.055011	ENSG00000171105;ENSG00000186951

GO:0031214	biomineral tissue development	BP	2	0.024367	0.055011	ENSG00000073756;ENSG00000186951
GO:1905952	regulation of lipid localization	BP	2	0.024367	0.055011	ENSG00000132170;ENSG00000186951
GO:0031000	response to caffeine	BP	1	0.024424	0.055011	ENSG00000132170
GO:0031652	positive regulation of heat generation	BP	1	0.024424	0.055011	ENSG00000073756
GO:0032966	negative regulation of collagen biosynthetic process	BP	1	0.024424	0.055011	ENSG00000132170
GO:0034356	NAD biosynthesis via nicotinamide riboside salvage pathway	BP	1	0.024424	0.055011	ENSG00000073756
GO:0036270	response to diuretic	BP	1	0.024424	0.055011	ENSG00000132170
GO:0042753	positive regulation of circadian rhythm	BP	1	0.024424	0.055011	ENSG00000143365
GO:0044117	growth of symbiont in host	BP	1	0.024424	0.055011	ENSG00000005381
GO:0045722	positive regulation of gluconeogenesis	BP	1	0.024424	0.055011	ENSG00000186951
GO:0045725	positive regulation of glycogen biosynthetic process	BP	1	0.024424	0.055011	ENSG00000171105
GO:0051767	nitric-oxide synthase biosynthetic process	BP	1	0.024424	0.055011	ENSG00000128052
GO:0051769	regulation of nitric-oxide synthase biosynthetic process	BP	1	0.024424	0.055011	ENSG00000128052
GO:0051883	killing of cells in other organism involved in symbiotic interaction	BP	1	0.024424	0.055011	ENSG00000180210
GO:0051969	regulation of transmission of nerve impulse	BP	1	0.024424	0.055011	ENSG00000178623
GO:0060263	regulation of respiratory burst	BP	1	0.024424	0.055011	ENSG00000171105
GO:0071380	cellular response to prostaglandin E stimulus	BP	1	0.024424	0.055011	ENSG00000132170
GO:0090185	negative regulation of kidney development	BP	1	0.024424	0.055011	ENSG00000100985
GO:1903209	positive regulation of oxidative stress-induced cell death	BP	1	0.024424	0.055011	ENSG00000149968
GO:2001267	regulation of cysteine-type endopeptidase activity involved in apoptotic signaling pathway	BP	1	0.024424	0.055011	ENSG00000100985
GO:0045787	positive regulation of cell cycle	BP	3	0.024545	0.05523123	ENSG00000100823;ENSG00000146648;ENSG00000171105
GO:0009145	purine nucleoside triphosphate biosynthetic process	BP	2	0.024639	0.05539133	ENSG00000171105;ENSG00000186951

GO:0060402	calcium ion transport into cytosol	BP	2	0.024912	0.0559528	ENSG00000170890;ENSG00000180210
GO:0034654	nucleobase-containing compound biosynthetic process	BP	12	0.025056	0.05622452	ENSG00000007171;ENSG00000073756;ENSG00000091831;ENSG00000100823;ENSG00000105976;ENSG00000106546;ENSG00000132170;ENSG00000143365;ENSG00000146648;ENSG00000170890;ENSG00000171105;ENSG00000186951
GO:1901342	regulation of vasculature development	BP	3	0.025283	0.05668076	ENSG00000073756;ENSG00000128052;ENSG00000132170
GO:0031326	regulation of cellular biosynthetic process	BP	12	0.025337	0.05674982	ENSG00000007171;ENSG00000073756;ENSG00000091831;ENSG00000100823;ENSG00000105976;ENSG00000106546;ENSG00000132170;ENSG00000143365;ENSG00000146648;ENSG00000170890;ENSG00000171105;ENSG00000186951
GO:0051100	negative regulation of binding	BP	2	0.025461	0.05677881	ENSG00000105976;ENSG00000186951
GO:0032989	cellular component morphogenesis	BP	5	0.02558	0.05677881	ENSG00000105426;ENSG00000105976;ENSG00000128052;ENSG00000146648;ENSG00000180210
GO:0008610	lipid biosynthetic process	BP	4	0.025597	0.05677881	ENSG00000073756;ENSG00000095303;ENSG00000159228;ENSG00000170890
GO:0010942	positive regulation of cell death	BP	4	0.025597	0.05677881	ENSG00000073756;ENSG00000100985;ENSG00000132170;ENSG00000149968
GO:0035051	cardiocyte differentiation	BP	2	0.025738	0.05677881	ENSG00000146648;ENSG00000186951
GO:0007597	blood coagulation, intrinsic pathway	BP	1	0.025843	0.05677881	ENSG00000180210
GO:0010713	negative regulation of collagen metabolic process	BP	1	0.025843	0.05677881	ENSG00000132170
GO:0015732	prostaglandin transport	BP	1	0.025843	0.05677881	ENSG00000007171
GO:0019372	lipoxygenase pathway	BP	1	0.025843	0.05677881	ENSG00000073756
GO:0030540	female genitalia development	BP	1	0.025843	0.05677881	ENSG00000091831
GO:0030809	negative regulation of nucleotide biosynthetic process	BP	1	0.025843	0.05677881	ENSG00000186951
GO:0031998	regulation of fatty acid beta-oxidation	BP	1	0.025843	0.05677881	ENSG00000186951
GO:0044110	growth involved in symbiotic interaction	BP	1	0.025843	0.05677881	ENSG00000005381
GO:0044116	growth of symbiont involved in interaction with host	BP	1	0.025843	0.05677881	ENSG00000005381
GO:0045723	positive regulation of fatty acid biosynthetic process	BP	1	0.025843	0.05677881	ENSG00000073756
GO:0051852	disruption by host of symbiont cells	BP	1	0.025843	0.05677881	ENSG00000180210
GO:0070166	enamel mineralization	BP	1	0.025843	0.05677881	ENSG00000186951
GO:0070875	positive regulation of glycogen metabolic process	BP	1	0.025843	0.05677881	ENSG00000171105

GO:0071318	cellular response to ATP	BP	1	0.025843	0.05677881	ENSG00000073756
GO:0090335	regulation of brown fat cell differentiation	BP	1	0.025843	0.05677881	ENSG00000073756
GO:1900372	negative regulation of purine nucleotide biosynthetic process	BP	1	0.025843	0.05677881	ENSG00000186951
GO:0051223	regulation of protein transport	BP	4	0.025915	0.05688555	ENSG00000007171;ENSG00000073756;ENSG00000146648;ENSG00000170890
GO:0009201	ribonucleoside triphosphate biosynthetic process	BP	2	0.026016	0.05700334	ENSG00000171105;ENSG00000186951
GO:0099173	postsynapse organization	BP	2	0.026016	0.05700334	ENSG00000105426;ENSG00000171105
GO:0043412	macromolecule modification	BP	12	0.026277	0.05752462	ENSG00000007171;ENSG00000073756;ENSG00000091831;ENSG00000100823;ENSG00000100985;ENSG00000105426;ENSG00000105976;ENSG00000128052;ENSG00000146648;ENSG00000170890;ENSG00000171105;ENSG00000180210
GO:0051707	response to other organism	BP	5	0.02702	0.05877033	ENSG00000005381;ENSG00000007171;ENSG00000073756;ENSG00000170890;ENSG00000180210
GO:0031960	response to corticosteroid	BP	2	0.027139	0.05877033	ENSG00000073756;ENSG00000146648
GO:0043207	response to external biotic stimulus	BP	5	0.027192	0.05877033	ENSG00000005381;ENSG00000007171;ENSG00000073756;ENSG00000170890;ENSG00000180210
GO:0006527	arginine catabolic process	BP	1	0.027259	0.05877033	ENSG00000007171
GO:0010544	negative regulation of platelet activation	BP	1	0.027259	0.05877033	ENSG00000180210
GO:0031650	regulation of heat generation	BP	1	0.027259	0.05877033	ENSG00000073756
GO:0032095	regulation of response to food	BP	1	0.027259	0.05877033	ENSG00000186951
GO:0032930	positive regulation of superoxide anion generation	BP	1	0.027259	0.05877033	ENSG00000146648
GO:0036148	phosphatidylglycerol acyl-chain remodeling	BP	1	0.027259	0.05877033	ENSG00000170890
GO:0051546	keratinocyte migration	BP	1	0.027259	0.05877033	ENSG00000100985
GO:0071731	response to nitric oxide	BP	1	0.027259	0.05877033	ENSG00000149968
GO:1902170	cellular response to reactive nitrogen species	BP	1	0.027259	0.05877033	ENSG00000149968
GO:1902644	tertiary alcohol metabolic process	BP	1	0.027259	0.05877033	ENSG00000198074
GO:1905288	vascular associated smooth muscle cell apoptotic process	BP	1	0.027259	0.05877033	ENSG00000132170
GO:1905459	regulation of vascular associated smooth muscle cell apoptotic process	BP	1	0.027259	0.05877033	ENSG00000132170

GO:1905874	regulation of postsynaptic density organization	BP	1	0.027259	0.05877033	ENSG00000105426
GO:1905939	regulation of gonad development	BP	1	0.027259	0.05877033	ENSG00000171105
GO:0006508	proteolysis	BP	7	0.027403	0.0589138	ENSG00000073756;ENSG00000087245;ENSG00000091831;ENSG00000100985;ENSG00000132170;ENSG00000149968;ENSG00000180210
GO:0001936	regulation of endothelial cell proliferation	BP	2	0.027423	0.0589138	ENSG00000128052;ENSG00000132170
GO:0009166	nucleotide catabolic process	BP	2	0.027423	0.0589138	ENSG00000171105;ENSG00000186951
GO:2001242	regulation of intrinsic apoptotic signaling pathway	BP	2	0.027423	0.0589138	ENSG00000073756;ENSG00000100985
GO:0015698	inorganic anion transport	BP	2	0.027995	0.06003517	ENSG00000085563;ENSG00000104267
GO:1903034	regulation of response to wounding	BP	2	0.027995	0.06003517	ENSG00000105426;ENSG00000180210
GO:0018130	heterocycle biosynthetic process	BP	12	0.02802	0.06003559	ENSG00000007171;ENSG00000073756;ENSG00000091831;ENSG00000100823;ENSG00000105976;ENSG00000106546;ENSG00000132170;ENSG00000143365;ENSG00000146648;ENSG00000170890;ENSG00000171105;ENSG00000186951
GO:0051171	regulation of nitrogen compound metabolic process	BP	15	0.028102	0.06015724	ENSG00000007171;ENSG00000073756;ENSG00000091831;ENSG00000100823;ENSG00000100985;ENSG00000105976;ENSG00000106546;ENSG00000128052;ENSG00000132170;ENSG00000143365;ENSG00000146648;ENSG00000170890;ENSG00000171105;ENSG00000180210;ENSG00000186951
GO:0009127	purine nucleoside monophosphate biosynthetic process	BP	2	0.028283	0.06038431	ENSG00000171105;ENSG00000186951
GO:0009168	purine ribonucleoside monophosphate biosynthetic process	BP	2	0.028283	0.06038431	ENSG00000171105;ENSG00000186951
GO:0030307	positive regulation of cell growth	BP	2	0.028283	0.06038431	ENSG00000146648;ENSG00000180210
GO:0019438	aromatic compound biosynthetic process	BP	12	0.028414	0.06052561	ENSG00000007171;ENSG00000073756;ENSG00000091831;ENSG00000100823;ENSG00000105976;ENSG00000106546;ENSG00000132170;ENSG00000143365;ENSG00000146648;ENSG00000170890;ENSG00000171105;ENSG00000186951
GO:0019538	protein metabolic process	BP	15	0.02845	0.06052561	ENSG00000007171;ENSG00000073756;ENSG00000087245;ENSG00000091831;ENSG00000100985;ENSG00000105426;ENSG00000105976;ENSG00000128052;ENSG00000132170;ENSG00000146648;ENSG00000149968;ENSG00000170890;ENSG00000171105;ENSG00000180210;ENSG00000186951
GO:2001257	regulation of cation channel activity	BP	2	0.028571	0.06052561	ENSG00000100985;ENSG00000178623
GO:0016137	glycoside metabolic process	BP	1	0.028674	0.06052561	ENSG00000198074
GO:0032225	regulation of synaptic transmission, dopaminergic	BP	1	0.028674	0.06052561	ENSG00000073756
GO:0034393	positive regulation of smooth muscle cell apoptotic process	BP	1	0.028674	0.06052561	ENSG00000132170
GO:0043586	tongue development	BP	1	0.028674	0.06052561	ENSG00000146648
GO:0060602	branch elongation of an epithelium	BP	1	0.028674	0.06052561	ENSG00000091831
GO:0060749	mammary gland alveolus development	BP	1	0.028674	0.06052561	ENSG00000091831

GO:0061377	mammary gland lobule development	BP	1	0.028674	0.06052561	ENSG00000091831
GO:0097062	dendritic spine maintenance	BP	1	0.028674	0.06052561	ENSG00000171105
GO:0097107	postsynaptic density assembly	BP	1	0.028674	0.06052561	ENSG00000105426
GO:2000696	regulation of epithelial cell differentiation involved in kidney development	BP	1	0.028674	0.06052561	ENSG00000100985
GO:0002682	regulation of immune system process	BP	7	0.028701	0.06052887	ENSG00000091831;ENSG00000104267;ENSG00000105426;ENSG00000106546;ENSG00000132170;ENSG00000170890;ENSG00000180210
GO:0009896	positive regulation of catabolic process	BP	3	0.028832	0.06069976	ENSG00000128052;ENSG00000171105;ENSG00000186951
GO:0030099	myeloid cell differentiation	BP	3	0.028832	0.06069976	ENSG00000100985;ENSG00000104267;ENSG00000132170
GO:1901292	nucleoside phosphate catabolic process	BP	2	0.029153	0.06132182	ENSG00000171105;ENSG00000186951
GO:0002366	leukocyte activation involved in immune response	BP	4	0.029224	0.06141912	ENSG00000005381;ENSG00000100985;ENSG00000143365;ENSG00000163464
GO:0009142	nucleoside triphosphate biosynthetic process	BP	2	0.029445	0.06177574	ENSG00000171105;ENSG00000186951
GO:0060401	cytosolic calcium ion transport	BP	2	0.029445	0.06177574	ENSG00000170890;ENSG00000180210
GO:0060249	anatomical structure homeostasis	BP	3	0.029637	0.0621246	ENSG00000100823;ENSG00000104267;ENSG00000146648
GO:0002263	cell activation involved in immune response	BP	4	0.029684	0.06216913	ENSG00000005381;ENSG00000100985;ENSG00000143365;ENSG00000163464
GO:0009607	response to biotic stimulus	BP	5	0.029962	0.06225798	ENSG00000005381;ENSG00000007171;ENSG00000073756;ENSG00000170890;ENSG00000180210
GO:0010042	response to manganese ion	BP	1	0.030087	0.06225798	ENSG00000073756
GO:0010310	regulation of hydrogen peroxide metabolic process	BP	1	0.030087	0.06225798	ENSG00000149968
GO:0015669	gas transport	BP	1	0.030087	0.06225798	ENSG00000104267
GO:0019370	leukotriene biosynthetic process	BP	1	0.030087	0.06225798	ENSG00000170890
GO:0032098	regulation of appetite	BP	1	0.030087	0.06225798	ENSG00000186951
GO:0033189	response to vitamin A	BP	1	0.030087	0.06225798	ENSG00000132170
GO:0035162	embryonic hemopoiesis	BP	1	0.030087	0.06225798	ENSG00000128052
GO:0045821	positive regulation of glycolytic process	BP	1	0.030087	0.06225798	ENSG00000171105

GO:0048670	regulation of collateral sprouting	BP	1	0.030087	0.06225798	ENSG00000105426
GO:0051818	disruption of cells of other organism involved in symbiotic interaction	BP	1	0.030087	0.06225798	ENSG00000180210
GO:0051900	regulation of mitochondrial depolarization	BP	1	0.030087	0.06225798	ENSG00000128052
GO:0055093	response to hyperoxia	BP	1	0.030087	0.06225798	ENSG00000132170
GO:0071636	positive regulation of transforming growth factor beta production	BP	1	0.030087	0.06225798	ENSG00000073756
GO:0044282	small molecule catabolic process	BP	3	0.03029	0.0626247	ENSG00000007171;ENSG00000186951;ENSG00000198074
GO:0043408	regulation of MAPK cascade	BP	4	0.030499	0.06300307	ENSG00000128052;ENSG00000146648;ENSG00000170890;ENSG00000171105
GO:0001909	leukocyte mediated cytotoxicity	BP	2	0.030626	0.06321281	ENSG00000007171;ENSG00000180210
GO:0010558	negative regulation of macromolecule biosynthetic process	BP	6	0.031151	0.06424021	ENSG00000091831;ENSG00000100823;ENSG00000106546;ENSG00000132170;ENSG00000143365;ENSG00000186951
GO:0050729	positive regulation of inflammatory response	BP	2	0.031224	0.06433685	ENSG00000073756;ENSG00000146648
GO:0003012	muscle system process	BP	3	0.031283	0.06435135	ENSG00000073756;ENSG00000148680;ENSG00000186951
GO:0070201	regulation of establishment of protein localization	BP	4	0.031447	0.06435135	ENSG00000007171;ENSG00000073756;ENSG00000146648;ENSG00000170890
GO:0010226	response to lithium ion	BP	1	0.031497	0.06435135	ENSG00000073756
GO:0030728	ovulation	BP	1	0.031497	0.06435135	ENSG00000073756
GO:0030813	positive regulation of nucleotide catabolic process	BP	1	0.031497	0.06435135	ENSG00000171105
GO:0036150	phosphatidylserine acyl-chain remodeling	BP	1	0.031497	0.06435135	ENSG00000170890
GO:0048714	positive regulation of oligodendrocyte differentiation	BP	1	0.031497	0.06435135	ENSG00000132170
GO:0051197	positive regulation of coenzyme metabolic process	BP	1	0.031497	0.06435135	ENSG00000171105
GO:0060261	positive regulation of transcription initiation from RNA polymerase II promoter	BP	1	0.031497	0.06435135	ENSG00000091831
GO:0061436	establishment of skin barrier	BP	1	0.031497	0.06435135	ENSG00000105976
GO:0002040	sprouting angiogenesis	BP	2	0.031525	0.06435334	ENSG00000073756;ENSG00000128052
GO:0021543	pallium development	BP	2	0.031827	0.06491465	ENSG00000105426;ENSG00000146648

GO:0051222	positive regulation of protein transport	BP	3	0.032124	0.06536723	ENSG00000073756;ENSG00000146648;ENSG00000170890
GO:0001935	endothelial cell proliferation	BP	2	0.03213	0.06536723	ENSG00000128052;ENSG00000132170
GO:0061138	morphogenesis of a branching epithelium	BP	2	0.03213	0.06536723	ENSG00000091831;ENSG00000105976
GO:0051345	positive regulation of hydrolase activity	BP	4	0.032412	0.06581988	ENSG00000091831;ENSG00000132170;ENSG00000146648;ENSG00000170890
GO:0009156	ribonucleoside monophosphate biosynthetic process	BP	2	0.032434	0.06581988	ENSG00000171105;ENSG00000186951
GO:2000045	regulation of G1/S transition of mitotic cell cycle	BP	2	0.032434	0.06581988	ENSG00000100823;ENSG00000146648
GO:0052548	regulation of endopeptidase activity	BP	3	0.032464	0.06582654	ENSG00000073756;ENSG00000100985;ENSG00000132170
GO:0006812	cation transport	BP	5	0.032809	0.06600301	ENSG00000073756;ENSG00000100985;ENSG00000170890;ENSG00000178623;ENSG00000180210
GO:0031281	positive regulation of cyclase activity	BP	1	0.032906	0.06600301	ENSG00000007171
GO:0031649	heat generation	BP	1	0.032906	0.06600301	ENSG00000073756
GO:0032799	low-density lipoprotein receptor particle metabolic process	BP	1	0.032906	0.06600301	ENSG00000132170
GO:0035024	negative regulation of Rho protein signal transduction	BP	1	0.032906	0.06600301	ENSG00000105976
GO:0035357	peroxisome proliferator activated receptor signaling pathway	BP	1	0.032906	0.06600301	ENSG00000132170
GO:0048012	hepatocyte growth factor receptor signaling pathway	BP	1	0.032906	0.06600301	ENSG00000105976
GO:0050996	positive regulation of lipid catabolic process	BP	1	0.032906	0.06600301	ENSG00000186951
GO:0051349	positive regulation of lyase activity	BP	1	0.032906	0.06600301	ENSG00000007171
GO:0071379	cellular response to prostaglandin stimulus	BP	1	0.032906	0.06600301	ENSG00000132170
GO:0071404	cellular response to low-density lipoprotein particle stimulus	BP	1	0.032906	0.06600301	ENSG00000132170
GO:2000637	positive regulation of gene silencing by miRNA	BP	1	0.032906	0.06600301	ENSG00000146648
GO:2000726	negative regulation of cardiac muscle cell differentiation	BP	1	0.032906	0.06600301	ENSG00000186951
GO:0055086	nucleobase-containing small molecule metabolic process	BP	4	0.033395	0.06688351	ENSG00000007171;ENSG00000073756;ENSG00000171105;ENSG00000186951
GO:0051246	regulation of protein metabolic process	BP	9	0.0334	0.06688351	ENSG00000007171;ENSG00000073756;ENSG00000100985;ENSG00000128052;ENSG00000132170;ENSG00000146648;ENSG00000170890;ENSG00000171105;ENSG00000180210

GO:0010821	regulation of mitochondrion organization	BP	2	0.033972	0.06775713	ENSG00000100985;ENSG00000128052
GO:0007369	gastrulation	BP	2	0.034282	0.06775713	ENSG00000087245;ENSG00000100985
GO:0019637	organophosphate metabolic process	BP	5	0.034296	0.06775713	ENSG00000007171;ENSG00000073756;ENSG00000170890;ENSG00000171105;ENSG00000186951
GO:0007530	sex determination	BP	1	0.034313	0.06775713	ENSG00000171105
GO:0030325	adrenal gland development	BP	1	0.034313	0.06775713	ENSG00000171105
GO:0032928	regulation of superoxide anion generation	BP	1	0.034313	0.06775713	ENSG00000146648
GO:0034505	tooth mineralization	BP	1	0.034313	0.06775713	ENSG00000186951
GO:0036315	cellular response to sterol	BP	1	0.034313	0.06775713	ENSG00000143365
GO:0051446	positive regulation of meiotic cell cycle	BP	1	0.034313	0.06775713	ENSG00000171105
GO:0051882	mitochondrial depolarization	BP	1	0.034313	0.06775713	ENSG00000128052
GO:0060065	uterus development	BP	1	0.034313	0.06775713	ENSG00000091831
GO:0060148	positive regulation of posttranscriptional gene silencing	BP	1	0.034313	0.06775713	ENSG00000146648
GO:0061050	regulation of cell growth involved in cardiac muscle cell development	BP	1	0.034313	0.06775713	ENSG00000186951
GO:0098698	postsynaptic specialization assembly	BP	1	0.034313	0.06775713	ENSG00000105426
GO:1901032	negative regulation of response to reactive oxygen species	BP	1	0.034313	0.06775713	ENSG00000105976
GO:1903206	negative regulation of hydrogen peroxide-induced cell death	BP	1	0.034313	0.06775713	ENSG00000105976
GO:2001039	negative regulation of cellular response to drug	BP	1	0.034313	0.06775713	ENSG00000105976
GO:0080090	regulation of primary metabolic process	BP	15	0.035187	0.06942682	ENSG00000007171;ENSG00000073756;ENSG00000091831;ENSG00000100823;ENSG00000100985;ENSG00000105976;ENSG00000106546;ENSG00000128052;ENSG00000132170;ENSG00000143365;ENSG00000146648;ENSG00000170890;ENSG00000171105;ENSG00000180210;ENSG00000186951
GO:0032940	secretion by cell	BP	6	0.035223	0.06944153	ENSG00000005381;ENSG00000007171;ENSG00000100985;ENSG00000146648;ENSG00000163464;ENSG00000170890
GO:0043254	regulation of protein complex assembly	BP	3	0.03543	0.06979043	ENSG00000091831;ENSG00000105976;ENSG00000149968
GO:0045766	positive regulation of angiogenesis	BP	2	0.035538	0.06979043	ENSG00000073756;ENSG00000128052
GO:0002407	dendritic cell chemotaxis	BP	1	0.035717	0.06979043	ENSG00000163464

GO:0032104	regulation of response to extracellular stimulus	BP	1	0.035717	0.06979043	ENSG00000186951
GO:0032107	regulation of response to nutrient levels	BP	1	0.035717	0.06979043	ENSG00000186951
GO:0033561	regulation of water loss via skin	BP	1	0.035717	0.06979043	ENSG00000105976
GO:0034695	response to prostaglandin E	BP	1	0.035717	0.06979043	ENSG00000132170
GO:0051894	positive regulation of focal adhesion assembly	BP	1	0.035717	0.06979043	ENSG00000128052
GO:1900409	positive regulation of cellular response to oxidative stress	BP	1	0.035717	0.06979043	ENSG00000149968
GO:1903579	negative regulation of ATP metabolic process	BP	1	0.035717	0.06979043	ENSG00000186951
GO:1905563	negative regulation of vascular endothelial cell proliferation	BP	1	0.035717	0.06979043	ENSG00000132170
GO:0035265	organ growth	BP	2	0.035854	0.07000091	ENSG00000091831;ENSG00000186951
GO:1901362	organic cyclic compound biosynthetic process	BP	12	0.035886	0.0700076	ENSG00000007171;ENSG00000073756;ENSG00000091831;ENSG00000100823;ENSG00000105976;ENSG00000106546;ENSG00000132170;ENSG00000143365;ENSG00000146648;ENSG00000170890;ENSG00000171105;ENSG00000186951
GO:0009124	nucleoside monophosphate biosynthetic process	BP	2	0.036172	0.07045055	ENSG00000171105;ENSG00000186951
GO:0016052	carbohydrate catabolic process	BP	2	0.036172	0.07045055	ENSG00000171105;ENSG00000186951
GO:0001763	morphogenesis of a branching structure	BP	2	0.036491	0.0709486	ENSG00000091831;ENSG00000105976
GO:0000122	negative regulation of transcription by RNA polymerase II	BP	4	0.03658	0.0709486	ENSG00000091831;ENSG00000132170;ENSG00000143365;ENSG00000186951
GO:0051090	regulation of DNA-binding transcription factor activity	BP	3	0.036691	0.0709486	ENSG00000091831;ENSG00000132170;ENSG00000170890
GO:0022604	regulation of cell morphogenesis	BP	3	0.036873	0.0709486	ENSG00000105426;ENSG00000128052;ENSG00000180210
GO:0031327	negative regulation of cellular biosynthetic process	BP	6	0.036893	0.0709486	ENSG00000091831;ENSG00000100823;ENSG00000106546;ENSG00000132170;ENSG00000143365;ENSG00000186951
GO:0052547	regulation of peptidase activity	BP	3	0.037055	0.0709486	ENSG00000073756;ENSG00000100985;ENSG00000132170
GO:0051248	negative regulation of protein metabolic process	BP	5	0.037078	0.0709486	ENSG00000007171;ENSG00000073756;ENSG00000100985;ENSG00000146648;ENSG00000180210
GO:0031645	negative regulation of neurological system process	BP	1	0.03712	0.0709486	ENSG00000178623
GO:0035584	calcium-mediated signaling using intracellular calcium source	BP	1	0.03712	0.0709486	ENSG00000128052
GO:0045332	phospholipid translocation	BP	1	0.03712	0.0709486	ENSG00000085563

GO:0045686	negative regulation of glial cell differentiation	BP	1	0.03712	0.0709486	ENSG00000180210
GO:0046697	decidualization	BP	1	0.03712	0.0709486	ENSG00000073756
GO:0050927	positive regulation of positive chemotaxis	BP	1	0.03712	0.0709486	ENSG00000128052
GO:0060330	regulation of response to interferon-gamma	BP	1	0.03712	0.0709486	ENSG00000132170
GO:0060334	regulation of interferon-gamma-mediated signaling pathway	BP	1	0.03712	0.0709486	ENSG00000132170
GO:0060444	branching involved in mammary gland duct morphogenesis	BP	1	0.03712	0.0709486	ENSG00000091831
GO:0060571	morphogenesis of an epithelial fold	BP	1	0.03712	0.0709486	ENSG00000146648
GO:0060740	prostate gland epithelium morphogenesis	BP	1	0.03712	0.0709486	ENSG00000091831
GO:0072539	T-helper 17 cell differentiation	BP	1	0.03712	0.0709486	ENSG00000143365
GO:0097186	amelogenesis	BP	1	0.03712	0.0709486	ENSG00000186951
GO:1900120	regulation of receptor binding	BP	1	0.03712	0.0709486	ENSG00000100985
GO:1903959	regulation of anion transmembrane transport	BP	1	0.03712	0.0709486	ENSG00000085563
GO:1904385	cellular response to angiotensin	BP	1	0.03712	0.0709486	ENSG00000104267
GO:0050679	positive regulation of epithelial cell proliferation	BP	2	0.037131	0.0709486	ENSG00000128052;ENSG00000146648
GO:0045892	negative regulation of transcription, DNA-templated	BP	5	0.037184	0.07099291	ENSG00000091831;ENSG00000106546;ENSG00000132170;ENSG00000143365;ENSG00000186951
GO:0001817	regulation of cytokine production	BP	4	0.03737	0.07129157	ENSG00000007171;ENSG00000073756;ENSG00000105426;ENSG00000180210
GO:0010608	posttranscriptional regulation of gene expression	BP	4	0.038304	0.07234657	ENSG00000091831;ENSG00000100823;ENSG00000132170;ENSG00000146648
GO:1902806	regulation of cell cycle G1/S phase transition	BP	2	0.038426	0.07234657	ENSG00000100823;ENSG00000146648
GO:0010288	response to lead ion	BP	1	0.038521	0.07234657	ENSG00000073756
GO:0030194	positive regulation of blood coagulation	BP	1	0.038521	0.07234657	ENSG00000180210
GO:0032461	positive regulation of protein oligomerization	BP	1	0.038521	0.07234657	ENSG00000149968
GO:0032967	positive regulation of collagen biosynthetic process	BP	1	0.038521	0.07234657	ENSG00000180210

GO:0036149	phosphatidylinositol acyl-chain remodeling	BP	1	0.038521	0.07234657	ENSG00000170890
GO:0036152	phosphatidylethanolamine acyl-chain remodeling	BP	1	0.038521	0.07234657	ENSG00000170890
GO:0045932	negative regulation of muscle contraction	BP	1	0.038521	0.07234657	ENSG00000073756
GO:0050926	regulation of positive chemotaxis	BP	1	0.038521	0.07234657	ENSG00000128052
GO:0051497	negative regulation of stress fiber assembly	BP	1	0.038521	0.07234657	ENSG00000105976
GO:0060343	trabecula formation	BP	1	0.038521	0.07234657	ENSG00000087245
GO:0072378	blood coagulation, fibrin clot formation	BP	1	0.038521	0.07234657	ENSG00000180210
GO:0080111	DNA demethylation	BP	1	0.038521	0.07234657	ENSG00000100823
GO:0090140	regulation of mitochondrial fission	BP	1	0.038521	0.07234657	ENSG00000128052
GO:1900048	positive regulation of hemostasis	BP	1	0.038521	0.07234657	ENSG00000180210
GO:1900543	negative regulation of purine nucleotide metabolic process	BP	1	0.038521	0.07234657	ENSG00000186951
GO:1902884	positive regulation of response to oxidative stress	BP	1	0.038521	0.07234657	ENSG00000149968
GO:1904861	excitatory synapse assembly	BP	1	0.038521	0.07234657	ENSG00000105426
GO:2000171	negative regulation of dendrite development	BP	1	0.038521	0.07234657	ENSG00000105426
GO:0009890	negative regulation of biosynthetic process	BP	6	0.038983	0.07315824	ENSG00000091831;ENSG00000100823;ENSG00000106546;ENSG00000132170;ENSG00000143365;ENSG00000186951
GO:0008544	epidermis development	BP	3	0.039094	0.07319563	ENSG00000146648;ENSG00000171105;ENSG00000186951
GO:0016458	gene silencing	BP	3	0.039094	0.07319563	ENSG00000091831;ENSG00000132170;ENSG00000146648
GO:1904951	positive regulation of establishment of protein localization	BP	3	0.039094	0.07319563	ENSG00000073756;ENSG00000146648;ENSG00000170890
GO:0016051	carbohydrate biosynthetic process	BP	2	0.039409	0.07365839	ENSG00000171105;ENSG00000186951
GO:1901576	organic substance biosynthetic process	BP	15	0.039718	0.07365839	ENSG00000007171;ENSG00000073756;ENSG00000091831;ENSG00000095303;ENSG00000100823;ENSG00000105976;ENSG00000106546;ENSG00000128052;ENSG00000132170;ENSG00000143365;ENSG00000146648;ENSG00000159228;ENSG00000170890;ENSG00000171105;ENSG00000186951
GO:0007266	Rho protein signal transduction	BP	2	0.039738	0.07365839	ENSG00000105976;ENSG00000178623
GO:0048468	cell development	BP	7	0.039902	0.07365839	ENSG00000091831;ENSG00000105426;ENSG00000105976;ENSG00000132170;ENSG00000146648;ENSG00000180210;ENSG00000186951

GO:0006525	arginine metabolic process	BP	1	0.03992	0.07365839	ENSG00000007171
GO:0008209	androgen metabolic process	BP	1	0.03992	0.07365839	ENSG00000091831
GO:0010575	positive regulation of vascular endothelial growth factor production	BP	1	0.03992	0.07365839	ENSG00000073756
GO:0010714	positive regulation of collagen metabolic process	BP	1	0.03992	0.07365839	ENSG00000180210
GO:0033598	mammary gland epithelial cell proliferation	BP	1	0.03992	0.07365839	ENSG00000091831
GO:0034204	lipid translocation	BP	1	0.03992	0.07365839	ENSG00000085563
GO:0036151	phosphatidylcholine acyl-chain remodeling	BP	1	0.03992	0.07365839	ENSG00000170890
GO:0036296	response to increased oxygen levels	BP	1	0.03992	0.07365839	ENSG00000132170
GO:0042730	fibrinolysis	BP	1	0.03992	0.07365839	ENSG00000180210
GO:0043457	regulation of cellular respiration	BP	1	0.03992	0.07365839	ENSG00000007171
GO:0045980	negative regulation of nucleotide metabolic process	BP	1	0.03992	0.07365839	ENSG00000186951
GO:0048668	collateral sprouting	BP	1	0.03992	0.07365839	ENSG00000105426
GO:0050820	positive regulation of coagulation	BP	1	0.03992	0.07365839	ENSG00000180210
GO:0060512	prostate gland morphogenesis	BP	1	0.03992	0.07365839	ENSG00000091831
GO:0099560	synaptic membrane adhesion	BP	1	0.03992	0.07365839	ENSG00000105426
GO:0097164	ammonium ion metabolic process	BP	2	0.040401	0.07448903	ENSG00000170890;ENSG00000198074
GO:0034404	nucleobase-containing small molecule biosynthetic process	BP	2	0.040734	0.07504548	ENSG00000171105;ENSG00000186951
GO:0005979	regulation of glycogen biosynthetic process	BP	1	0.041317	0.07544479	ENSG00000171105
GO:0006730	one-carbon metabolic process	BP	1	0.041317	0.07544479	ENSG00000133742
GO:0010962	regulation of glucan biosynthetic process	BP	1	0.041317	0.07544479	ENSG00000171105
GO:0034162	toll-like receptor 9 signaling pathway	BP	1	0.041317	0.07544479	ENSG00000105426
GO:0035902	response to immobilization stress	BP	1	0.041317	0.07544479	ENSG00000132170

GO:0036336	dendritic cell migration	BP	1	0.041317	0.07544479	ENSG00000163464
GO:0045737	positive regulation of cyclin-dependent protein serine/threonine kinase activity	BP	1	0.041317	0.07544479	ENSG00000146648
GO:0051123	RNA polymerase II preinitiation complex assembly	BP	1	0.041317	0.07544479	ENSG00000091831
GO:0051194	positive regulation of cofactor metabolic process	BP	1	0.041317	0.07544479	ENSG00000171105
GO:0071168	protein localization to chromatin	BP	1	0.041317	0.07544479	ENSG00000091831
GO:2000144	positive regulation of DNA-templated transcription, initiation	BP	1	0.041317	0.07544479	ENSG00000091831
GO:0042886	amide transport	BP	7	0.041325	0.07544479	ENSG00000007171;ENSG00000073756;ENSG00000085563;ENSG00000104267;ENSG00000132170;ENSG00000146648;ENSG00000170890
GO:0019222	regulation of metabolic process	BP	16	0.042053	0.07671593	ENSG00000007171;ENSG00000073756;ENSG00000091831;ENSG00000100823;ENSG00000100985;ENSG00000105976;ENSG00000106546;ENSG00000128052;ENSG00000132170;ENSG00000143365;ENSG00000146648;ENSG00000149968;ENSG00000170890;ENSG00000171105;ENSG00000180210;ENSG00000186951
GO:0071900	regulation of protein serine/threonine kinase activity	BP	3	0.042357	0.0772134	ENSG00000146648;ENSG00000170890;ENSG00000171105
GO:0008406	gonad development	BP	2	0.042414	0.07725779	ENSG00000091831;ENSG00000171105
GO:0007202	activation of phospholipase C activity	BP	1	0.042712	0.07745086	ENSG00000146648
GO:0019430	removal of superoxide radicals	BP	1	0.042712	0.07745086	ENSG00000005381
GO:0035025	positive regulation of Rho protein signal transduction	BP	1	0.042712	0.07745086	ENSG00000178623
GO:0048679	regulation of axon regeneration	BP	1	0.042712	0.07745086	ENSG00000105426
GO:0060260	regulation of transcription initiation from RNA polymerase II promoter	BP	1	0.042712	0.07745086	ENSG00000091831
GO:1900016	negative regulation of cytokine production involved in inflammatory response	BP	1	0.042712	0.07745086	ENSG00000180210
GO:0006091	generation of precursor metabolites and energy	BP	3	0.043343	0.0784776	ENSG00000007171;ENSG00000171105;ENSG00000186951
GO:0050708	regulation of protein secretion	BP	3	0.043343	0.0784776	ENSG00000007171;ENSG00000146648;ENSG00000170890
GO:1903506	regulation of nucleic acid-templated transcription	BP	10	0.043673	0.0789161	ENSG00000091831;ENSG00000100823;ENSG00000105976;ENSG00000106546;ENSG00000132170;ENSG00000143365;ENSG00000146648;ENSG00000170890;ENSG00000171105;ENSG00000186951
GO:0090276	regulation of peptide hormone secretion	BP	2	0.043776	0.0789161	ENSG00000007171;ENSG00000146648
GO:0033043	regulation of organelle organization	BP	5	0.043878	0.0789161	ENSG00000100985;ENSG00000105426;ENSG00000105976;ENSG00000128052;ENSG00000171105
GO:0010543	regulation of platelet activation	BP	1	0.044105	0.0789161	ENSG00000180210

GO:0032232	negative regulation of actin filament bundle assembly	BP	1	0.044105	0.0789161	ENSG00000105976
GO:0042953	lipoprotein transport	BP	1	0.044105	0.0789161	ENSG00000132170
GO:0044872	lipoprotein localization	BP	1	0.044105	0.0789161	ENSG00000132170
GO:0045672	positive regulation of osteoclast differentiation	BP	1	0.044105	0.0789161	ENSG00000104267
GO:0045742	positive regulation of epidermal growth factor receptor signaling pathway	BP	1	0.044105	0.0789161	ENSG00000100985
GO:0072538	T-helper 17 type immune response	BP	1	0.044105	0.0789161	ENSG00000143365
GO:0072606	interleukin-8 secretion	BP	1	0.044105	0.0789161	ENSG00000007171
GO:1901661	quinone metabolic process	BP	1	0.044105	0.0789161	ENSG00000198074
GO:1903205	regulation of hydrogen peroxide-induced cell death	BP	1	0.044105	0.0789161	ENSG00000105976
GO:1903393	positive regulation of adherens junction organization	BP	1	0.044105	0.0789161	ENSG00000128052
GO:0002221	pattern recognition receptor signaling pathway	BP	2	0.044119	0.0789161	ENSG00000091831;ENSG00000105426
GO:0050920	regulation of chemotaxis	BP	2	0.044119	0.0789161	ENSG00000105976;ENSG00000128052
GO:1903827	regulation of cellular protein localization	BP	3	0.04414	0.0789161	ENSG00000073756;ENSG00000146648;ENSG00000180210
GO:0009987	cellular process	BP	29	0.044337	0.0791658	ENSG00000005381;ENSG00000007171;ENSG00000073756;ENSG00000085563;ENSG00000087245;ENSG00000091831;ENSG00000095303;ENSG00000100823;ENSG00000100985;ENSG00000104267;ENSG00000105426;ENSG00000105976;ENSG00000106546;ENSG00000118777;ENSG00000128052;ENSG00000132170;ENSG00000133742;ENSG00000143365;ENSG00000146648;ENSG00000148680;ENSG00000149968;ENSG00000159228;ENSG00000163464;ENSG00000170890;ENSG00000171105:ENSG00000178623:ENSG00000180210:ENSG00000186951:ENSG00000198074
GO:0060341	regulation of cellular localization	BP	4	0.044345	0.0791658	ENSG00000073756;ENSG00000146648;ENSG00000170890;ENSG00000180210
GO:0045137	development of primary sexual characteristics	BP	2	0.044464	0.07931966	ENSG00000091831;ENSG00000171105
GO:0006464	cellular protein modification process	BP	11	0.04455	0.07935681	ENSG00000007171;ENSG00000073756;ENSG00000091831;ENSG00000100985;ENSG00000105426;ENSG00000105976;ENSG00000128052;ENSG00000146648;ENSG00000170890;ENSG00000171105;ENSG00000180210
GO:0036211	protein modification process	BP	11	0.04455	0.07935681	ENSG00000007171;ENSG00000073756;ENSG00000091831;ENSG00000100985;ENSG00000105426;ENSG00000105976;ENSG00000128052;ENSG00000146648;ENSG00000170890;ENSG00000171105;ENSG00000180210
GO:2001141	regulation of RNA biosynthetic process	BP	10	0.044633	0.07939682	ENSG00000091831;ENSG00000100823;ENSG00000105976;ENSG00000106546;ENSG00000132170;ENSG00000143365;ENSG00000146648;ENSG00000170890;ENSG00000171105;ENSG00000186951
GO:0043549	regulation of kinase activity	BP	4	0.044638	0.07939682	ENSG00000146648;ENSG00000170890;ENSG00000171105;ENSG00000180210
GO:0006357	regulation of transcription by RNA polymerase II	BP	8	0.044869	0.07970336	ENSG00000091831;ENSG00000105976;ENSG00000106546;ENSG00000132170;ENSG00000143365;ENSG00000146648;ENSG00000170890;ENSG00000186951
GO:0016055	Wnt signaling pathway	BP	3	0.045146	0.07970336	ENSG00000091831;ENSG00000105976;ENSG00000146648

GO:0015893	drug transport	BP	2	0.045155	0.07970336	ENSG00000104267;ENSG00000118777
GO:0019219	regulation of nucleobase-containing compound metabolic process	BP	11	0.0454	0.07970336	ENSG00000007171;ENSG00000091831;ENSG00000100823;ENSG00000105976;ENSG00000106546;ENSG00000132170;ENSG00000143365;ENSG00000146648;ENSG00000170890;ENSG00000171105;ENSG00000186951
GO:0003401	axis elongation	BP	1	0.045496	0.07970336	ENSG00000091831
GO:0006658	phosphatidylserine metabolic process	BP	1	0.045496	0.07970336	ENSG00000170890
GO:0033198	response to ATP	BP	1	0.045496	0.07970336	ENSG00000073756
GO:0038128	ERBB2 signaling pathway	BP	1	0.045496	0.07970336	ENSG00000146648
GO:0042744	hydrogen peroxide catabolic process	BP	1	0.045496	0.07970336	ENSG00000005381
GO:0042759	long-chain fatty acid biosynthetic process	BP	1	0.045496	0.07970336	ENSG00000073756
GO:0050482	arachidonic acid secretion	BP	1	0.045496	0.07970336	ENSG00000170890
GO:0051482	positive regulation of cytosolic calcium ion concentration involved in phospholipase C-activating G protein-	BP	1	0.045496	0.07970336	ENSG00000178623
GO:0055022	negative regulation of cardiac muscle tissue growth	BP	1	0.045496	0.07970336	ENSG00000186951
GO:0061117	negative regulation of heart growth	BP	1	0.045496	0.07970336	ENSG00000186951
GO:0071295	cellular response to vitamin	BP	1	0.045496	0.07970336	ENSG00000132170
GO:0071450	cellular response to oxygen radical	BP	1	0.045496	0.07970336	ENSG00000005381
GO:0071451	cellular response to superoxide	BP	1	0.045496	0.07970336	ENSG00000005381
GO:0097242	amyloid-beta clearance	BP	1	0.045496	0.07970336	ENSG00000171105
GO:1903963	arachidonate transport	BP	1	0.045496	0.07970336	ENSG00000170890
GO:2001171	positive regulation of ATP biosynthetic process	BP	1	0.045496	0.07970336	ENSG00000171105
GO:1904018	positive regulation of vasculature development	BP	2	0.045502	0.07970336	ENSG00000073756;ENSG00000128052
GO:0198738	cell-cell signaling by wnt	BP	3	0.045552	0.07970923	ENSG00000091831;ENSG00000105976;ENSG00000146648
GO:0033036	macromolecule localization	BP	9	0.045572	0.07970923	ENSG00000007171;ENSG00000073756;ENSG00000085563;ENSG00000091831;ENSG00000132170;ENSG00000146648;ENSG00000170890;ENSG00000180210;ENSG00000186951
GO:0010556	regulation of macromolecule biosynthetic process	BP	11	0.045664	0.07981373	ENSG00000091831;ENSG00000100823;ENSG00000105976;ENSG00000106546;ENSG00000128052;ENSG00000132170;ENSG00000143365;ENSG00000146648;ENSG00000170890;ENSG00000171105;ENSG00000186951

GO:0045087	innate immune response	BP	5	0.046507	0.08095206	ENSG00000007171;ENSG00000091831;ENSG00000105426;ENSG00000132170;ENSG00000170890
GO:0044242	cellular lipid catabolic process	BP	2	0.04655	0.08095206	ENSG00000186951;ENSG00000198074
GO:0048588	developmental cell growth	BP	2	0.04655	0.08095206	ENSG00000105426;ENSG00000186951
GO:0045088	regulation of innate immune response	BP	3	0.04678	0.08095206	ENSG00000091831;ENSG00000105426;ENSG00000132170
GO:0006691	leukotriene metabolic process	BP	1	0.046885	0.08095206	ENSG00000170890
GO:0010574	regulation of vascular endothelial growth factor production	BP	1	0.046885	0.08095206	ENSG00000073756
GO:0032647	regulation of interferon-alpha production	BP	1	0.046885	0.08095206	ENSG00000105426
GO:0034694	response to prostaglandin	BP	1	0.046885	0.08095206	ENSG00000132170
GO:0035510	DNA dealkylation	BP	1	0.046885	0.08095206	ENSG00000100823
GO:0045745	positive regulation of G protein-coupled receptor signaling pathway	BP	1	0.046885	0.08095206	ENSG00000180210
GO:0045987	positive regulation of smooth muscle contraction	BP	1	0.046885	0.08095206	ENSG00000073756
GO:0055094	response to lipoprotein particle	BP	1	0.046885	0.08095206	ENSG00000132170
GO:0060325	face morphogenesis	BP	1	0.046885	0.08095206	ENSG00000087245
GO:0090200	positive regulation of release of cytochrome c from mitochondria	BP	1	0.046885	0.08095206	ENSG00000100985
GO:0099068	postsynapse assembly	BP	1	0.046885	0.08095206	ENSG00000105426
GO:1901186	positive regulation of ERBB signaling pathway	BP	1	0.046885	0.08095206	ENSG00000100985
GO:1904031	positive regulation of cyclin-dependent protein kinase activity	BP	1	0.046885	0.08095206	ENSG00000146648
GO:0045860	positive regulation of protein kinase activity	BP	3	0.047194	0.08142735	ENSG00000146648;ENSG00000170890;ENSG00000171105
GO:0044267	cellular protein metabolic process	BP	13	0.047362	0.08165982	ENSG00000007171;ENSG00000073756;ENSG00000087245;ENSG00000091831;ENSG00000100985;ENSG00000105426;ENSG00000105976;ENSG00000128052;ENSG00000132170;ENSG00000146648;ENSG00000170890;ENSG00000171105;ENSG00000180210
GO:1903047	mitotic cell cycle process	BP	4	0.047482	0.08180891	ENSG00000085563;ENSG00000100823;ENSG00000146648;ENSG00000171105
GO:0060255	regulation of macromolecule metabolic process	BP	15	0.047573	0.08184907	ENSG00000007171;ENSG00000073756;ENSG00000091831;ENSG00000100823;ENSG00000100985;ENSG00000105976;ENSG00000106546;ENSG00000128052;ENSG00000132170;ENSG00000143365;ENSG00000146648;ENSG00000170890;ENSG00000171105;ENSG00000180210;ENSG00000186951
GO:0001894	tissue homeostasis	BP	2	0.047607	0.08184907	ENSG00000104267;ENSG00000146648

GO:0060560	developmental growth involved in morphogenesis	BP	2	0.047607	0.08184907	ENSG00000091831;ENSG00000105426
GO:0032268	regulation of cellular protein metabolic process	BP	8	0.047763	0.08205808	ENSG00000073756;ENSG00000100985;ENSG00000128052;ENSG00000132170;ENSG00000146648;ENSG00000170890;ENSG00000171105;ENSG00000180210
GO:0030001	metal ion transport	BP	4	0.047941	0.08223182	ENSG00000073756;ENSG00000170890;ENSG00000178623;ENSG00000180210
GO:0031669	cellular response to nutrient levels	BP	2	0.047961	0.08223182	ENSG00000073756;ENSG00000132170
GO:0003382	epithelial cell morphogenesis	BP	1	0.048272	0.08223182	ENSG00000105976
GO:0019228	neuronal action potential	BP	1	0.048272	0.08223182	ENSG00000178623
GO:0032148	activation of protein kinase B activity	BP	1	0.048272	0.08223182	ENSG00000171105
GO:0048147	negative regulation of fibroblast proliferation	BP	1	0.048272	0.08223182	ENSG00000132170
GO:0070570	regulation of neuron projection regeneration	BP	1	0.048272	0.08223182	ENSG00000105426
GO:0071549	cellular response to dexamethasone stimulus	BP	1	0.048272	0.08223182	ENSG00000146648
GO:0098664	G protein-coupled serotonin receptor signaling pathway	BP	1	0.048272	0.08223182	ENSG00000148680
GO:2000352	negative regulation of endothelial cell apoptotic process	BP	1	0.048272	0.08223182	ENSG00000128052
GO:2001024	negative regulation of response to drug	BP	1	0.048272	0.08223182	ENSG00000105976
GO:2001038	regulation of cellular response to drug	BP	1	0.048272	0.08223182	ENSG00000105976
GO:0030162	regulation of proteolysis	BP	4	0.04871	0.08292025	ENSG00000073756;ENSG00000100985;ENSG00000132170;ENSG00000180210
GO:1901575	organic substance catabolic process	BP	7	0.048954	0.08323064	ENSG00000007171;ENSG00000100823;ENSG00000146648;ENSG00000170890;ENSG00000171105;ENSG00000186951;ENSG00000198074
GO:0030258	lipid modification	BP	2	0.04903	0.08323064	ENSG00000132170;ENSG00000186951
GO:0046434	organophosphate catabolic process	BP	2	0.04903	0.08323064	ENSG00000171105;ENSG00000186951
GO:0097529	myeloid leukocyte migration	BP	2	0.04903	0.08323064	ENSG00000163464;ENSG00000170890
GO:0002791	regulation of peptide secretion	BP	3	0.049499	0.08347451	ENSG00000007171;ENSG00000146648;ENSG00000170890
GO:0000303	response to superoxide	BP	1	0.049657	0.08347451	ENSG00000005381
GO:0007435	salivary gland morphogenesis	BP	1	0.049657	0.08347451	ENSG00000146648

GO:0010573	vascular endothelial growth factor production	BP	1	0.049657	0.08347451	ENSG00000073756
GO:0032228	regulation of synaptic transmission, GABAergic	BP	1	0.049657	0.08347451	ENSG00000104267
GO:0032607	interferon-alpha production	BP	1	0.049657	0.08347451	ENSG00000105426
GO:0034368	protein-lipid complex remodeling	BP	1	0.049657	0.08347451	ENSG00000005381
GO:0034369	plasma lipoprotein particle remodeling	BP	1	0.049657	0.08347451	ENSG00000005381
GO:0036314	response to sterol	BP	1	0.049657	0.08347451	ENSG00000143365
GO:0036474	cell death in response to hydrogen peroxide	BP	1	0.049657	0.08347451	ENSG00000105976
GO:0046471	phosphatidylglycerol metabolic process	BP	1	0.049657	0.08347451	ENSG00000170890
GO:0060603	mammary gland duct morphogenesis	BP	1	0.049657	0.08347451	ENSG00000091831
GO:0070873	regulation of glycogen metabolic process	BP	1	0.049657	0.08347451	ENSG00000171105
GO:0071402	cellular response to lipoprotein particle stimulus	BP	1	0.049657	0.08347451	ENSG00000132170
GO:0005576	extracellular region	CC	17	7.04E-05	0.01691121	ENSG00000005381;ENSG00000085563;ENSG00000087245;ENSG00000095303;ENSG00000100985;ENSG00000104267;ENSG00000105426;ENSG00000105976;ENSG00000128052;ENSG00000133742;ENSG00000146648;ENSG00000149968;ENSG00000159228;ENSG00000170890;ENSG00000171105;ENSG00000180210;ENSG00000198074
GO:0031982	vesicle	CC	15	0.00017	0.01691121	ENSG00000005381;ENSG00000007171;ENSG00000085563;ENSG00000095303;ENSG00000100985;ENSG00000104267;ENSG00000105426;ENSG00000128052;ENSG00000133742;ENSG00000146648;ENSG00000159228;ENSG00000163464;ENSG00000170890;ENSG00000171105;ENSG00000180210
GO:0043235	receptor complex	CC	5	0.000234	0.01691121	ENSG00000105976;ENSG00000106546;ENSG00000128052;ENSG00000146648;ENSG00000171105
GO:0005615	extracellular space	CC	14	0.000271	0.01691121	ENSG00000005381;ENSG00000085563;ENSG00000087245;ENSG00000095303;ENSG00000100985;ENSG00000104267;ENSG00000105426;ENSG00000133742;ENSG00000146648;ENSG00000149968;ENSG00000159228;ENSG00000170890;ENSG00000171105;ENSG00000180210
GO:0044421	extracellular region part	CC	14	0.000478	0.01895336	ENSG00000005381;ENSG00000085563;ENSG00000087245;ENSG00000095303;ENSG00000100985;ENSG00000104267;ENSG00000105426;ENSG00000133742;ENSG00000146648;ENSG00000149968;ENSG00000159228;ENSG00000170890;ENSG00000171105;ENSG00000180210
GO:0009986	cell surface	CC	7	0.000535	0.01895336	ENSG00000085563;ENSG00000105976;ENSG00000146648;ENSG00000163464;ENSG00000170890;ENSG00000171105;ENSG00000180210
GO:0070062	extracellular exosome	CC	10	0.000785	0.01895336	ENSG00000005381;ENSG00000085563;ENSG00000095303;ENSG00000100985;ENSG00000104267;ENSG00000105426;ENSG00000133742;ENSG00000159228;ENSG00000171105;ENSG00000180210
GO:1903561	extracellular vesicle	CC	10	0.000853	0.01895336	ENSG00000005381;ENSG00000085563;ENSG00000095303;ENSG00000100985;ENSG00000104267;ENSG00000105426;ENSG00000133742;ENSG00000159228;ENSG00000171105;ENSG00000180210
GO:0043230	extracellular organelle	CC	10	0.000858	0.01895336	ENSG00000005381;ENSG00000085563;ENSG00000095303;ENSG00000100985;ENSG00000104267;ENSG00000105426;ENSG00000133742;ENSG00000159228;ENSG00000171105;ENSG00000180210
GO:0044459	plasma membrane part	CC	12	0.000921	0.01895336	ENSG00000073756;ENSG00000085563;ENSG00000104267;ENSG00000105426;ENSG00000105976;ENSG00000128052;ENSG00000146648;ENSG00000148680;ENSG00000163464;ENSG00000171105;ENSG00000178623;ENSG00000180210
GO:0045121	membrane raft	CC	4	0.001044	0.01895336	ENSG00000073756;ENSG00000128052;ENSG00000146648;ENSG00000171105

GO:0098857	membrane microdomain	CC	4	0.001055	0.01895336	ENSG00000073756;ENSG00000128052;ENSG00000146648;ENSG00000171105
GO:0009925	basal plasma membrane	CC	2	0.001163	0.01895336	ENSG00000105976;ENSG00000146648
GO:0098589	membrane region	CC	4	0.001202	0.01895336	ENSG00000073756;ENSG00000128052;ENSG00000146648;ENSG00000171105
GO:0098590	plasma membrane region	CC	7	0.001355	0.01895336	ENSG00000073756;ENSG00000085563;ENSG00000104267;ENSG00000105426;ENSG00000105976;ENSG00000146648;ENSG00000171105
GO:0034752	cytosolic aryl hydrocarbon receptor complex	CC	1	0.001365	0.01895336	ENSG00000106546
GO:0044797	cytoplasmic transcription factor complex	CC	1	0.001365	0.01895336	ENSG00000106546
GO:0097489	multivesicular body, internal vesicle lumen	CC	1	0.001365	0.01895336	ENSG00000146648
GO:0005667	transcription factor complex	CC	4	0.00176	0.0231606	ENSG00000100823;ENSG00000106546;ENSG00000132170;ENSG00000186951
GO:0045178	basal part of cell	CC	2	0.002462	0.03077966	ENSG00000105976;ENSG00000146648
GO:0070435	Shc-EGFR complex	CC	1	0.002727	0.03247006	ENSG00000146648
GO:0016323	basolateral plasma membrane	CC	3	0.003715	0.04221327	ENSG00000104267;ENSG00000105976;ENSG00000146648
GO:0044444	cytoplasmic part	CC	22	0.004181	0.04544464	ENSG00000005381;ENSG00000007171;ENSG00000073756;ENSG00000087245;ENSG00000091831;ENSG00000095303;ENSG00000100823;ENSG00000100985;ENSG00000104267;ENSG00000105426;ENSG00000106546;ENSG00000118777;ENSG00000128052;ENSG00000132170;ENSG00000133742;ENSG00000146648;ENSG00000159228;ENSG00000163464;ENSG00000170890;ENSG00000171105;ENSG00000180210;ENSG00000198074
GO:0005886	plasma membrane	CC	16	0.004949	0.05044247	ENSG00000007171;ENSG00000073756;ENSG00000085563;ENSG00000087245;ENSG00000091831;ENSG00000104267;ENSG00000105426;ENSG00000105976;ENSG00000118777;ENSG00000128052;ENSG00000146648;ENSG00000148680;ENSG00000163464;ENSG00000171105;ENSG00000178623;ENSG00000180210
GO:0034751	aryl hydrocarbon receptor complex	CC	1	0.005448	0.05044247	ENSG00000106546
GO:0097013	phagocytic vesicle lumen	CC	1	0.005448	0.05044247	ENSG00000005381
GO:0097487	multivesicular body, internal vesicle	CC	1	0.005448	0.05044247	ENSG00000146648
GO:0071944	cell periphery	CC	16	0.00606	0.05289348	ENSG00000007171;ENSG00000073756;ENSG00000085563;ENSG00000087245;ENSG00000091831;ENSG00000104267;ENSG00000105426;ENSG00000105976;ENSG00000118777;ENSG00000128052;ENSG00000146648;ENSG00000148680;ENSG00000163464;ENSG00000171105;ENSG00000178623;ENSG00000180210
GO:0005901	caveola	CC	2	0.006261	0.05289348	ENSG00000073756;ENSG00000171105
GO:0031012	extracellular matrix	CC	4	0.006347	0.05289348	ENSG00000087245;ENSG00000100985;ENSG00000149968;ENSG00000180210
GO:0005899	insulin receptor complex	CC	1	0.006805	0.05488105	ENSG00000171105
GO:0097443	sorting endosome	CC	1	0.008161	0.06375722	ENSG00000128052
GO:0043227	membrane-bounded organelle	CC	25	0.008566	0.06489109	ENSG00000005381;ENSG00000007171;ENSG00000073756;ENSG00000085563;ENSG00000087245;ENSG00000091831;ENSG00000095303;ENSG00000100823;ENSG00000100985;ENSG00000104267;ENSG00000105426;ENSG00000106546;ENSG00000118777;ENSG00000128052;ENSG00000132170;ENSG00000133742;ENSG00000143365;ENSG00000146648;ENSG00000159228;ENSG00000163464;ENSG00000170890;ENSG00000171105;ENSG00000180210;ENSG00000186951;ENSG00000198074

GO:0012505	endomembrane system	CC	13	0.00919	0.06607508	ENSG00000005381;ENSG00000073756;ENSG00000091831;ENSG00000095303;ENSG00000100823;ENSG00000100985;ENSG00000105426;ENSG00000128052;ENSG00000146648;ENSG00000163464;ENSG00000170890;ENSG00000171105;ENSG00000180210
GO:0043005	neuron projection	CC	6	0.009387	0.06607508	ENSG00000073756;ENSG00000095303;ENSG00000104267;ENSG00000105426;ENSG00000148680;ENSG00000171105
GO:0097550	transcriptional preinitiation complex	CC	1	0.009515	0.06607508	ENSG00000091831
GO:0044853	plasma membrane raft	CC	2	0.011073	0.07482009	ENSG00000073756;ENSG00000171105
GO:0005887	integral component of plasma membrane	CC	7	0.011513	0.07574667	ENSG00000105426;ENSG00000105976;ENSG00000128052;ENSG00000146648;ENSG00000148680;ENSG00000171105;ENSG00000178623
GO:0031226	intrinsic component of plasma membrane	CC	7	0.014368	0.09210466	ENSG00000105426;ENSG00000105976;ENSG00000128052;ENSG00000146648;ENSG00000148680;ENSG00000171105;ENSG00000178623
GO:0099503	secretory vesicle	CC	5	0.01596	0.09974808	ENSG00000005381;ENSG00000100985;ENSG00000105426;ENSG00000163464;ENSG00000170890
GO:0045177	apical part of cell	CC	3	0.017507	0.1067506	ENSG00000085563;ENSG00000104267;ENSG00000146648
GO:0048471	perinuclear region of cytoplasm	CC	4	0.018287	0.108853	ENSG00000007171;ENSG00000100823;ENSG00000132170;ENSG00000146648
GO:0062023	collagen-containing extracellular matrix	CC	3	0.019745	0.11479684	ENSG00000087245;ENSG00000100985;ENSG00000180210
GO:0031410	cytoplasmic vesicle	CC	8	0.021083	0.1178712	ENSG00000005381;ENSG00000100985;ENSG00000105426;ENSG00000128052;ENSG00000146648;ENSG00000163464;ENSG00000170890;ENSG00000171105
GO:0097708	intracellular vesicle	CC	8	0.021217	0.1178712	ENSG00000005381;ENSG00000100985;ENSG00000105426;ENSG00000128052;ENSG00000146648;ENSG00000163464;ENSG00000170890;ENSG00000171105
GO:0090575	RNA polymerase II transcription factor complex	CC	2	0.024679	0.13412673	ENSG00000132170;ENSG00000186951
GO:0071682	endocytic vesicle lumen	CC	1	0.028283	0.15044092	ENSG00000005381
GO:0044433	cytoplasmic vesicle part	CC	6	0.029132	0.15172955	ENSG00000005381;ENSG00000100985;ENSG00000105426;ENSG00000146648;ENSG00000163464;ENSG00000171105
GO:0031974	membrane-enclosed lumen	CC	13	0.031269	0.15327909	ENSG00000005381;ENSG00000007171;ENSG00000073756;ENSG00000091831;ENSG00000100823;ENSG00000100985;ENSG00000106546;ENSG00000132170;ENSG00000143365;ENSG00000146648;ENSG00000171105;ENSG00000180210;ENSG00000186951
GO:0043233	organelle lumen	CC	13	0.031269	0.15327909	ENSG00000005381;ENSG00000007171;ENSG00000073756;ENSG00000091831;ENSG00000100823;ENSG00000100985;ENSG00000106546;ENSG00000132170;ENSG00000143365;ENSG00000146648;ENSG00000171105;ENSG00000180210;ENSG00000186951
GO:0070013	intracellular organelle lumen	CC	13	0.031269	0.15327909	ENSG00000005381;ENSG00000007171;ENSG00000073756;ENSG00000091831;ENSG00000100823;ENSG00000100985;ENSG00000106546;ENSG00000132170;ENSG00000143365;ENSG00000146648;ENSG00000171105;ENSG00000180210;ENSG00000186951
GO:0009897	external side of plasma membrane	CC	3	0.032582	0.15427	ENSG00000163464;ENSG00000171105;ENSG00000180210
GO:0097458	neuron part	CC	6	0.032705	0.15427	ENSG00000073756;ENSG00000095303;ENSG00000104267;ENSG00000105426;ENSG00000148680;ENSG00000171105
GO:0035327	transcriptionally active chromatin	CC	1	0.033582	0.15547142	ENSG00000091831
GO:0032809	neuronal cell body membrane	CC	1	0.036221	0.1600959	ENSG00000171105

GO:0044798	nuclear transcription factor complex	CC	2	0.036662	0.1600959	ENSG00000132170;ENSG00000186951
GO:0012506	vesicle membrane	CC	4	0.037123	0.1600959	ENSG00000007171;ENSG00000105426;ENSG00000146648;ENSG00000163464
GO:0044298	cell body membrane	CC	1	0.037538	0.1600959	ENSG00000171105
GO:0030141	secretory granule	CC	4	0.038248	0.1600959	ENSG00000005381;ENSG00000100985;ENSG00000163464;ENSG00000170890
GO:0043226	organelle	CC	25	0.038423	0.1600959	ENSG00000005381;ENSG00000007171;ENSG00000073756;ENSG00000085563;ENSG00000087245;ENSG00000091831;ENSG00000095303;ENSG00000100823;ENSG00000100985;ENSG00000104267;ENSG00000105426;ENSG00000106546;ENSG00000118777;ENSG00000128052;ENSG00000132170;ENSG00000133742;ENSG00000143365;ENSG00000146648;ENSG00000159228;ENSG00000163464;ENSG00000170890;ENSG00000171105;ENSG00000180210;ENSG00000186951;ENSG00000198074
GO:0098805	whole membrane	CC	6	0.042982	0.17615432	ENSG00000073756;ENSG00000105426;ENSG00000128052;ENSG00000146648;ENSG00000163464;ENSG00000171105
GO:0030285	integral component of synaptic vesicle membrane	CC	1	0.04801	0.19359047	ENSG00000105426
GO:0004879	nuclear receptor activity	MF	5	1.05E-08	1.76E-06	ENSG00000091831;ENSG00000106546;ENSG00000132170;ENSG00000143365;ENSG00000186951
GO:0098531	transcription factor activity, direct ligand regulated sequence-specific DNA binding	MF	5	1.05E-08	1.76E-06	ENSG00000091831;ENSG00000106546;ENSG00000132170;ENSG00000143365;ENSG00000186951
GO:0001223	transcription coactivator binding	MF	4	2.46E-08	2.74E-06	ENSG00000091831;ENSG00000106546;ENSG00000143365;ENSG00000186951
GO:0001221	transcription cofactor binding	MF	4	4.07E-07	3.40E-05	ENSG00000091831;ENSG00000106546;ENSG00000143365;ENSG00000186951
GO:0043167	ion binding	MF	23	6.59E-07	4.40E-05	ENSG00000005381;ENSG00000007171;ENSG00000073756;ENSG00000085563;ENSG00000087245;ENSG00000091831;ENSG00000095303;ENSG00000100823;ENSG00000100985;ENSG00000104267;ENSG00000105426;ENSG00000105976;ENSG00000118777;ENSG00000128052;ENSG00000132170;ENSG00000133742;ENSG00000143365;ENSG00000146648;ENSG00000149968;ENSG00000170890;ENSG00000171105;ENSG00000180210;ENSG00000186951
GO:0003707	steroid hormone receptor activity	MF	4	1.69E-06	9.41E-05	ENSG00000091831;ENSG00000132170;ENSG00000143365;ENSG00000186951
GO:0004666	prostaglandin-endoperoxide synthase activity	MF	2	1.99E-06	9.50E-05	ENSG00000073756;ENSG00000095303
GO:0004714	transmembrane receptor protein tyrosine kinase activity	MF	4	3.30E-06	0.00013335	ENSG00000105976;ENSG00000128052;ENSG00000146648;ENSG00000171105
GO:0008270	zinc ion binding	MF	9	3.86E-06	0.00013335	ENSG00000087245;ENSG00000091831;ENSG00000100985;ENSG00000104267;ENSG00000132170;ENSG00000133742;ENSG00000143365;ENSG00000149968;ENSG00000186951
GO:0038023	signaling receptor activity	MF	12	3.99E-06	0.00013335	ENSG00000091831;ENSG00000105976;ENSG00000128052;ENSG00000132170;ENSG00000143365;ENSG00000146648;ENSG00000148680;ENSG00000163464;ENSG00000171105;ENSG00000178623;ENSG00000180210;ENSG00000186951
GO:0060089	molecular transducer activity	MF	12	5.39E-06	0.00016374	ENSG00000091831;ENSG00000105976;ENSG00000128052;ENSG00000132170;ENSG00000143365;ENSG00000146648;ENSG00000148680;ENSG00000163464;ENSG00000171105;ENSG00000178623;ENSG00000180210;ENSG00000186951
GO:0003824	catalytic activity	MF	21	9.36E-06	0.00026064	ENSG00000005381;ENSG00000007171;ENSG00000073756;ENSG00000085563;ENSG00000087245;ENSG00000095303;ENSG00000100823;ENSG00000100985;ENSG00000104267;ENSG00000105426;ENSG00000105976;ENSG00000118777;ENSG00000128052;ENSG00000133742;ENSG00000146648;ENSG00000149968;ENSG00000159228;ENSG00000170890;ENSG00000171105;ENSG00000180210;ENSG00000198074
GO:0019199	transmembrane receptor protein kinase activity	MF	4	1.04E-05	0.00026708	ENSG00000105976;ENSG00000128052;ENSG00000146648;ENSG00000171105
GO:0004064	arylesterase activity	MF	2	2.98E-05	0.00070999	ENSG00000104267;ENSG00000133742
GO:0046914	transition metal ion binding	MF	9	3.48E-05	0.00077558	ENSG00000087245;ENSG00000091831;ENSG00000100985;ENSG00000104267;ENSG00000132170;ENSG00000133742;ENSG00000143365;ENSG00000149968;ENSG00000186951

GO:0001085	RNA polymerase II transcription factor binding	MF	4	4.72E-05	0.00098569	ENSG00000091831;ENSG00000106546;ENSG00000132170;ENSG00000186951
GO:0016494	C-X-C chemokine receptor activity	MF	2	5.55E-05	0.00102896	ENSG00000163464;ENSG00000178623
GO:0030235	nitric-oxide synthase regulator activity	MF	2	5.55E-05	0.00102896	ENSG00000091831;ENSG00000146648
GO:0004713	protein tyrosine kinase activity	MF	4	6.01E-05	0.00105645	ENSG00000105976;ENSG00000128052;ENSG00000146648;ENSG00000171105
GO:0004601	peroxidase activity	MF	3	7.39E-05	0.00122828	ENSG00000005381;ENSG00000073756;ENSG00000095303
GO:0020037	heme binding	MF	4	7.72E-05	0.00122828	ENSG00000005381;ENSG00000007171;ENSG00000073756;ENSG00000095303
GO:0016684	oxidoreductase activity, acting on peroxide as acceptor	MF	3	9.51E-05	0.00141832	ENSG00000005381;ENSG00000073756;ENSG00000095303
GO:0046906	tetrapyrrole binding	MF	4	9.77E-05	0.00141832	ENSG00000005381;ENSG00000007171;ENSG00000073756;ENSG00000095303
GO:0033293	monocarboxylic acid binding	MF	3	0.00012	0.00166853	ENSG00000132170;ENSG00000170890;ENSG00000186951
GO:0043168	anion binding	MF	13	0.000129	0.00167708	ENSG00000005381;ENSG00000007171;ENSG00000085563;ENSG00000105426;ENSG00000105976;ENSG00000118777;ENSG00000128052;ENSG00000132170;ENSG00000146648;ENSG00000170890;ENSG00000171105;ENSG00000180210;ENSG00000186951
GO:0016491	oxidoreductase activity	MF	7	0.000131	0.00167708	ENSG00000005381;ENSG00000007171;ENSG00000073756;ENSG00000095303;ENSG00000100823;ENSG00000159228;ENSG00000198074
GO:0046872	metal ion binding	MF	16	0.000136	0.00167708	ENSG00000005381;ENSG00000007171;ENSG00000073756;ENSG00000087245;ENSG00000091831;ENSG00000095303;ENSG00000100823;ENSG00000100985;ENSG00000104267;ENSG00000132170;ENSG00000133742;ENSG00000143365;ENSG00000149968;ENSG00000170890;ENSG00000180210;ENSG00000186951
GO:0004252	serine-type endopeptidase activity	MF	4	0.000153	0.00178245	ENSG00000087245;ENSG00000100985;ENSG00000149968;ENSG00000180210
GO:0016835	carbon-oxygen lyase activity	MF	3	0.000155	0.00178245	ENSG00000100823;ENSG00000104267;ENSG00000133742
GO:0043169	cation binding	MF	16	0.000176	0.001904	ENSG00000005381;ENSG00000007171;ENSG00000073756;ENSG00000087245;ENSG00000091831;ENSG00000095303;ENSG00000100823;ENSG00000100985;ENSG00000104267;ENSG00000132170;ENSG00000133742;ENSG00000143365;ENSG00000149968;ENSG00000170890;ENSG00000180210;ENSG00000186951
GO:0042802	identical protein binding	MF	10	0.000177	0.001904	ENSG00000007171;ENSG00000073756;ENSG00000091831;ENSG00000100985;ENSG00000105976;ENSG00000106546;ENSG00000118777;ENSG00000128052;ENSG00000132170;ENSG00000146648
GO:0019902	phosphatase binding	MF	4	0.000197	0.00205605	ENSG00000105976;ENSG00000132170;ENSG00000146648;ENSG00000186951
GO:0004089	carbonate dehydratase activity	MF	2	0.000207	0.0020916	ENSG00000104267;ENSG00000133742
GO:0031406	carboxylic acid binding	MF	4	0.000224	0.00220156	ENSG00000007171;ENSG00000132170;ENSG00000170890;ENSG00000186951
GO:0008236	serine-type peptidase activity	MF	4	0.000241	0.00229762	ENSG00000087245;ENSG00000100985;ENSG00000149968;ENSG00000180210
GO:0017171	serine hydrolase activity	MF	4	0.000263	0.00243841	ENSG00000087245;ENSG00000100985;ENSG00000149968;ENSG00000180210
GO:0043177	organic acid binding	MF	4	0.000277	0.00249808	ENSG00000007171;ENSG00000132170;ENSG00000170890;ENSG00000186951

GO:0008134	transcription factor binding	MF	6	0.000375	0.00329171	ENSG00000091831;ENSG00000100823;ENSG00000106546;ENSG00000132170;ENSG00000143365;ENSG00000186951
GO:0016209	antioxidant activity	MF	3	0.000392	0.00335732	ENSG00000005381;ENSG00000073756;ENSG00000095303
GO:0001091	RNA polymerase II basal transcription factor binding	MF	2	0.000411	0.00343275	ENSG00000091831;ENSG00000106546
GO:0017025	TBP-class protein binding	MF	2	0.000494	0.00402759	ENSG00000091831;ENSG00000106546
GO:0003690	double-stranded DNA binding	MF	7	0.000511	0.00406133	ENSG00000091831;ENSG00000100823;ENSG00000106546;ENSG00000132170;ENSG00000143365;ENSG00000146648;ENSG00000186951
GO:0004222	metalloendopeptidase activity	MF	3	0.000575	0.00446755	ENSG00000087245;ENSG00000100985;ENSG00000149968
GO:0001637	G protein-coupled chemoattractant receptor activity	MF	2	0.000633	0.00467012	ENSG00000163464;ENSG00000178623
GO:0004950	chemokine receptor activity	MF	2	0.000633	0.00467012	ENSG00000163464;ENSG00000178623
GO:0008289	lipid binding	MF	6	0.000643	0.00467012	ENSG00000091831;ENSG00000132170;ENSG00000143365;ENSG00000170890;ENSG00000180210;ENSG00000186951
GO:0000978	RNA polymerase II proximal promoter sequence-specific DNA binding	MF	5	0.000952	0.00676619	ENSG00000091831;ENSG00000106546;ENSG00000132170;ENSG00000143365;ENSG00000186951
GO:0000987	proximal promoter sequence-specific DNA binding	MF	5	0.001067	0.00742535	ENSG00000091831;ENSG00000106546;ENSG00000132170;ENSG00000143365;ENSG00000186951
GO:0008144	drug binding	MF	9	0.001108	0.00755009	ENSG00000007171;ENSG00000085563;ENSG00000105976;ENSG00000118777;ENSG00000128052;ENSG00000132170;ENSG00000146648;ENSG00000171105;ENSG00000186951
GO:0001103	RNA polymerase II repressing transcription factor binding	MF	2	0.00115	0.00768453	ENSG00000132170;ENSG00000186951
GO:0005504	fatty acid binding	MF	2	0.001217	0.00796065	ENSG00000132170;ENSG00000186951
GO:0003682	chromatin binding	MF	5	0.001239	0.00796065	ENSG00000005381;ENSG00000091831;ENSG00000100823;ENSG00000132170;ENSG00000146648
GO:0019838	growth factor binding	MF	3	0.001274	0.00802685	ENSG00000128052;ENSG00000146648;ENSG00000171105
GO:0019903	protein phosphatase binding	MF	3	0.001323	0.00812921	ENSG00000105976;ENSG00000132170;ENSG00000146648
GO:0004888	transmembrane signaling receptor activity	MF	8	0.001395	0.00812921	ENSG00000105976;ENSG00000128052;ENSG00000132170;ENSG00000146648;ENSG00000148680;ENSG00000163464;ENSG00000171105;ENSG00000178623
GO:0005008	hepatocyte growth factor-activated receptor activity	MF	1	0.001436	0.00812921	ENSG00000105976
GO:0047021	15-hydroxyprostaglandin dehydrogenase (NADP+) activity	MF	1	0.001436	0.00812921	ENSG00000159228
GO:0050221	prostaglandin-E2 9-reductase activity	MF	1	0.001436	0.00812921	ENSG00000159228
GO:0099038	ceramide-translocating ATPase activity	MF	1	0.001436	0.00812921	ENSG00000085563

GO:0016787	hydrolase activity	MF	11	0.001462	0.00813956	ENSG00000085563;ENSG00000087245;ENSG00000100823;ENSG00000100985;ENSG00000104267;ENSG00000105426;ENSG00000118777;ENSG00000133742;ENSG00000149968;ENSG00000170890;ENSG00000180210
GO:0097367	carbohydrate derivative binding	MF	10	0.00152	0.00825819	ENSG00000005381;ENSG00000007171;ENSG00000085563;ENSG00000105426;ENSG00000105976;ENSG00000118777;ENSG00000128052;ENSG00000146648;ENSG00000171105;ENSG00000180210
GO:0052689	carboxylic ester hydrolase activity	MF	3	0.001533	0.00825819	ENSG00000104267;ENSG00000133742;ENSG00000170890
GO:0044877	protein-containing complex binding	MF	7	0.001608	0.00852513	ENSG00000100823;ENSG00000100985;ENSG00000106546;ENSG00000128052;ENSG00000146648;ENSG00000171105;ENSG00000186951
GO:0030331	estrogen receptor binding	MF	2	0.001654	0.00863356	ENSG00000091831;ENSG00000132170
GO:0051879	Hsp90 protein binding	MF	2	0.001897	0.00974833	ENSG00000106546;ENSG00000128052
GO:0008201	heparin binding	MF	3	0.002013	0.01018531	ENSG00000005381;ENSG00000105426;ENSG00000180210
GO:0016705	oxidoreductase activity, acting on paired donors, with incorporation or reduction of molecular	MF	3	0.002214	0.01103723	ENSG00000007171;ENSG00000073756;ENSG00000095303
GO:0070888	E-box binding	MF	2	0.002337	0.01147904	ENSG00000106546;ENSG00000132170
GO:0008237	metallopeptidase activity	MF	3	0.002732	0.01261293	ENSG00000087245;ENSG00000100985;ENSG00000149968
GO:0016836	hydro-lyase activity	MF	2	0.002821	0.01261293	ENSG00000104267;ENSG00000133742
GO:0004918	interleukin-8 receptor activity	MF	1	0.00287	0.01261293	ENSG00000163464
GO:0005006	epidermal growth factor-activated receptor activity	MF	1	0.00287	0.01261293	ENSG00000146648
GO:0008311	double-stranded DNA 3'-5' exodeoxyribonuclease activity	MF	1	0.00287	0.01261293	ENSG00000100823
GO:0016890	site-specific endodeoxyribonuclease activity, specific for altered base	MF	1	0.00287	0.01261293	ENSG00000100823
GO:0045550	geranylgeranyl reductase activity	MF	1	0.00287	0.01261293	ENSG00000198074
GO:0090555	phosphatidylethanolamine-translocating ATPase activity	MF	1	0.00287	0.01261293	ENSG00000085563
GO:0016829	lyase activity	MF	3	0.003143	0.01363498	ENSG00000100823;ENSG00000104267;ENSG00000133742
GO:0036094	small molecule binding	MF	10	0.003918	0.01614468	ENSG00000007171;ENSG00000085563;ENSG00000105976;ENSG00000118777;ENSG00000128052;ENSG00000132170;ENSG00000146648;ENSG00000170890;ENSG00000171105;ENSG00000186951
GO:0030374	nuclear receptor transcription coactivator activity	MF	2	0.004155	0.01614468	ENSG00000132170;ENSG00000186951
GO:0001093	TFIIB-class transcription factor binding	MF	1	0.004302	0.01614468	ENSG00000091831
GO:0004517	nitric-oxide synthase activity	MF	1	0.004302	0.01614468	ENSG00000007171

GO:0005009	insulin-activated receptor activity	MF	1	0.004302	0.01614468	ENSG00000171105
GO:0008559	xenobiotic transmembrane transporting ATPase activity	MF	1	0.004302	0.01614468	ENSG00000118777
GO:0019959	interleukin-8 binding	MF	1	0.004302	0.01614468	ENSG00000163464
GO:0047655	allyl-alcohol dehydrogenase activity	MF	1	0.004302	0.01614468	ENSG00000198074
GO:0047718	indanol dehydrogenase activity	MF	1	0.004302	0.01614468	ENSG00000198074
GO:0050692	DBD domain binding	MF	1	0.004302	0.01614468	ENSG00000132170
GO:0051425	PTB domain binding	MF	1	0.004302	0.01614468	ENSG00000171105
GO:1904121	phosphatidylethanolamine transporter activity	MF	1	0.004302	0.01614468	ENSG00000085563
GO:0001098	basal transcription machinery binding	MF	2	0.004653	0.01689349	ENSG00000091831;ENSG00000106546
GO:0001099	basal RNA polymerase II transcription machinery binding	MF	2	0.004653	0.01689349	ENSG00000091831;ENSG00000106546
GO:0070491	repressing transcription factor binding	MF	2	0.004653	0.01689349	ENSG00000132170;ENSG00000186951
GO:0001227	DNA-binding transcription repressor activity, RNA polymerase II-specific	MF	3	0.004934	0.01753053	ENSG00000132170;ENSG00000143365;ENSG00000186951
GO:0005539	glycosaminoglycan binding	MF	3	0.004934	0.01753053	ENSG00000005381;ENSG00000105426;ENSG00000180210
GO:0005102	signaling receptor binding	MF	8	0.00502	0.01764899	ENSG00000007171;ENSG00000091831;ENSG00000128052;ENSG00000132170;ENSG00000146648;ENSG00000170890;ENSG00000171105;ENSG00000180210
GO:0004175	endopeptidase activity	MF	4	0.005397	0.0180001	ENSG00000087245;ENSG00000100985;ENSG00000149968;ENSG00000180210
GO:0140096	catalytic activity, acting on a protein	MF	9	0.005486	0.0180001	ENSG00000087245;ENSG00000100985;ENSG00000105426;ENSG00000105976;ENSG00000128052;ENSG00000146648;ENSG00000149968;ENSG00000171105;ENSG00000180210
GO:0000977	RNA polymerase II regulatory region sequence-specific DNA binding	MF	5	0.005637	0.0180001	ENSG00000091831;ENSG00000106546;ENSG00000132170;ENSG00000143365;ENSG00000186951
GO:0004844	uracil DNA N-glycosylase activity	MF	1	0.005732	0.0180001	ENSG00000100823
GO:0008309	double-stranded DNA exodeoxyribonuclease activity	MF	1	0.005732	0.0180001	ENSG00000100823
GO:0034056	estrogen response element binding	MF	1	0.005732	0.0180001	ENSG00000091831
GO:0034617	tetrahydrobiopterin binding	MF	1	0.005732	0.0180001	ENSG00000007171
GO:0048408	epidermal growth factor binding	MF	1	0.005732	0.0180001	ENSG00000146648

GO:0070053	thrombospondin receptor activity	MF	1	0.005732	0.0180001	ENSG00000180210
GO:0090554	phosphatidylcholine-translocating ATPase activity	MF	1	0.005732	0.0180001	ENSG00000085563
GO:0097506	deaminated base DNA N-glycosylase activity	MF	1	0.005732	0.0180001	ENSG00000100823
GO:1901681	sulfur compound binding	MF	3	0.005766	0.0180001	ENSG00000005381;ENSG00000105426;ENSG00000180210
GO:0001012	RNA polymerase II regulatory region DNA binding	MF	5	0.005843	0.01806861	ENSG00000091831;ENSG00000106546;ENSG00000132170;ENSG00000143365;ENSG00000186951
GO:0016788	hydrolase activity, acting on ester bonds	MF	5	0.006084	0.01850021	ENSG00000100823;ENSG00000104267;ENSG00000105426;ENSG00000133742;ENSG00000170890
GO:0043565	sequence-specific DNA binding	MF	6	0.006093	0.01850021	ENSG00000091831;ENSG00000100823;ENSG00000106546;ENSG00000132170;ENSG00000143365;ENSG00000186951
GO:0008142	oxysterol binding	MF	1	0.00716	0.02061618	ENSG00000143365
GO:0030284	estrogen receptor activity	MF	1	0.00716	0.02061618	ENSG00000091831
GO:0042910	xenobiotic transmembrane transporter activity	MF	1	0.00716	0.02061618	ENSG00000118777
GO:0043559	insulin binding	MF	1	0.00716	0.02061618	ENSG00000171105
GO:0050544	arachidonic acid binding	MF	1	0.00716	0.02061618	ENSG00000132170
GO:0051525	NFAT protein binding	MF	1	0.00716	0.02061618	ENSG00000186951
GO:0000976	transcription regulatory region sequence-specific DNA binding	MF	5	0.007576	0.02162704	ENSG00000091831;ENSG00000106546;ENSG00000132170;ENSG00000143365;ENSG00000186951
GO:0048037	cofactor binding	MF	4	0.007672	0.02171539	ENSG00000005381;ENSG00000007171;ENSG00000073756;ENSG00000095303
GO:0051213	dioxygenase activity	MF	2	0.008185	0.02294228	ENSG00000073756;ENSG00000095303
GO:0035258	steroid hormone receptor binding	MF	2	0.00852	0.02294228	ENSG00000091831;ENSG00000132170
GO:0003958	NADPH-hemoprotein reductase activity	MF	1	0.008586	0.02294228	ENSG00000007171
GO:0004090	carbonyl reductase (NADPH) activity	MF	1	0.008586	0.02294228	ENSG00000159228
GO:0019958	C-X-C chemokine binding	MF	1	0.008586	0.02294228	ENSG00000163464
GO:0050542	icosanoid binding	MF	1	0.008586	0.02294228	ENSG00000132170
GO:0050543	icosatetraenoic acid binding	MF	1	0.008586	0.02294228	ENSG00000132170

GO:0004896	cytokine receptor activity	MF	2	0.008861	0.02348903	ENSG00000163464;ENSG00000178623
GO:0005496	steroid binding	MF	2	0.009208	0.02421716	ENSG00000091831;ENSG00000143365
GO:1990837	sequence-specific double-stranded DNA binding	MF	5	0.009309	0.02429149	ENSG00000091831;ENSG00000106546;ENSG00000132170;ENSG00000143365;ENSG00000186951
GO:0004528	phosphodiesterase I activity	MF	1	0.01001	0.02532902	ENSG00000100823
GO:0005021	vascular endothelial growth factor-activated receptor activity	MF	1	0.01001	0.02532902	ENSG00000128052
GO:0038085	vascular endothelial growth factor binding	MF	1	0.01001	0.02532902	ENSG00000128052
GO:0050693	LBD domain binding	MF	1	0.01001	0.02532902	ENSG00000132170
GO:0042562	hormone binding	MF	2	0.010471	0.02629489	ENSG00000146648;ENSG00000171105
GO:0001758	retinal dehydrogenase activity	MF	1	0.011432	0.0276697	ENSG00000198074
GO:0004523	RNA-DNA hybrid ribonuclease activity	MF	1	0.011432	0.0276697	ENSG00000100823
GO:0031995	insulin-like growth factor II binding	MF	1	0.011432	0.0276697	ENSG00000171105
GO:0035620	ceramide transporter activity	MF	1	0.011432	0.0276697	ENSG00000085563
GO:0097371	MDM2/MDM4 family protein binding	MF	1	0.011432	0.0276697	ENSG00000186951
GO:0016651	oxidoreductase activity, acting on NAD(P)H	MF	2	0.0122	0.02931488	ENSG00000007171;ENSG00000159228
GO:0003691	double-stranded telomeric DNA binding	MF	1	0.012853	0.02981074	ENSG00000100823
GO:0004955	prostaglandin receptor activity	MF	1	0.012853	0.02981074	ENSG00000132170
GO:0032052	bile acid binding	MF	1	0.012853	0.02981074	ENSG00000170890
GO:0034618	arginine binding	MF	1	0.012853	0.02981074	ENSG00000007171
GO:0035374	chondroitin sulfate binding	MF	1	0.012853	0.02981074	ENSG00000105426
GO:0044212	transcription regulatory region DNA binding	MF	5	0.013097	0.03016741	ENSG00000091831;ENSG00000106546;ENSG00000132170;ENSG00000143365;ENSG00000186951
GO:0001067	regulatory region nucleic acid binding	MF	5	0.013202	0.03020111	ENSG00000091831;ENSG00000106546;ENSG00000132170;ENSG00000143365;ENSG00000186951
GO:0003713	transcription coactivator activity	MF	3	0.01351	0.03069677	ENSG00000100823;ENSG00000132170;ENSG00000186951

GO:0046983	protein dimerization activity	MF	6	0.014001	0.03156568	ENSG00000007171;ENSG00000073756;ENSG00000106546;ENSG00000118777;ENSG00000132170;ENSG00000146648
GO:0004954	prostanoid receptor activity	MF	1	0.014271	0.03156568	ENSG00000132170
GO:0008525	phosphatidylcholine transporter activity	MF	1	0.014271	0.03156568	ENSG00000085563
GO:0140078	class I DNA-(apurinic or apyrimidinic site) endonuclease activity	MF	1	0.014271	0.03156568	ENSG00000100823
GO:1901363	heterocyclic compound binding	MF	16	0.014426	0.03169989	ENSG00000005381;ENSG00000007171;ENSG00000073756;ENSG00000085563;ENSG00000091831;ENSG00000095303;ENSG00000100823;ENSG00000105976;ENSG00000106546;ENSG00000118777;ENSG00000128052;ENSG00000132170;ENSG00000143365;ENSG00000146648;ENSG00000171105;ENSG00000186951
GO:0004672	protein kinase activity	MF	4	0.014607	0.03188763	ENSG00000105976;ENSG00000128052;ENSG00000146648;ENSG00000171105
GO:0070011	peptidase activity, acting on L-amino acid peptides	MF	4	0.016198	0.03497126	ENSG00000087245;ENSG00000100985;ENSG00000149968;ENSG00000180210
GO:0005178	integrin binding	MF	2	0.016229	0.03497126	ENSG00000128052;ENSG00000146648
GO:0097159	organic cyclic compound binding	MF	16	0.016472	0.03526779	ENSG00000005381;ENSG00000007171;ENSG00000073756;ENSG00000085563;ENSG00000091831;ENSG00000095303;ENSG00000100823;ENSG00000105976;ENSG00000106546;ENSG00000118777;ENSG00000128052;ENSG00000132170;ENSG00000143365;ENSG00000146648;ENSG00000171105;ENSG00000186951
GO:0001094	TFIID-class transcription factor complex binding	MF	1	0.017101	0.03569867	ENSG00000106546
GO:0031994	insulin-like growth factor I binding	MF	1	0.017101	0.03569867	ENSG00000171105
GO:0043560	insulin receptor substrate binding	MF	1	0.017101	0.03569867	ENSG00000171105
GO:0046624	sphingolipid transporter activity	MF	1	0.017101	0.03569867	ENSG00000085563
GO:0016616	oxidoreductase activity, acting on the CH-OH group of donors, NAD or NADP as acceptor	MF	2	0.017372	0.03581675	ENSG00000159228;ENSG00000198074
GO:0031072	heat shock protein binding	MF	2	0.017372	0.03581675	ENSG00000106546;ENSG00000128052
GO:0008233	peptidase activity	MF	4	0.018314	0.03709736	ENSG00000087245;ENSG00000100985;ENSG00000149968;ENSG00000180210
GO:0004032	alditol:NADP+ 1-oxidoreductase activity	MF	1	0.018513	0.03709736	ENSG00000198074
GO:0008296	3'-5'-exodeoxyribonuclease activity	MF	1	0.018513	0.03709736	ENSG00000100823
GO:0016653	oxidoreductase activity, acting on NAD(P)H, heme protein as acceptor	MF	1	0.018513	0.03709736	ENSG00000007171
GO:0042626	ATPase activity, coupled to transmembrane movement of substances	MF	2	0.018549	0.03709736	ENSG00000085563;ENSG00000118777
GO:0016614	oxidoreductase activity, acting on CH-OH group of donors	MF	2	0.019514	0.03868917	ENSG00000159228;ENSG00000198074
GO:0008528	G protein-coupled peptide receptor activity	MF	2	0.019758	0.03868917	ENSG00000163464;ENSG00000178623

GO:0003906	DNA-(apurinic or apyrimidinic site) endonuclease activity	MF	1	0.019924	0.03868917	ENSG00000100823
GO:0019104	DNA N-glycosylase activity	MF	1	0.019924	0.03868917	ENSG00000100823
GO:0036041	long-chain fatty acid binding	MF	1	0.019924	0.03868917	ENSG00000132170
GO:0015405	P-P-bond-hydrolysis-driven transmembrane transporter activity	MF	2	0.020499	0.03957687	ENSG00000085563;ENSG00000118777
GO:0015399	primary active transmembrane transporter activity	MF	2	0.020749	0.03982867	ENSG00000085563;ENSG00000118777
GO:0010181	FMN binding	MF	1	0.021332	0.04071389	ENSG00000007171
GO:0001653	peptide receptor activity	MF	2	0.021506	0.04081207	ENSG00000163464;ENSG00000178623
GO:0043492	ATPase activity, coupled to movement of substances	MF	2	0.022274	0.04203097	ENSG00000085563;ENSG00000118777
GO:0035257	nuclear hormone receptor binding	MF	2	0.022532	0.04219262	ENSG00000091831;ENSG00000132170
GO:0004953	icosanoid receptor activity	MF	1	0.022739	0.04219262	ENSG00000132170
GO:0005159	insulin-like growth factor receptor binding	MF	1	0.022739	0.04219262	ENSG00000171105
GO:0016773	phosphotransferase activity, alcohol group as acceptor	MF	4	0.024142	0.04382479	ENSG00000105976;ENSG00000128052;ENSG00000146648;ENSG00000171105
GO:0043395	heparan sulfate proteoglycan binding	MF	1	0.024143	0.04382479	ENSG00000105426
GO:0046965	retinoid X receptor binding	MF	1	0.024143	0.04382479	ENSG00000132170
GO:0047498	calcium-dependent phospholipase A2 activity	MF	1	0.024143	0.04382479	ENSG00000170890
GO:0052650	NADP-retinol dehydrogenase activity	MF	1	0.026946	0.04864858	ENSG00000198074
GO:0032553	ribonucleotide binding	MF	7	0.027111	0.04868258	ENSG00000007171;ENSG00000085563;ENSG00000105976;ENSG00000118777;ENSG00000128052;ENSG00000146648;ENSG00000171105
GO:0016500	protein-hormone receptor activity	MF	1	0.028345	0.05048773	ENSG00000171105
GO:0005524	ATP binding	MF	6	0.028418	0.05048773	ENSG00000085563;ENSG00000105976;ENSG00000118777;ENSG00000128052;ENSG00000146648;ENSG00000171105
GO:0005515	protein binding	MF	25	0.028833	0.05095332	ENSG00000007171;ENSG00000073756;ENSG00000085563;ENSG00000087245;ENSG00000091831;ENSG00000100823;ENSG00000100985;ENSG00000104267;ENSG00000105426;ENSG00000105976;ENSG00000106546;ENSG00000118777;ENSG00000128052;ENSG00000132170;ENSG00000133742;ENSG00000143365;ENSG00000146648;ENSG00000148680;ENSG00000149968;ENSG00000163464;ENSG00000170890;ENSG00000171105;ENSG00000180210;ENSG00000186951;ENSG00000198074
GO:0016799	hydrolase activity, hydrolyzing N-glycosyl compounds	MF	1	0.031136	0.05473401	ENSG00000100823
GO:0051427	hormone receptor binding	MF	2	0.031747	0.05543193	ENSG00000091831;ENSG00000132170

GO:0004012	phospholipid-translocating ATPase activity	MF	1	0.032529	0.05543193	ENSG00000085563
GO:0016493	C-C chemokine receptor activity	MF	1	0.032529	0.05543193	ENSG00000163464
GO:0016895	exodeoxyribonuclease activity, producing 5'-phosphomonoesters	MF	1	0.032529	0.05543193	ENSG00000100823
GO:0102567	phospholipase A2 activity (consuming 1,2-dipalmitoylphosphatidy lcholine)	MF	1	0.032529	0.05543193	ENSG00000170890
GO:0102568	phospholipase A2 activity consuming 1,2-dioleoylphosphatidylet hanolamine)	MF	1	0.032529	0.05543193	ENSG00000170890
GO:0032559	adenyl ribonucleotide binding	MF	6	0.032936	0.05584061	ENSG00000085563;ENSG00000105976;ENSG00000118777;ENSG00000128052;ENSG00000146648;ENSG00000171105
GO:0030554	adenyl nucleotide binding	MF	6	0.033844	0.0569307	ENSG00000085563;ENSG00000105976;ENSG00000118777;ENSG00000128052;ENSG00000146648;ENSG00000171105
GO:0019957	C-C chemokine binding	MF	1	0.03392	0.0569307	ENSG00000163464
GO:0005516	calmodulin binding	MF	2	0.035098	0.05861444	ENSG00000007171;ENSG00000146648
GO:0004529	exodeoxyribonuclease activity	MF	1	0.035309	0.05867222	ENSG00000100823
GO:0016301	kinase activity	MF	4	0.03719	0.06149265	ENSG00000105976;ENSG00000128052;ENSG00000146648;ENSG00000171105
GO:1901567	fatty acid derivative binding	MF	1	0.038081	0.06239761	ENSG00000132170
GO:0042803	protein homodimerization activity	MF	4	0.038111	0.06239761	ENSG00000007171;ENSG00000073756;ENSG00000106546;ENSG00000118777
GO:0004709	MAP kinase kinase kinase activity	MF	1	0.039464	0.06398531	ENSG00000146648
GO:0042974	retinoic acid receptor binding	MF	1	0.039464	0.06398531	ENSG00000132170
GO:0005520	insulin-like growth factor binding	MF	1	0.040845	0.06558803	ENSG00000171105
GO:0051059	NF-kappaB binding	MF	1	0.040845	0.06558803	ENSG00000100823
GO:0008106	alcohol dehydrogenase (NADP+) activity	MF	1	0.042225	0.06747849	ENSG00000198074
GO:0016702	oxidoreductase activity, acting on single donors with incorporation of molecular oxygen, incorporation of two	MF	1	0.043602	0.06846162	ENSG00000073756
GO:0031624	ubiquitin conjugating enzyme binding	MF	1	0.043602	0.06846162	ENSG00000186951
GO:0043548	phosphatidylinositol 3-kinase binding	MF	1	0.043602	0.06846162	ENSG00000171105
GO:0046982	protein heterodimerization activity	MF	3	0.04366	0.06846162	ENSG00000106546;ENSG00000132170;ENSG00000146648

GO:0016701	oxidoreductase activity, acting on single donors with incorporation of molecular oxygen	MF	1	0.044977	0.06987198	ENSG00000073756
GO:0019956	chemokine binding	MF	1	0.044977	0.06987198	ENSG00000163464
GO:0000166	nucleotide binding	MF	7	0.046115	0.0711176	ENSG00000007171;ENSG00000085563;ENSG00000105976;ENSG00000118777;ENSG00000128052;ENSG00000146648;ENSG00000171105
GO:1901265	nucleoside phosphate binding	MF	7	0.046205	0.0711176	ENSG00000007171;ENSG00000085563;ENSG00000105976;ENSG00000118777;ENSG00000128052;ENSG00000146648;ENSG00000171105
GO:0004623	phospholipase A2 activity	MF	1	0.047723	0.0724518	ENSG00000170890
GO:0004993	G protein-coupled serotonin receptor activity	MF	1	0.047723	0.0724518	ENSG00000148680
GO:0099589	serotonin receptor activity	MF	1	0.047723	0.0724518	ENSG00000148680
GO:0004033	aldo-keto reductase (NADP) activity	MF	1	0.049093	0.07385991	ENSG00000198074
GO:0051393	alpha-actinin binding	MF	1	0.049093	0.07385991	ENSG00000132170
