# Supplementary material for: Mechanism of Fructus Mume Pills Underlying Their Protective Effects in Rats with Acetic Acid-Inducedulcerative Colitis via the Regulation of Inflammatory Cytokines and the VEGF-PI3K/Akt-eNOS Signaling Pathway
Source: Evid Based Complement Alternat Med. 2022 May 2;2022:4621131. doi: 10.1155/2022/4621131 (PMC9129976; doi:10.1155/2022/4621131)
Supplement: Supplementary Materials — Supplement 1 FMP Quality Control Methods and Results. Supplement 2 FMP Active Compounds and Targets Supplement 3 Ulcerative Colitis Targets Supplement 4 GO Enrichment Result Supplement 5 KEGG Enrichment Results. [file 4621131.f1.zip › 4621131.f1/Supplement 5 KEGG Enrichment Results.pdf]

Table 1 KEGG enrichment results

| Term | Count | PValue | Genes |
|---|---|---|---|
| hsa04060:Cytokine-cytokine receptor interaction | 104 | 3.42E-42 | OSMR, IL18, IL19, TGFB3, TNFSF15, IL13, IL15, CXCL12, IL10, TGFB1, CXCL10, TGFB2, IL11, IFNG, IL1B, IL15RA, FAS, IL1A, EGFR, IL26, CCL4L1, TNFRSF17, CCL4L2, TNFRSF14, IL24, CD40, IL21, IL22, IL20, CCR9, CCR7, CCR6, CCR5, CD40LG, CCR3, VEGFA, CX3CR1, PDGFRB, TNFRSF6B, IL1R2, IL1R1, CCL3, CCL2, CCL8, CX3CL1, CCL5, CCL4, IL17A, IFNA1, IL17B, IL23A, EGF, IL23R, TGFBR2, MET, HGF, KDR, GH1, CXCL13, CXCL16, BMP7, LEPR, CXCR1, CXCR2, CXCR3, TNFRSF11A, CXCR4, IL4R, CXCR6, IFNGR2, CSF2RA, LTA, IL18RAP, IL6R, TNFRSF9, TNFSF13B, IL12B, CXCL1, TNF, CXCL5, CXCL6, IL7R, CCL26, CCL24, CCL25, TNFRSF1A, TNFRSF1B, CCL20, CCL21, IL10RB, IL10RA, IL4, IL18R1, IL3, IL6, IL2RA, FLT1, IL5, IL7, IL9, CCL11, LEP, TSLP, IFNA13, IL2 |
| hsa04672:Intestinal immune network for IgA production | 30 | 2.48E-18 | HLA-DQB1, HLA-DRB1, HLA-DRB3, TGFB3, IL15, CXCL12, TGFB1, IL10, TGFB2, CCL25, CXCR4, ITGB7, IL15RA, CD28, IL4, IL6, IL5, TNFRSF17, ITGA4, CD40, CCR9, CD86, TNFSF13B, CD80, CD40LG, MADCAM1, AICDA, ICOSLG, HLA-DRA, IL2 |
| hsa04620:Toll-like receptor signaling pathway | 39 | 5.66E-15 | CCL3, TNF, TOLLIP, TLR1, TLR2, NFKBIA, TLR3, TLR4, NFKB1, TLR5, TLR6, CCL5, CCL4, TLR7, CXCL10, TLR9, FOS, IFNA1, MYD88, MAP3K8, RAC1, IL1B, MAP2K7, SPP1, IRAK1, IL6, MAP2K2, RELA, CD40, STAT1, MAPK1, CD86, IRF5, CD80, MAPK14, RIPK1, IL12B, IFNA13, CD14 |
| hsa05200:Pathways in cancer | 77 | 6.09E-15 | HRAS, FGF7, PTGS2, STAT5A, MMP9, PPARG, TGFB3, MLH1, NFKB1, NFKB2, MMP2, MMP1, TGFB1, GLI1, CTNNB1, TGFB2, FOS, CASP3, CDKN2A, TGFA, NOS2, HHIP, FAS, FGF2, CSF2RA, EGFR, HSP90AA1, BRAF, RELA, TP53, RAD51, PRKCB, MAPK1, SMO, CCND1, VEGFA, PDGFRB, MDM2, LAMC2, WNT11, GSTP1, DCC, EGLN3, NFKBIA, CDH1, TCF7L2, ITGB1, TPM3, KRAS, BCL2, RAC1, EGF, LAMB1, APC, IL6, MSH2, MAP2K2, TGFBR2, MET, FZD1, SMAD3, FZD3, HGF, FZD5, STAT1, BIRC3, FZD4, BIRC2, STAT3, CDKN1A, HSP90B1, ETS1, RASSF1, BAX, PLCG2, MTOR, CRK |
| hsa04630:Jak-STAT signaling pathway | 49 | 9.33E-15 | OSMR, STAT5A, LEPR, IL19, IL13, IL15, IL7R, IL10, IL11, STAT6, STAT4, IFNA1, IL23A, IL10RB, IL4R, IL10RA, IFNG, IL15RA, IFNGR2, CSF2RA, IL13RA2, IL4, IL3, IL6, IL2RA, IL23R, IL5, IL7, SOCS3, SOCS1, IL9, IL26, IL6R, IL24, IL21, STAT1, IL22, STAT3, IL20, PTPN11, LEP, TYK2, GH1, TSLP, CCND1, JAK2, IL12B, IFNA13, IL2 |
| hsa04062:Chemokine signaling pathway | 52 | 3.72E-13 | ADCY3, HRAS, ADCY7, CXCR1, CXCR2, NFKB1, FOXO3, CXCR3, CXCL12, CXCL10, CXCR4, CXCR6, PAK1, LYN, BRAF, RELA, CCL4L1, CCL4L2, PRKCB, CCR9, MAPK1, CCR7, CCR6, ARRB2, CCR5, CCR3, CX3CR1, CXCL1, PARD3, CCL3, CCL2, CXCL5, CCL8, NFKBIA, CXCL6, CX3CL1, CCL5, CCL4, CCL26, CCL24, CCL25, KRAS, CCL20, CCL21, RAC1, STAT1, STAT3, CCL11, CXCL13, CXCL16, RAP1A, JAK2, CRK |
| hsa05330:Allograft rejection | 21 | 2.86E-12 | HLA-DQB1, IL4, TNF, IL5, HLA-DRB1, HLA-DRB3, HLA-A, GZMB, CD40, HLA-B, IL10, HLA-G, CD86, CD80, CD40LG, IFNG, FAS, IL12B, IL2, CD28, HLA-DRA |
| hsa04514:Cell adhesion molecules (CAMs) | 41 | 3.97E-12 | HLA-DQB1, ITGAL, CLDN7, CLDN18, HLA-DRB1, CLDN4, HLA-DRB3, CDH1, CDH3, ITGB1, PDCD1, ITGAM, VCAM1, ITGB7, CD6, CD28, ICAM1, F11R, PTPRC, MPZ, SELL, ICAM2, CTLA4, HLA-A, HLA-B, ITGA4, CD40, HLA-G, SDC1, CD86, CD80, CD34, CD40LG, PECAM1, CLDN1, CLDN2, VCAN, MADCAM1, CD226, ICOSLG, HLA-DRA |
| hsa04621:NOD-like receptor signaling pathway | 27 | 6.87E-12 | CXCL1, TNF, CCL2, IL18, NFKBIA, CCL8, NFKB1, CCL5, NOD2, NOD1, MEFV, IL1B, CASP1, IL6, CARD8, HSP90AA1, CARD9, RELA, BIRC3, NLRP3, BIRC2, CCL11, MAPK1, HSP90B1, MAPK14, RIPK2, TNFAIP3 |
| hsa04940:Type I diabetes mellitus | 20 | 1.05E-09 | HLA-DQB1, TNF, HLA-DRB1, INS-IGF2, HLA-DRB3, HLA-A, IGF2, GZMB, HLA-B, HLA-G, CD86, CD80, IFNG, IL1B, FAS, IL12B, IL1A, LTA, IL2, CD28, HLA-DRA |
| hsa05320:Autoimmune thyroid disease | 22 | 1.15E-09 | HLA-DQB1, TG, IL4, IL5, HLA-DRB1, HLA-DRB3, CTLA4, HLA-A, GZMB, CD40, HLA-B, IL10, HLA-G, IFNA1, CD86, CD80, CD40LG, FAS, IFNA13, IL2, CD28, HLA-DRA |
| hsa05210:Colorectal cancer | 28 | 3.35E-09 | DCC, TGFB3, MLH1, TCF7L2, TGFB1, TGFB2, CTNNB1, FOS, CASP3, KRAS, BCL2, RAC1, APC, EGFR, BRAF, MSH2, TGFBR2, MET, FZD1, TP53, SMAD3, FZD3, FZD5, FZD4, MAPK1, CCND1, BAX, PDGFRB |
| hsa05332:Graft-versus-host disease | 18 | 1.63E-08 | HLA-DQB1, IL6, TNF, HLA-DRB1, HLA-DRB3, HLA-A, GZMB, HLA-B, HLA-G, CD86, CD80, IFNG, IL1B, FAS, IL1A, IL2, CD28, HLA-DRA |
| hsa04640:Hematopoietic cell lineage | 27 | 2.79E-08 | IL4, IL3, IL1R2, IL1R1, IL6, IL2RA, IL5, TNF, HLA-DRB1, IL7, HLA-DRB3, IL6R, ITGA4, IL7R, ITGAM, IL11, CD55, CD19, CD44, CD34, IL4R, CD33, IL1B, IL1A, CSF2RA, CD14, HLA-DRA |
| hsa05219:Bladder cancer | 18 | 6.23E-08 | EGFR, HRAS, BRAF, MAP2K2, MMP9, TP53, CDH1, MMP2, MMP1, MAPK1, CCND1, CDKN1A, CDKN2A, KRAS, RASSF1, VEGFA, MDM2, EGF |
| hsa04010:MAPK signaling pathway | 52 | 2.81E-07 | HRAS, FGF7, GNA12, TGFB3, NFKB1, NFKB2, TGFB1, TGFB2, FOS, CASP3, MAP3K8, PLA2G1B, IL1B, FAS, PAK1, MAP2K7, FGF2, IL1A, EGFR, BRAF, RELA, TP53, FLNB, PRKCB, MAPK1, ARRB2, PLA2G2A, PLA2G6, PDGFRB, NGF, IL1R2, IL1R1, TNF, MKNK2, HSPA1A, HSPA1B, HSPA1L, TNFRSF1A, KRAS, HSPA2, DUSP16, RAC1, NFATC2, EGF, NTF3, MAP2K2, TGFBR2, NR4A1, DUSP1, MAPK14, RAP1A, CRK, CD14 |
| hsa05310:Asthma | 14 | 5.59E-07 | IL4, HLA-DQB1, IL3, IL5, TNF, HLA-DRB1, HLA-DRB3, IL9, IL13, CD40, IL10, CCL11, CD40LG, HLA-DRA |
| hsa05212:Pancreatic cancer | 22 | 1.20E-06 | EGFR, BRAF, ARHGEF6, RELA, TGFBR2, TP53, TGFB3, SMAD3, NFKB1, STAT1, TGFB1, STAT3, TGFB2, RAD51, MAPK1, CCND1, CDKN2A, KRAS, VEGFA, RAC1, TGFA, EGF |
| hsa04660:T cell receptor signaling pathway | 27 | 3.76E-06 | IL4, PTPRC, HRAS, TNF, IL5, MAP2K2, RELA, CTLA4, NFKBIA, NFKB1, IL10, PDCD1, MAPK1, PRKCQ, FOS, KRAS, CD40LG, MAPK14, MAP3K8, IFNG, PAK1, GRAP2, NFATC2, MAP2K7, IL2, CD28, NFATC1 |
| hsa05215:Prostate cancer | 24 | 3.80E-06 | EGFR, HRAS, HSP90AA1, BRAF, INS-IGF2, MAP2K2, RELA, TP53, NFKBIA, IGF2, NFKB1, TCF7L2, CTNNB1, MAPK1, HSP90B1, CCND1, CDKN1A, KRAS, BCL2, MDM2, TGFA, PDGFRB, MTOR, EGF, GSTP1 |
| hsa04722:Neurotrophin signaling pathway | 29 | 6.08E-06 | YWHAZ, HRAS, NFKBIA, NFKB1, FOXO3, IRAK3, KRAS, BCL2, RAC1, SH2B3, MAP2K7, CAMK2A, ARHGDIA, IRAK2, IRAK1, NTF3, BRAF, MAP2K2, RELA, TP53, PTPN11, MAPK1, MAPK14, BAX, PLCG2, RAP1A, RIPK2, CRK, NGF |

| | | | |
|---|---|---|---|
| hsa04650:Natural killer cell mediated cytotoxicity | 30 | 8.63E-06 | ITGAL, HRAS, MICB, MICA, TNF, IFNA1, CASP3, KRAS, RAC1, IFNG, FAS, PAK1, FCGR3A, NFATC2, IFNGR2, FCGR3B, NFATC1, ICAM1, BRAF, MAP2K2, ICAM2, HLA-A, GZMB, HLA-B, HLA-G, PTPN11, PRKCB, MAPK1, PLCG2, IFNA13 |
| hsa04210:Apoptosis | 23 | 9.07E-06 | IRAK2, CFLAR, IRAK1, IL3, IL1R1, TNF, RELA, TP53, NFKBIA, NFKB1, BIRC3, BIRC2, TNFRSF1A, IRAK3, CASP3, MYD88, BAX, BCL2, RIPK1, IL1B, FAS, IL1A, NGF |
| hsa05220:Chronic myeloid leukemia | 21 | 9.83E-06 | HRAS, BRAF, MAP2K2, STAT5A, RELA, TGFBR2, TP53, TGFB3, NFKBIA, SMAD3, NFKB1, TGFB1, PTPN11, TGFB2, MAPK1, CCND1, CDKN1A, CDKN2A, KRAS, MDM2, CRK |
| hsa05216:Thyroid cancer | 12 | 2.94E-05 | MAPK1, HRAS, CCND1, KRAS, BRAF, MAP2K2, PPARG, TP53, CDH1, TCF7L2, CTNNB1, TPM3 |
| hsa05214:Glioma | 18 | 3.88E-05 | EGFR, HRAS, BRAF, MAP2K2, TP53, PRKCB, MAPK1, CCND1, CDKN1A, CDKN2A, KRAS, PLCG2, MDM2, TGFA, PDGFRB, MTOR, EGF, CAMK2A |
| hsa05211:Renal cell carcinoma | 19 | 4.67E-05 | HRAS, BRAF, MAP2K2, MET, EGLN3, TGFB3, HGF, TGFB1, PTPN11, TGFB2, MAPK1, KRAS, ETS1, VEGFA, RAC1, RAP1A, TGFA, PAK1, CRK |
| hsa05223:Non-small cell lung cancer | 16 | 7.68E-05 | EGFR, FHIT, HRAS, BRAF, MAP2K2, TP53, FOXO3, PRKCB, MAPK1, CCND1, CDKN2A, KRAS, RASSF1, PLCG2, TGFA, EGF |
| hsa04623:Cytosolic DNA-sensing pathway | 16 | 9.68E-05 | IL6, IL18, RELA, NFKBIA, CCL4L1, CCL4L2, NFKB1, IL33, CCL5, CCL4, CXCL10, DDX58, IFNA1, RIPK1, IL1B, IFNA13, CASP1 |
| hsa05120:Epithelial cell signaling in Helicobacter pylori infection | 18 | 1.11E-04 | EGFR, CXCL1, F11R, LYN, RELA, MET, NFKBIA, CXCR1, CXCR2, NFKB1, CCL5, PTPN11, CASP3, NOD1, MAPK14, PLCG2, RAC1, PAK1 |
| hsa04670:Leukocyte transendothelial migration | 25 | 1.66E-04 | ITGAL, ICAM1, CLDN7, F11R, CLDN18, CLDN4, MMP9, NOX1, ITGA4, ITGB1, CXCL12, MMP2, ITGAM, PRKCB, PTPN11, CTNNB1, VCAM1, CXCR4, MAPK14, PLCG2, PECAM1, RAC1, CLDN1, CLDN2, RAP1A |
| hsa05213:Endometrial cancer | 15 | 1.92E-04 | EGFR, HRAS, BRAF, MAP2K2, TP53, MLH1, CDH1, FOXO3, TCF7L2, CTNNB1, MAPK1, CCND1, KRAS, EGF, APC |
| hsa05416:Viral myocarditis | 18 | 1.96E-04 | HLA-DQB1, ITGAL, ICAM1, HLA-DRB1, HLA-DRB3, HLA-A, CD40, HLA-B, HLA-G, CD55, CASP3, CCND1, CD86, CD80, CD40LG, RAC1, CD28, HLA-DRA |
| hsa05218:Melanoma | 18 | 1.96E-04 | EGFR, HRAS, FGF7, BRAF, MAP2K2, MET, TP53, CDH1, HGF, MAPK1, CCND1, CDKN1A, CDKN2A, KRAS, PDGFRB, MDM2, EGF, FGF2 |
| hsa04370:VEGF signaling pathway | 18 | 3.96E-04 | HRAS, PTGS2, MAP2K2, SPHK1, KDR, PRKCB, MAPK1, KRAS, MAPK14, VEGFA, PLCG2, RAC1, PLA2G1B, PLA2G2A, PLA2G6, NOS3, NFATC2, NFATC1 |
| hsa04664:Fc epsilon RI signaling pathway | 18 | 6.43E-04 | IL4, IL3, HRAS, TNF, IL5, LYN, MAP2K2, IL13, PRKCB, MAPK1, KRAS, MAPK14, PLCG2, RAC1, PLA2G1B, PLA2G2A, PLA2G6, MAP2K7 |
| hsa04920:Adipocytokine signaling pathway | 16 | 9.86E-04 | PPARA, TNF, SOCS3, LEPR, RELA, NFKBIA, NFKB1, STAT3, PTPN11, LEP, PRKCQ, TNFRSF1A, TNFRSF1B, ACSL1, JAK2, MTOR |
| hsa04012:ErbB signaling pathway | 18 | 0.002306974 | EGFR, HRAS, BRAF, MAP2K2, STAT5A, RPS6KB1, PRKCB, MAPK1, CDKN1A, KRAS, PLCG2, TGFA, MTOR, PAK1, EGF, MAP2K7, CRK, CAMK2A |
| hsa04612:Antigen processing and presentation | 17 | 0.003542604 | HLA-DQB1, HSP90AA1, HLA-DRB1, HLA-DRB3, HLA-A, HSPA1A, HSPA1B, HLA-B, HLA-G, CD74, B2M, HSPA1L, IFNA1, HSPA2, HSPA4, IFNA13, LTA, HLA-DRA |
| hsa05020:Prion diseases | 10 | 0.003775901 | EGR1, MAPK1, IL6, NOTCH1, MAP2K2, BAX, IL1B, HSPA1A, HSPA1B, CCL5, IL1A |
| hsa05221:Acute myeloid leukemia | 13 | 0.006123592 | HRAS, BRAF, MAP2K2, RELA, STAT5A, NFKB1, RPS6KB1, TCF7L2, STAT3, MAPK1, CCND1, KRAS, MTOR |
| hsa04662:B cell receptor signaling pathway | 15 | 0.008290603 | HRAS, LYN, MAP2K2, RELA, NFKBIA, NFKB1, PRKCB, MAPK1, FOS, KRAS, CD19, PLCG2, RAC1, NFATC2, NFATC1 |
| hsa04730:Long-term depression | 14 | 0.009964289 | HRAS, GNAO1, BRAF, LYN, MAP2K2, GNA12, PRKCB, CRHR1, MAPK1, KRAS, CRH, PLA2G1B, PLA2G2A, PLA2G6 |

| | | | |
|---|---|---|---|
| hsa05217:Basal cell carcinoma | 12 | 0.011030698 | SMO, TP53, FZD1, FZD3, WNT11, HHIP, FZD5, FZD4, TCF7L2, GLI1, CTNNB1, APC |
| hsa05014:Amyotrophic lateral sclerosis (ALS) | 11 | 0.022242682 | TNFRSF1A, CASP3, TNFRSF1B, TNF, MAPK14, BCL2, BAX, RAC1, TP53, CAT, CASP1 |
| hsa04666:Fc gamma R-mediated phagocytosis | 16 | 0.028030163 | PTPRC, LYN, SPHK1, ASAP2, RPS6KB1, PRKCB, MAPK1, ARPC2, PLCG2, RAC1, PLA2G6, FCGR2A, PAK1, FCGR3A, CRK, DNM2 |
| hsa04510:Focal adhesion | 28 | 0.031188708 | HRAS, ITGB1, CTNNB1, BCL2, ITGB7, RAC1, PAK1, LAMB1, EGF, SPP1, EGFR, FLT1, BRAF, MET, ITGA4, HGF, BIRC3, FLNB, BIRC2, KDR, PRKCB, MAPK1, CCND1, VEGFA, RAP1A, PDGFRB, LAMC2, CRK |
| hsa05322:Systemic lupus erythematosus | 16 | 0.038852647 | HLA-DQB1, TNF, HLA-DRB1, HLA-DRB3, CD40, IL10, CD86, CD80, CD40LG, IFNG, H2AFX, FCGR2A, FCGR3A, FCGR3B, HLA-DRA, CD28 |
| hsa04916:Melanogenesis | 16 | 0.038852647 | ADCY3, HRAS, GNAO1, ADCY7, MAP2K2, FZD1, FZD3, FZD5, FZD4, TCF7L2, CTNNB1, PRKCB, MAPK1, KRAS, WNT11, CAMK2A |
| hsa05222:Small cell lung cancer | 14 | 0.045100052 | FHIT, PTGS2, RELA, TP53, NFKBIA, NFKB1, BIRC3, BIRC2, ITGB1, CCND1, BCL2, LAMC2, NOS2, LAMB1 |
